# Bioresponsive *pseudo*Glucosinolates (*ps*GSLs) Release Isothiocyanates (ITCs) in the Presence of Nitroreductases

**DOI:** 10.1002/chem.71012

**Published:** 2026-04-17

**Authors:** Claire C. Jimidar, Charity S. G. Ganskow, Mervic D. Kagho, Aishi Chakrabarti, Lorenz Wiese, Michael Zollo, Ulrike Beutling, Leona C. Cesar, Julia Morud, Kamila Bugaj, Mark Brönstrup, Stephan A. Sieber, Stephan M. Hacker, Philipp Klahn

**Affiliations:** ^1^ Department of Chemistry and Molecular Biology University of Gothenburg Gothenburg Sweden; ^2^ Institute of Organic Chemistry Technische Universität Braunschweig Braunschweig Germany; ^3^ Center for Functional Protein Assemblies Technische Universität München Garching Germany; ^4^ Department for Chemical Biology Helmholtz Center for Infection Research Braunschweig Germany; ^5^ Department of Molecular Physiology, Leiden Institute of Chemistry Leiden University Leiden The Netherlands

**Keywords:** artificial glucosinolates, bioresponsive protein labelling, isothiocyanates, prodrugs, pseudoglucosinolates

## Abstract

Glucosinolates (GSLs) are plant secondary metabolites that release bioactive isothiocyanates (ITCs) upon myrosinase‐mediated activation. While ITCs display diverse antimicrobial and chemoprotective activities, their application is limited by dependence on myrosinase and intrinsic hydrolytic instability. Here, we introduce pseudoglucosinolates (psGSLs), a synthetic platform that mimics the natural GSL activation mechanism but replaces the thioglucosidic trigger with an enzyme‐responsive *para*‐aminobenzylthiol motif. Using nitroreductase (NfsB) as a noncanonical activating enzyme, we synthesized and characterized a series of nitro‐masked psGSLs, including azide‐, alkyne‐, and fluorophore‐functionalized derivatives. Enzymatic reduction induces a self‐immolative 1,6‐elimination and subsequent thio‐Lossen rearrangement, releasing ITCs under physiological conditions. The liberated ITCs covalently modify peptides and proteins, showing predominant lysine reactivity in chemoproteomic analyses of the *Staphylococcus aureus* proteome, including functional sites of essential proteins. Fluorescent probes enabled visualization of enzyme‐dependent protein labeling and demonstrated nitroreductase‐triggered ITC release in vivo in *Caenorhabditis elegans*. Together, psGSLs establish a modular, bioresponsive prodrug and chemical biology platform for enzyme‐controlled ITC delivery, expanding the scope of ITC‐based covalent modification beyond natural myrosinase‐dependent systems.

## Graphical abstract

This work introduces the concept of *pseudo*glucosinolates (psGSLs) and reports the synthesis and evaluation of nitroreductase‐responsive *ps*GSLs. These compounds represent a complementary prodrug strategy to natural glucosinolates (GSLs) for the controlled release of isothiocyanates (ITCs), enabling bio‐responsive protein labeling, as demonstrated in living *C. elegans*.

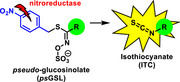

## 1 Introduction

**SCHEME 1:**
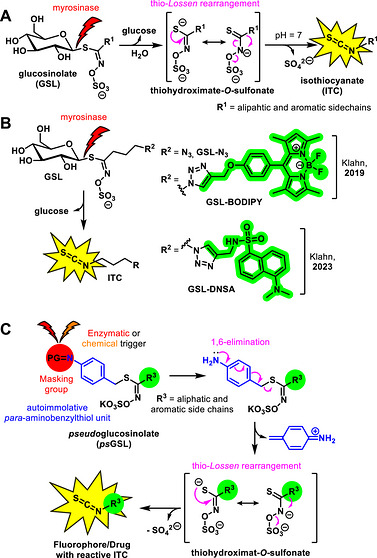
(A): GSL breakdown by myrosinase in plants of the order Brassicales. (B): Artificial, fluorescent and azide containing GSLs by *Klahn* and coworkers [38, 39]. (C): Design concept of novel *pseudo*glucosinolates (*ps*GSLs) [40]. PG = protective group.

Glucosinolates (GSLs) are secondary metabolites produced by plants of the order *Brassicales*, such as broccoli, horse radish or white mustard as part of the GSL‐myrosinase herbivore defense system [1]. Chemically, GSLs are glycosidic thiohydroximate‐*O*‐sulfonates biogenetically derived from amino acids, which are stored in the tissue of the producing plants separated from the enzyme myrosinase, a thioglucosidase. Upon tissue damage, the thioglycosidic bond of the GSLs is cleaved by the myrosinase releasing glucose and a thiohydroximate‐*O*‐sulfonate aglycone [2], which undergoes a subsequent thio‐*Lossen* rearrangement forming isothiocyanates (ITCs) as active feeding deterrent agents (Scheme 1A) [3, 4]. Beyond their feeding deterrent properties, ITCs have raised interest in recent years due to multiple interesting biological activities such as pollinator attraction [5], antimicrobial activities [6, 7] against bee‐colony infecting fungi *Nosema ceranae* [8, 9]. Methicillin‐resistant *Staphylococcus aureus* (MRSA) [10], enterohemorrhagic *Escherichia coli* (EHEC) [11], *Pseudomonas aeruginosa*, and related biofilms [12]. In addition, multiple specific bioactivities in mammalian cells have been reported, which might be summarized as neuroprotective, cardioprotective, and chemoprotective [13, 14, 15, 16, 17, 18]. Most of the biological activities of ITCs seem to be associated with their electrophilic reaction with biological nucleophiles [11, 19, 20]. GSLs as naturally occurring ITC‐releasing molecules have therefore gained interest as intestinal formation of ITCs from GSLs is known to mediate the health beneficiary effects of *Brassicales* enriched dietary [21, 22, 23].

Considering their interesting biological activities, it is not surprising that natural [17, 24, 25, 26, 27, 28] as well as artificial GSLs have been synthesized including representatives bearing unnatural [17, 28, 29] or isotopically labeled [29, 30, 31, 32, 33] aglycones and glucose units as well as α‐anomeric GSLs [34, 35] Furthermore, artificial GSLs have been designed to serve as myrosinase‐responsive tool compounds in chemical biology. In 2018, *Tatibouёt* and coworkers, reported the synthesis of mannoside‐GSL conjugates, which allowed for myrosinase‐triggered specific labeling of lectins [36] with released ITCs or synthesis of glycosylated proteins [37].

In 2019, we reported the synthesis and biochemical evaluation of **GSL‐N_3_** and **GSL‐BODIPY** (Scheme 1B), the first examples of an azide bearing and artificial, fluorescent GSL, respectively, forming fluorescent **ITC‐BODIPY** in the presence of myrosinase [38]. A concept which was later used by *Lang* et al. to fluorescently label mammalian gut bacteria in vivo [41].

Independently, *Tatibouёt* and coworkers, reported a similar approach for fluorescent GSLs and demonstrated their ability to serve as labeling probes for proteins [42]. Recently, we have reported also the synthesis of **GSL‐DNSA** (Scheme 1B), which was utilized to image uptake through GTR receptors of *Arabidopsis thaliana* [39]. The major limitation for the application of these probes in broader chemical biology context is the dependence on myrosinase, a thioglycosidase with limited existence in plants. Beyond that ITCs are hydrolysis‐sensitive electrophiles showing bad pharmacokinetic properties leaving their interesting biological activities unexploited [1, 43, 44].

Within our attempts to design bio‐responsive molecular entities [45, 46, 47, 48, 49, 50, 51] and explore the chemistry of artificial GSLs [38, 39, 40, 52], we aimed to translate the natural myrosinase‐mediated release mechanism of ITCs from GSLs (Scheme 1A) toward artificial GSLs, which are bioresponsive toward noncanonical enzymes or even chemical triggers.

Therefore, we envisaged the design of artificial GSLs, in which the thioglycosidic trigger is substituted by a chemically masked *para*‐aminobenzylthiol unit (Scheme 1C), which we named *pseudo*glucosinolates (*ps*GSLs) [40, 52]. Upon enzymatic or chemical removal of the masking group *ps*GSLs were planned to mimic the natural release mechanism of ITCs from GSLs by undergoing an auto‐immolative 1,6‐elimination of the free *para*‐aminobenzylthiol moiety, leading to the thiohydroximate‐*O*‐sulfonate as intermediate, followed by the final formation of ITCs through a thio‐*Lossen* rearrangement. Thus, *ps*GSLs could serve as prodrugs and tool compounds for the release of ITCs and their response might be adjusted to different noncanonical enzymes and chemical triggers. Within this proof‐of‐principle study, we aimed to showcase the general *ps*GSL concept within *ps*GSLs releasing ITCs upon reaction with nitroreductases by masking the 1,6‐elimination reactivity by a nitro function.

Nitroreductases‐responsive prodrugs [53, 54, 55] and imaging probes [56, 57] based on *para*‐nitrobenzyl moieties have been investigated earlier for treatment and imaging of cancer and bacteria, but probes releasing ITCs in the presence of nitroreductases are unknown to date. In this context, the bacterial nitroreductase NfsB, which plays a role in susceptibility and resistance in Gram‐negative pathogens, is of particular interest as a potential enzyme for triggering the release of ITCs from *ps*GSL probes [58, 59].

## 2 Result and Discussion

**SCHEME 2:**
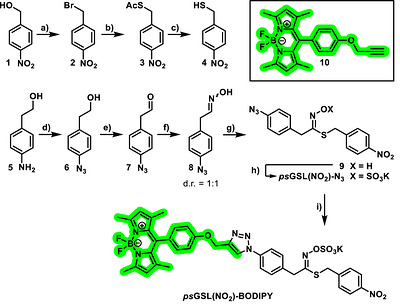
Synthesis of *para*‐nitrobenzylthiol (**4**) and potentially nitroreductase‐responsive *ps*GSLs, namely *ps*GSL(NO_2_)‐N_3_ and *ps*GSL(NO_2_)‐BODIPY. (A) PPh_3_ (1.1 equiv), Br_2_ (1.25 equiv), (CH_2_Cl_2_), ‐78 – 28°C, 10 min, 95%; (B) KSAc (1.5 equiv), (DMF), 23°C, 18 h, 97%; (C) HCl (1.0 equiv, 0.5 M in MeOH), (MeOH), 60°C, 16 h, 97%; (D) NaNO_2_ (1.5 equiv), NaN_3_ (4.0 equiv), (6 M HCl aq.), 0°C, 2.5 h, 95%; (E) IBX (1.1 equiv), (DMSO), 23°C, 17 h, 45%; (F) HONH_2_^.^HCl (1.2 equiv), NaOAc (1.0 equiv), (H_2_O:MeCN/1:3), 23°C, 26 h, 92%; (G) 1) NCS (1.05 equiv), (DMF), 23°C, 1 h, 2) **4** (1.24 equiv), DiPEA (6.0 equiv), (THF), 23°C, 26 h, 68%; (H) 1) SO_3_^.^Py (5.0 equiv), Py (10 equiv), (THF), 23°C, 2 h, 2) KHCO3 (2 M aq., 1.6 equiv), 23°C, 1 h, 57%; (I) TBTA (0.1 equiv), NaAsc (0.2 equiv), Cu(II)SO_4_ (0.05 equiv),(DMSO:MeOH:THF:H_2_O/2:1:1:1), 23°C, 25 h, 46%.

In order to prove our design concept of *ps*GSLs, we first aimed to synthesize potentially nitroreductase‐responsive *ps*GSLs ***ps*GSL(NO_2_)‐N_3_** and fluorescently labelled ***ps*GSL(NO_2_)‐BODIPY** bearing a nitro‐masked *para*‐aminobenzylthiol unit as outlined in Scheme 2. For this purpose, commercially available *para*‐nitrobenzylalcohol (**1**) was converted into thiol (**4**), which served as nitro‐masked *para*‐aminobenzylthiol building block. Initial *Appel*‐type reaction at ‐78°C gave access to bromide **2**, which was subsequently substituted in the presence of potassium thioacetate to form the corresponding thioacetate **3**. Hydrolysis with methanolic HCl under an argon atmosphere provided the desired *para*‐nitrobenzyl thiol (**4**). While basic conditions for the cleavage of the thioacetate led to formation of the respective disulfide, hydrolysis with methanolic HCl under an argon atmosphere provided the desired *para*‐nitrobenzyl thiol (**4**) in 89% yield over 3 steps. As building block for the “*pseudo*‐aglycone,” we synthesized azido oxime **8** starting from the commercially available 2‐(4‐aminophenyl)ethanol **5**. Formation of the diazonium salt and subsequent substitution with sodium azide gave access to azido alcohol **6**, which was oxidized to the corresponding azido aldehyde **7** using 2‐iodoxybenzoic acid (IBX), and final conversion with hydroxylamine led to azido oxime **8** in 39% yield over 3 steps. Both building blocks, **8** and **4**, were coupled adapting the classical approach for the synthesis of GSLs [24, 28, 38, 60]. First azido oxime **8** was converted in situ into its respective chloro oxime, and subsequently the substitution of the chloro atom with thiol **4** in the presence of *Hünig's* base facilitated formation of thiohydroximate **9** in 68% yield.

Treatment of **9** with pyridine SO_3_ complex and subsequent stirring with aqueous potassium bicarbonate solution gave access to ***ps*GSL(NO_2_)‐N_3_** in form of its potassium salt in 57% yield. Subsequent copper(I)‐catalyzed azide‐alkyne cycloaddition click reaction (CuAAC) with *meso*‐substituted, alkyne‐bearing BODIPY dye **10** [38] led to formation of the fluorescently labelled potential nitroreductase‐responsive *ps*GSL, namely ***ps*GSL(NO_2_)‐BODIPY**, in 46% yield.

**SCHEME 3:**
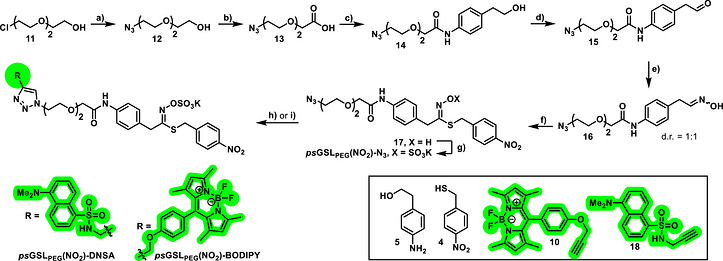
Synthesis of ***ps*GSL_PEG_(NO_2_)‐N_3_, *ps*GSL_PEG_(NO_2_)‐BODIPY** and ***ps*GSL_PEG_(NO_2_)‐DNSA**. (A) NaN_3_ (2.03 equiv), (H_2_O), 115°C, 8 h, 95%; (B) Jones reagent (3.0 M CrO3, 3.0 equiv), (Acetone), 0–23°C, 2 h, 92%; (C) 1) iBuO(CO)Cl (1.1 equiv), NMM (1.1 equiv), (THF), ‐78°C, 7 h, 2) **5** (1.2 equiv), NMM (1.2 equiv), ‐78‐23°C, 18 h, 63%; (D) IBX (1.1 equiv), (DMSO), 23°C, 3 h, 86%; (E) H_2_NOH HCl (1.2 equiv), NaOAc (1.0 equiv), (H_2_O:MeCN/1:3), 23°C, 17 h, 98%; (F) 1) NCS (1.05 equiv); (DMF), 23°C, 19 h, 2) **4** (1.24 equiv), DiPEA (6.0 equiv), (THF), 23°C, 7 h, 91%; (G) 1) SO_3_ Py (5.0 equiv), Py (10.0 equiv), (CH_2_Cl_2_), 60°C, 4 h, 2) KHCO_3_ aq. 2 M (16 equiv), 23°C, 30 min, 61%; (H) **10** (1.2 equiv), Cu(II)SO_4_ (0.05 equiv), TBTA (0.1 equiv), NaAsc (0.2 equiv), (DMSO:H2O:MeOH:THF/5:2:1:1), 25°C, 3 h, 100%; (I) **18** (1.2 equiv), Cu(II)SO_4_ (0.05 equiv), TBTA (0.1 equiv), NaAsc (0.2 equiv), (DMSO:H_2_O/5:1), 25°C, 6 h, 83%.

Unfortunately, although ***ps*GSL(NO_2_)‐N_3_** was water soluble, the *O*‐sulfonate moiety was not sufficient to enable water solubility of ***ps*GSL(NO_2_)‐BODIPY**, a requirement for the biochemical evaluation of the probe in the presence of nitroreductases. As the incorporation of water‐soluble groups to the aromatic core of the *para*‐aminobenzylthiol unit turned out to be synthetically challenging and flexibility for different fluorescent dyes was desired, we incorporated a PEG_2_ motif into the core structure as outlined in Scheme 3. To this end, chloride **11** was converted to azide **12** in the presence of sodium azide at 115°C and subsequent *Jones* oxidation gave the ω‐azido PEG_2_ carboxylic acid **13**. *Iso*‐butylchloro formate mediated amide coupling with aniline **5** gave alcohol **14** in 63% yield. Oxidation to the aldehyde **15** worked in 86% using IBX. Oxime formation, chlorination, coupling with *para*‐nitrophenylthiol **4** and final installation of the *O*‐sulfonate proceeded smoothly and gave ***ps*GSL_PEG_(NO_2_)‐N_3_**. The CuAAC with the dansylamide **18** and BODIPY dye **10**, both bearing a terminal alkyne, provided access to ***ps*GSL_PEG_(NO_2_)‐DNSA** and ***ps*GSL_PEG_(NO_2_)‐BODIPY**, respectively, both showing decent water solubility. All synthesized *ps*GSLs showed decent chemical stability over months stored at ‐20°C or over weeks stored at 25°C being dissolved in either DMSO or HEPES buffer (pH 7.4) containing 10% of DMSO (Compare Figure S2).

Next, we tested our hypothesis of ITC release from ***ps*GSL_PEG_(NO_2_)‐N_3_** in the presence of nitroreductases by LC‐MS analysis after incubation of the compounds with the commercially available nitroreductase (NTR) NfsB from *Escherichia coli* and required cofactors NADH and FMN in HEPES buffer (20 mM, pH 7.4) at 37°C.

Coming from the enzymatic investigation of GSLs with the thioglucosidase myrosinase, which shows a relative slow conversion of their substrates [38], we first incubated ***ps*GSL_PEG_(NO_2_)‐N_3_** for 1 h at 37°C.

**FIGURE 1:**
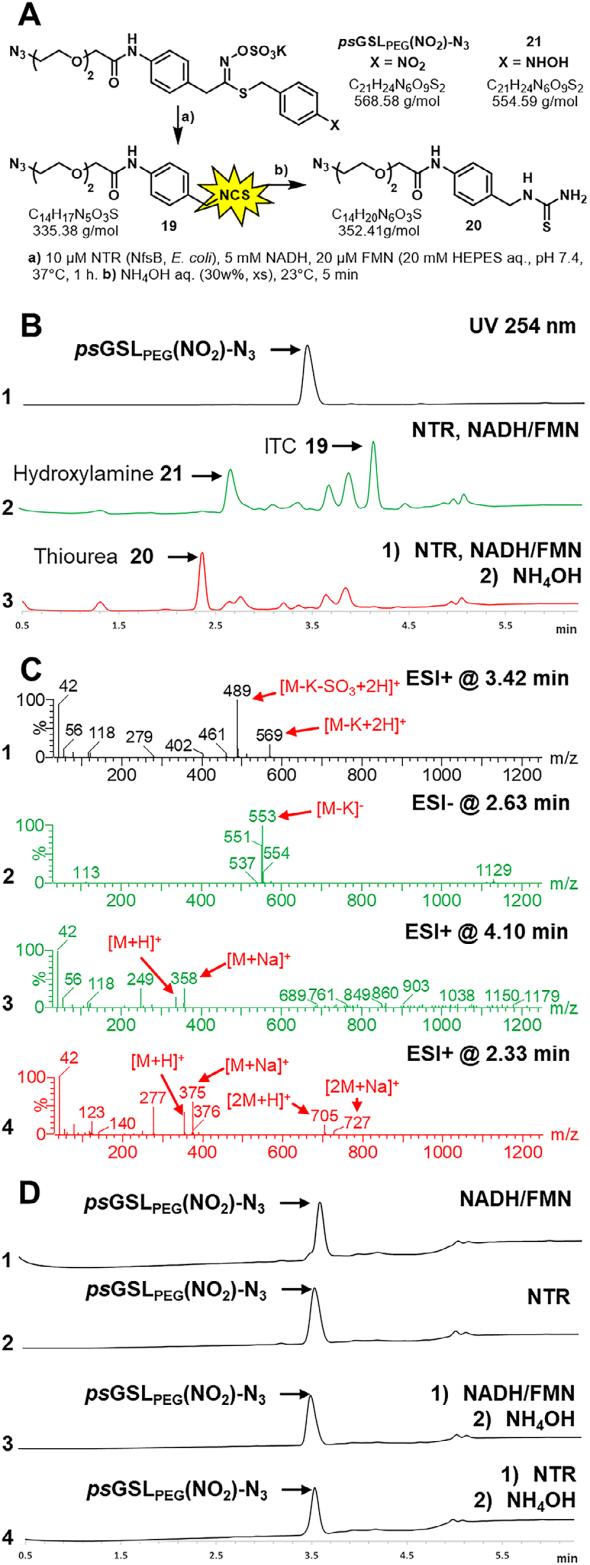
LC‐MS analysis after incubation of ***ps*GSL_PEG_(NO_2_)‐N_3_** with nitroreductase (NTR) NfsB from *E. coli* and subsequent derivatization with NH_4_OH solution. (A): Structures of ***ps*GSL_PEG_(NO_2_)‐N_3_**, ITC **19**, thiourea **20** and hydroxylamine **21**. (B): UV chromatogram at 254 nm of (B1) pure ***ps*GSL_PEG_(NO_2_)‐N_3_** (500 µM) in HEPES buffer (20 mM, pH 7.4), (B2) incubation of ***ps*GSL_PEG_(NO_2_)‐N_3_** (500 µM) with NfsB (10 µM), NADH (5 mM) and FMN (20 µM) in HEPES buffer (20 mM, pH 7.4) after 1 h at 37°C and (B3) A2 and addition of aqueous NH_4_OH solution (30 w%, 10 µL). (C): ESI+ or ESI‐ mass analysis at (C1) 3.42 min of B1, (C2 and C3) 4.10 min and 2.63 min of B2 and (C4) 2.33 min of B3. D: Control experiments following B2 or B3 in absence of NTR (D1 and D3) or the cofactors NADH and FMN (D2 and D3).

As shown in Figure 1, ***ps*GSL_PEG_(NO_2_)‐N_3_** (see Figure 1A: structure, B1: R*_t_* 3.42 min and C1: mass 569 m/z for [M‐K+2H]^+^) is converted by NfsB and the formation of the corresponding ITC **19** (see Figure 1A: structure, B2: R*_t_* = 4.10 min, and C3: mass 358 m/z for [M+Na]^+^ and 336 m/z for [M+H]^+^) is observed. In addition, we saw the formation of the corresponding hydroxylamine **21** (see Figure 1A: structure, B2: *R*_t_ = 2.63 min, and C2: mass 553 m/z for [M‐K]^−^).Furthermore, adding an excess of concentrated aqueous ammonia, both, ITC **19** and hydroxylamine **21** were rapidly converted into the corresponding thiourea **20** within minutes (see Figure 1A: structure, B3: *R*_t_ = 2.33 min, and C4: mass 375 m/z for [M+Na]^+^, 353 m/z for [M+H]^+^, 705 m/z for [2M+H]^+^, and 727 m/z for [2M+Na]^+^).Next, we performed several control experiments (See Figure 1,D1–4) for the conversion of ***ps*GSL_PEG_(NO_2_)‐N_3_** in the absence of the enzyme or cofactors NADH and FMN, with or without subsequent addition of an excess of 30% aqueous ammonia (10 µL ∼ 157 µmol ∼ 6284‐fold excess compared to ***ps*GSL_PEG_(NO_2_)‐N_3_**). In none of the control experiments we observed any conversion of ***ps*GSL_PEG_(NO_2_)‐N_3_**; and in addition, the compound was chemically stable under these highly basic conditions.

**FIGURE 2:**
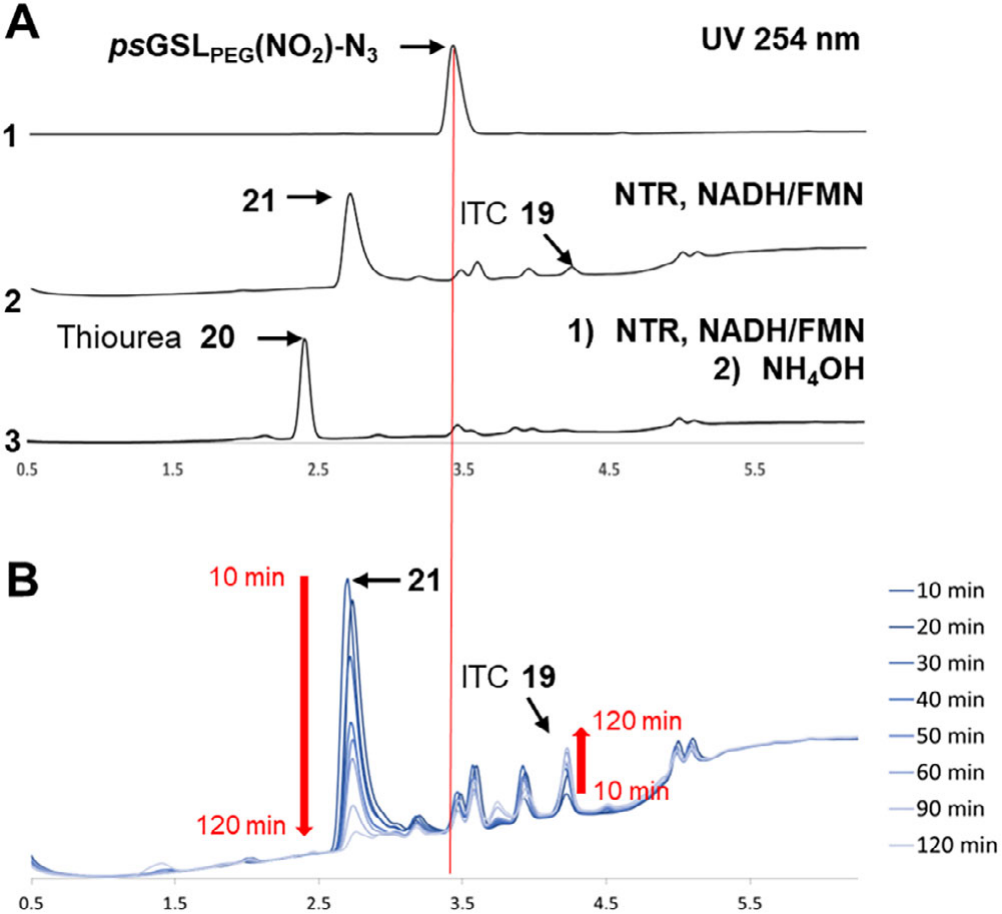
LC‐MS analysis after incubation of ***ps*GSL_PEG_(NO_2_)‐N_3_** with nitroreductase (NTR) NfsB from *E. coli* and subsequent derivatization with NH_4_OH solution. (A): UV chromatogram at 254 nm of (A1) pure ***ps*GSL_PEG_(NO_2_)‐N_3_** (500 µM) in HEPES buffer (20 mM, pH 7.4), (A2) incubation of ***ps*GSL_PEG_(NO_2_)‐N_3_** (500 µM) with NfsB (10 µM), NADH (5 mM) and FMN (20 µM) in HEPES buffer (20 mM, pH 7.4) after 10 min at 37°C and (A3) A2 and addition of aqueous NH_4_OH solution (30 w%, 5 µL) at 23°C. (B): UV chromatogram at 254 nm of the conversion of ***ps*GSL_PEG_(NO_2_)‐N_3_** (500 µM) in presence of NfsB (10 µM), NADH (5 mM) and FMN (20 µM) in HEPES buffer (20 mM, pH 7.4) over the course of 2 h at 37°C (at t = 10, 20, 30, 40, 50, 60, 90, 120 min).

When monitoring the conversion of ***ps*GSL_PEG_(NO_2_)‐N_3_** in the presence of NfsB and FMN/NADH after 10 min full conversion to the hydroxylamine **21** with only little conversion to the ITC **19** was observed in the LC‐MS (See Figure 2, A2).

Interestingly, both compounds were immediately converted into thiourea **20** when the reaction mixture was treated with an excess of aqueous ammonia (See Figure 2, A3) indicating a corresponding 1,6‐elimination from the hydroxylamine **21** and addition of ammonia to the resulting ITC **19**.

When the conversion of ***ps*GSL_PEG_(NO_2_)‐N_3_** in the presence of NfsB and FMN/NADH was monitored over the course of 2 h, **21** was consumed during the reaction under formation of the ITC **19** (Figures 2,B).

**SCHEME 4:**
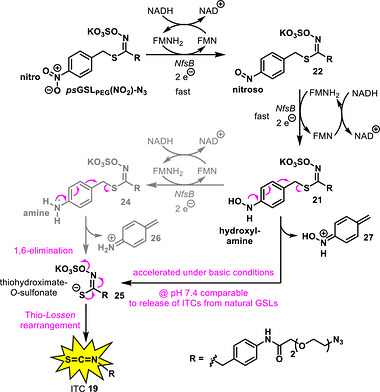
Proposed reduction of ***ps*GSL_PEG_(NO_2_)‐N_3_** catalyzed by the Type I oxygen‐insensitive nitroreductase (NTR) NfsB from *E. coli* via ping‐pong bi‐bi mechanism and conversion to ITC **19**.

Considering that hydroxylamines are intrinsic intermediates in the 6‐electron reduction of a nitro group to its corresponding amine by nitroreductases (Scheme 4), the observation of **21** as rapidly formed reduction product is reasonable. In addition, the nitroreductase NfsB from *E. coli* belongs to the oxygen‐insensitive nitroreductases of Type 1 performing two electron reductions [61, 62] via a strongly substrate dependent ping‐pong‐bi‐bi mechanism [63, 64] Such ping‐pong‐bi‐bi mechanism requires that each 2‐electron reduction product has to leave the active center of the enzyme to allow the binding of a molecule NADH to reduce the prosthetic group FMN [61] again to FMNH_2_. Afterwards, the release of NAD^+^ allows binding of the next substrate in the active center to be further reduced. Strongly depending on the affinity for the substrates, different product distributions are observed for *NsfB*. Indeed, NfsB is known to contribute to chloramphenicol resistance in Gram‐negative bacteria by reduction of chloramphenicol to its corresponding inactive amino‐chloramphenicol [58, 59], but likewise induces susceptibility of Gram‐negative bacteria toward metronidazol by conversion into its hydroxylamine [59] involved in radical processes damaging the bacterial DNA. In our current mechanistic understanding of the overall reaction, the initial reduction of ***ps*GSL_PEG_(NO_2_)‐N_3_** to the corresponding nitroso compound **22** (only detected in traces, Scheme 4) and subsequently to the hydroxylamine **21** as major reduction product is fast. Whether the corresponding amine **24** (See, Scheme 4) is at all formed during the reaction is not clear.

At least we do not detect **24** and it would be a relatively short‐living intermediate anyway. However, the envisaged 1,6‐elimination forming the thiohydroximate‐*O*‐sulfonate **25** and subsequent formation of the ITC **19** via thio‐*Lossen* rearrangement is observed over 2 h, which is comparable to the slow conversion of natural GSLs into ITCs by myrosinase [38], In contrast, upon addition of aqueous ammonia under basic conditions, the 1,6‐elimination and subsequent thio‐*Lossen* rearrangement seem to be significantly accelerated.

**FIGURE 3:**
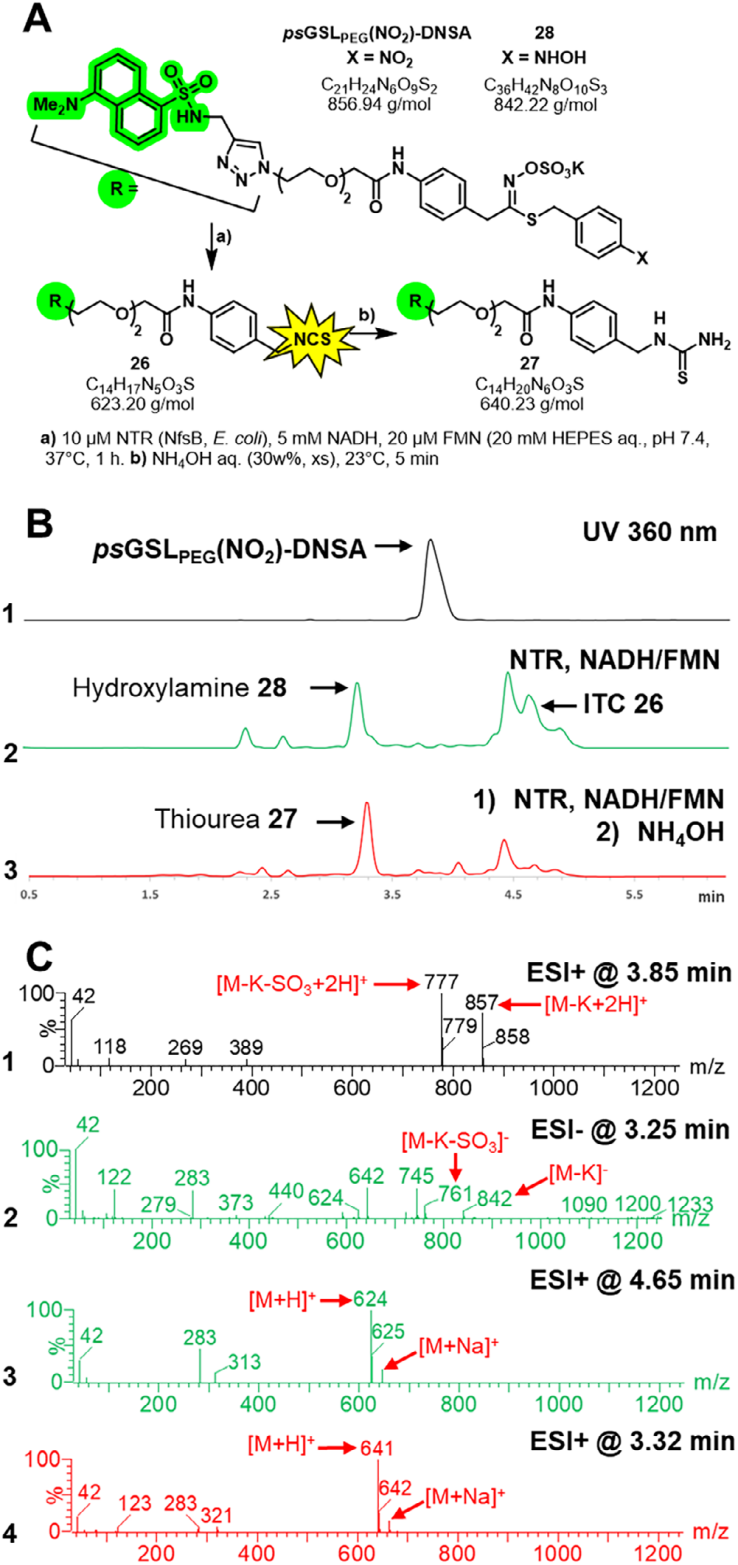
LC‐MS analysis after incubation of ***ps*GSL_PEG_(NO_2_)‐DNSA** with nitroreductase (NTR) NfsB from *E. coli* and subsequent derivatization with NH_4_OH solution. (A): Structures of ***ps*GSL_PEG_(NO_2_)‐DNSA**, ITC **26**, thiourea **27** and hydroxylamine **28**. (B): UV chromatogram at 254 nm of (B1) pure ***ps*GSL_PEG_(NO_2_)‐DNSA** (500 µM) in HEPES buffer (20 mM, pH 7.4), (B2) incubation of ***ps*GSL_PEG_(NO_2_)‐DNSA** (500 µM) with NfsB (10 µM), NADH (5 mM) and FMN (20 µM) in HEPES buffer (20 mM, pH 7.4) after 1 h at 37°C and (B3) A2 and addition of aqueous NH_4_OH solution (30 w%, 10 µL) at 23°C. (C): ESI+ or ESI‐ mass analysis at (C1) 3.85 min of B1, (C2 and C3) 3.25 min, and 4.65 min of B2 and (C4) 3.32 min of B3.

**FIGURE 4:**
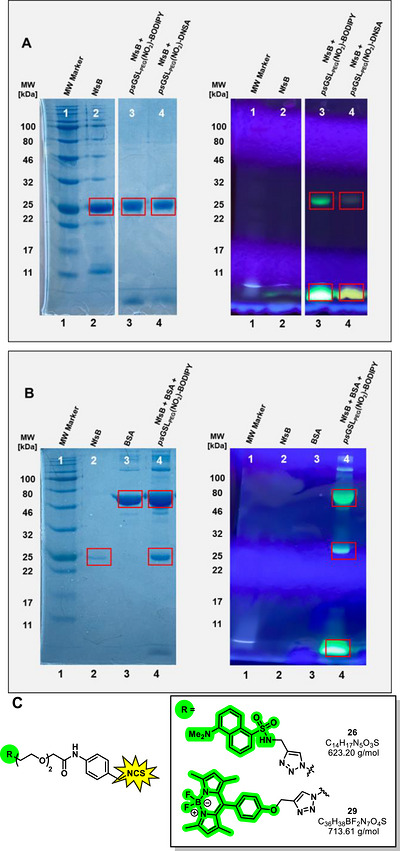
**A**: SDS‐PAGE gel analysis of the conversion of ***ps*GSL_PEG_(NO_2_)‐BODIPY** and ***ps*GSL_PEG_(NO_2_)‐DNSA** in the presence of nitroreductase (NTR) NfsB from *E. coli*, left: visible light/Coomassie blue stain, right: UV light (366 nm), (1) molecular weight marker (3 µL, 2 mg/mL), (2) nitroreductase NfsB (10 µL, 2 mg/mL), (3) 10 µL of ***ps*GSL_PEG_(NO_2_)‐BODIPY** (500 µM) with NfsB (10 µM), NADH (5 mM), and FMN (20 µM) in HEPES buffer (20 mM, pH 7.4) after 2 h at 37°C, (4) 10 µL of ***ps*GSL_PEG_(NO_2_)‐DNSA** (500 µM) with NfsB (10 µM), NADH (5 mM) and FMN (20 µM) in HEPES buffer (20 mM, pH 7.4) after 2 h at 37°C. Lanes have been spliced together from the same gel to remove empty space. B: SDS‐PAGE gel analysis of the labeling of bovine serum albumin (BSA) with ***ps*GSL_PEG_(NO_2_)‐BODIPY** in the presence of nitroreductase (NTR) NfsB from *E. coli*, left: visible light/Coomassie blue stain, right: UV light (366 nm), (1) molecular weight marker (3 µL, 2 mg/mL), (2) nitroreductase NfsB (10 µL, 0.5 mg/mL), (3) BSA (10 µL, 2 mg/mL), (4) 10 µL of ***ps*GSL_PEG_(NO_2_)‐BODIPY** (500 µM) with NfsB (10 µM), NADH (5 mM) and FMN (20 µM) in HEPES buffer (20 mM, pH 7.4) and BSA (2 mg/mL) after 2 h at 37°C. **C**: Structures of ITCs **26** and **29**.

Similarly, also the fluorophore labelled compounds, ***ps*GSL_PEG_(NO_2_)‐DNSA** and ***ps*GSL_PEG_(NO_2_)‐BODIPY**, showed the release of their corresponding ITCs in the presence of nitroreductase (Figures 3 and 4). Again, addition of an excess of concentrated aqueous ammonia, rapidly converted both, ITC **26** and hydroxylamine **28** into the corresponding thiourea **27** within minutes (see Figure 3A: structure, B3: R*_t_* = 3.32 min, and C4: mass 641 m/z for [M+H]^+^ and 663 m/z for [M+Na]^+^).

Next, we wondered if the ITCs formed during the conversion of the *ps*GSLs may label the nitroreductase NfsB through covalent binding. When ***ps*GSL_PEG_(NO_2_)‐DNSA** (see Figure 3A: structure, B1: R*_t_* 3.85 min and C1: mass 857 m/z for [M‐K+2H]^+^ and 777 m/z for [M‐K‐SO_3_+2H]^+^) was incubated with NfsB in the presence of FMN and NADH for 1 h at 37°C, formation of the ITC **26** (see Figure 3A: structure, B2: R*_t_* 4.65 min and C3: mass 646 m/z for [M+Na]^+^ and 624 m/z for [M+H]^+^) alongside with the hydroxylamine **28** (see Figure 3A: structure, B2: R*_t_* 3.25 min and C2: mass 842 m/z for [M‐K]^−^ and 761 m/z for [M‐K‐SO_3_]^−^) was observed.

To prove this, we performed an SDS‐PAGE analysis of the reaction mixtures of ***ps*GSL_PEG_(NO_2_)‐DNSA** and ***ps*GSL_PEG_(NO_2_)‐BODIPY** incubated with NfsB, FMN, and NADH at 37°C for 2 h (see Figure 4A). The gel image revealed fluorescent bands at the height of the enzyme when excited at 366 nm for both reactions, indicating a covalent labeling of NfsB (MW: 24727 Da, Uniprot P38489 with a His_6_‐tag).

Additionally, in the flow through fluorescent small molecules, presumably corresponding to the fluorescent ITCs **26** and **29** (for structures see Figures 4, C) or their hydrolysis products are visible. Furthermore, we obtained evidence for the covalent adduct by intact protein mass spectrometry. We determined a deconvoluted mass of 24726.9 Da for unmodified NfsB, which matched with the expected mass of 24727.8 Da (Uniprot P38489 with a His_6_‐tag). When ***ps*GSL_PEG_(NO_2_)‐N_3_** was incubated with NfsB, FMN, and NADH at 37°C for 2 h and analyzed by LC/HRMS, we were able to observe a chromatographic peak for an NfsB variant with 964.9594 m/z with z = 26, corresponding to a deconvoluted protein molecular mass of 25062.9 Da. The mass shift of +336 Da fits well with the one expected for modification with ITC **19** (+335 Da) within the experimental error of ca. ± 3 Da (compare Figures S3 and S4). Beyond the labeling of the converting enzyme NfsB, we could demonstrate that bovine serum albumin (BSA, 69.3 kDa, Uniprot P02769) added to the reaction mixture was also labelled efficiently with ***ps*GSL_PEG_(NO_2_)‐BODIPY** in the presence of NfsB as show in Figure 4B.

ITCs have been demonstrated to covalently bind peptides via irreversible formation of thioureas upon reacting with lysine residues or via reversible formation of dithiocarbamates upon reacting with cysteine residues [65, 66, 67]. While the pH optimum for the thiourea formation has been shown to be between pH 9–11, dithiocarbamates form predominately at pH 6–8.[66]

Although dithiocarbamates might be the initially formed adducts, they might function as resting intermediate, reforming the ITC under specific conditions and finally leading to lysine thiourea adducts as stable modifications [65].

**SCHEME 5:**
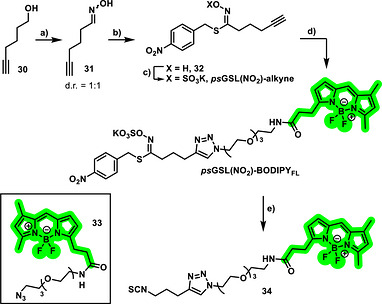
Synthesis of ***ps*GSL(NO_2_)‐alkyne** and ***ps*GSL(NO_2_)‐BODIPY_FL_**. (a) 1) IBX (1.5 equiv), (DMSO), 22°C, 2.5 h, 2) HONH_2_^.^HCl (1.05 equiv), NaOAc (1.05 equiv), (MeCN:H_2_O/3:1), 23°C, 10 h, 67%; (b) 1) NCS (1.1 equiv), (DMF), 0°C, 2 h, 2) 4 (1.24 equiv), DiPEA (6.0 equiv), (Et_2_O), 23°C, 10 h, 89%; (c) 1) SO_3_^.^Py (5.0 equiv), Py (10 equiv), (THF), 60°C, 4 h; 2) KHCO_3_ (2 M aq., 16 equiv) 23°C, 1 h, 69%; (d) Cu(II)SO_4_^.^5 H_2_O (0.2 equiv), TBTA (0.2 equiv), NaAsc (0.4 equiv), (DMF:H_2_O/5:1), 23°C, 15 h, 65%; (e) 10 µM NTR (NfsB, *E. coli*), 5 mM NADH, 20 µM FMN, (20 mM HEPES aq., pH 7.4), 37°C, 1 h.

To further elucidate how ITCs released from *ps*GSLs covalently bind to peptides and proteins, we synthesized the nitroreductase‐responsive probes ***ps*GSL(NO_2_)‐alkyne** and ***ps*GSL(NO_2_)‐BODIPY_FL_** as outlined in Scheme 5.

***ps*GSL(NO_2_)‐alkyne** provides a terminal alkyne moiety compatible with copper‐mediated azide‐alkyne click chemistry within isotopically labelled desthiobiotin‐activity‐based protein profiling (isoDTB‐ABPP) [68, 69] approach (vide infra).

Starting from hex‐5‐yn‐1‐ol (**30**) oxidation with IBX and subsequent conversion with hydroxylamine gave oxime **31** as 1:1‐mixture of diastereoisomers in 67% yield. Formation of the chloro oxime and subsequent coupling with para‐nitrobenzylthiol (**4**) gave the thiohydroximate **32**.

Next, we incubated five model peptides bearing either cysteins or lysines residues or both with ***ps*GSL(NO_2_)‐BODIPY_FL_** in presence of nitroreductase NfsB from *E. coli*, FMN and NADH for 1 h at 37°C (see Table 1) to form peptide adducts.

**TABLE 1:** Labeling of model peptides 1–5 (500 µM) with 500 µM of *ps*GSL(NO_2_)‐BODIPY_FL_ under different conditions. All data is based on technical triplicates.

Entry	Compound	Sequence	Exact mass of the peptide in Da	Labeling conditions	Site of ΔDa +617.27671 = C_28_H_38_BF_2_N_7_O_4_S	Site of ΔDa +585.30463 = C_28_H_38_BF_2_N_7_O_4_
1	**Model peptide 1**	NACGKNAGK	861.41269	A	C3	C3^a^
				B	C3	C3^a^
				C	C3	C3^a^
2	**Model peptide 2**	AcNACGKNAGK	903.42325	A	C3	—
				B	C3	C3^a^
				C	C3	C3^a^
3	**Model peptide 3**	NAGGKNAGK	815.42496	A	—	—
				B	K5/K9	K5^a^
				C	K5	K5^a^
4	**Model peptide 4**	AcNAGGKNAGK	857.43553	A		K5/K9
				B	K9	K9^a^
				C	K5	K5/K9^a^
5	**Model peptide 5**	AcNACGGNAGG	761.27625	A	—	—
				B	C3	—
				C	—	—

**A**: 10 µM NTR (NfsB, E. coli), 5 mM NADH, 20 µM FMN, 20 mM HEPES aq., pH 7.4, 37°C, 1 h. **B**: 1) 10 µM NTR (NfsB, E. coli), 5 mM NADH, 20 µM FMN, 20 mM HEPES aq., pH 7.4, 37°C, 1 h. 2) DiPEA → pH 8.5‐9, 37°C, 2 h. **C**: 10 µM NTR (NfsB, E. coli), 5 mM NADH, 20 µM FMN, 20 mM HEPES aq., pH 7.4, 37°C, 1 h. 2) DiPEA, 37°C, 2 h. 3) TEAB (0.1 M in H_2_O)→ pH 8.5‐9, 37°C, 2 h.

^a^ Asn (N) deaminated peptide mass was found.

Treatment of **32** with pyridine SO_3_ complex and potassium hydrogen carbonate solution gave ***ps*GSL(NO_2_)‐alkyne** in 69% yield. Additionally, ***ps*GSL(NO_2_)‐BODIPY_FL_** could be obtained via CuAAC with BODIPY_FL_ derivative **33** [70] in 65% yield.

Additional samples were adjusted to pH 8.5‐9 by addition of DiPEA or tetraethylammonium bromide (TEAB) solution (0.1 M in H_2_O) and incubated for additional 2 h at 37°C. After precipitation and filtration of NfsB, the samples were subjected to a streamlined mass sample preparation and the modified peptides were analyzed by LC‐MS/MS.

For **model peptide 1** and **model peptide 2**, each containing a cysteine in 3‐position and two lysines in the 5 and 9 positions modification as dithiocarbamate exclusively at C3 reflected by the covalent adduct mass (+617) matching the expected ITC **34** was observed (Entry 1 and 2, Table 1). For both peptides, additionally, a covalent adduct mass (+585) matching the corresponding imidothioate was observed under almost all conditions.

Furthermore, for **model peptide 5** bearing cysteine in the 3‐position but no lysines in the presence of DiPEA the dithiocarbamate formation at C3 was observed (Entry 5, Table 1). For **model peptide 3** and **model peptide 4** with lysines in 5‐ and 9‐position, but lacking any cysteines, modification as thiourea in the presence of DiPEA or TEAB occurred at either K5, K9 or both reflected by the covalent adduct mass (+617) matching the expected ITC **34** (Entry 3 and 4, Table 1). Additionally, for both peptides a covalent adduct mass (+585) matching the corresponding amidine was observed in the presence of DiPEA or TEAB.

For model peptide 4 this amidine adduct could also be found at pH 7.4, while the thiourea adduct was absent under these conditions. Modification of free *N*‐termini for model peptide **1** and **3** was not observed.

Additionally, a shift from S→N modifications (dithiocarbamate to thiourea) under basic was not observed for **model peptide 1** and **2** containing both cysteine and lysine residues (Entry 1 and 2, Table 1).

To further elucidate how ITCs released from *ps*GSLs covalently bind to a given proteome, we next performed a chemoproteomics experiment with ***ps*GSL(NO_2_)‐alkyne** in the proteome of *Staphylococcus aureus* SH1000 utilizing our isotopically labelled desthiobiotin‐activity‐based protein profiling technology (isoDTB‐ABPP) [68, 69], which is based on the isotopic tandem orthogonal proteolysis‐ABPP (isoTOP‐ABPP) [71, 72] platform. Specifically, we utilized an MSFragger [68, 69], based workflow that we recently established to monitor electrophile reactivity and selectivity in a completely unbiased fashion [69].

**FIGURE 5:**
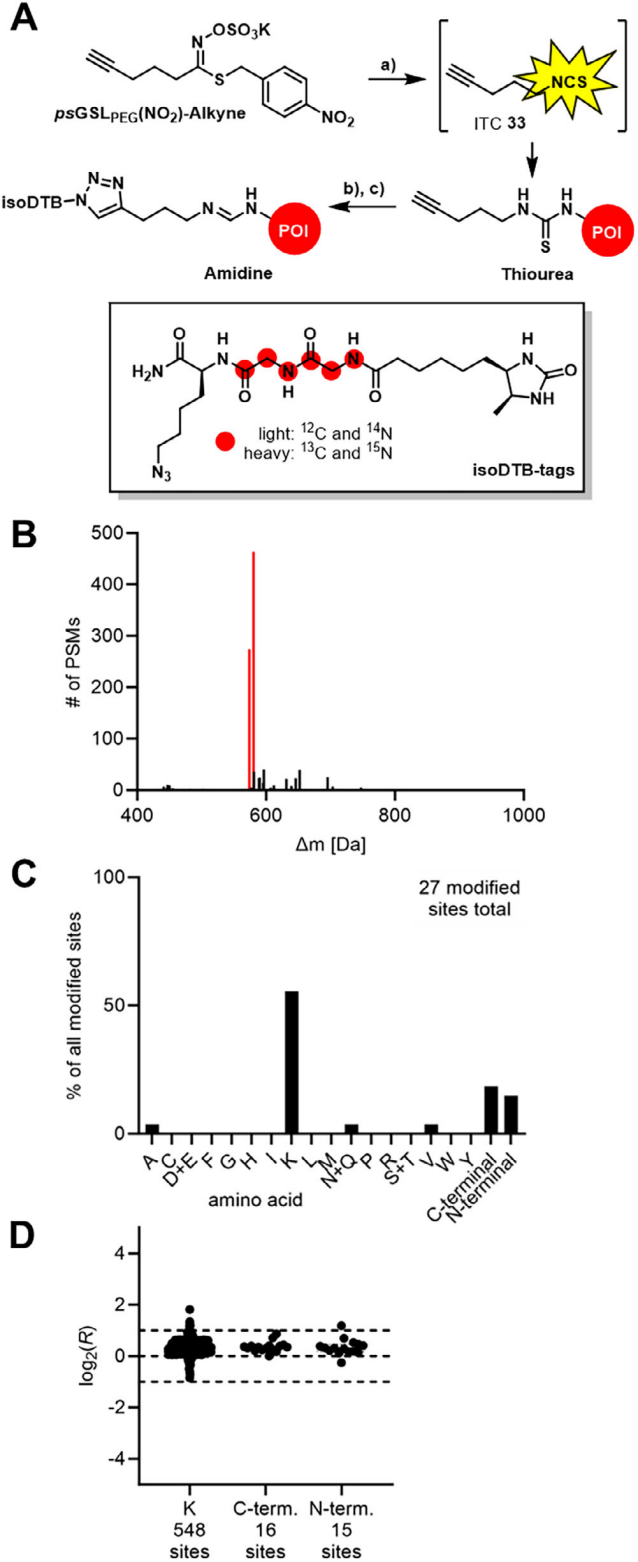
Labeling of *S. aureus* SH1000 proteome (at 1 mg/mL) with 100 µM of ***ps*GSL(NO_2_)‐alkyne** using the isoDTB‐ABPP workflow [68]: (A): Structures of ***ps*GSL(NO_2_)‐alkyne**, ITC **33** and the heavy and light **isoDTB‐tags**; general structures of thioureas and isoDTB‐tag‐clicked amidines (POI = protein of interest). (a) 10 µM NfsB (*E. coli*), 5 mM NADH, 20 µM FMN (20 mM HEPES aq., pH 7.4), 1 mg/mL lysate S. aureus SH1000, 37°C, 1 h; (b) TBTA, CuSO_4_ 5 H_2_O, TCEP, isoDTB‐tag (light or heavy), (c) Pull‐down with streptavidin‐agarose beads and MS work flow of enriched proteins, see SI. B: Masses of modification determined through analysis with an Open Search in MSFragger [75, 76]. (C): Amino acid selectivity determined through an Offset Search that localizes the modification to the modified amino acid(s). In this way, selectivity is assessed across all proteinogenic amino acids. The bar represents the fraction of all modified sites that is modified at the indicated amino acid. (D): Quantification of the modification at the main amino acid residues identified in the offset search at the selected masses was performed using a Closed Search and the IonQuant [77] feature. The heavy and light samples were mixed at a ratio of 1:1. The dashed lines indicate the expected values of log2(*R*) = 0 and the preferred window of quantification (−1 < log2(*R*) < 1). All data is based on technical duplicates. For a representative MS/MS spectrum with the relative b and y ions, see Figure S5.

Therefore, two identical samples of freshly prepared lysate of *S. aureus* SH1000 were incubated with ***ps*GSL(NO_2_)‐alkyne** in presence of nitroreductase NfsB from *E. coli*, FMN and NADH for 1 h at 37°C (see Figure 5A) to form protein adducts.

In one sample the heavy isoDTB‐tag was attached to the terminal alkyne via CuAAC and in the other one the corresponding light isoDTB‐tag. Afterwards, the samples were combined and a pull‐down of isoDTB‐labelled proteins utilizing streptavidin‐agarose beads was performed. The enriched protein fractions were digested, subjected to a streamlined mass sample preparation and the modified peptides were analyzed by LC‐MS/MS.

Surprisingly, the almost exclusively observed proteome modification was found to be an amidine instead of the expected thiourea or dithiocarbamate obtained in the other experiments performed (Figure 5B).

Currently, we believe that a substrate‐dependent, acid‐catalyzed desulfurization is occurring in the presence of TFA during the sample preparation of the isoDTB‐ABPP workflow or during ionization in the LCMS. Oxidative formation of amidines from thioureas is known in the presence of oxidants such as hydrogen peroxide under acidic conditions already at 25°C [73] or in the presence of oxidative enzymes [74]. Similarly, we had seen amidine formation at basic conditions in our peptide modification experiments (Table 1).

However, attempts to facilitate amidine formation under strong acidic conditions led to formation of only traces of corresponding amidines in presence of formic acid or TFA at 23°C. (See Figure S6).

We ruled out a direct reaction of ***ps*GSL(NO_2_)‐alkyne** with lysine residues, as no reaction of ***ps*GSL(NO_2_)‐alkyne** with BSA or lysine is observed in the absence of NfsB and as shown in Figure 1D *ps*GSLs are stable in the presence of high concentration of nitrogen nucleophiles even at pH 14. The exact mechanism and nature of this desulfurization is still under investigation.

However, according to the isoDTB‐ABPP workflow, this is the main modification that is detected by chemoproteomic analysis (for a representative MS/MS spectrum with the relative b and y ions, see Figure S5). We next used the mass of modification of amidine formation as input for an offset search to study the amino acid selectivity (Figure 5C) [69].

The modification was mainly localized to amino groups in the detected proteins (lysines and protein *N*‐termini) with some surprising reactivity also at protein *C*‐termini.

Overall, the modification does not seem to be very stable under the detection conditions as seen by the low number of localized sites after the application of the very stringent filters of the offset search (27 sites unambiguously localized). This indicates that the modification is easily lost upon fragmentation in the mass spectrometer. Modification of cysteines with ITCs, although potentially occurring initially, could not be detected under the conditions of the isoDTB‐ABPP workflow. Moreover, as no S→N shifts were observed during incubation with the **modelpeptides 1** and **modelpeptide 2** (Entries 1 and 2, Table 1), it appears that any dithiocarbamate modifications are cleaved under the isoDTB‐ABPP conditions, leaving only amidine modifications detectable. Overall, amidine modifications in the proteome were found to be mainly localized to lysine residues and protein termini.

In the final stage of the isoDTB‐ABPP analysis, we quantified the residues that are modified with the amidine modification. It is striking that under the less stringent localization filters used for quantification, we mainly identify lysine modification indicating that stability of the modification at lysines is the main limitation to high quality localization and that the probe mainly leads to lysine‐modified peptides. Of the 548 modified lysine sites, the vast majority were quantified with a small spread around the expected log_2_(*R*) of 0 indicating reliable quantification of these modified peptides. Among the 548 modified lysines were 265 that were identified in 89 different essential proteins. These include lysines in important functional sites of the proteins such as those forming Schiff bases during catalysis (K82 of pyridoxal 5'‐phosphate synthase pdxS and K151 of deoxyribose‐phosphate aldolase deoC), other residues at active sites (K596 of glutamine‐fructose‐6‐phosphate aminotransferase glmS and K217 of ribitol‐5‐phosphate cytidylyltransferase 1 tarI), residues at nucleotide binding sites (K71 at the ATP binding site of formate‐tetrahydrofolate ligase fhs, K23 at the GTP binding site of elongation factor G fusA and K46 of the ATP binding site of succinate‐CoA ligase sucC) and, strikingly, at DNA binding sites of transcriptional regulators (K52 and K56 of HTH‐type transcriptional regulator SarR and K57 and K74 of HTH‐type transcriptional regulator MgrA). While the performed isoDTB‐ABPP experiment does not inform on the stoichiometry of engagement at these residues, it is a good indication that ITCs can exert an effect in bacteria through engagement of a variety of functional lysines in proteins.

Finally, we wondered if the conversion of *ps*GSLs in the presence of nitroreductase could also be achieved in living organisms, thus allowing for in vivo labeling of amines and lysine residues. Therefore, we aimed to release the BODIPY‐labelled ITC **29** (compare Figure 4C) in the presence of NfsB in living *Caenorhabditis elegans* as an easy‐to‐handle model organism expecting to label free amines through covalent binding present in intestinal membranes.

**FIGURE 6:**
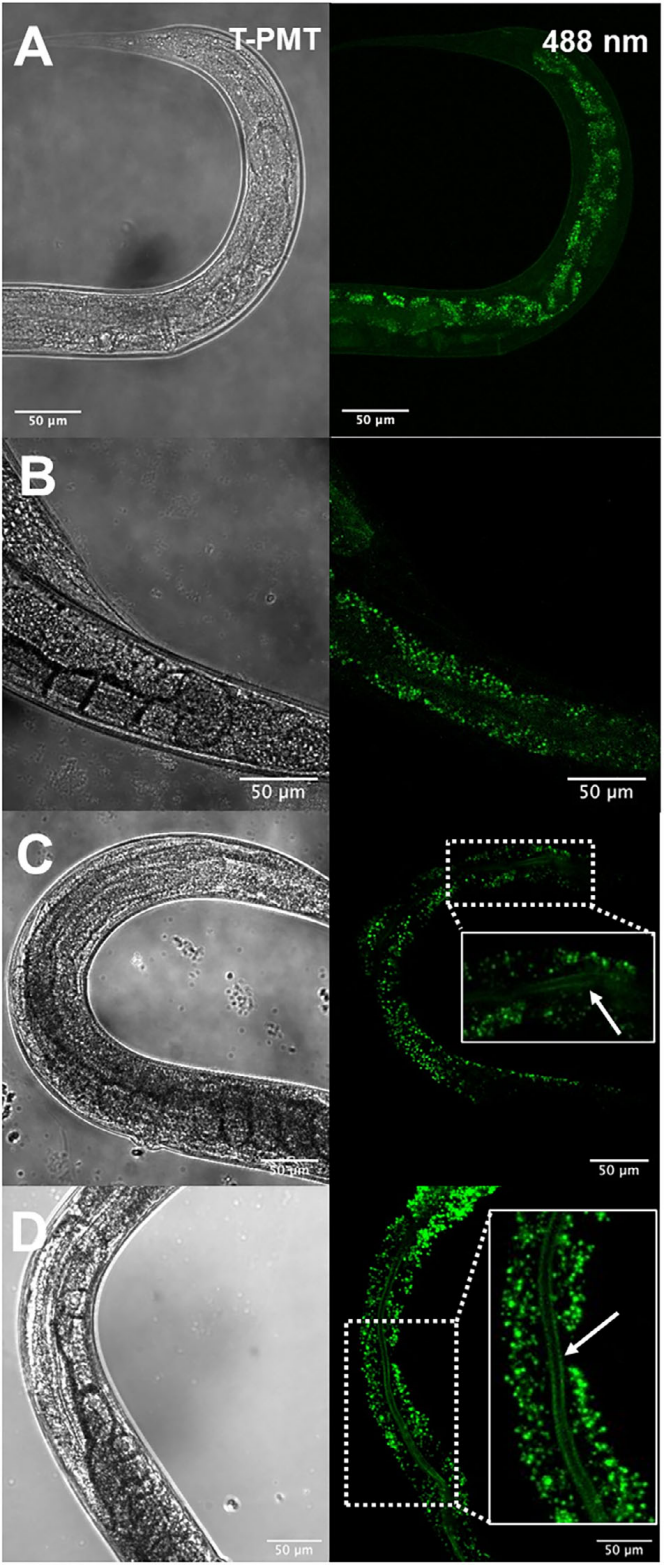
Fluorescence microscopy imaging of *C. elegans* 24 h after an initial pre‐incubation step with ***ps*GSL_PEG_(NO_2_)‐BODIPY** for 1 h. (A): untreated worms. (B): pre‐incubation with ***ps*GSL_PEG_(NO_2_)‐BODIPY** at 23°C (but no NfsB or cofactors). (C): 1 h preincubation with ***ps*GSL_PEG_(NO_2_)‐BODIPY** at 23°C and 2 h incubation with cofactors NADH and FMN at 23°C (but no NfsB). (D): 1 h preincubation with ***ps*GSL_PEG_(NO_2_)‐BODIPY** at 23°C and 2 h incubation with NfsB and cofactors NADH and FMN at 23°C. All conditions included washing out of excessive unconsumed and unbound ***ps*GSL_PEG_(NO_2_)‐BODIPY** for 2 h with M9 buffer as well as 24 h incubation on NGM plates in presence of OP50 *E. coli*. White arrows indicate the labelled intestinal membrane of *C. elegans*.

To investigate this, day 1 adult *C. elegans* worms were pre‐incubated with ***ps*GSL_PEG_(NO_2_)‐BODIPY** for 1 h at 23°C, before NfsB, FMN and NADH were added for a 2 h incubation at 23°C, followed by an immediate washing step with M9 buffer for 2 h (see Figure 6D). As controls, the worms were incubated with ***ps*GSL_PEG_(NO_2_)‐BODIPY** alone (see Figure 6B) and ***ps*GSL_PEG_(NO_2_)‐BODIPY** and the cofactors FMN and NADH but without external nitro‐reductase (NfsB) from *E. coli* (see Figure 6C). After the excess of ***ps*GSL_PEG_(NO_2_)‐BODIPY**, NfsB, FMN and NADH were washed out, confocal fluorescence microscopy imaging of the worms was carried out at 488 nm 24 h after initial incubation with ***ps*GSL_PEG_(NO_2_)‐BODIPY**.

As additional control, we recorded pictures of untreated worms showing the naturally occurring autofluorescence from *E. coli* filled gut granules in the intestines of the worms (Figure 6A, see green dots), which are fed with *E. coli*. To our delight, preincubation with ***ps*GSL_PEG_(NO_2_)‐BODIPY** followed by subsequent incubation with NfsB and cofactors led to strong fluorescent labeling of the membrane of the intestinal lumen (see Figure 6D). In contrast, when incubation was performed with only ***ps*GSL_PEG_(NO_2_)‐BODIPY**, without NfsB and the cofactors NADH and FMN, no specific signal after 24 h was detected, except for a diffuse signal in the intestinal lumen arising from potentially unbound compound (see Figure 6B). When we performed the incubation with the cofactors only, not adding external NfsB, a weak and diffuse signal was observed in only a few of the worms in the very posterior part of the intestinal lumen (see Figure 6C). This finding is reasonable as the worms feed on *E. coli*, thus, endogenous NTR from intestinal *E. coli* lysate might lead to a low degree conversion of the probe.

Therefore, the specificity in NfsB mediated ITC formation leading to covalent labeling is clearly shown in these experiments and serves as a first in vivo proof‐of‐concept, which can be widely expanded into further applications. Furthermore, it is worth mentioning that all worms survived the treatment with ***ps*GSL_PEG_(NO_2_)‐BODIPY** and were not particularly affected by the ITC **29**. Additionally, the worms displayed a normal broad size with no obvious developmental effects in the new generation.

## 3 Conclusion

Here, we have introduced the concept of *pseudo*glucosinolates (*ps*GSLs) that can be activated into isothiocyanates (ITCs) with noncanonical enzymes. We have demonstrated the release of ITCs from ***ps*GSL_PEG_(NO_2_)‐N_3_**, ***ps*GSL_PEG_(NO_2_)‐BODIPY**, ***ps*GSL_PEG_(NO_2_)‐DNSA**, and ***ps*GSL(NO_2_)‐alkyne** in the presence of nitroreductase NfsB from *E. coli*. The released ITCs were demonstrated to be effective in labeling of BSA and show a predominantly lysine‐selective modification of proteins in the entire proteome including many functional sites of essential proteins. Additionally, we performed NfsB‐mediated ITC formation and subsequent covalent BODIPY‐labeling of the membrane in the intestinal lumen of *C. elegans*, demonstrating conversion, functionality, and tolerability of the probes in vivo.

In contrast to natural GSLs, *ps*GSLs represent a complementary prodrug approach for bio‐responsive release of ITCs, which holds potential to be adjusted in their bio‐responsiveness toward multiple enzymes and chemical microenvironments. From a translational perspective, challenges remain, including achieving selective activation in complex mammalian systems, controlling ITC reactivity to minimize off‐target protein modification, and optimizing pharmacokinetic stability and tissue distribution. At the same time, the modular design of *ps*GSLs offers opportunities to tailor activation to disease‐associated enzymatic activities, such as bacterial nitroreductases or tumor‐specific redox environments, thereby enabling localized ITC delivery.

The concept of *ps*GSLs might therefore find application not only in chemical biology for enzyme‐dependent labeling of biomolecules, but also in the development of targeted prodrug strategies to exploit the broad variety of biological activities of potentially hydrolysis‐sensitive ITCs [20].

Currently, the protection of the para‐aminobenzylthiol unit with different peptidase‐, oxidoreductase‐ [52], and hydrolase‐responsive masking groups is under investigation to expand the concept of psGSLs toward a platform technology for bio‐responsive protein labeling and ITC‐based covalent inhibitors of proteins, with future studies addressing selectivity, safety, and potential therapeutic window in more advanced biological models.

## Author Contributions

Conceptualization: PK; Methodology: CCJ, CSGG, MDK, AC, LW, MZ, LCC, JML, and UB; Software: –; Validation: CSGG, MZ and CCJ; Formal analysis: MZ; Investigation: CCJ, CSGG, MDK, AC, LW, MZ, LCC, JM, KB, and UB; Resources: PK, MB, SAS, SMH, and JM; Data curation: PK; Writing –original draft: CSGG, CCJ, AC, MDK, MZ, SMH, and PK; Writing –review & editing: all authors; Visualization: PK, AC, CSGG, MDK, MZ, JM, and SMH; Supervision: PK, MB, SAS, JM, and SMH; Project administration: PK; Funding acquisition: PK, MB, SAS, JM, and SMH. Contribution are given with CRediT definition according to Brand et al.,[91].

## Conflicts of Interest

The authors declare no conflicts of interest.

## Supporting information




**Supplementary File 1**: The authors have cited additional references within the Supporting Information [68, 69, 70, 75, 76, 77, 78, 79, 80, 81, 82, 83, 84, 85, 86, 87, 88, 89, 90, 92].

**Supplementary File 2**: chem71012‐sup‐0002‐SuppMat.zip.

## Data Availability

Nuclear magnetic resonance raw data of all synthesized compounds have been deposited to the NMRXiv (https://nmrxiv.org) with the DOI:10.57992/nmrrxiv.p60 and DOI:10.57992/nmrxiv.p121. Mass spectrometric data for all proteomic analyses have been deposited to the ProteomeXchange Consortium (http://proteomecentral.proteomexchange.org) via the PRIDE partner repository (https://www.ebi.ac.uk/pride/archive/) [92] with the dataset identifier PXD049355 (total Proteomics) and PXD065044 (synthetic peptides). An Excel files named “description of proteomics data (total proteomics).xlsx” and “description of proteomics data (synthetic peptides)”.xlsx containing the descriptions of the proteomics data is available at the journals website as Supporting Information.
