# Supplementary material for: Bioresponsive pseudoGlucosinolates (psGSLs) Release Isothiocyanates (ITCs) in the Presence of Nitroreductases
Source: Chemistry. 2026 Apr 17;32(25):e71012. doi: 10.1002/chem.71012 (PMC13331584; doi:10.1002/chem.71012)

Supporting Information

Bioresponsive *pseudo*-glucosinolates (*ps*GSLs) release isothiocyanates (ITCs) in the presence of nitroreductases

Claire C. Jimidar,[b],# Charity S. G. Ganskow,[a],# Mervic D. Kagho,[a],# Aishi Chakrabarti,[a],# Lorenz Wiese,[b] Michael Zollo,[c] Ulrike Beutling,[d] Leona C. Cesar,[a] Julia Morud,[a] Kamila Bugaj,[a] Mark Brönstrup,[d] Stephan A. Sieber,[c] Stephan M. Hacker,[e] and Philipp Klahn*[a],[b]

Abstract: Glucosinolates (GSLs) are plant secondary metabolites that release bioactive isothiocyanates (ITCs) upon myrosinase-mediated activation. While ITCs display diverse antimicrobial and chemoprotective activities, their application is limited by dependence on myrosinase and intrinsic hydrolytic instability. Here, we introduce *pseudo*glucosinolates (*ps*GSLs), a synthetic platform that mimics the natural GSL activation mechanism but replaces the thioglucosidic trigger with an enzyme-responsive para-aminobenzylthiol motif. Using nitroreductase (NfsB) as a non-canonical activating enzyme, we synthesized and characterized a series of nitro-masked *ps*GSLs, including azide-, alkyne-, and fluorophore-functionalized derivatives. Enzymatic reduction induces a self-immolative 1,6-elimination and subsequent thio-Lossen rearrangement, releasing ITCs under physiological conditions. The liberated ITCs covalently modify peptides and proteins, showing predominant lysine reactivity in chemoproteomic analyses of the Staphylococcus aureus proteome, including functional sites of essential proteins. Fluorescent probes enabled visualization of enzyme-dependent protein labeling and demonstrated nitroreductase-triggered ITC release *in vivo* in *Caenorhabditis elegans*. Together, *ps*GSLs establish a modular, bioresponsive prodrug and chemical biology platform for enzyme-controlled ITC delivery, expanding the scope of ITC-based covalent modification beyond natural myrosinase-dependent systems.

[a] Department of Chemistry and Molecular Biology,
University of Gothenburg
Natrium, Medicinaregatan 7B, 413 90 Gothenburg, Sweden
E-mail: philipp.klahn@gu.se

[b] Institute of Organic Chemistry
Technische Universität Braunschweig
Hagenring 30, 38106 Braunschweig, Germany

[c] Center for Functional Protein Assemblies
Technische Universität München
Ernst-Otto-Fischer-Straße 8, 85748 Garching, Germany,

[d] Department for Chemical Biology
Helmholtz Center for Infection Research
Inhoffenstraße 7, 38124 Braunschweig, Germany.

[e] Department of Molecular Physiology, Leiden Institute of Chemistry
Leiden University
Einsteinweg 55, 2333 CC Leiden, The Netherlands

Table of Contents

Experimental Procedures 3

General Methods 3

Synthesis of compounds 5

Synthesis of **model peptide 1-5** 17

LC-MS analysis of the purity of ***ps*GSL$_{PEG}$(NO$_2$)-N$_3$**, ***ps*GSL$_{PEG}$(NO$_2$)-BODIPY**, ***ps*GSL$_{PEG}$(NO$_2$)-DNSA** and ***ps*GSL$_{PEG}$(NO$_2$)-BODIPY$_{FL}$** 20

LC-MS analysis of the stability of ***ps*GSL$_{PEG}$(NO$_2$)-N$_3$** 21

Biochemical and biological evaluation of the compounds 22

- LC-MS analysis of the nitroreductase mediated release of ITCs from *ps*GSLs 22
- SDS-PAGE of enzymatic conversion and analysis of protein labeling 23
- HRMS analysis of NfsB labelled with ***ps*GSL$_{PEG}$(NO$_2$)-N$_3$** 24
- Modification of synthetic model peptides with ***ps*GSL(NO$_2$)-BODIPY$_{FL}$** 26
- Modification of the proteome of *S. aureus* SH1000 with ***ps*GSL(NO$_2$)-alkyne** 27
- LC-MS analysis of amidine formation from the nitro-reductase mediated release of ITCs from *ps*GSLs 31
- Fluorescence microscopy imaging in *C. elegans* showing enzymatic conversion of *ps*GSLs into corresponding ITCs and covalent binding to intestinal lumen 32

References 33

Author Contributions 34

Appendix: ^{1}H, ^{13}C, ^{19}F and ^{11}B NMR Spectra of the Compounds 35

Experimental Procedures

General Methods

Unless otherwise noted, all reagents were purchased from commercial suppliers and used without further purification. *N,N'*-Dimethylformamide (DMF): *Acros Organics*, puriss., extra dry, over molesieve (water ≤ 0.005%), Pyridine (Py): *Acros Organics*, puriss., extra dry, over molesieve (water ≤ 0.005%), Dimethylsulfoxid (DMSO): *Acros Organics*, puriss., extra dry, over molesieve (water ≤ 0.005%), Methanol (MeOH): *Acros Organics*, puriss., extra dry (water ≤ 0.005%)) 2-Iodoxybenzoic acid (IBX) was synthesized according to the procedure of *Frigerio et al.*.[1]
Moisture sensitive reactions were performed under argon atmosphere in dried glassware. Dry dichloromethane, diethyl ether, toluene and tetrahydrofuran for moisture sensitive reactions have been taken from a MB-SPS-800 (MBraun) solvent purifications system and stored under argon. All solvents used for workup and purification were of HPLC grade. Reactions were monitored by TLC, LCMS or NMR. Solution of compounds in organic solvents were concentrated using rotary evaporators at a water bath temperature of max. 35°C. Solvent residues were removed in high vacuum at pressure of appr. 10^{-2} mbar.

Flash chromatography was done using appropriate glass columns filled with silica gel (Merck Millipore, Geduran® Si60, 1.11567.9025, 40-63 µm) or was performed using the automated chromatography system Biotage® Select. Cartridges packed with silica gel (Merck Millipore, Geduran® Si60, 1.11567.9025, 40-63 µm) using a Büchi Cartridger® C-670 were used for this system.

Preparative reversed phase high pressure liquid chromatography (prep. HPLC RP) was performed on a Hypersil GOLD C18 RP-column (Part No. 25005-259270), 5 µm, 250 mm×21.2 mm (10 mL/min) using a Thermo Fisher Scientific Dionex Ultimate 3000 HPLC system. Eluents, gradients and additives are given in parentheses. As eluents HPLC grade acetonitrile and water (VWR Chemicals, HPLC grade) containing 0.1% of TFA (Carl Roth, 6957.1, 99.9%) were used. Product containing fractions were combined, diluted with dist. H_2O (min. 1:1/solvent:H_2O), frozen and lyophilized using the freeze dryer Zirbus Technology VaCo2, -80°C.

Thin-layer chromatography (TLC) was performed on pre-coated glass plates (Merck TLC Silicagel 60 F254, 1.15341.0001, 2.5x7.5 cm) and components were visualized by observation under UV light (λ = 254 nm [UV254] or λ = 366 nm [UV366]), visible light or treatment of developed plates with TLC staining solutions (for preparation see list below) followed by heating. Eluent or eluent-mixtures used are reported in parentheses.
$KMnO_4$ staining solution [KMnO4]: 1.5 g $KMnO_4$, 10 g K_2CO_3, and 1.25 mL 10% NaOH in 200 mL H_2O; CAM staining solution [CAM]: 1 g $Ce(IV)(SO_4)_2$, 2.5 g $(NH_4)_6Mo_4O_7$ in 100 mL 10% H_2SO_4; Ninhydrin staining solution [ninhydrin]: 1.5 g Ninhydrin in 100 mL abs. EtOH and 3.0 mL HOAc; Triphenyl phosphine reducing solution [PPh_3]: 5.0 g PPh_3 in 50 mL CH_2Cl_2.

NMR spectra were recorded on a Bruker AV-300, AVIII400, AVIIIHD500, and Bruker Avance NEO600 spectrometer with cryoprobe system or Varian Inova 400 spectrometer at 293.15 K. ^1H NMR spectra were recorded at 300 MHz, 400 MHz, 500 MHz, and 600 MHz. ^{13}C NMR spectra were recorded at 76 MHz, 100 MHz, 126 MHz or 150 MHz. Chemical shifts (δ) are reported in parts per million [ppm] relative to solvent signal. Multiplicity is indicated as follows: s (singlet); bs (broad singlet); d (doublet); t (triplet); q (quartet); m (multiplet); dd (doublet of doublets), etc.. For the processing of the raw data the software MestReNova (Version 14.2.0-26256) from MestreLab Research S.L. was utilized.

IR spectra were recorded on a Bruker Tensor 27 IR spectrometer with ATR-technique. Only wave numbers of observed absorption peaks are given.

Low resolution mass spectrometry (LRMS) data were recorded using:

a) a Waters™ LCMS system consisting of an Acquity Arc HPLC, an ACQ Arc Column Heater/Cooler (0-60°C), 2489 UV/Vis Detector (200-600 nm) and an Acquity QDa Mass Detector (ESI +/-, Quadropole, max.1250 Da, cone voltage: 20.0 V, probe temperature 600°C) equipped with a MS/UV Splitter (10:1) and a column switch with three analytical RP columns (1. Waters™ C18 RP Column XBridge BEH C18 130Å, 2.5 µm, 2.1 x 50 mm with Guard Column XBridge BEH C18 V-Gd Cart 2.5 µ, 2.1 x 5 mm, 2. Waters™ C8 RP Column XBridge PRM BEH 130Å C8 2.5 µm 2.1 x 50 mm with Guard Column XBridge BEH C18 V-Gd Cart 2.5 µm, 2.1 x 5 mm, 3. Waters™ C18 RP Column XBridge BEH C18 130Å, 3.5 µm, 2.1 x 150 mm with Guard Column XBridge BEH C18 V-Gd Cart 2.5 µ, 2.1 x 5 mm); Data were analyzed with the Software MassLynx 4.1.

b) LCMS system consisting of an Agilent 1100 HPLC system equipped with DAD detector and an Applied Biosystems API 150 EX quadrupole mass detector with electron spray ionization (ESI).

High resolution mass spectrometry (HRMS) data were recorded using:

a) a linear iontrap coupled with orbitrap mass analyser LTQ-Orbitrap Velos from Thermo Fisher Scientific (Resolution: 100000 FWHM (at m/z = 400 amu), Scan: 130-2000 amu (resulting in acquisition times of 1.6 sec per cycle)). Electrospraymeasurements were performed in direct infusion mode using a custom made microspray-device mounted on a Proxeon nanospray ion source. The microspray-device allows for the sample infusion through a stainless steel capillary (90 µm I. D.). Accurate mass measurements in the orbitrap were performed using the lock mass option of the instrument control software using the cation of tetradecyltrimethylammonium bromide (256.29988 amu) as internal mass reference. (Sample concentration: approx. 50 µg/mL. Solvent: MeOH spiked with 0.1 mg/mL tetradecyltrimethylammonium bromide (unless otherwise stated). Flow: approx. 1 µL/min. Typical spray voltage pos. mode: 2.3 - 2.8 kV. Typical spray voltage neg. mode: 1.7 - 2.5 kV).

b) an Agilent LC-QTOF 6520 with Agilent Infinity 1260 HPLC and Agilent 6520 quadrupole time-of-flight mass analyzer equipped with an ESI or APCI ionization source.

UV-Vis spectroscopy data:

a) of media and protein solution were recorded using a NanoPhotometer NP80 (Implen). Optical density was measured at a wavelength of 600 nm and protein concentration was measured at a wavelength of 280 nm.

b) of compounds were recorded using a Cary 100 Bio UV/Vis spectrometer (Varian).

Fluorescence emission spectroscopy of compounds was performed using a Cary Eclipse fluorescence emission spectrometer (Varian).

Incubation of media was performed in an INCU-Line Incubator (VWR) and a MaxQ 8000 shaker (Thermo Fisher Scientific).

SDS-PAGE was performed in a Mini PROTEAN SDS-PAGE system (Biorad) with a MS 300V power supply (Major Science) and analyzed with a MicroDOC gel imaging system.
For the SDS PAGE Gel Electrophoresis, handcasted 12% Polyacrylamide gels were used for the BSA labeling. Gels were handcasted according to BioRad protocol: 12% stacking gel solution was prepared using 30% acrylamide/bis (6.0 mL), 1.5 M TRIS HCl, pH 8.8 (3.75 mL), 10% SDS (150 µL), diH_2O (5.03 mL), TEMED (7.5 µL), 10% APS (75 µL) and poured into glass cassette sandwich using a glass pipette until the solution reaches the mark 1 cm below the teeth of the comb. Gel was allowed to polymerize within 45-60 min. Comb was placed in the cassette and 4% stacking gel solution consisting of 30% acrylamide/bis (1.98 mL), 0.5 M TRIS HCl, pH 6.8 (3.78 mL), 10% SDS (150 µL), diH_2O (9 µL), TEMED (15 µL), 10% APS (75 µL) was poured down the spacer nearest the upturned side of the comb until all teeth of the comb were covered by the solution. Gel was allowed to polymerize within 30-45 min. Handcasted gels were used with running buffer containing 25 mM TRIS·HCl, 192 mM glycine, 0.1% SDS in dH_2O. All samples were loaded onto the handcast gels in the presence of 4X LDS sample loading buffer (sample:loading buffer/3:1). Precision plus protein dual Xtra standardsTM was used as a protein ladder.
Additionally, 8-16% Mini-PROTEAN® TGX™ Precast Protein Gels, 10-well, 50 µL (Biorad, #4561104) were used with running buffer containing 25 mM TRIS·HCl, 192 mM glycine, 0.1% SDS in diH_2O. All samples were loaded onto the precast gels in the presence of 4X LDS sample loading buffer (sample:loading buffer/3:1).

Synthesis of compounds

1-(Bromomethyl)-4-nitrobenzene (2)

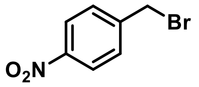

Chemical Formula: $C_7H_6BrNO_2$
Exact Mass: 214,9582

Bromine (2.1 mL, 6.52 g, 40.81 mmol, 1.25 equiv) was added dropwise to triphenylphosphine (9.42 g, 35.92 mmol, 1.1 equiv) in CH_2Cl_2 (120 mL) under an argon atm. at 0°C. The mixture was allowed to warm up to 25 °C and stirred for 90 min. Afterwards, the mixture was cooled to -78°C and (4-nitrophenyl)methanol (**1**) (5 g, 32.65 mmol, 1.0 equiv) dissolved in dry CH_2Cl_2 (180 mL) was added slowly. After allowing the mixture to warm up to 25 °C over 18 h, aq. $Na_2S_2O_3$ (0.95 M, 150 mL) and aq. $NaHCO_3$ (1.15 M, 150 mL) were added, and the mixture was stirred vigorously until the yellow color disappeared. The phases were separated, and the aqueous phase was extracted with CH_2Cl_2 (2 x 150 mL). The combined organic phases were dried over Na_2SO_4, filtered, and concentrated under reduced pressure. The residue was purified by flash chromatography through silica gel (EtOAc:Hex/1:4) yielding 1-(bromomethyl)-4-nitrobenzene (**2**) as a white solid (6.71 g, 31.06 mmol, 95%).

TLC (EtOAc:Hex/1:9) R_f = 0.31 [UV^{254}]. **^{1}H-NMR** (300 MHz, $CDCl_3$) δ [ppm]: 8.23 – 8.17 (m, 2H, C_{Ar}-**H**), 7.60 – 7.51 (m, 2H, C_{Ar}-**H**), 4.52 (s, 2H, C**H**$_2$-Br). **^{13}C-NMR** (75 MHz, $CDCl_3$) δ [ppm]: 147.8 (**C**$_{Ar}$-NO_2), 144.9 (**C**$_{Ar}$-CH_2Br), 130.1 (**C**$_{Ar}$-H), 124.2 (**C**$_{Ar}$-H), 31.1 (**C**H_2-Br). The analytic data were in accordance with prior published data.[3]

***S*-(4-Nitrobenzyl)ethanethioate**

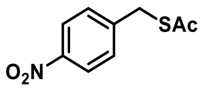

Chemical Formula: $C_9H_9NO_3S$
Exact Mass: 211,0303

Potassium thioacetate (5.32 g, 46.59 mmol, 1.5 equiv) was added to 1-(bromomethyl)-4-nitrobenzene (**2**) (6.71 g, 31.06 mmol, 1.0 equiv) in DMF (155 mL) at 23°C under an argon atm. and stirred for 30 min. Afterwards, the mixture was diluted with water (1900 mL) and extracted with EtOAc (3 x 330 mL). The combined organic phases were dried over Na_2SO_4, filtered, and concentrated under reduced pressure. The residue was purified by flash chromatography through silica gel (EtOAc:Hex/1:4) yielding *S*-(4-nitrobenzyl)ethanethioate (**3**) as an off-white solid (6.71 g, 31.06 mmol, 97%).

TLC (EtOAc:Hex/1:5) R_f = 0.46 [UV^{254}, CAM, $KMnO_4$]. **^{1}H-NMR** (300 MHz, $CDCl_3$) δ [ppm]: 8.21 – 8.10 (m, 2H, C_{Ar}-**H**), 7.52 – 7.40 (m, C_{Ar}-**H**), 4.16 (s, C_{Ar}-C**H**$_2$-SAc), 2.37 (s, 3H, -S(C=O)C**H**$_3$). **^{13}C-NMR** (75 MHz, $CDCl_3$) δ [ppm]: 194.5 (-S(**C**=O)CH_3), 147.3 (**C**$_{Ar}$-NO_2), 145.7 (**C**$_{Ar}$-**C**H_2SAc), 129.8 (C_{Ar}-**H**), 124.0 (C_{Ar}-**H**), 32.9 (C_{Ar}-**C**H_2Sac), 30.5(-S(C=O)**C**H_3). The analytic data were in accordance with prior published data.[4]

(4-Nitrophenyl)methanethiol (4)

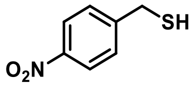

Chemical Formula: $C_7H_7NO_2S$
Exact Mass: 169,0197

$HCl_{(MeOH)}$ (0.5 M, 3.12 mL, 1.56 mmol, 1.0 equiv) was added to (4-nitrobenzyl)ethanethioate (**3**) (0.33 g, 1.56 mmol, 1.0 equiv) in degassed MeOH (4.7 mL) at 23°C under an argon atm. and the mixture was heated to 60°C for 14 h. Afterwards, the mixture was concentrated under reduced pressure and the residue was purified by flash chromatography through silica gel (EtOAc:Hex/1:4) yielding (4-nitrophenyl)methanethiol (**4**) as an off-white solid (257 mg, 1.52 mmol, 97%).

TLC (EtOAc:Hex/1:5) R_f = 0.55 [UV^{254}, CAM, $KMnO_4$]. **^{1}H-NMR** (300 MHz, $CDCl_3$) δ [ppm]: 8.23 – 8.14 (m, 2H, C_{Ar}-**H**), 7.54 – 7.46 (m, 2H, C_{Ar}-**H**), 3.82 (d, J = 7.8 Hz, 2H, -C**H**$_2$-SH), 1.83 (t, J = 7.9 Hz, 1H, -CH_2-S**H**). **^{13}C-NMR** (75 MHz, $CDCl_3$) δ [ppm]: 148.6 (**C**$_{Ar}$-NO_2), 147.1(**C**$_{Ar}$-CH_2-SH), 129.1 (**C**$_{Ar}$-H), 124.1 (**C**$_{Ar}$-H), 28.6 (-**C**H_2-SH). The analytic data were in accordance with prior published data.[4]

2-(4-Azidophenyl)ethan-1-ol (6)

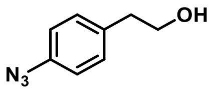

Chemical Formula: $C_8H_9N_3O$
Exact Mass: 163,0746

A solution of $NaNO_2$ (1.51 g, 21.87 mmol, 1.5 equiv) in water (60 mL) was added dropwise to 2-(4-aminophenyl)ethan-1-ol (**5**) (2 g, 14.58 mmol, 1.0 equiv) in aq. HCl (6 M, 24 mL) at 0°C and the mixture was stirred for 30 min. A solution of NaN_3 (3.79 g, 58.32 mmol, 4.0 equiv) in water (100 mL) was added dropwise. After stirring for 2 h the mixture was extracted with Et_2O (3 x 50 mL). The combined organic phases were washed with sat. aq. $NaHCO_3$ (100 mL), brine (100 mL), dried over Na_2SO_4, filtered and concentrated under reduced pressure yielding 2-(4-azidophenyl)ethan-1-ol (**6**) as an orange liquid (2.25 g, 13.79 mmol, 95 %) which was used in the next step without further purification.

TLC (EtOAc:Hex/2:3) R_f = 0.45 [UV^{254}, CAM, $KMnO_4$]. **^{1}H-NMR** (300 MHz, $CDCl_3$) δ [ppm]: 7.24 – 7.18 (m, 2H, C_{Ar}-**H**), 7.03 – 6.92 (m, 2H, C_{Ar}-**H**), 3.83 (t, J = 6.5 Hz, 2H, C_{Ar}-CH_2-C**H**$_2$-OH), 2.84 (t, J = 6.5 Hz, 2H, C_{Ar}-C**H**$_2$-CH_2-OH). The analytic data were in accordance with prior published data.[5]

2-(4-Azidophenyl)acetaldehyde (7)

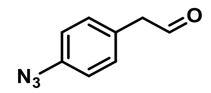

Chemical Formula: $C_8H_7N_3O$
Exact Mass: 161,0589

IBX (4.19 g, 14.97 mmol, 1.1 equiv) was added to 2-(4-azidophenyl)ethan-1-ol (**6**) (2.22 g, 13.6 mmol, 1.0 equiv) in DMSO (27 mL) at 23°C under an argon atm. and the mixture was stirred for 17 h. Afterwards, the mixture was filtered through a patch of celite. The celite was rinsed with CH_2Cl_2 (50 mL). The combined filtrates were washed with water (3 x 400 mL), sat. aq. $NaHCO_3$ (100 mL), brine (100 mL), dried over Na_2SO_4, filtered and concentrated under reduced pressure. The residue was purified by flash chromatography through silica gel (EtOAc:Hex/1:9) yielding 2-(4-azidophenyl)acetaldehyde (**7**) as a cloudy, off-white liquid (986 mg, 6.12 mmol, 45 %).

TLC (EtOAc:Hex/1:5) R_f = 0.62 [UV^{254}, $KMnO_4$]. **^{1}H-NMR** (300 MHz, $CDCl_3$) δ [ppm]: 9.75 (t, J = 2.2 Hz, 1H, -C**H**O), 7.24 – 7.17 (m, 2H, C_{Ar}-**H**), 7.09 – 6.96 (m, 2H, C_{Ar}-**H**), 3.69 (d, J = 2.2 Hz, 2H, C_{Ar}-C**H**$_2$-CHO).

2-(4-Azidophenyl)acetaldehyde oxime (8)

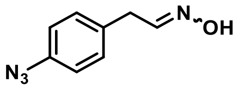

Chemical Formula: $C_8H_8N_4O$
Exact Mass: 176,0698

A solution of 2-(4-azidophenyl)acetaldehyde (**7**) (300 mg, 1.86 mmol, 1.0 equiv) in MeCN (4.65 mL) was added to hydroxylammonium chloride (155 mg, 2.23 mmol, 1.2 equiv) and NaOAc (152 mg, 1.86 mmol, 1.0 equiv) in water (1.6 mL) at 23°C and the mixture was stirred for 26 h. Afterwards, the mixture was extracted with CH_2Cl_2 (3 x 40 mL). The combined organic phases were dried over Na_2SO_4, filtered and concentrated under reduced pressure. The residue was purified by flash chromatography through silica gel (EtOAc:Hex/1:19 →1:1) yielding 2-(4-azidophenyl)-acetaldehyde oxime (**8**) in a 1:1-mixture of (E)- and (Z)-isomers as an off-white crystalline solid (303 mg, 1.72 mmol, 92 %).

TLC (EtOAc:Hex/1:5) R_f = 0.44 [UV^{254}, $KMnO_4$]. **^{1}H-NMR** (300 MHz, $CDCl_3$) δ [ppm]: 7.52 (t, J = 6.2 Hz, 1H, -C**H**=NHOH of (E)-isomer), 7.25 – 7.14 (m, 4H, C_{Ar}-**H** of (E)-and (Z)-isomer), 7.08 – 6.93 (m, 4H, C_{Ar}-**H** of (E)-and (Z)-isomer), 6.87 (t, J = 5.4 Hz, 1H-C**H**=NHOH of (Z)-isomer), 3.71 (d, J = 5.4 Hz, 2H, C_{Ar}-C**H**$_2$-CH=NHOH of (E)-isomer), 3.51 (d, J = 6.2 Hz, 2H, C_{Ar}-C**H**$_2$-CH=NHOH of (Z)-isomer). **^{13}C-NMR** (75 MHz, $CDCl_3$) δ [ppm]: 150.6 (**C**$_{Ar}$-N_3), 138.9 (**C**$_{Ar}$-H), 133.4 (**C**$_{Ar}$-CH_2-CH=NHOH), 132.9 (**C**$_{Ar}$-CH_2-CH=NHOH), 130.4 (**C**$_{Ar}$-H), 130.3(**C**$_{Ar}$-H), 119.51 (**C**$_{Ar}$-H), 119.48 (**C**$_{Ar}$-H), 35.4 (C_{Ar}-**C**H$_2$-CH=NHOH of (E)-isomer), 31.1 (C_{Ar}-**C**H$_2$-CH=NHOH of (Z)-isomer).

4-Nitrobenzyl (*Z*)-2-(4-azidophenyl)-*N*-hydroxyethanimidothioate (9)

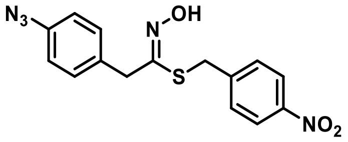

Chemical Formula: $C_{15}H_{13}N_5O_3S$
Exact Mass: 343,0739

NCS (130 mg, 927 µmol, 1.05 equiv) was added to 2-(4-azidophenyl)acetaldehyde oxime (**8**) (163 mg, 927 µmol, 1.0 equiv) in DMF (1 mL) at 23°C under an argon atm and the mixture was stirred for 1 h under light exclusion. Afterwards, the mixture was diluted with water (120 mL) and extracted with Et_2O (3 x 30 mL). The combined organic phases were dried over Na_2SO_4, filtered and concentrated under reduced pressure yielding 2-(4-azidophenyl)-*N*-hydroxyacetimidoyl chloride as a yellowish oil which was used in the next step without further purification.

(4-Nitrophenyl)methanethiol (**4**) (112 mg, 662 µmol, 1.0 equiv) and dry DIPEA (675 µL, 513 mg, 3.97 mmol, 6.0 equiv) in dry THF (2.5 mL) were added to 2-(4-azidophenyl)-*N*-hydroxyacetimidoyl chloride (195 mg, 927 µmol, 1.4 equiv) in THF (2.5 mL) at 23°C under an argon atm. and the mixture was stirred for 26 h under light exclusion. The mixture was poured into ice-cold water (60 mL) and extracted with Et_2O (3 x 40 mL). The combined organic phases were washed with sat. aq. NH_4Cl (60 mL), brine (60 mL), dried over Na_2SO_4, filtered and concentrated under reduced pressure. The residue was purified by flash chromatography through silica gel (EtOAc:Hex/1:19→3:7) yielding 4-nitrobenzyl (Z)-2-(4-azidophenyl)-N-hydroxyethanimido-thioate (**9**) as an off-white solid (155 mg, 451 µmol, 68 %).

TLC (EtOAc:Hex/1:1) R_f = 0.65 [UV^{254}, $KMnO_4$]. ^{1}**H-NMR** (300 MHz, MeOD-d_4) δ [ppm]: 8.10 – 8.02 (m, 2H, C_{Ar1}-**H**), 7.42 – 7.33 (m, 2H, C_{Ar1}-**H**), 7.25 – 7.17 (m, 2H, C_{Ar2}-**H**), 6.97 – 6.88 (m, 2H, C_{Ar2}-**H**), 4.12 (s, 2H, C_{Ar1}-C**H**$_2$-C(=N-OH)S-CH_2-C_{Ar2}), 3.70 (s, 2H, C_{Ar1}-CH_2-C(=N-OH)S-C**H**$_2$-C_{Ar2}). ^{13}**C-NMR** (76 MHz, MeOD-d_4) δ [ppm]: 152.0 (-**C**(=N-OH)S-CH_2-C_{Ar2}), 148.4 (**C**$_{Ar2}$-NO_2), 146.8 (**C**$_{Ar2}$-CH_2-S-), 140.1 (**C**$_{Ar1}$-N_3), 134.7 (**C**$_{Ar1}$-CH_2-), 131.0 (C_{Ar1}-**H**), 130.7 (C_{Ar2}-**H**), 124.6 (C_{Ar2}-**H**), 120.1(C_{Ar1}-**H**), 39.7 (C_{Ar1}-**C**H_2-C(=N-OH)S-CH_2-C_{Ar2}), 34.2 (C_{Ar1}-CH_2-C(=N-OH)S-**C**H_2-C_{Ar2}). **HRMS** (ESI) [m/z]: 366.06347, calculated 366.06313 for $C_{15}H_{13}N_5NaO_3S$ $[M+Na]^+$.

Potassium (*Z*)-(2-(4-azidophenyl)-1-((4-nitrobenzyl)thio)ethylidene)amino sulfate (*ps*GSL(NO$_2$)-N$_3$)

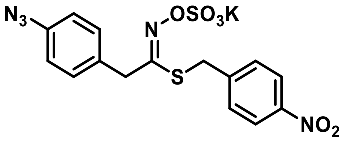

Chemical Formula: $C_{15}H_{12}KN_5O_6S_2$
Exact Mass: 460,9866

Sulfur trioxide pyridinium complex (359 mg, 2.26 mmol, 5.0 equiv) and pyridine (364 µL, 357 mg, 4.51 mmol, 10.0 equiv) were added to 4-nitrobenzyl (*Z*)-2-(4-azidophenyl)-*N*-hydroxyethaneimidothioate (**9**) (155 mg, 451 µmol, 1.0 equiv) in THF (900 µL) at 23°C under an argon atm. and heated to 60°C for 3 h. Afterwards, the mixture was allowed to cool down to 23°C and aq. $KHCO_3$ (2 M, 3.61 mL) was added. The mixture was stirred vigorously for 30 min, before it was concentrated under reduced pressure. The residue was purified by flash chromatography through silica gel (solid load, $MeOH:CH_2Cl_2$/5:100→20:100) yielding ***ps*GSL(NO$_2$)-N$_3$** as a yellow resin-like solid (118 mg, 256 µmol, 57 %).

TLC ($MeOH:CH_2Cl_2$/20:100) R_f = 0.29 [UV^{254}, $KMnO_4$, CAM]. ^{1}**H-NMR** (300 MHz, MeOD-d_4) δ [ppm]: 8.12 – 8.03 (m, 2H, C_{Ar1}-**H**), 7.46 – 7.37 (m, 2H, C_{Ar1}-**H**), 7.34 – 7.25 (m, 2H, C_{Ar2}-**H**), 7.01 – 6.90 (m, 2H, C_{Ar2}-**H**), 4.17 (s, 2H, C_{Ar1}-C**H**$_2$-C(=N-OSO_3K)-S-CH_2-C_{Ar2}), 3.84 (s, 2H, C_{Ar1}-CH_2-C(=N-OSO_3K)-S-C**H**$_2$-C_{Ar2}). ^{13}**C-NMR** (76 MHz, MeOD-d_4) δ [ppm]: 159.3(-**C**(=N-OH)-S-CH_2-C_{Ar2}), 145.8 (**C**$_{Ar2}$-NO_2), 140.4 (**C**$_{Ar2}$-CH_2-S-), 133.7 (**C**$_{Ar1}$-N_3), 131.2 (**C**$_{Ar1}$-CH_2-), 130.9 (**C**$_{Ar1}$-H), 124.6 (**C**$_{Ar2}$-H), 120.3 (**C**$_{Ar1}$-H), 39.6 (C_{Ar1}-**C**H_2-C(=N-OSO_3K)-S-CH_2-C_{Ar2}), 34.8 (C_{Ar1}-CH_2-C(=N-OSO_3K)-S-**C**H_2-C_{Ar2}). **IR** (ATR) [cm^{-1}]: 3497, 2924, 2852, 2110, 1512, 1346, 1229, 1063, 795, 720, 629, 570. **HRMS** (ESI) [m/z]: 468.00216, calculated 468.00189 for $C_{15}H_{12}N_5O_6S_2Na_2$ $[M-K+2Na]^+$.

Potassium (*Z*)-(2-(4-(4-((4-(5,5-difluoro-1,3,7,9-tetramethyl-5*H*-4λ^4,5λ^4-dipyrrolo[1,2-c:2',1'-*f*][1,3,2]diazaborinin-10-yl)phenoxy)methyl)-1*H*-1,2,3-triazol-1-yl)phenyl)-1-((4-nitrobenzyl)thio)ethylidene)amino sulfate (*ps*GSL(NO$_2$)-BODIPY)

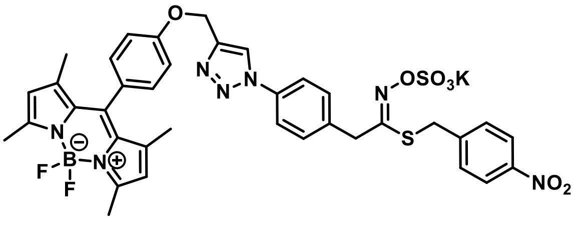

Chemical Formula: $C_{37}H_{33}BF_2KN_7O_7S_2$
Exact Mass: 839,1581

5,5-difluoro-1,3,7,9-tetramethyl-10-(4-(prop-2-yn-1-yloxy)phenyl)-5*H*-4λ^4,5λ^4-dipyrrolo[1,2-c:2',1'-*f*][1,3,2]diazaborinine (**10**) (14.64 mg, 31.73 µmol, 1 equiv), TBTA (1.68 mg, 3.17 µmol, 0.1 equiv; taken from stock solution 168 mg/mL in DMSO), sodium ascorbate (1.26 mg, 6.35 µmol, 0.2 equiv; taken from stock solution 100 mg/mL in water) and $CuSO_4$ (253 µg, 1.59 µmol, 0.05 equiv; taken from stock solution 25 mg/mL in water) were added to Potassium (*Z*)-(2-(4-azidophenyl)-1-((4-nitrobenzyl)thio)-ethylidene)amino sulfate (***ps*GSL(NO$_2$)-N$_3$**) (12 mg, 31.73 µmol, 1.0 equiv) in a mixture of DMSO (264 µL), MeOH (132 µL), THF (132 µL) and water (132 µL) at 23°C and the mixture was stirred for 25 h. Afterwards, the mixture was purified by flash chromatography through silica gel (solid load, $MeOH:CH_2Cl_2$/10:90) yielding ***ps*GSL(NO$_2$)-BODIPY** as a dark-orange solid (12.2 mg, 14.53 µmol, 46 %).

TLC (MeOH:CH_2Cl_2/2:10) R_f = 0.4 [UV^{254}, UV^{366}, $KMnO_4$, CAM]. **^{1}H-NMR** (400 MHz, DMSO-d_6) δ [ppm]: 8.95 (s, 1H, $C_{Triazol}$-**H**), 8.13 – 8.08 (m, 2H, C_{Ar1}-**H**), 7.88 – 7.82 (m, 2H, C_{Ar1}-**H**), 7.55 – 7.50 (m, 2H, C_{Ar2}-**H**), 7.50 – 7.44 (m, 2H, C_{Ar2}-**H**), 7.33 – 7.24 (m, 4H, -C_6**H_4**-BODIPY), 6.17 (s, 2H, $C_{Ar\text{-}BODIPY}$-**H**), 5.31 (s, 2H, -C**H_2**-O-), 4.21 (s, 2H, C_{Ar2}-C**H_2**-C(=N-OSO_3K)-S-C**H_2**-C_{Ar1}), 3.97 (s, 2H, C_{Ar2}-C**H_2**-C(=N-OSO_3K)-S-CH_2-C_{Ar1}), 2.44 (s, 6H, -$C_{Ar\text{-}BODIPY}$-C**H_3**), 1.40 (s, 6H, -$C_{Ar\text{-}BODIPY}$-C**H_3**). **^{13}C-NMR** (126 MHz, DMSO-d_6) δ [ppm]: 158.6 (C_{Ar2}-CH_2-**C**(=N-OSO_3K)-S-CH_2-C_{Ar1}), 154.7 (-O-**C**$_6H_4$-BODIPY), 153.8 (**C**$_{Ar\text{-}BODIPY}$), 146.5 (**C**$_{Ar1}$-NO_2), 145.0 (**C**$_{Ar\text{-}BODIPY}$), 143.5 (**C**$_{Ar\text{-}BODIPY}$), 142.8 (**C**$_{Ar\text{-}BODIPY}$), 142.0 (**C**$_{Ar2}$-CH_2-S-), 136.8 (**C**$_{Ar1}$-Triazol), 135.3 (-**C**$_6H_4$-BODIPY), 131.1 (**C**$_{Ar1}$-CH_2-), 130.0 (**C**$_{Ar1}$-H), 129.6 (-**C**$_6H_4$-BODIPY), 129.2 (**C**$_{Ar\text{-}BODIPY}$), 126.4 (**C**$_{Ar1}$-H), 123.6 (**C**$_{Ar\text{-}BODIPY}$), 123.0 (**C**$_{Ar\text{-}BODIPY}$), 121.3 (**C**$_{Ar\text{-}BODIPY}$), 120.3 (**C**$_{Ar1}$-H), 115.6 (-**C**$_6H_4$-BODIPY), 61.2 (-**C**H_2-O-), 40.4 (C_{Ar1}-**C**H_2-C(=N-OSO_3K)-S-CH_2-C_{Ar2}), 37.4 (C_{Ar1}-CH_2-C(=N-OSO_3K)-S-**C**H_2-C_{Ar2}), 32.6 (-$C_{Ar\text{-}BODIPY}$-**C**H_3), 14.2 (-$C_{Ar\text{-}BODIPY}$-**C**H_3). **^{19}F-NMR** (377 MHz, DMSO-d_6) δ [ppm]: -143.18 (dd, J = 65.8, 31.6 Hz). **^{11}B-NMR** (128 MHz, DMSO-d_6) δ [ppm]: 1.37 (t, J = 33.2 Hz). **IR** (ATR) [cm^{-1}]: 3466, 2924, 1513, 1345, 1237, 1191, 1058, 977, 792, 628. **HRMS** (ESI) [m/z]: 846.17413, calculated 846.17404 for $C_{37}H_{33}BF_2N_7O_7S_2Na_2$ [M-K+2 Na]$^+$.

2-(2-(2-Azidoethoxy)ethoxy)ethan-1-ol (12)

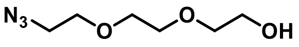

Chemical Formula: $C_6H_{13}N_3O_3$
Exact Mass: 175,0957

2-(2-(2-Chloroethoxy)ethoxy)ethan-1-ol (**11**) (1.77 mL, 2 g, 11.86 mmol, 1.0 equiv) was added to NaN_3 (1.57 g, 23.08 mmol, 2.03 equiv) in water (80 mL) at 23°C and the mixture was heated to 115°C for 20 h. Afterwards, the mixture was cooled to 0°C and extracted with CH_2Cl_2 (3 x 200 mL). The combined organic phases were dried over Na_2SO_4, filtered and concentrated under reduced pressure yielding 2-(2-(2-azidoethoxy)ethoxy)ethan-1-ol (**12**) as a colorless oil (1.97 g, 11.25 mmol, 95%) which was used in the next step without further purification.

TLC (EtOAc:Hex/1:1) R_f = 0.27 [$KMnO_4$]. **^{1}H-NMR** (300 MHz, $CDCl_3$) δ [ppm]: 3.78 – 3.54 (m, 10H, -C**H_2**-O-), 3.37 (t, J = 5.0 Hz, 2H, -C**H_2**-N_3), 2.57 (t, J = 6.0 Hz, 1H, -CH_2-O**H**). **^{13}C-NMR** (75 MHz, $CDCl_3$) δ [ppm]: 72.6, 70.7 (-**C**H_2-O-), 70.4 (-**C**H_2-O-), 70.1 (-**C**H_2-O-), 61.8 (-**C**H_2-OH), 50.7 (-**C**H_2-N_3). The analytic data were in accordance with prior published data.[6]

2-(2-(2-Azidoethoxy)ethoxy)acetic acid (13)

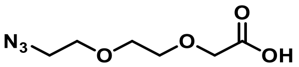

Chemical Formula: $C_6H_{11}N_3O_4$
Exact Mass: 189,0750

Jones reagent (3 M CrO_3 in 20 % $H_2SO_{4(aq)}$, 11.0 mL, 33.06 mmol, 3.0 equiv) was added slowly to 2-(2-(2-azidoethoxy)ethoxy)ethan-1-ol (**12**) (1.93 g, 11.02 mmol, 1.0 equiv) in acetone (110 mL) at 0°C. The mixture was allowed to warm up to 23°C and stirred for 18 h. Isopropanol (5 mL) was added, and the mixture was stirred for 30 min. Afterwards, the mixture was filtered through a patch of celite. The combined filtrates were concentrated under reduced pressure and the residue was purified by flash chromatography through silica gel (MeOH:CH_2Cl_2/5:100) yielding 2-(2-(2-azidoethoxy)ethoxy)acetic acid (**13**) as a colorless oil (1.92 g, 10.5 mmol, 92%).

^{1}H-NMR (300 MHz, $CDCl_3$) δ [ppm]: 8.68 (s, 1H, -CO_2**H**), 4.20 (s, 2H, -O-C**H_2**-CO_2H), 3.79 – 3.74 (m, 2H, N_3-CH_2-C**H_2**-O-), 3.73 – 3.66 (m, 4H, -O-C**H_2**-C**H_2**-O-), 3.46 – 3.35 (m, 2H, N_3-C**H_2**-). **^{13}C-NMR** (75 MHz, $CDCl_3$) δ [ppm]: 174.4 (-**C**O_2H), 71.3 (-O-**C**H_2-), 70.6 (-O-**C**H_2-), 70.2 (-O-**C**H_2-), 68.6 (-O-**C**H_2-CO_2H), 50.7 (N_3-**C**H_2-). The analytic data were in accordance with prior published data.[7]

2-(2-(2-Azidoethoxy)ethoxy)-*N*-(4-(2-hydroxyethyl)phenyl)acetamide (14)

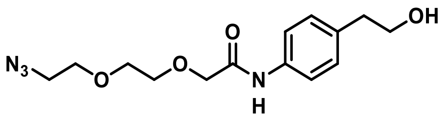

Chemical Formula: $C_{14}H_{20}N_4O_4$
Exact Mass: 308,1485

NMM (64 µL, 58.8 mg, 0.58 mmol, 1.1 equiv) and *iso*-butyl chloroformate (75 µL, 79.4 mg, 0.58 mmol, 1.1 equiv) were added to 2-(2-(2-azidoethoxy)ethoxy)acetic acid (**13**) (100 mg, 0.53 mmol, 1.0 equiv) in THF (4 mL) at -40°C under an argon and the mixture was stirred for 3 h. NMM (70 µL, 64.2 mg, 0.63 mmol, 1.2 equiv) and 2-(4-aminophenyl)ethan-1-ol (**5**) (87 mg, 0.63 mmol, 1.2 equiv) were added and the mixture was allowed to warm up to 23°C over 23 h. Afterwards, the mixture was concentrated under reduced pressure. The residue was purified by flash chromatography through silica gel (EtOAc:Hex/1:1)

yielding 2-(2-(2-azidoethoxy)ethoxy)-*N*-(4-(2-hydroxyethyl)phenyl)acetamide (**14**) as a colorless viscous oil (103 mg, 0.33 mmol, 63%).

TLC (MeOH:CH_2Cl_2/1:9) R_f = 0.7 [UV^{254}, $KMnO_4$]. **IR** (ATR) [cm^{-1}]: 3376, 2922, 2872, 2101, 1671, 1526, 1106, 1045, 912, 819, 728. **HRMS** (ESI) [m/z]: 331.13784, calculated 331.13768 for $C_{14}H_{20}N_4NaO_4$ $[M+Na]^+$. **^{1}H-NMR** (300 MHz, $CDCl_3$) δ [ppm]: 8.55 (s, 1H, -CON**H**-), 7.55 – 7.48 (m, 2H, C_{Ar}-**H**), 7.23 – 7.16 (m, 2H, C_{Ar}-**H**), 4.11 (s, 2H, -O-C**H**$_2$-CO_2H), 3.82 (t, J = 6.6 Hz, 2H, -C**H**$_2$-OH), 3.79 – 3.75 (m, 2H, N_3-CH_2-C**H**$_2$-O-), 3.75 – 3.69 (m, 4H, -C**H**$_2$-O-), 3.45 – 3.39 (m, 2H, -C**H**$_2$-N_3), 2.83 (t, J = 6.6 Hz, 2H, C_{Ar}-C**H**$_2$-). **^{13}C-NMR** (75 MHz, $CDCl_3$) δ [ppm]: 168.0 (-**C**ONH-), 135.8 (-NH-**C**$_{Ar}$), 134.9 (-**C**$_{Ar}$-CH_2-), 129.7 (**C**$_{Ar}$-H), 120.5 (**C**$_{Ar}$-H), 71.1 (-**C**H_2-O-), 70.7 (-**C**H_2-O-), 70.30 (-**C**H_2-O-), 70.25 (-O-**C**H_2-CONH-), 63.8 (-**C**H_2-OH), 50.7 (-**C**H_2-N_3), 38.8 (C_{Ar}-**C**H_2-).

2-(2-(2-Azidoethoxy)ethoxy)-*N*-(4-(2-oxoethyl)phenyl)acetamide (15)

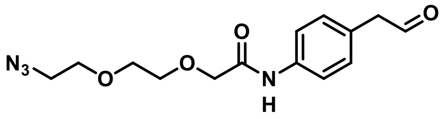

Chemical Formula: $C_{14}H_{18}N_4O_4$
Exact Mass: 306,1328

2-(2-(2-Azidoethoxy)ethoxy)-*N*-(4-(2-hydroxyethyl)phenyl)acetamide (**14**) (1.7 g, 5.51 mmol, 1.0 equiv) was added to IBX (1.7 g, 6.06 mol, 1.1 equiv) in DMSO (5.5 mL) at 23°C and the mixture was stirred for 3 h. CH_2Cl_2 (18 mL) was added and the mixture was stirred for 45 min. Afterwards, the precipitate was filtered off. The combined filtrates were washed with water (3 x 100 mL), sat. aq. $NaHCO_3$ (100 mL), brine (100 mL), dried over Na_2SO_4, filtered, and concentrated under reduced pressure. 2-(2-(2-azidoethoxy)ethoxy)-*N*-(4-(2-oxo-ethyl)phenyl)acetamide (**15**) was obtained as a colorless viscous oil (1.44 g, 4.72 mmol, 86%) and used directly without further purification.

TLC (EtOAc:Hex/3:1) R_f = 0.5 [UV^{254}, $KMnO_4$, CAM]. **IR** (ATR) [cm^{-1}]: 3467, 2928, 2862, 2107, 1664, 1519, 1348, 1278, 1240, 1063, 799, 639. **HRMS** (ESI) [m/z]: 361.14842, calculated 361.14824 for $C_{15}H_{22}O_5N_4Na$ $[M+MeOH+Na]^+$. **^{1}H-NMR** (300 MHz, $CDCl_3$) δ [ppm]: 9.72 (t, J = 2.3 Hz, 1H, -C**H**O), 8.58 (s, 1H, -CON**H**-), 7.62 – 7.57 (m, 2H, C_{Ar}-**H**), 7.21 – 7.17 (m, 2H, C_{Ar}-**H**), 4.12 (s, 2H, -O-C**H**$_2$-CONH-), 3.80 – 3.77 (m, 2H, N_3-CH_2-C**H**$_2$-O), 3.75 – 3.72 (m, 4H, -C**H**$_2$-O-), 3.66 (d, J = 2.4 Hz, 2H, -C**H**$_2$-N_3), 3.46 – 3.41 (m, 2H, C_{Ar}-C**H**$_2$-).). **^{13}C-NMR** (75 MHz, $CDCl_3$) δ [ppm]: 199.4 (-**C**HO), 168.1 (-**C**ONH-), 136.7 (-NH-**C**$_{Ar}$), 130.3(-**C**$_{Ar}$-CH_2-), 127.9 (**C**$_{Ar}$-H), 120.7 (**C**$_{Ar}$-H), 71.2 (-**C**H_2-O-), 70.7(-**C**H_2-O-), 70.3 (-**C**H_2-O-), 70.3 (-O-**C**H_2-CONH-), 50.7 (-**C**H_2-N_3), 50.1 (C_{Ar}-**C**H_2-CHO).

2-(2-(2-Azidoethoxy)ethoxy)-N-(4-(2-(hydroxyimino)ethyl)phenyl)acetamide (16)

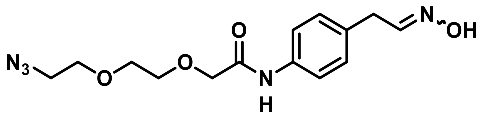

Chemical Formula: $C_{14}H_{19}N_5O_4$
Exact Mass: 321,1437

A solution of 2-(2-(2-azidoethoxy)ethoxy)-*N*-(4-(2-oxoethyl)phenyl)acetamide (**15**) (347 mg, 1.13 mmol, 1.0 equiv) in MeCN (3 mL) was added to hydroxylammonium chloride (94.4 mg, 1.36 mmol, 1.2 equiv) and NaOAc (93 mg, 1.36 mmol, 1.0 equiv) in water (1 mL) at 23°C and the mixture was stirred for 17 h. Afterwards, the mixture was extracted with CH_2Cl_2 (3 x 25 mL). The combined organic phases were dried over Na_2SO_4, filtered, and concentrated under reduced pressure. The residue was purified by flash chromatography through silica gel (EtOAc:Hex/1:1) yielding 2-(2-(2-azidoethoxy)ethoxy)-*N*-(4-(2-(hydroxyimino)ethyl)phenyl)-acetamide (**16**) as an off-white amorphous solid in a 1:1mixture of (E)- and (Z)-isomers (355.8 mg, 1.11 mmol, 98%).

TLC (EtOAc:Hex/1:1) R_f = 0.68 [UV^{254}, $KMnO_4$, PPh_3 + Ninhydrin]. **IR** (ATR) [cm^{-1}]: 3336, 2915, 2104, 1672, 1599, 1528, 1415, 1297, 1252, 1110, 829, 653, 590. **HRMS** (ESI) [m/z]: 344.13312, calculated 344.13293 for $C_{14}H_{19}N_5NaO_4$ $[M+Na]^+$. **^{1}H-NMR** (300 MHz, $CDCl_3$) δ [ppm]: 8.58 (s, 2H, -CON**H**- of (E) and (Z)-isomer), 7.57 – 7.51 (m, 4H, C_{Ar}-**H** of (E) and (Z)-isomer), 7.51 – 7.46 (m, 1H, -C**H**=NOH of (E)-isomer), 7.22 – 7.13 (m, 4H C_{Ar}-**H** of (E) and (Z)-isomer), 6.86 (t, J = 5.4 Hz, 1H, -C**H**=NOH of (Z)-isomer), 4.12 (s, 2H, -O-C**H**$_2$-CONH- of (E)-isomer), 4.12 (s, 2H, -O-C**H**$_2$-CONH- of (Z)-isomer), 3.81 – 3.75 (m, 4H, N_3-CH_2-C**H**$_2$-O- of (E) and (Z)-isomer), 3.75 – 3.72 (m, 6H, -C**H**$_2$-O- of (E) and (Z)-isomer and C_{Ar}-C**H**$_2$- of (E)-isomer), 3.72 – 3.62 (m, 4H, -C**H**$_2$-N_3 of (E) and (Z)-isomer), 3.49 (d, J = 6.3 Hz, 2H, C_{Ar}-C**H**$_2$- of (Z)-isomer), 3.46 – 3.34 (m, 4H, C_{Ar}-C**H**$_2$- of (E) and (Z)-isomer). **^{13}C-NMR** (75 MHz, $CDCl_3$) δ [ppm]: 168.1 (-**C**ONH- of (E) and (Z)-isomer), 150.8 (-**C**H=NOH of (E)-isomer), 150.6 (-**C**H=NOH of (Z)-isomer), 136.1 (-NH-**C**$_{Ar}$ of (E)-isomer), 135.9 (-NH-**C**$_{Ar}$ of (Z)-isomer), 133.0 (-**C**$_{Ar}$-CH_2-), 132.5 (-**C**$_{Ar}$-CH_2- of (Z)-isomer), 129.4 (**C**$_{Ar}$-H of (E)-isomer), 120.6 (**C**$_{Ar}$-H of (E)-isomer), 120.6 (**C**$_{Ar}$-H of (Z)-isomer), 71.1 (-**C**H_2-O- of (E) and (Z)-isomer), 70.7 (-**C**H_2-O- of (E) and (Z)-isomer), 70.3 (-**C**H_2-O- of (E) and (Z)-isomer), 70.2 (-O-**C**H_2-CONH- of (E) and (Z)-isomer), 50.6 (-**C**H_2-N_3 of (E) and (Z)-isomer), 35.5 (C_{Ar}-**C**H_2-CH=NOH of (E)-isomer), 31.2 (C_{Ar}-**C**H_2-CH=NOH of (Z)-isomer).

4-Nitrobenzyl (*Z*)-2-(4-(2-(2-(2-azidoethoxy)ethoxy)acetamido)phenyl)-*N*-hydroxyethan-imidothioate (17)

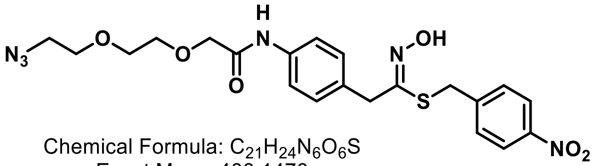

Chemical Formula: $C_{21}H_{24}N_6O_6S$
Exact Mass: 488,1478

NCS (52 mg, 389 µmol, 1.05 equiv) was added to 2-(2-(2-azidoethoxy)ethoxy)-*N*-(4-(2-(hydroxyimino)ethyl)phenyl)acetamide (**16**) (119 mg, 370 µmol, 1.0 equiv) in DMF (370 µL) at 23°C under an argon atm. and the mixture was stirred for 19 h under light exclusion. Afterwards, the mixture was diluted with water (10 mL) and extracted with CH_2Cl_2 (5 x 10 mL). The combined organic phases were dried over Na_2SO_4, filtered and concentrated under reduced pressure yielding (*Z*)-2-(4-(2-(2-(2-azidoethoxy)ethoxy)acetamido)-phenyl)-*N*-hydroxy acetimidoyl chloride as a yellowish oil, which was used in the next step without further purification.

(4-Nitrophenyl)methanethiol (**4**) (44.8 mg, 265 µmol, 1.0 equiv) and dry DIPEA (278 µL, 205.5 mg, 1.59 mmol, 6.0 equiv) in THF (2.0 mL) were added to (*Z*)-2-(4-(2-(2-(2-azidoethoxy)ethoxy)acetamido)phenyl)-*N*-hydroxyacetimidoyl chloride (132 mg, 371 µmol, 1.4 equiv) in THF (2.0 mL) at 23°C under an argon atm. and the mixture was stirred for 16 h under light exclusion. Afterwards, the mixture was poured into ice-cold water (20 mL) and extracted with CH_2Cl_2 (4 x 50 mL). The combined organic phases were dried over Na_2SO_4, filtered, and concentrated under reduced pressure. The residue was purified by flash chromatography through silica gel (EtOAc:Hex/3:1) yielding 4-Nitrobenzyl (*Z*)-2-(4-(2-(2-(2-azido-ethoxy)ethoxy)acetamido)phenyl)-*N*-hydroxyethaneimidothioate (**17**) as an off-white solid (118 mg, 242 µmol, 91%).

TLC (EtOAc:Hex/3:1) R_f = 0.38 [UV^{254}, $KMnO_4$]. **IR** (ATR) [cm^{-1}]: 3335, 2919, 2862, 2103, 1669, 1599, 1522, 1415, 1345, 1309, 1243, 1184, 1106, 978, 900, 856, 808, 726, 662, 588. **HRMS** (ESI) [m/z]: 511.13700, calculated 511.13702 for $C_{21}H_{24}N_6NaO_6S$ $[M+Na]^+$. **^{1}H NMR** (600 MHz, MeOD-*d₄*) δ [ppm]: 8.03 (d, *J* = 8.7 Hz, 2H, C_{Ar}-**H**), 7.47 (d, *J* = 8.5 Hz, 2H, C_{Ar}-**H**), 7.33 (d, *J* = 8.7 Hz, 2H, C_{Ar}-**H**), 7.14 (d, *J* = 8.5 Hz, 2H, C_{Ar}-**H**), 4.14 (s, 2H, -O-C**H**2-CONH-), 4.11 (s, 2H, -S-C**H**2-C_{Ar}), 3.82 – 3.79 (m, 2H, -C**H**2-O-), 3.78 – 3.75 (m, 2H, N_3-CH_2-C**H**2-O-), 3.76 – 3.71 (m, 2H, -C**H**2-O-), 3.70 (s, 2H, -C**H**2-N_3), 3.46 – 3.41 (m, 2H, C_{Ar}-C**H**2-C(=NOH)-S-). **^{13}C NMR** (151 MHz, MeOD) δ [ppm]: 170.9 (-**C**ONH-), 152.3 (-**C**(=N-OH)-S-), 148.3 (**C**Ar-NO_2), 146.8 (-S-CH_2-**C**Ar), 137.6 (-CONH-C_{Ar}), 134.0 (**C**Ar-CH_2-C(=N-OH)-S-), 130.7 (**C**Ar-H), 129.9 (**C**Ar-H), 124.5 (**C**Ar-H), 121.7 (**C**Ar-H), 72.1 (-**C**H2-O-), 71.5 (-**C**H2-O-), 71.3 (-**C**H2-O-), 71.1 (N_3-CH_2-**C**H2-O-), 51.7 (-**C**H2-N_3), 39.8 (C_{Ar}-**C**H2-C(=NOH)-S-), 34.2 (-S-**C**H2-C_{Ar}).

Potassium (*Z*)-(2-(4-(2-(2-(2-azidoethoxy)ethoxy)acetamido)phenyl)-1-((4-nitrobenzyl)thio)ethylidene)amino sulfate (*ps*GSL$_{PEG}$(NO_2)-N_3)

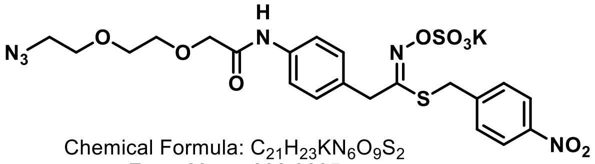

Chemical Formula: $C_{21}H_{23}KN_6O_9S_2$
Exact Mass: 606,0605

Sulfur trioxide pyridinium complex (189 mg, 1.19 mmol, 5.0 equiv) and pyridine (112.05 µL, 188 mg, 2.37 mmol, 10.0 equiv) were added to 4-Nitrobenzyl (*Z*)-2-(4-(2-(2-(2-azidoethoxy)ethoxy)acetamido)phenyl)-*N*-hydroxyethanimido-thioate (**17**) (116 mg, 237.45 µmol, 1.0 equiv) in CH_2Cl_2 (476 µL) at 23°C under an argon atm. and the mixture was heated to 60°C for 6 h. Afterwards, the mixture was allowed to cool down to 23°C and aq. $KHCO_3$ (2 M, 1.9 mL) was added. The mixture was stirred for 30 min, before it was concentrated under reduced pressure. The residue was purified by flash chromatography through silica gel (solid load, MeOH:CH_2Cl_2/1:9→1:4) yielding ***ps*GSL$_{PEG}$(NO_2)-N_3** as a crystalline off-white solid (88 mg, 145 µmol, 61%).

TLC (MeOH:CH_2Cl_2/1:4) R_f = 0.48 [UV^{254}, $KMnO_4$]. **IR** (ATR) [cm^{-1}]: 3430, 3375, 2924, 2106, 1678, 1604, 1522, 1418, 1347, 1242, 1137, 1110, 1062, 966, 933, 888, 795, 722, 639, 574. **HRMS** (ESI) [m/z]: 567.09937, calculated 567.09734 for $C_{21}H_{23}N_6O_9S_2$ $[M-K]^-$. **^{1}H-NMR** (300 MHz, MeOD-d_4) δ [ppm]: 8.07 – 8.01 (m, 2H, C_{Ar}-**H**), 7.53 – 7.46 (m, 2H, C_{Ar}-**H**), 7.39 – 7.33 (m, 2H, C_{Ar}-**H**), 7.27 – 7.21 (m, 2H, C_{Ar}-**H**), 4.17 (s, 2H, -O-C**H**2-CONH-), 4.15 (s, 2H, -S-C**H**2-C_{Ar}), 3.85 (s, 2H, -C**H**2-O-), 3.81 – 3.71 (m, 6H, -C**H**2-N_3, N_3-CH_2-C**H**2-O- and -C**H**2-O-), 3.46 – 3.40 (m, 2H, C_{Ar}-C**H**2-C(=$NOSO_3K$)-S-). **^{13}C-NMR** (75 MHz, MeOD-d_4) δ [ppm]: 170.9 (-**C**ONH-), 159.8 (-**C**(=N-OH)-S-), 148.4 (**C**Ar-NO_2), 145.8 (-S-CH_2-**C**Ar), 137.9 (-CONH-C_{Ar}), 132.9 (**C**Ar-CH_2-C(=N-OSO_3K)-S-), 130.8 (**C**Ar-H), 130.0 (**C**Ar-H), 124.6 (**C**Ar-H), 121.8 (**C**Ar-H), 72.1 (-**C**H2-O-), 71.6 (-**C**H2-O-), 71.3(-**C**H2-O-), 71.1 (N_3-CH_2-**C**H2-O-), 51.7 (-**C**H2-N_3), 39.7 (C_{Ar}-**C**H2-C(=$NOSO_3K$)-S-), 34.2 (-S-**C**H2-C_{Ar}).

4-(Prop-2-yn-1-yloxy)benzaldehyde

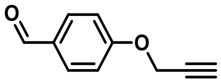

Chemical Formula: $C_{10}H_8O_2$
Exact Mass: 160,0524

K_2CO_3 (4.91 g, 35.54 mmol, 1.4 equiv) and propargyl bromide (5.68 mL (80 % in toluene), 50.77 mmol, 2.0 equiv) were added to 4-Hydroxybenzaldehyde (3.1 g, 25.38 mmol, 1.0 equiv) acetone (127 mL) at 23°C under an argon atm. and the mixture was heated to 65 °C for 2 h. Afterwards, the mixture was concentrated under reduced pressure. The residue was dissolved in EtOAc (120 mL) and washed with water (120 mL). The phases were separated, and the aqueous phase was extracted with EtOAc (2 x 120 mL). The combined organic phases were dried over Na_2SO_4, filtered and concentrated. The residue was purified by flash chromatography through silica gel (EtOAc:Hex/1:9) yielding 4-(Prop-2-yn-1-yloxy)benzaldehyde white crystalline solid (3.44 g, 21.48 mmol, 85 %).

TLC (EtOAc:Hex/1:4) R_f = 0.33 [UV^{254}, $KMnO_4$, CAM]. **^{1}H-NMR** (300 MHz, $CDCl_3$) δ [ppm]: 9.90 (s, 1H, -C**H**O), 7.88 – 7.83 (m, 2H, C_{Ar}-**H**), 7.12 – 7.06 (m, 2H, C_{Ar}-**H**), 4.78 (d, J = 2.4 Hz, 2H, -CH_2-), 2.57 (t, J = 2.4 Hz, 1H, -C≡C-**H**). The analytic data were in accordance with prior published data.[8]

5,5-difluoro-1,3,7,9-tetramethyl-10-(4-(prop-2-yn-1-yloxy)phenyl)-5*H*-4λ⁴,5λ⁴-dipyrrolo-[1,2-c:2',1'-*f*][1,3,2]diazaborinine (10)

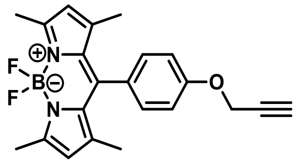

Chemical Formula: $C_{22}H_{21}BF_2N_2O$
Exact Mass: 378,1715

4-(Prop-2-yn-1-yloxy)benzaldehyde (841.73 mg, 5.26 mmol, 1.0 equiv) and TFA (42 µL) were added to 2,4-Dimethylpyrrole (1 g, 10.51 mmol, 2.0 equiv) in dry CH_2Cl_2 (210 mL) at 23°C under an argon atm. and the mixture was stirred for 19 h under light exclusion. *p*-Chloranil (1.55 g, 6.31 mmol, 1.2 equiv) in CH_2Cl_2 (250 mL) was added and the mixture was stirred for 4 h. DIPEA (6.17 mL, 4.69 g, 36.26 mmol, 6.9 equiv) and $BF_3 \cdot OEt_2$ (13.54 mL, 7.31 g, 51.5 mmol, 9.8 equiv) were added and the mixture was stirred for 3 h. Afterwards, the mixture was concentrated under reduced pressure. The residue was dissolved in EtOAc (100 mL) and washed with water (120 mL). The phases were separated, and the aqueous phase was extracted with EtOAc (3 x 100 mL). The combined organic phases were dried over Na_2SO_4, filtered, and concentrated under reduced pressure. The residue was purified by flash chromatography through silica gel (EtOAc:Hex/1:100→1:10) yielding 5,5-difluoro-1,3,7,9-tetramethyl-10-(4-(prop-2-yn-1-yloxy)-phenyl)-5*H*-4λ⁴,5λ⁴-dipyrrolo-[1,2-c:2',1'-*f*][1,3,2]diazaborinine (**10**) as a dark-purple amorphous solid (200 mg, 529 µmol, 10 %).

TLC (EtOAc:Hex/1:4) R_f = 0.44 [UV^{254}, UV^{366}]. **^{1}H-NMR** (300 MHz, $CDCl_3$) δ [ppm]: 7.23 – 7.17 (m, 2H, C_{Ar}-**H**), 7.11 – 7.06 (m, 2H, C_{Ar}-**H**), 5.98 (s, 2H, $C_{Ar\text{-}BODIPY}$-**H**), 4.76 (d, J = 2.4 Hz, 2H, -C**H**$_2$-), 2.55 (s, 6H, -C**H**$_3$), 1.55 (s, 1H, -C≡C-**H**), 1.42 (s, 6H, -C**H**$_3$). **^{13}C-NMR** (76 MHz, $CDCl_3$) δ [ppm]: 158.2 (**C**$_{Ar}$-O-), 155.5 (C_{Ar}=N), 143.3 (C_{Ar}-H), 141.6 (**C**$_{Ar\text{-}BODIPY}$), 131.9 (**C**$_{Ar\text{-}BODIPY}$-H), 130.5 (**C**$_{Ar\text{-}BODIPY}$), 129.4 (C_{Ar}-H), 128.2 (**C**$_{Ar\text{-}BODIPY}$), 121.3 (**C**$_{Ar\text{-}BODIPY}$), 115.8 (**C**$_{Ar}$-H), 78.2 (-**C**≡C-H), 76.0 (-C≡**C**-H), 56.2 (-**C**H$_3$), 14.7 (-**C**H$_3$). **^{19}F-NMR** (282 MHz, $CDCl_3$) δ [ppm]: -146.55, -146.66, -146.78, -146.90. **^{11}B-NMR** (96 MHz, $CDCl_3$) δ [ppm]: 0.99 (t, J = 33.2 Hz). **UV/Vis** (CH_2Cl_2, c = 33 µg/10 mL) λ_{max} = 501 nm, ε = 107571.90 L mol^{-1} cm^{-1}. **Fluorescence emission** (CH_2Cl_2, c = 33 µg/10 mL) λ_{Ex} = 495 nm, λ_{Em} = 515 nm, The analytic data were in accordance with prior published data.[8]

5-(Dimethylamino)-*N*-(prop-2-yn-1-yl)naphthalene-1-sulfonamide (18)

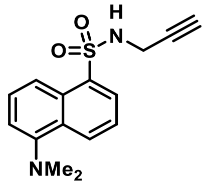

Chemical Formula: $C_{15}H_{16}N_2O_2S$
Exact Mass: 288,0932

Propargylamine (13 µL, 11 mg, 204 µmol, 1.1 equiv) and DIPEA (66 µL, 48 mg, 371 µmol, 2.0 equiv) were added to 5-(Dimethylamino)naphthalene-1-sulfonyl chloride (50 mg, 185.36 µmol, 1.0 equiv) in CH_2Cl_2 (2 mL) at 0°C and the mixture was allowed to warm up to 23°C and stirred for 16 h. Afterwards, the mixture was concentrated under reduced pressure. The

residue was purified by flash chromatography through silica gel (EtOAc:Hex/1:9→3:7) yielding 5-(dimethylamino)-*N*-(prop-2-yn-1-yl)naphthalene-1-sulfonamide (**18**) as a green oil (47.4 mg, 164.4 mmol, 89%).

TLC (EtOAc:Hex/3:7) R_f = 0.4 [UV254, UV366, $KMnO_4$]. **^{1}H-NMR** (300 MHz, $CDCl_3$) δ [ppm]: 8.60 – 8.53 (m, 1H, **H**-C_{Ar}-*p*-SO_2NH-), 8.34 – 8.18 (m, 2H, **H**-C_{Ar}-*o*-SO_2NH- and **H**-C_{Ar}-*p*-NMe_2), 7.63 – 7.49 (m, 2H, **H**-C_{Ar}-*m*-SO_2NH- and **H**-C_{Ar}-*m*-NMe_2), 7.23 – 7.16 (m, 1H, **H**-C_{Ar}-*o*-NMe2), 4.85 (t, J = 6.2 Hz, 1H, -SO_2N**H**-), 3.77 (dd, J = 6.1, 2.5 Hz, 2H, -C**H**$_2$-), 2.89 (s, 6H, -N(C**H**$_3$)$_2$), 1.91 (t, J = 2.5 Hz, 1H, -C≡C-**H**). **^{13}C-NMR** (76 MHz, $CDCl_3$) δ [ppm]: 152.2 (**C**$_{Ar}$-NMe_2), 134.3 (**C**$_{Ar}$-SO_2NH-), 131.0 (**C**$_{Ar}$), 130.1 (**C**$_{Ar}$-H), 130.0 (**C**$_{Ar}$-H), 129.9 (**C**$_{Ar}$-H), 128.7 (**C**$_{Ar}$-H), 123.3 (**C**$_{Ar}$-H), 118.7 (**C**$_{Ar}$), 115.4 (**C**$_{Ar}$-H), 77.9 (-**C**≡C-H), 72.8 (-C≡**C**-H), 45.6 (-**C**H$_2$-), 33.2 (-N(**C**H$_3$)$_2$). The analytic data were in accordance with prior published data.[9]

Potassium (*Z*)-(2-(4-(2-(2-(2-(4-((4-(5,5-difluoro-1,3,7,9-tetramethyl-5*H*-4λ⁴,5λ⁴-dipyrrolo-[1,2-*c*:2',1'-*f*][1,3,2]diazaborinin-10-yl)phenoxy)methyl)-1*H*-1,2,3-triazol-1-yl)ethoxy)-ethoxy)acetamido)phenyl)-1-((4-nitrobenzyl)thio)ethylidene)amino sulfate (*ps*GSL$_{PEG}$(NO$_2$)-BODIPY)

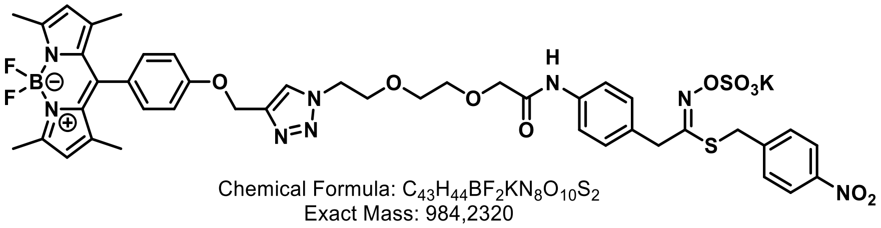

5,5-difluoro-1,3,7,9-tetramethyl-10-(4-(prop-2-yn-1-yloxy)phenyl)-5*H*-4λ⁴,5λ⁴-dipyrrolo[1,2-c:2',1'-*f*][1,3,2]diazaborinine (**10**) (7.5 mg, 19.8 µmol, 1.2 equiv), TBTA (875 µg, 1.65 µmol, 0.1 equiv; taken from stock solution 168 mg/mL in DMSO), sodium ascorbate (653 µg, 3.3 µmol, 0.2 equiv; taken from stock solution 100 mg/mL in H_2O) and $CuSO_4$ (132 µg, 830 nmol, 0.05 equiv; taken from stock solution 25.3 mg/mL in H_2O) were added to potassium (*Z*)-(2-(4-(2-(2-(2-azidoethoxy)ethoxy)acetamido)phenyl)-1-((4-nitrobenzyl)thio)e-thylidene)amino sulfate (***ps*GSL$_{PEG}$(NO$_2$)-N$_3$**) (10 mg, 16.48 µmol, 1.0 equiv) in a mixture of DMSO (825 µL), MeOH (55 µL), THF (55 µL) and water (110 µL) at 23°C and the mixture was stirred for 3 h. Afterwards, the mixture was loaded onto silica and purified by flash chromatography through silica gel (MeOH:CH_2Cl_2/0:1→1:9→1:4) yielding ***ps*GSL$_{PEG}$(NO$_2$)-BODIPY** as a dark-orange solid (16.4 mg, 16.7 µmol, 100%).

TLC (MeOH:CH_2Cl_2/1:4) R_f = 0.73 [UV254, $KMnO_4$]. **IR** (ATR) [cm^{-1}]: 2926, 2863, 1680, 1609, 1543, 1516, 1466, 1410, 1349, 1306, 1284, 1190, 1150, 1113, 1061, 980, 886, 832, 795, 753, 642, 575. **HRMS** (ESI) [m/z]: 991.24872, calculated 991.24728 for $C_{43}H_{44}BF_2N_8O_{10}S_2Na_2$ [M-K+2Na]$^+$. **^{1}H-NMR** (500 MHz, DMSO-d$_6$) δ [ppm]: 9.65 (s, 1H, -CONH-), 8.27 (s, 1H, C$_{Ar-Triazol}$-**H**), 8.14 – 8.08 (m, 2H, C$_{Ar}$-**H**), 7.60 – 7.55 (m, 2H, C$_{Ar}$-**H**), 7.50 – 7.44 (m, 2H, C$_{Ar}$-**H**), 7.29 – 7.24 (m, 2H, C$_{Ar}$-**H**), 7.23 – 7.18 (m, 4H, C$_{Ar}$-**H**), 6.17 (s, 2H, C$_{Ar-BODIPY}$-**H**), 5.18 (s, 2H, -O-C**H**$_2$-C$_{Ar-Triazol}$), 4.59 (t, J = 5.2 Hz, 2H, -O-C**H**$_2$-CH$_2$-N$_{Triazol}$), 4.12 (s, 2H, -S-C**H**$_2$-C$_{Ar}$), 4.06 (s, 2H, -O-C**H**$_2$-CONH-), 3.88 (t, J = 5.2 Hz, 2H, -O-CH$_2$-C**H**$_2$-N$_{Triazol}$), 3.79 (s, 2H, -C**H**$_2$-O-),), 3.67 – 3.62 (m, 4H, C$_{Ar}$-C**H**$_2$-C(=NOH)-S- and -C**H**$_2$-O), 2.44 (s, 6H, -C**H**$_3$), 1.38 (s, 6H, -C**H**$_3$). **^{13}C-NMR** (126 MHz, DMSO-d$_6$) δ [ppm]: 168.1 (-**C**ONH-), 158.7 (-**C**(=N-OH)-S-), 154.7 (**C**$_{Ar}$-O-), 154.3 (C$_{Ar}$=N), 146.5 (**C**$_{Ar}$-NO$_2$), 145.0 (-S-CH$_2$-**C**$_{Ar}$), 142.8 (C$_{Ar}$-H), 142.2 (**C**$_{Ar-BODIPY}$), 142.5 (**C**$_{Ar-Triazol}$), 137.2 (-CONH-C$_{Ar}$), 131.1 (**C**$_{Ar}$-CH$_2$-C(=N-OSO$_3$K)-S-), 130.9 (**C**$_{Ar-BODIPY}$-H), 129.9 (**C**$_{Ar}$-H), 129.1 (**C**$_{Ar}$-H), 128.4 (**C**$_{Ar-BODIPY}$), 126.3 (**C**$_{Ar-BODIPY}$), 125.2 (C$_{Ar}$-H), 123.6 (C$_{Ar}$-H), 121.3 (**C**$_{Ar-BODIPY}$), 119.8 (C$_{Ar}$-H), 115.4 (C$_{Ar}$-H), 70.18 (-**C**H$_2$-O-), 70.17 (-**C**H$_2$-O-), 69.4 (-**C**H$_2$-O-), 68.7 (N$_{Triazol}$-CH$_2$-**C**H$_2$-O-), 61.2 (-**C**H$_3$), 49.4 (-**C**H$_2$-N$_{Triazol}$), 48.6, 37.4 (C$_{Ar}$-**C**H$_2$-C(=NOH)-S-), 32.5 (-S-**C**H$_2$-C$_{Ar}$), 14.2 (-**C**H$_3$). **^{11}B-NMR** (161 MHz, DMSO-d$_6$) δ [ppm]: 1.35 (t, J = 33.2 Hz). **^{19}F-NMR** (377 MHz, DMSO-d$_6$) δ [ppm]: -143.18 (dd, J = 65.7, 31.5 Hz). **UV/Vis** (H_2O, c = 45 µg/10 mL) λ_{max} = 500 nm, ε = 52673.44 L mol^{-1} cm^{-1}. **Fluorescence emission** (H_2O, c = 45 µg/10 mL) λ_{Ex} = 485 nm, λ_{Em} = 510 nm.

Potassium (*Z*)-(2-(4-(2-(2-(2-(4-(((5-(dimethylamino)naphthalene)-1-sulfonamido)-methyl)-1*H*-1,2,3-triazol-1-yl)ethoxy)ethoxy)-acetamido)phenyl)-1-((4-nitrobenzyl)thio)-ethylidene)amino sulfate (*ps*GSL$_{PEG}$(NO$_2$)-DNSA)

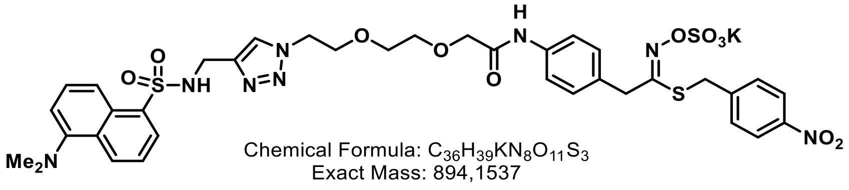

5-(Dimethylamino)-*N*-(prop-2-yn-1-yl)naphthalene-1-sulfonamide (**18**) (5.7 mg, 19.8 µmol, 1.2 equiv), TBTA (0.87 mg, 1.65 µmol, 0.1 equiv; taken from stock solution 168 mg/mL in DMSO), sodium ascorbate (0.63 mg, 3.3 µmol, 0.2 equiv; taken from stock solution 100 mg/mL in H_2O) and $CuSO_4$ (181 µg, 0.83 µmol, 0.05 equiv; taken from stock solution 25.3 mg/mL in H_2O) were added to potassium (*Z*)-(2-(4-(2-(2-(2-azidoethoxy)ethoxy)acetamido)phenyl)-1-((4-nitrobenzyl)thio)e-thylidene)amino sulfate (***ps*GSL$_{PEG}$(NO$_2$)-N$_3$**)(10 mg, 16.5 µmol, 1.0 equiv) in a solvent mixture of DMSO (275 µL) and water (55 µL) at 23°C and the mixture was stirred for 16 h. Afterwards, the mixture was diluted with water (500 µl), MeOH (100 µl) and DMSO (100 µl) and filtered through a 45° µm Whatman®filter. The filtrate was purified by preparative HPLC (Hypersil

Gold, RP-18, MeCN:H_2O/10:90 → 95:5 + 0.01% TFA in 45 min) and the product containing fractions were lyophilized yielding ***ps*GSL$_{PEG}$(NO_2)-DNSA** as an off-white crystalline solid (12.2 mg, 13.6 µmol, 83%).

TLC (MeOH:CH_2Cl_2/1:4) R_f = 0.44 [UV^{254}, UV^{366}, $KMnO_4$]. **IR** (ATR) [cm^{-1}]: 2923, 2854, 2346, 1741, 1684, 1603, 1574, 1521, 1459, 1414, 1348, 1316, 1238, 1143, 1105, 1060, 938, 794, 754, 630, 580, 554, 538. **HRMS** (ESI) [m/z]: 901.16980, calculated 901.16903 for $C_{36}H_{39}N_8O_{11}S_3Na_2$ [M-K+2Na]$^+$. **^{1}H-NMR** (500 MHz, DMSO-d_6) δ [ppm]: 9.62 (s, 1H, -CON**H**-), 8.54 – 8.49 (m, 1H, **H**-C_{Ar}-*p*-SO_2NH-), 8.47 – 8.44 (m, 1H, **H**-C_{Ar}-*o*-SO_2NH-), 8.43 – 8.38 (m, 1H, **H**-C_{Ar}-*p*-NMe_2), 8.16 – 8.13 (m, 1H, -SO_2N**H**-), 8.11 – 8.08 (m, 2H, C_{Ar}-**H**), 7.74 (s, 1H, $C_{Ar-Triazol}$-H), 7.70 – 7.62 (m, 2H, **H**-C_{Ar}-*m*-SO_2NH- and **H**-C_{Ar}-*m*-NMe_2), 7.58 – 7.54 (m, 2H, C_{Ar}-**H**), 7.53 – 7.48 (m, 1H, **H**-C_{Ar}-*o*-NMe2), 7.48 – 7.44 (m, 2H, C_{Ar}-**H**), 7.21 – 7.16 (m, 2H, C_{Ar}-**H**), 4.40 (t, J = 5.2 Hz, 2H, -O-C**H**$_2$-CONH-), 4.12 (s, 2H, -S-C**H**$_2$-C_{Ar}), 4.07 (d, J = 5.6 Hz, 2H, -SO_2NH-C**H**$_2$-), 4.03 (s, 2H, -C**H**$_2$-O-), 3.79 (s, 2H, -C**H**$_2$-O-), 3.74 (t, J = 5.3 Hz, 2H, $N_{Triazol}$-CH_2-C**H**$_2$-O-), 3.63 – 3.56 (m, 4H, C_{Ar}-C**H**$_2$-C(=NOSO$_3$K)-S- and -C**H**$_2$-$N_{Triazol}$), 2.97 (s, 6H, -N(C**H**$_3$)$_2$). **^{13}C-NMR** (126 MHz, DMSO-d_6) δ [ppm]: 168.1 (-**C**ONH-), 158.2 (-**C**(=N-OSO$_3$K)-S-), 154.3 (**C**$_{Ar}$-NMe_2), 146.5 (**C**$_{Ar}$-NO_2), 145.0 (-S-CH_2-**C**$_{Ar}$), 143.4 ($C_{Ar-Triazol}$), 137.2 (-CONH-C_{Ar}), 136.3 (**C**$_{Ar}$-SO_2NH-), 133.0 (**C**$_{Ar}$-CH_2-C(=N-OSO$_3$K)-S-), 130.9 (**C**$_{Ar}$), 129.9 (**C**$_{Ar}$-H), 128.9 (**C**$_{Ar}$-H), 128.6 (**C**$_{Ar}$-H), 128.4 (**C**$_{Ar}$-H), 127.7 (**C**$_{Ar}$-H), 124.4 (**C**$_{Ar}$-H), 123.6 (**C**$_{Ar}$-H), 123.6 (**C**$_{Ar-Triazol}$-H), 121.3 (**C**$_{Ar}$), 119.8 (**C**$_{Ar}$), 70.2 (-CH_2-O-), 70.1 (-**C**H$_2$-O-), 69.4 (-**C**H$_2$-O-), 68.9 ($N_{Triazol}$-CH_2-**C**H$_2$-O-), 49.2 ($N_{Triazol}$-**C**H$_2$-CH_2-O-), 45.6 (-SO_2NH-**C**H$_2$-), 37.9 (C_{Ar}-**C**H$_2$-C(=NOSO$_3$K)-S-), 37.4 (-S-**C**H$_2$-C_{Ar}), 32.5 (-N(**C**H$_3$)$_2$). **Fluorescence emission:** (H_2O, c = 43 µg/10 mL) λ_{Ex} = 365 nm, λ_{Em} = 509 nm.

Hex-5-ynal oxime (31)

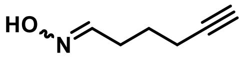

Chemical Formula: C_6H_9NO
Molecular Weight: 111,1440

Hex-5-yn-1-ol (**30**) (1.50 g, 15.28 mmol, 1.0 equiv) was added dropwise to a solution of IBX (6.42 g, 22.93 mmol, 1.5 equiv) in anhydrous DMSO (38 mL, 0.402 M). The resulting solution was stirred for 2.5 h at 22°C. Dichloromethane (50 mL) was then added to the reaction mixture, and stirring was continued for 20 min at 22°C (until a white precipitate was formed). The resulting precipitate was filtered off and extracted with dichloromethane (5×10 mL). The combined filtrates were washed with brine (5×20 mL), dried over anhydrous Na_2SO_4, and filtered. The solvent was evaporated under reduced pressure (Caution: The heating bath was maintained at approximately 22°C, to avoid product evaporation) yielding hex-5-ynal (882 mg, 9.18 mmol, 60%) as a yellow oil that was used for the next step without further purification.

TLC (*n*-pentanes:Et_2O/10:1), R_f: 0.40 [CAM]. **^{1}H-NMR** (300 MHz, $CDCl_3$) δ [ppm]: 9.79 (t, *J* = 1.3 Hz, 1H, -C**H**O), 2.59 (td, *J* = 7.2, 1.3 Hz, 2H, -C**H**$_2$-CHO), 2.25 (td, *J* = 6.9, 2.7 Hz, 2H, -C**H**$_2$-CH_2-CHO), 1.97 (t, *J* = 2.6 Hz, 1H, -CH_2-C≡C-**H**), 1.89–1.75 (m, 2H, -C**H**$_2$-C≡C-H). **^{13}C-NMR** (75 MHz, $CDCl_3$) δ [ppm]: 201.77 (-**C**HO), 83.13 (-CH_2-**C**≡C-H), 69.33 (-CH_2-C≡**C**-H), 42.47 (-CH_2-**C**H$_2$-CHO), 20.75 (-**C**H$_2$-CH_2-CHO), 17.72 (-**C**H$_2$-C≡C-H).

Sodium acetate (744 mg, 9.07 mmol, 1.05 equiv) and hydroxylamine hydrochloride (630 mg, 9.07 mmol, 1.05 equiv) were added sequivuentially at 23°C to a solution of hex-5-ynal (830 mg, 8.634 mmol, 1.0 equiv) in anhydrous acetonitrile (19.2 mL, 0.45 M) and anhydrous methanol (9.6 mL, 0.9 M) under argon. The resulting mixture was then stirred, and the progress of the reaction was monitored by TLC. Upon completion of the reaction after 10 h, water (10 mL) was added, and the mixture was extracted with dichloromethane (4×25 mL). The organic phases were combined, washed with brine (4×20 mL), dried over anhydrous Na_2SO_4, filtered, and concentrated by a rotary evaporator. The resulting crude product was then purified by flash column chromatography on silica gel using (Hex:EtOAc/100:0 → 75:25) to yield hex-5-ynal oxime (**31**) (719 mg, 6.46 mmol, 75%) in a 1:1 ratio of E and Z-isomers as a white amorphous solid.

TLC (hexane:EtOAc/3:1), R_f: 0.33 [UV^{254}, CAM]. **^{1}H-NMR** (600 MHz, $CDCl_3$) δ [ppm]: 7.89 (s, 2H, -CH=NO**H** of (E)- and (Z)-isomer), 7.44 (t, *J* = 5.9 Hz, 1H, -CH=NO**H** of (E)-isomer), 6.87 (s, 1H, -CH=NO**H** of (Z)-isomer), 2.57 – 2.47 (m, 2H, -CH_2-C**H**$_2$-CH=NOH of (E)-isomer), 2.34 (q, *J* = 6.9 Hz, 2H, -CH_2-C**H**$_2$-CH=NOH of (Z)-isomer), 2.30 – 2.19 (m, 4H of -C**H**$_2$-CH_2-CH=NOH of (E)- and (Z)-isomer), 2.07 – 1.95 (m, 2H, -CH_2-C≡C-**H** of (E)- and (Z)-isomer), 1.81 – 1.68 (m, 4H, -C**H**$_2$-C≡C-H of (E)- and (Z)-isomer). **^{13}C-NMR** (151 MHz, $CDCl_3$) δ [ppm]: 152.1 (-**C**H=NOH of (E)-isomer), 151.3 (-**C**H=NOH of (Z)-isomer), 83.4 (-CH_2-**C**≡C-H of (E)-isomer), 83.3 (-CH_2-**C**≡C-H of (Z)-isomer), 69.2 (-CH_2-C≡**C**-H of (E)-isomer), 69.2 (-CH_2-C≡**C**-H of (Z)-isomer), 29.7 (-CH_2-**C**H$_2$-CH=NOH of (E)-isomer), 28.4 (-CH_2-**C**H$_2$-CH=NOH of (Z)-isomer), 25.26 (-**C**H$_2$-CH_2-CH=NOH of (E)-isomer), 24.8 (-**C**H$_2$-CH_2-CH=NOH of (Z)-isomer), 18.3 (-**C**H$_2$-C≡C-H of (E)-isomer), 17.9 (-**C**H$_2$-C≡C-H of (Z)-isomer). The analytic data were in accordance with prior published data.[10]

4-Nitrobenzyl (*Z*)-*N*-hydroxyhex-5-ynimidothioate (32)

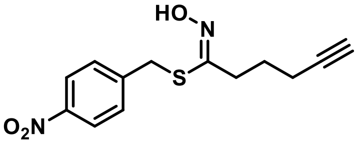

Chemical Formula: $C_{13}H_{14}N_2O_3S$
Molecular Weight: 278,3260

N-Chlorosuccinimide (198 mg, 1.49 mmol, 1.1 equiv) was added in 5 portions to a solution of the mixture of oximes (150 mg, 1.3496 mmol, 1.0 equiv) in anhydrous DMF (1 mL, 1.3496 M) under argon atmosphere and at 0°C. The reaction mixture was warmed up to 23°C and stirred under light exclusion for 2 h. A solution of (4-nitrophenyl)methanethiol (**31**) (343 mg, 2.02 mmol, 1.5 equiv) and DiPEA (349 mg, 0.5 mL, 2.70 mmol, 2.0 equiv) in anhydrous Et_2O (1 mL, 2.0245 M) was added dropwise to the precooled reaction mixture at 0°C and under argon atmosphere. The resulting solution was warmed to 23°C and stirring continued for 10 h, before dilution with EtOAc (10 mL) and H_2O (10 mL). The resulting mixture was then extracted with EtOAc (3×10 mL). The combined organic layers were washed with brine (3×10 mL), dried over anhydrous Na_2SO_4, filtered and the solvent was removed under reduced pressure. The resulting residue was purified by flash column chromatography (CH_2Cl_2:MeOH/100:0 → 95:5) to afford 4-nitrobenzyl (Z)-*N*-hydroxyhex-5-ynimidothioate (**32**) (334 mg, 1.20 mmol, 89%) as a white amorphous solid.

TLC (CH_2Cl_2), R_f: 0.20 [UV^{254}, CAM]. **IR** (ATR) [cm^{-1}]: 3286, 2941, 2857, 1708, 1600, 1518, 1425, 1344, 1245, 1183, 1114, 945, 857, 808, 717, 644. **HRMS** (ESI) [m/z]: 279.07983, calculated 279.07979 for $[C_{13}H_{15}N_2O_3S]^+$, 301.06173, calculated 301.06177 for $[C_{13}H_{14}N_2NaO_3S]^+$. **^{1}H-NMR** (600 MHz, CD_3OD) δ [ppm]: 8.20 (d, *J* = 8.8 Hz, 2H, C_{Ar}-**H**), 7.64 (d, *J* = 8.8 Hz, 2H, C_{Ar}-**H**), 4.30 (s, 2H, C_{Ar}-C**H**$_2$-S-), 2.49–2.46 (m, 2H, -S-(HON=)CH-C**H**$_2$-), 2.23 (t, *J* = 2.6 Hz, 1H, -CH_2-C≡C-**H**) , 2.17 (td, *J* = 7.0, 2.6 Hz, 2H, -S-(HON=)CH-CH_2-C**H**$_2$-), 1.77–1.68 (m, 2H, -C**H**$_2$-C≡C-H). **^{13}C-NMR** (151 MHz, CD_3OD) δ [ppm]: 152.9 (-**C**(=NOH)-S-), 148.5 (**C**$_{Ar}$-NO_2), 147.2 (**C**$_{Ar}$-CH_2-), 131.0 (**C**$_{Ar}$-H), 124.7 (**C**$_{Ar}$-H), 84.4 (-CH_2-**C**≡C-H), 70.2 (-CH_2-C≡**C**-H), 34.2 (C_{Ar}-**C**H_2-S-), 33.0 (-**C**H_2-CH_2-CH_2-C≡C-H), 27.6 (-**C**H_2-CH_2-C≡C-H), 18.3 (-**C**H_2-C≡C-H).

Potassium (*Z*)-(1-((4-nitrobenzyl)thio)hex-5-yn-1-ylidene)amino sulfate (*ps*GSL(NO_2)-alkyne)

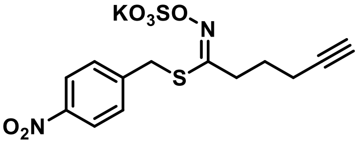

Chemical Formula: $C_{13}H_{13}KN_2O_6S_2$
Molecular Weight: 396,4733

Sulfur trioxide pyridine complex (358 mg, 2.2476 mmol, 5.0 equiv) and anhydrous pyridine (355 mg, 0.36 mL, 4.4950 mmol, 10.0 equiv) were added to a solution of 4-nitrobenzyl (*E*)-*N*-hydroxyhex-5-ynimidothioate (**32**) (125 mg, 0.4495 mmol, 1.0 equiv) in anhydrous CH_2Cl_2 (1 mL, 0.4495 M) and the reaction mixture was stirred for 4 h at 60°C. After cooling down the reaction mixture to 23°C, an aqueous solution of $KHCO_3$ (0.61 mL, 2.0 M, 20.0 equiv) was added and the mixture was stirred vigorously for an additional 30 min at 23°C. The solvents were removed under reduced pressure and the resulting residue was purified by flash column chromatography (CH_2Cl_2:MeOH/100:0 → 90:10 → 80:20) to afford ***ps*GSL(NO_2)-alkyne** (123 mg, 0.3102 mmol, 69%) as a yellowish amorphous solid.

TLC (CH_2Cl_2:MeOH/ 80:20), R_f: 0.27 [UV^{254}, CAM]. **IR** (ATR) [cm^{-1}]: 3452, 3391, 3279, 3112, 2935, 2860, 2065, 1719, 1663, 1592, 1518, 1408, 1348, 1288, 1243, 1118, 1071, 1007, 916, 849, 807, 718, 645, 585. **LRMS** (ESI) [m/z]: 357.2 for $[C_{13}H_{13}N_2O_6S_2]^-$. **HRMS** (ESI) [m/z]: 403.00063, calculated 403.00049 for $[C_{13}H_{13}N_2Na_2O_6S_2]^+$. **^{1}H-NMR** (600 MHz, CD_3OD) δ [ppm]: 8.20 (d, *J* = 8.9 Hz, 2H, C_{Ar}-**H**), 7.67 (d, *J* = 8.9 Hz, 2H, C_{Ar}-**H**), 4.36 (s, 2H, C_{Ar}-C**H**$_2$-S-), 2.67–2.58 (m, 2H, -S-(KO_3SON=)CH-C**H**$_2$-), 2.27–2.21 (m, 3H, -CH_2-C≡C-**H** and -S-(KO_3SON=)CH-CH_2-C**H**$_2$-), 1.87–1.76 (m, 2H, -C**H**$_2$-C≡C-H). **^{13}C-NMR** (151 MHz, CD_3OD) δ [ppm]: 160.9 (-**C**(=$NOSO_3K$)-S-), 148.6 (**C**$_{Ar}$-NO_2), 146.2 (**C**$_{Ar}$-CH_2-), 131.1 (**C**$_{Ar}$-H), 124.8 (**C**$_{Ar}$-H), 84.3 (-CH_2-**C**≡C-H), 70.4 (-CH_2-C≡**C**-H), 34.7 (C_{Ar}-**C**H_2-S-), 33.1 (-**C**H_2-CH_2-CH_2-C≡C-H), 27.3 (-**C**H_2-CH_2-C≡C-H), 18.3 (-**C**H_2-C≡C-H).

***N*-(2-(2-(2-(2-azidoethoxy)ethoxy)ethoxy)ethyl)-3-(5,5-difluoro-7,9-dimethyl-5*H*-5λ^4,6λ^4-dipyrrolo[1,2-*c*:2',1'-*f*][1,3,2]diazaborinin-3-yl)propenamide (33)**

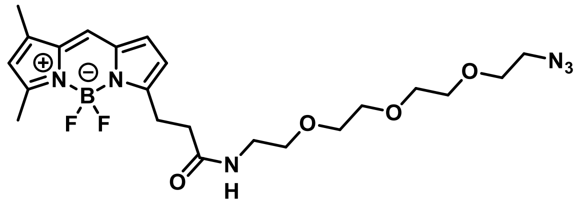

Chemical Formula: $C_{22}H_{31}BF_2N_6O_4$
Molecular Weight: 492,3348

$BODIPY_{FL}$ NHS ester(10.0 mg, 25.70 µmol, 1.0 equiv) was placed in an aluminum foil-wrapped 4 mL vial, and dissolved with dry CH_2Cl_2 (0.2 mL). To the resulting solution was added a solution of azido-PEG_3-amine (16.8 mg, 77.09 µmol, 3.0 equiv) and DiPEA (10.0 mg, 13.4 µL, 77.09 µmol, 3.0 equiv) in dry CH_2Cl_2 (0.5 mL, 0.05 M) at 0°C. Protected from light, the reaction mixture was warmed up to 23°C, and stirring was maintained for 16 h (the progress of the reaction was monitored by TLC). The reaction mixture was purified by preparative thin layer chromatography using CH_2Cl_2:MeOH/20:1 to yield compound **33** (12.5 mg, 25.39 µmol, 99%) as a dark orange amorphous solid.

TLC (CH_2Cl_2:MeOH/20:1), R_f = 0.5 [$UV^{254, 366}$, Vis (orange)]. **^{1}H-NMR** (600 MHz, $CDCl_3$) δ [ppm]: 7.07 (s, 1H, $C_{Ar\text{-}BODIPY\text{-}meso}$-**H**), 6.87 (d, *J* = 4.0 Hz, 1H, $C_{Ar\text{-}BODIPY}$-**H**), 6.29 (d, *J* = 4.1 Hz, 1H, $C_{Ar\text{-}BODIPY}$-**H**), 6.19 (s, 1H, -CON**H**-), 6.10 (s, 1H, $C_{Ar\text{-}BODIPY}$-**H**), 3.64 (d, *J* = 4.9 Hz, 2H, -O-C**H**$_2$-CH_2-NH-), 3.63 (s, 4H, -O-C**H**$_2$-C**H**$_2$-O-), 3.60 (dd, *J* = 5.7, 2.6 Hz, 2H, -O-C**H**$_2$-CH_2-O-), 3.57 (dd, *J* = 5.7, 2.6 Hz, 2H, -O-CH_2-C**H**$_2$-O-), 3.50 (t, *J* = 5.2 Hz, 2H, -O-C**H**$_2$-CH_2-N_3), 3.42 (q, *J* = 5.2 Hz, 2H, -O-CH_2-C**H**$_2$-N_3), 3.34 (t, *J* = 5.1 Hz, 2H, -O-CH_2-C**H**$_2$-NH-), 3.28 (t, *J* = 7.5 Hz, 2H, $C_{Ar\text{-}BODIPY}$-CH_2-C**H**$_2$-CONH-), 2.63 (t, *J* = 7.5 Hz, 2H, $C_{Ar\text{-}BODIPY}$-C**H**$_2$-CH_2-CONH-), 2.55 (s, 3H, -C**H**$_3$), 2.24 (s, 3H, -C**H**$_3$). **^{13}C-NMR** (151 MHz, $CDCl_3$) δ [ppm]: 171.8 (-**C**ONH-), 160.0 (**C**$_{Ar\text{-}BODIPY}$), 157.6 (**C**$_{Ar\text{-}BODIPY}$), 143.7 (**C**$_{Ar\text{-}BODIPY}$), 135.0 (**C**$_{Ar\text{-}BODIPY}$), 133.3 (**C**$_{Ar\text{-}BODIPY}$), 128.2 (**C**$_{Ar\text{-}BODIPY}$-H), 123.7 (**C**$_{Ar\text{-}BODIPY}$-H), 120.3 (**C**$_{Ar\text{-}BODIPY}$-H), 117.5 (**C**$_{Ar\text{-}BODIPY}$-H), 70.6 (-O-**C**H$_2$-CH_2-O-), 70.5 (-O-**C**H$_2$-CH_2-O-), 70.2 (-O-**C**H$_2$-CH_2-O-), 70.0 (-CONH-CH_2-**C**H$_2$-O-), 69.7 (-O-**C**H$_2$-CH_2-N_3), 50.6 (-O-CH_2-**C**H$_2$-N_3), 39.3 (-CONH-**C**H$_2$-CH_2-O-), 35.8 ($C_{Ar\text{-}BODIPY}$-CH_2-**C**H$_2$-CONH-), 24.8 ($C_{Ar\text{-}BODIPY}$-**C**H$_2$-CH_2-CONH-), 14.9 (-**C**H$_3$), 11.3 (-**C**H$_3$). The analytic data were in accordance with prior published data.[11]

Potassium (*Z*)-(4-(1-(15-(5,5-difluoro-7,9-dimethyl-5*H*-5λ^4,6λ^4-dipyrrolo[1,2-*c*:2',1'-*f*][1,3,2]diazaborinin-3-yl)-13-oxo-3,6,9-trioxa-12-azapentadecyl)-1*H*-1,2,3-triazol-4-yl)-1-((4-nitrobenzyl)thio)butylidene)amino sulfate (*ps*GSL$_{PEG}$(NO_2)-BODIPY$_{FL}$)

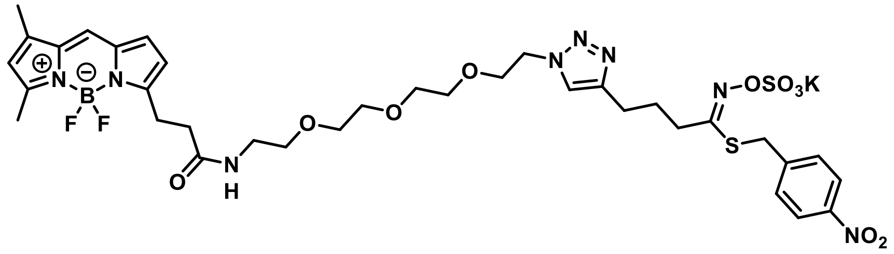

Chemical Formula: $C_{35}H_{44}BF_2KN_8O_{10}S_2$
Molecular Weight: 888,8081

Compound **33** (12.0 mg, 24.37 µmol, 1.0 equiv) and ***ps*GSL(NO_2)-alkyne** (9.7 mg, 24.37 µmol, 1.0 equiv) were dissolved in DMF (500 µL, 0.05 M). TBTA (2.6 mg, 4.87 µmol, 0.2 equiv) and a suspension of NaAsc (1.9 mg, 9.75 µmol, 0.4 equiv) and $CuSO_4$·5 H_2O (1.2 mg, 4.87 mol, 0.2 equiv) in H_2O (100 µL, 0.05 M) were added subsequently to the reaction mixture, and stirring was maintained for 15 h at 23°C under light exclusion. After completion of the reaction was indicated by LCMS and TLC, the reaction mixture was co-evaporated with toluene (3×800µL) under reduced pressure and light exclusion. The resulting crude product was diluted with CH_2Cl_2 (0.3 mL) and purified by preparative thin-layer chromatography using CH_2Cl_2:MeOH:/20:1 to afford ***ps*GSL$_{PEG}$(NO_2)-BODIPY$_{FL}$** (13.9 mg, 15.67 µmol, 65%) as an orange amorphous solid.

TLC (CH_2Cl_2:MeOH/20:1), R_f = 0.3 [$UV^{254, 366}$, Vis (orange)]. **IR** (ATR) [cm^{-1}]: 3323, 2952, 2924, 2855, 1662, 1604, 1521, 1488, 1441, 1346, 1248, 1175, 1135, 1085, 1060, 972, 898, 858, 795, 670, 639, 573, 483. **LRMS** (ESI) [m/z]: 849.41 for [$C_{35}H_{44}BF_2N_8O_{10}S_2$]$^-$. **HRMS** (ESI) [m/z]: calculated for [$C_{35}H_{44}BF_2N_8O_{10}S_2$]$^-$, 849.26884; found 849.26807. **^{1}H-NMR** (600 MHz, CD_3OD) δ [ppm]: 8.15 (d, *J* = 8.7 Hz, 2H, C_{Ar}-**H**), 7.81 (s, 1H, $C_{Ar\text{-}Triazol}$-**H**), 7.57 (d, *J* = 8.8 Hz, 2H, C_{Ar}-**H**), 7.41 (s, 1H, $C_{Ar\text{-}BODIPY\text{-}meso}$-**H**), 7.00 (d, *J* = 4.0 Hz, 1H, $C_{Ar\text{-}BODIPY}$-**H**), 6.33 (d, *J* = 4.0 Hz, 1H, $C_{Ar\text{-}BODIPY}$-**H**), 6.19 (s, 1H, $C_{Ar\text{-}BODIPY}$-**H**), 4.50 (t, *J* = 5.1 Hz, 2H, -O-CH_2-C**H**$_2$-$N_{Triazol}$), 4.25 (s, 2H, C_{Ar}-C**H**$_2$-S-), 3.84 (t, *J* = 5.1 Hz, 2H, -O-C**H**$_2$-CH_2-$N_{Triazol}$), 3.57 (s, 4H, -O-C**H**$_2$-C**H**$_2$-O-), 3.55 (s, 4H, -O-C**H**$_2$-C**H**$_2$-O-), 3.52 (t, *J* = 5.5 Hz, 2H, -O-C**H**$_2$-CH_2-NH-), 3.36 (t, *J* = 5.5 Hz, 2H, -O-CH_2-C**H**$_2$-NH-), 3.22 (t, *J* = 7.7 Hz, 2H, $C_{Ar\text{-}BODIPY}$-C**H**$_2$-CH_2-CONH-), 2.71 (t, *J* = 7.2 Hz, 2H, -S-(KO_3SON=)CH-CH_2-CH_2-C**H**$_2$-$C_{Ar\text{-}Triazol}$), 2.62 (d, *J* = 7.6 Hz, 2H, $C_{Ar\text{-}BODIPY}$-C**H**$_2$-

CH_2-CONH-), 2.49 (s, 3H, -C**H**$_3$), 2.26 (s, 3H, -C**H**$_3$), 1.95 (p, *J* = 7.3 Hz, 2H, -S-(KO_3SON=)CH-C**H**$_2$-CH_2-CH_2-$C_{Ar-Triazol}$), 1.36 – 1.28 (m, 2H, -S-(KO_3SON=)CH-CH_2-C**H**$_2$-CH_2-$C_{Ar-Triazol}$). **^{13}C-NMR** (151 MHz, CD_3OD) δ [ppm]: 174.7 (-**C**ONH-), 161.2 (-**C**(=$NOSO_3K$)-S-), 160.4 (**C**$_{Ar-BODIPY}$), 158.6 (**C**$_{Ar-BODIPY}$), 148.6 (**C**$_{Ar}$-NO_2), 148.0 (**C**$_{Ar-Triazol}$-H), 146.3 (**C**$_{Ar}$-CH_2-), 145.8 (**C**$_{Ar-BODIPY}$), 136.5 (**C**$_{Ar-BODIPY}$), 134.9 (**C**$_{Ar-BODIPY}$), 131.1 (**C**$_{Ar}$-H), 129.8 (**C**$_{Ar-BODIPY}$-H), 125.9 (**C**$_{Ar-BODIPY}$-H), 124.8 (**C**$_{Ar}$-H and **C**$_{Ar-Triazol}$), 121.3 (**C**$_{Ar-BODIPY}$-H), 117.8 (**C**$_{Ar-BODIPY}$-H), 71.5 (-O-**C**H$_2$-CH_2-O-), 71.4 (-O-**C**H$_2$-CH_2-O-), 71.3 (-O-**C**H$_2$-CH_2-O-), 70.6 (-O-**C**H$_2$-CH_2-O-), 70.3 (-CONH-CH_2-**C**H$_2$-O-), 51.2 (-O-CH_2-**C**H$_2$-$N_{Triazol}$), 40.4 (-CONH-**C**H$_2$-CH_2-O-), 35.8 ($C_{Ar-BODIPY}$-CH_2-C**H**$_2$-CONH-), 34.6 (C_{Ar}-**C**H$_2$-S-), 33.3 ($C_{Ar-Triazol}$-**C**H$_2$-CH_2-CH_2-C(=$NOSO_3K$)-S-), 27.8 ($C_{Ar-Triazol}$-CH_2-CH_2-**C**H$_2$-C(=$NOSO_3K$)-S-), 25.6 ($C_{Ar-Triazol}$-CH_2-**C**H$_2$-CH_2-C(=$NOSO_3K$)-S-), 25.1 ($C_{Ar-BODIPY}$-**C**H$_2$-CH_2-CONH-), 14.9 (-**C**H$_3$), 11.3 (-**C**H$_3$).

Synthesis of model peptide 1-5

Loading of (((9*H*-fluoren-9-yl)methoxy)carbonyl)lysine onto the resin: In a peptide synthesis vessel, 1.0 g of 2-chlorotrityl resin (loading capacity 1.15 mmol/g) were swollen in CH_2Cl_2 (1 x 15 min). The solvent was removed through filter of peptide synthesis vessel, and a suspension of Fmoc-Lys(Boc)-OH (1.08 mg, 2.0 equiv) in DiPEA (0.8 mL, 4.0 equiv) and CH_2Cl_2 (3.0 mL) was added to the resin, the reaction vessel flushed with CH_2Cl_2 (2.0 mL) and added to the resin. The reaction mixture was agitated for 12 h at 23^{0}C, after which the solution was removed. The resin was washed with CH_2Cl_2 (1 x 10.0 mL), DMF (3 x 10.0 mL) and CH_2Cl_2 (3 x 10.0 mL), then dried under reduced pressure for 2 h.

The loading of Fmoc-protected amino-acid onto the resin was determined by spectrophotometric quantification at λ=301 nm of the dibenzofulvene liberated after deprotection of the Fmoc group with piperidine:DMF/(1:4). To a precisely weighed sample of resin (1.21 mg), a solution of piperidine:DMF/(1:4, 1.0 mL) was added. The mixture was agitated for 20 min, after which 100 µL of the reaction mixture was transferred to a 20.0 mL flask, filled with 9.90 mL DMF. Absorption of the resulting solution was determined at λ=301 nm against a control of a solution of 100 µL piperidine:DMF/(1:4) diluted with DMF to a volume of 10.0 mL. Loading grades were calculated in mmol/g = [100 × absorbance] ÷ [7.8 × resin mass (mg)]. Measurements were carried out three times and the loading was determined as L=0.90 mmol/g (90% loading). The following procedures are performed using a Multisyntech Syro II Peptide Synthesizer by Biotage.

General procedure for preparation of a resin-bound peptide using automated SPPS: The Fmoc-deprotection of the previous coupling was achieved by adding a piperidine:DMF solution (2:3) to the resin, reacting it for 20 min and removing the reaction-mixture. The procedure is repeated with piperidine:DMF (1:4) before washing the resin with DMF (6x). A solution of Fmoc-protected amino acid (3.0 equiv) in DMF, HATU (3.0 equiv) in DMF, HOAt (3.0 equiv) in DMF and DiPEA (6.0 equiv) in NMP were added to the resin. The reaction mixture was incubated for 40 min at 23^{0}C, after which the solution was removed. The coupling was repeated as described above. The resin was washed with DMF (3x).

Preparation of acetylation of peptide and cleavage from resin: The Fmoc-deprotection of the previous coupling was achieved by adding a piperidine:DMF solution (2:3) to the resin, reacting it for 20 min and removing the reaction-mixture. The procedure is repeated with piperidine:DMF (1:4) before washing the resin with DMF (6x). For **Model peptide 2,4** and **5**, a solution of Ac_2O/Pyridine (20 equiv) in DMF was added to the resin. The reaction mixture was incubated for 30 min at 23^{0}C, after which the solution was removed. The acetylation was repeated as described above. The resin was washed with DMF (3x). The peptide loaded resin was gently shaken with a mixture of TFA/TIPS:H_2O (92.5:5:2.5, 2 mL) for 1 h at 23°C.

General procedure for purifiication of peptides: The solution was evaporated by an air stream until the volume was <500 µL and 4°C cold Et_2O (10 mL) was added to crash out the product. After centrifugation at 7800 rpm for 10 min at 4°C. The solution was removed and the residue was dissolved in 500 µL of H_2O, filtered through a CHROMAFIL® 45 µm filter and purified by preparative HPLC (Hypersil GOLD C18 RP-column, 5 µm, 250 mm×10.0 mm (4.5 mL/min), UV=220 nm, Eluents: H_2O (0.1% TFA):CH_3CN (0.1% TFA)/100:0 → 40:60 in 47 min). Product containing fractions were diluted with H_2O, frozen with liquid N_2 at -196°C and lyophilized yielding the peptides as white powder.

_L_-asparaginyl-_L_-alanyl-_L_-cysteinylglycyl-_L_-lysyl-_L_-asparaginyl-_L_-alanylglycyl-_L_-lysine (Model peptide 1)

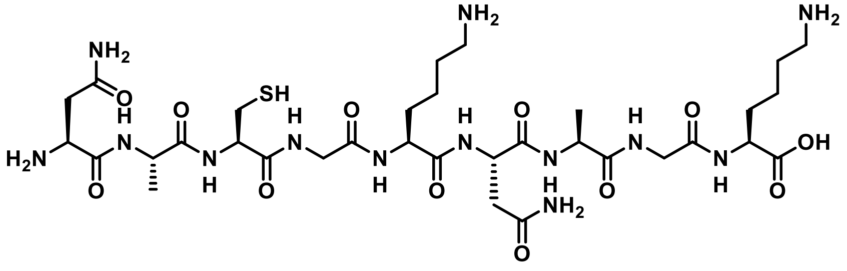

Chemical Formula: $C_{33}H_{59}N_{13}O_{12}S$
Molecular Weight: 861,9740

HRMS (ESI) [m/z]: 862.41840, calculated 862.41996 for $[C_{33}H_{59}N_{13}O_{12}S+H]^+$, err [ppm] 1.81. **^{1}H-NMR** (800 MHz, MeOD-d4) δ [ppm]: 4.67 (dd, *J* = 7.8, 5.5 Hz, 1H), 4.44 (dd, *J* = 9.8, 4.5 Hz, 1H), 4.37 (dd, *J* = 8.3, 5.3 Hz, 1H), 4.34 (t, *J* = 7.3 Hz, 1H), 4.29 (dd, *J* = 8.9, 5.4 Hz, 1H), 4.27 – 4.22 (m, 2H), 3.98 – 3.91 (m, 2H), 3.84 (dd, *J* = 16.7, 8.0 Hz, 2H), 3.06 (dd, *J* = 17.0, 6.3 Hz, 1H), 3.02 – 2.97 (m, 2H), 2.94 (t, *J* = 7.6 Hz, 4H), 2.93 – 2.89 (m, 1H), 2.84 (dd, *J* = 15.6, 5.5 Hz, 1H), 2.77 (dd, *J* = 15.6, 7.8 Hz, 1H), 1.97 – 1.91 (m, 1H), 1.88 (ddt, *J* = 14.9, 10.6, 5.9 Hz, 1H), 1.79 (dtd, *J* = 14.2, 9.4, 5.1 Hz, 2H), 1.74 – 1.62 (m, 4H), 1.53 – 1.47 (m, 2H), 1.44 (d, *J* = 7.3 Hz, 4H), 1.42 (d, *J* = 7.2 Hz, 3H). **^{13}C-NMR** (201 MHz, MeOD) δ [ppm]: 175.5, 175.2, 175.0, 174.4, 173.4, 173.3, 173.2, 172.5, 171.7, 170.5, 163.3, 163.1, 163.0, 162.8, 58.2, 55.3, 53.2, 52.0, 51.8, 51.3, 51.1, 44.2, 43.8, 40.6, 40.5, 37.4, 36.1, 31.9, 31.5, 27.9, 27.9, 26.2, 23.7, 23.6, 17.4, 17.4.

acetyl-*L*-asparaginyl-*L*-alanyl-*L*-cysteinylglycyl-*L*-lysyl-*L*-asparaginyl-*L*-alanylglycyl-*L*-lysine (Model peptide 2)

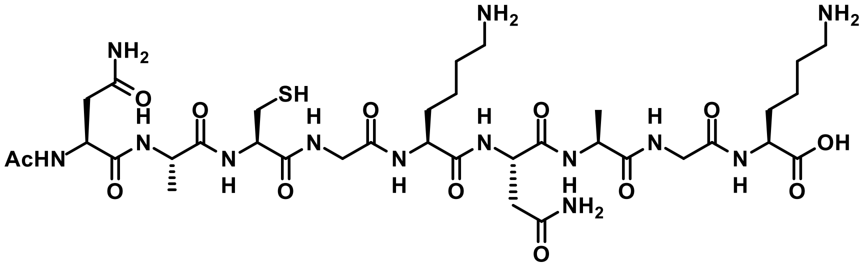

Chemical Formula: $C_{35}H_{61}N_{13}O_{13}S$
Molecular Weight: 904,0110

HRMS (ESI) [m/z]: 904.42865, calculated 904.43053 for $[C_{35}H_{61}N_{13}O_{13}S+H]^+$, err [ppm] 2.08. **^{1}H-NMR** (800 MHz, MeOD-d4) δ [ppm]: 4.69 (dq, *J* = 12.1, 5.5 Hz, 2H), 4.47 (dd, *J* = 10.4, 4.4 Hz, 1H), 4.30 (dd, *J* = 8.9, 5.0 Hz, 1H), 4.24 (dt, *J* = 14.9, 5.6 Hz, 3H), 3.95 – 3.88 (m, 2H), 3.83 (dd, *J* = 16.7, 12.3 Hz, 2H), 3.01 – 2.90 (m, 6H), 2.87 – 2.71 (m, 4H), 2.00 (s, 3H), 1.98 – 1.91 (m, 1H), 1.91 – 1.85 (m, 1H), 1.85 – 1.77 (m, 2H), 1.70 – 1.63 (m, 4H), 1.53 – 1.45 (m, 4H), 1.43 (t, *J* = 7.3 Hz, 6H). **^{13}C-NMR** (201 MHz, MeOD-d4) δ [ppm]: 176.0, 175.6, 175.0, 174.5, 174.2, 173.9, 173.4, 172.9, 171.8, 53.0, 52.1, 51.7, 51.4, 44.5, 43.9, 40.7, 40.5, 37.9, 37.4, 31.8, 31.3, 28.0, 27.8, 25.9, 23.7, 23.6, 22.5, 17.4, 17.2.

***L*-asparaginyl-*L*-alanylglycylglycyl-*L*-lysyl-*L*-asparaginyl-*L*-alanylglycyl-*L*-lysine (Model peptide 3)**

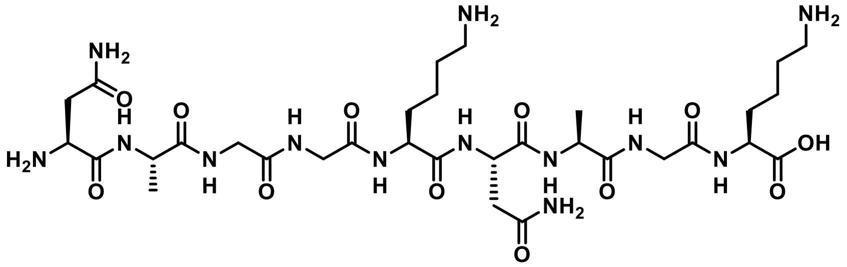

Chemical Formula: $C_{32}H_{57}N_{13}O_{12}$
Molecular Weight: 815,8870

HRMS (ESI) [m/z]: 816.43304, calculated 816.43224 for $[C_{32}H_{57}N_{13}O_{12}+H]^+$, err [ppm] 0.98. **^{1}H-NMR** (800 MHz, MeOD-d4) δ [ppm]: 4.66 (dd, *J* = 7.9, 5.5 Hz, 1H), 4.44 (dd, *J* = 9.8, 4.5 Hz, 1H), 4.34 (q, *J* = 7.1 Hz, 1H), 4.30 – 4.23 (m, 3H), 3.98 – 3.80 (m, 6H), 2.99 – 2.91 (m, 6H), 2.83 (dd, *J* = 15.5, 5.4 Hz, 1H), 2.76 (dd, *J* = 15.5, 7.9 Hz, 1H), 1.94 (dddd, *J* = 13.9, 9.5, 6.8, 4.5 Hz, 1H), 1.91 – 1.84 (m, 1H), 1.80 (dqd, *J* = 14.5, 9.6, 5.1 Hz, 2H), 1.73 – 1.62 (m, 4H), 1.56 – 1.45 (m, 4H), 1.42 (dd, *J* = 7.2, 3.5 Hz, 6H). **^{13}C-NMR** (201 MHz, MeOD-d4) δ [ppm]: 175.5, 175.0, 174.5, 173.4, 173.2, 172.8, 172.7, 171.7, 170.2, 163.1, 162.9, 55.4, 53.2, 52.0, 51.6, 51.2, 51.2, 44.1, 44.0, 43.7 40.63, 40.5, 37.5, 36.2, 31.9, 31.4, 27.9, 27.9, 23.7, 23.7, 17.4, 17.2.

acetyl-*L*-asparaginyl-*L*-alanylglycylglycyl-*L*-lysyl-*L*-asparaginyl-*L*-alanylglycyl-*L*-lysine (Model peptide 4)

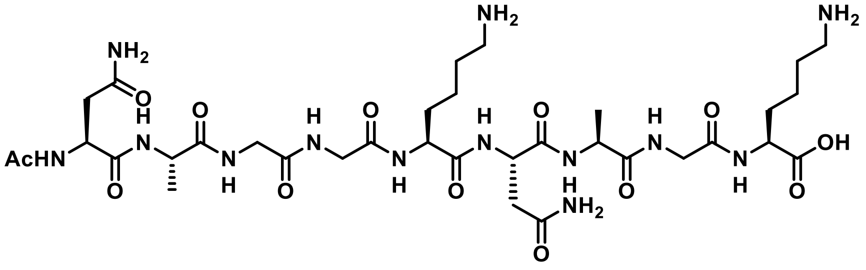

Chemical Formula: $C_{34}H_{59}N_{13}O_{13}$
Molecular Weight: 857,9240

HRMS (ESI) [m/z]: 858.44104, calculated 858.44281 for $[C_{34}H_{59}N_{13}O_{13}+H]^+$, err [ppm] 2.06. **^{1}H-NMR** (800 MHz, MeOD-d4) δ [ppm]: 4.72 (dd, *J* = 7.7, 5.7 Hz, 1H), 4.68 (dd, *J* = 8.1, 5.4 Hz, 1H), 4.45 (dd, *J* = 10.1, 4.3 Hz, 1H), 4.28 – 4.22 (m, 3H), 3.92 (d, *J* = 16.7 Hz, 2H), 3.90 – 3.81 (m, 4H), 2.95 (dt, *J* = 10.9, 6.6 Hz, 4H), 2.86 – 2.74 (m, 3H), 2.69 (dd, *J* = 15.5, 5.6 Hz, 1H), 2.00 (s, 3H), 1.97 – 1.91 (m, 1H), 1.90 – 1.85 (m, 1H), 1.80 (dtd, *J* = 14.3, 9.7, 4.9 Hz, 2H), 1.73 – 1.62 (m, 4H), 1.56 – 1.44 (m, 4H), 1.41 (dd, *J* = 16.3, 7.2 Hz, 6H). **^{13}C-NMR** (201 MHz, MeOD-d4) δ [ppm]: 176.2, 175.6, 175.1, 175.0, 174.9, 174.6, 174.0, 173.4, 173.4,

173.0, 173.0, 171.8, 162.9, 162.8, 55.7, 53.0, 52.1, 51.6, 51.6, 51.3, 44.3, 44.2, 43.8, 40.7, 40.5, 37.9, 37.4, 31.8, 31.3, 27.9, 27.9, 27.8, 23.7, 23.7, 22.6, 17.3, 17.1, 17.1.

acetyl-*L*-asparaginyl-*L*-alanyl-*L*-cysteinylglycylglycyl-*L*-asparaginyl-*L*-alanylglycylglycine (Model peptide 5)

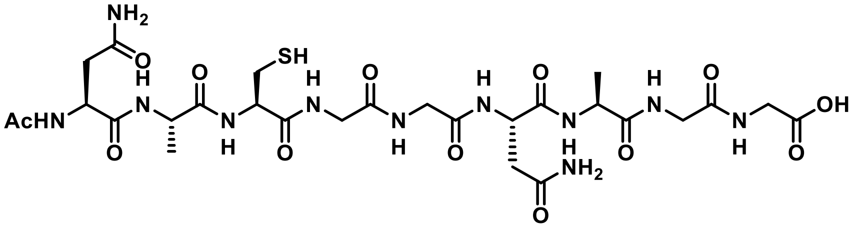

Chemical Formula: $C_{27}H_{43}N_{11}O_{13}S$
Molecular Weight: 761,7650

HRMS (ESI) [m/z]: 762.28351, calculated 762.28353 for $[C_{27}H_{43}N_{11}O_{13}S+H]^+$, err [ppm] 0.026. **^{1}H-NMR** (600 MHz, DMSO-d6) δ [ppm]: 8.25 (d, *J* = 6.8 Hz, 1H), 8.20 – 8.14 (m, 2H), 8.13 (d, *J* = 7.8 Hz, 1H), 8.08 (dd, *J* = 12.2, 7.9 Hz, 2H), 8.04 (t, *J* = 5.8 Hz, 2H), 7.98 (t, *J* = 5.9 Hz, 1H), 7.44 (s, 2H), 6.98 (d, *J* = 12.9 Hz, 2H), 4.54 (dq, *J* = 9.8, 6.9 Hz, 2H), 4.32 (td, *J* = 8.2, 4.8 Hz, 1H), 4.20 (h, *J* = 7.1 Hz, 2H), 3.81 – 3.64 (m, 9H), 2.84 (ddd, *J* = 13.8, 9.3, 4.8 Hz, 1H), 2.75 (dt, *J* = 13.6, 8.1 Hz, 1H), 2.56 (ddd, *J* = 15.4, 11.8, 6.8 Hz, 2H), 2.43 (ddd, *J* = 11.6, 6.8, 2.5 Hz, 3H), 1.83 (s, 3H), 1.24 (t, *J* = 7.2 Hz, 6H). **^{13}C-NMR** (151 MHz, DMSO-d6) δ [ppm]: 172.5, 172.4, 172.0, 171.9, 171.5, 171.2, 170.9, 170.2, 169.4, 169.2, 169.1, 168.7, 55.5, 49.7, 48.9, 48.8, 42.2, 42.0, 40.6, 37.3, 37.2, 25.9, 22.6, 17.6, 17.5.

LC-MS analysis of the purity of *ps*GSL$_{PEG}$(NO$_2$)-N$_3$, *ps*GSL$_{PEG}$(NO$_2$)-BODIPY, *ps*GSL$_{PEG}$(NO$_2$)-DNSA and *ps*GSL$_{PEG}$(NO$_2$)-BODIPY$_{FL}$

***ps*GSL$_{PEG}$(NO$_2$)-N$_3$**, ***ps*GSL$_{PEG}$(NO$_2$)-BODIPY**, ***ps*GSL$_{PEG}$(NO$_2$)-DNSA** and ***ps*GSL$_{PEG}$(NO$_2$)-BODIPY$_{FL}$** were dissolved in DMSO (5 mM) stock solutions. Aliquots were diluted with H_2O:CH_3CN/7:3 + 0.1% TFA and analyzed by LCMS (λ = 254, 360 or 500 nm Eluent: H_2O:CH_3CN + 0.01% FA 10-90% in 4.5 min). HPLC purity was determined as the percentage of the main compound in a sample relative to all detected peaks, calculated as a percentage of the total peak area. All compounds show HPLC-purity ≥96%. Results are summarized in Figure S1.

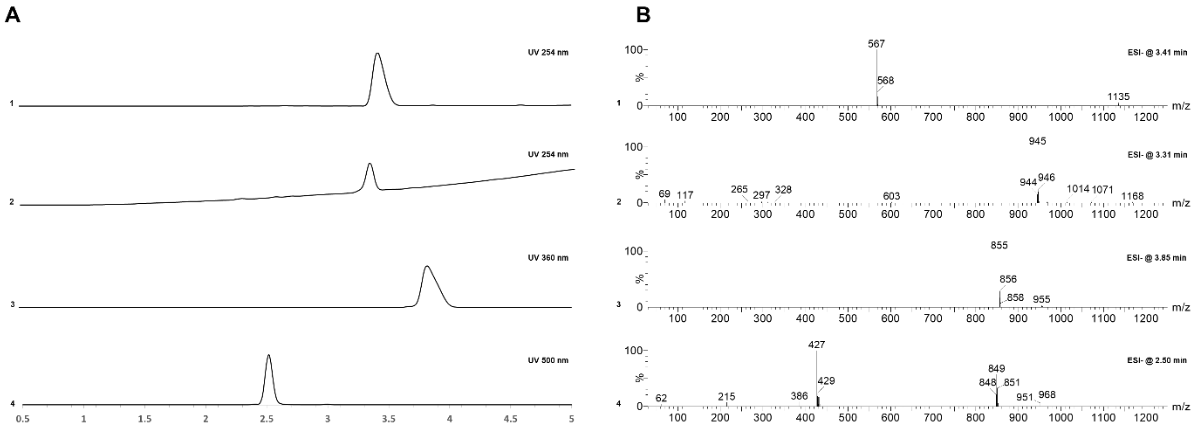

Figure S1: LC-MS analysis of ***ps*GSL$_{PEG}$(NO$_2$)-N$_3$**, ***ps*GSL$_{PEG}$(NO$_2$)-BODIPY**, ***ps*GSL$_{PEG}$(NO$_2$)-DNSA** and ***ps*GSL$_{PEG}$(NO$_2$)-BODIPY$_{FL}$**. **A:** UV chromatogram at of (A1) ***ps*GSL$_{PEG}$(NO$_2$)-N$_3$** at 254 nm. (A2) ***ps*GSL$_{PEG}$(NO$_2$)-BODIPY** at 254 nm. (A3) ***ps*GSL$_{PEG}$(NO$_2$)-DNSA** at 360 nm. (A4) ***ps*GSL$_{PEG}$(NO$_2$)-BODIPY$_{FL}$** at 500 nm. **B:** ESI- mass analysis at (B1) 3.41 min of A1, (B2) 3.31 min of A2, (B3) 3.85 min of A3, (B4) 2.50 min of A4.

LC-MS analysis of the stability of ***ps*****GSL$_{PEG}$(NO$_2$)-N$_3$**

ps**GSL$_{PEG}$(NO$_2$)-N$_3$** were stored in DMSO (5 mM) stock solution at -20°C or TRIS buffer (pH 7.4) containing 10% of DMSO (0.5 mM) at 25°C and analyzed by LCMS (λ = 230 nm, Eluent: H_2O:CH_3CN + 0.01% FA 10-90% in 4.5 min). Results are summarized in Figure S2.

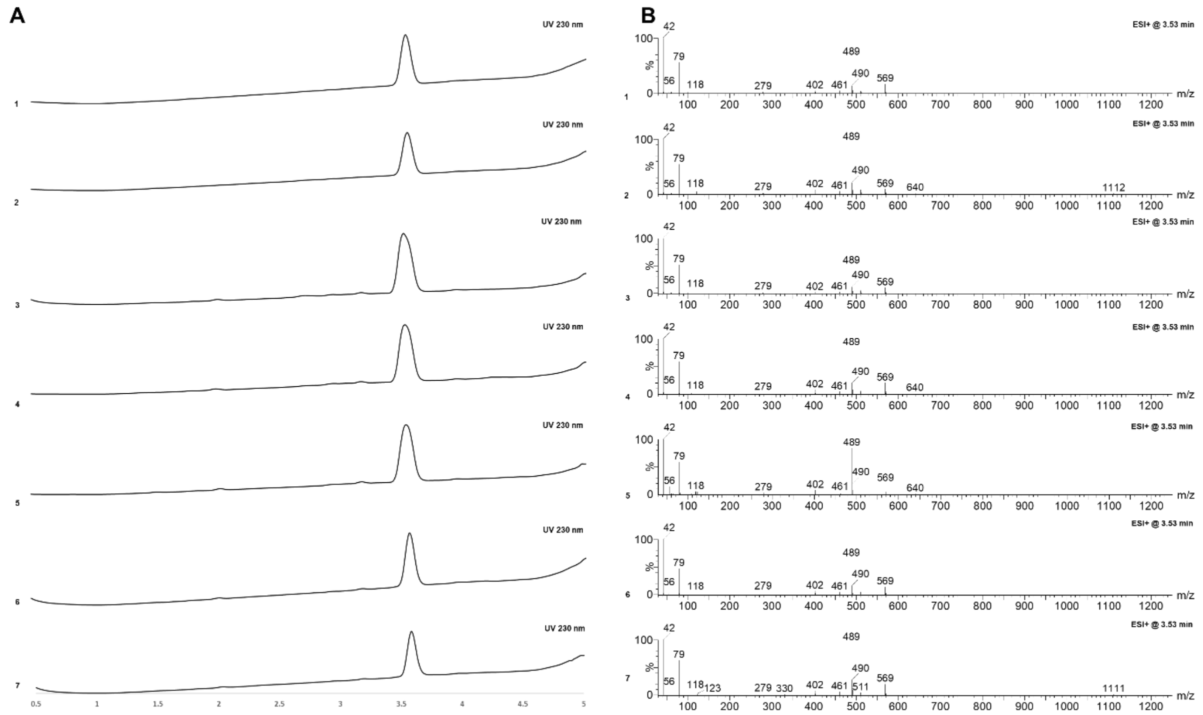

Figure S2. LC-MS analysis of ***ps*****GSL$_{PEG}$(NO$_2$)-N$_3$**. **A:** UV chromatogram at 230 nm of (A1) ***ps*****GSL$_{PEG}$(NO$_2$)-N$_3$** (5 mM) in DMSO stock solution (1 µL injection volume). (A2) ***ps*****GSL$_{PEG}$(NO$_2$)-N$_3$** (5 mM) in DMSO stock solution after stored at -20°C for 1 month (1 µL injection volume). (A3) ***ps*****GSL$_{PEG}$(NO$_2$)-N$_3$** (5 mM) in DMSO stock solution after stored at -20°C for 6 month (1 µL injection volume). (A4) ***ps*****GSL$_{PEG}$(NO$_2$)-N$_3$** (5 mM) in DMSO stock solution after stored at -20°C for 9 month (1 µL injection volume). (A5) ***ps*****GSL$_{PEG}$(NO$_2$)-N$_3$** (0.5 mM) in HEPES buffer (20mM, pH 7.4) with 10% DMSO (10 µL injection volume). (A6) ***ps*****GSL$_{PEG}$(NO$_2$)-N$_3$** (0.5 mM) in HEPES buffer (20mM, pH 7.4) with 10% DMSO after storage for 1 week at 25°C (10 µL injection volume). (A7) ***ps*****GSL$_{PEG}$(NO$_2$)-N$_3$** (0.5 mM) in HEPES buffer (20mM, pH 7.4) with 10% DMSO after storage for 3 weeks at 25°C (10 µL injection volume). **B:** ESI+ mass analysis at (B1) 3.53 min of A1, (B2) 3.53 min of A2, (B3) 3.53 min of A3, (B4) 3.53 min of A4, (B5) 3.53 min of A5, (B6) 3.53 min of A6, and (B7) 3.53 min of A7.

Biochemical and biological evaluation of the compounds

LC-MS analysis of the nitroreductase mediated release of ITCs from *ps*GSLs

Incubation of ***ps*GSL$_{PEG}$(NO$_2$)-N$_3$**, ***ps*GSL$_{PEG}$(NO$_2$)-DNSA** or ***ps*GSL$_{PEG}$(NO$_2$)-BODIPY** were carried out with a substrate concentration of 500 µM with 5 mM NADH, 20 µM FMN, and 10 µM nitroreductase (NfsB) from *E. coli* (Sigma-Aldrich, #N9284) in 20 mM HEPES buffer (Sigma-Aldrich, #H4034, Lot No. SLCD6051) pH 7.4 (total volume 50 µL) at 37°C for 1 or 2 h (Concentrations of stock solutions are given in Table S1). 4°C cold CH_3CN (50 µL) was added to precipitate NfsB and the mixture was centrifuged at 4°C and 7800 x *g* for 2 min twice using Centrifugal Nanosep column with 10K Omega (LOT: FJ4655) to separate the protein precipitate from the supernatant. The filtrate was divided into two LCMS vials and one sample was immediately used to analyze resulting ITCs with LCMS at λ = 230 nm and 254 nm with H_2O:CH_3CN + 0.01% FA 10-90% in 4.5 min. To the second vial, NH_4OH (30%, 10 µL) were added and resulting thiourea were analyzed with LCMS at λ = 230 nm and 254 nm with H_2O:CH_3CN + 0.01% FA 10-90% in 4.5 min.
Control reactions lacking the enzyme or the co-factors were carried out separately for ***ps*GSL$_{PEG}$(NO$_2$)-N$_3$**.
Progression development of NTR reduction over time was investigated using ***ps*GSL$_{PEG}$(NO$_2$)-N$_3$** and analyzing samples after incubation at 37°C at different timepoints (10 min, 20 min, 30 min, 40 min, 50 min, 60 min, 90 min and 120 min).

Table S1. Concentrations of stock solutions for enzymatic assays using nitroreductase (NfsB).

Compound	C_{Stock}	V_{Stock}	C_{Assay}
*ps*GSL(NO$_2$)	5 mM in DMSO	5 µL	500 µM
NADH	10 mM in HEPES buffer 20 mM, pH 7.4	25 µL	5 mM
FMN	2 mM in HEPES buffer 20 mM, pH 7.4	0.5 µL	20 µM
NfsB	41.7 µM in HEPES buffer 20 mM, pH 7.4	12 µL	10 µM
+ 7.5 µL HEPES buffer 20 mM, pH 7.4			

SDS-PAGE of enzymatic conversion and analysis of protein labeling

ps**GSL$_{PEG}$(NO$_2$)-BODIPY** and ***ps*****GSL$_{PEG}$(NO$_2$)-DNSA** were incubated with nitro-reductase (NfsB) from *E. coli* (Sigma-Aldrich, #N9284, Lot No. 0000206122), NADH (Sigma-Aldrich, #N8129, Lot No. SLCB5013) and FMN (Sigma-Aldrich, #F6750, Lot No. MKCJ3258) at a final c_{Assay} of 1 mg/mL at 37°C for 1 or 2 h (see table S2). Samples (5 µL) were treated with SDS-loading buffer (5 µL), heated for 5 min to 95°C, centrifuged for 30 s at 6000 rpm, and 10 µL/sample were loaded onto a 12% acrylamide handcasted or precasted gels (as described above in *General Methods*). Color Prestained Protein Standard, Broad Range (10–250 kDa) (New England Biolabs) was used a protein ladder. The gels were run at 120 V for 40 min and imaged under UV^{366} using a MicroDOC gel imaging system.

Table S2. Concentrations of stock solutions for protein labeling assays analyzed by SDS PAGE.

Compound	C_{Stock}	V_{Stock}	final C_{Assay} in 50 µL
*ps*GSL(NO$_2$)	5 mM in DMSO	5 µL	500 µM
NADH	10 mM in HEPES buffer 20 mM, pH 7.4	25 µL	5 mM
FMN	2 mM in HEPES buffer 20 mM, pH 7.4	0.5 µL	20 µM
NfsB	41.7 µM in HEPES buffer 20 mM, pH 7.4	12 µL	10 µM
+ 7.5 µL HEPES buffer 20 mM, pH 7.4			
+ 0.5 µL of 100µg/µL stock solution of BSA for final c_{assay} in 50 µL at 1 mg/mL			

For the labeling of albumin ***ps*****GSL$_{PEG}$(NO$_2$)-BODIPY** were incubated with nitroreductase (NfsB) from *E. coli* (Sigma-Aldrich, #N9284, Lot No. 0000206122), NADH (Sigma-Aldrich, #N8129, Lot No. SLCB5013) and FMN (Sigma-Aldrich, #F6750, Lot No. MKCJ3258) at 37°C as described above in presence of bovine serum albumin (BSA, Sigma-Aldrich, #A9418, 1 mg/mL) (see Table S2). Clear labeling of BSA was observed with both ***ps*****GSL$_{PEG}$(NO$_2$)-BODIPY** and ***ps*****GSL$_{PEG}$(NO$_2$)-DNSA**, which was confirmed by staining of the gels with Coomassie blue™ (0.1% Brilliant Blue R 250 in 10% acetic acid, 40% dH$_2$O, 50% MeOH) for 1 h at 23°C with gentle shaking and de-stained in de-staining solution (10% acetic acid, 40% dH$_2$O, 50% MeOH) within 48 h at 23°C with gentle shaking and several changes of the de-staining solution.

HRMS analysis of NfsB labelled with *ps*GSL$_{PEG}$(NO$_2$)-N$_3$

The NfsB protein was produced recombinantly from *E. coli* (Sigma-Aldrich, #N9284, Lot No. 0000206122, Source 0000204168. The protein sequence corresponds to Uniprot P38489 with a His$_6$-tag (223 AA, theoretical mass: 24727.8 Da).

Protein masses were determined by liquid chromatography coupled to mass spectrometry (LC/MS). A UPLC (Ultimate 3000RS, Thermo Fisher Scientific, Dreieich, Germany), equipped with autosampler, binary high gradient pump, column oven, 6-port-column-switching-option and DAD-detector was used for sample separation via a C4 column (Xbridge BEH300, 3.5 µm pore size, 4.6 x 50 mm, Waters, Eschborn, Germany). As solvents water with 0.1% formic acid and acetonitrile with 0.1% formic acid were used at a flowrate of 900 µl/min. The used gradient started at 1%B up to 100% B within 6 minutes, holding 100% for 30 seconds and returning to the starting conditions. Masses were measured with a time-of-flight mass spectrometer (maxis HD UHR-TOF, Bruker Bremen, Germany) equipped with an Apollo II electrospray source. The MS was used in ESI positive ion mode, scan range 500-4000 m/z, capillary voltage 4500V, nebulizer pressure 4 bar, dry heater 200°C, dry gas 9 l/min, transfer time 115 µs, prepuls storage 30 µs. For HR mass calibration, a sodium formiate cluster was infused within the first 0,3 minutes of each run and used as calibrant for the internal mass calibration. Additional hexakis(2,2-difluoroethoxy)phosphazene was used for lock mass calibration. Calibration were done in DataAnalysis 6.1 (Calibration Mode HPC + LockMass). The deconvolution of measured protein mass spectra where performed with the MaximumEntropy module of DataAnalysis to get the singly charged mass spectrum for every protein.

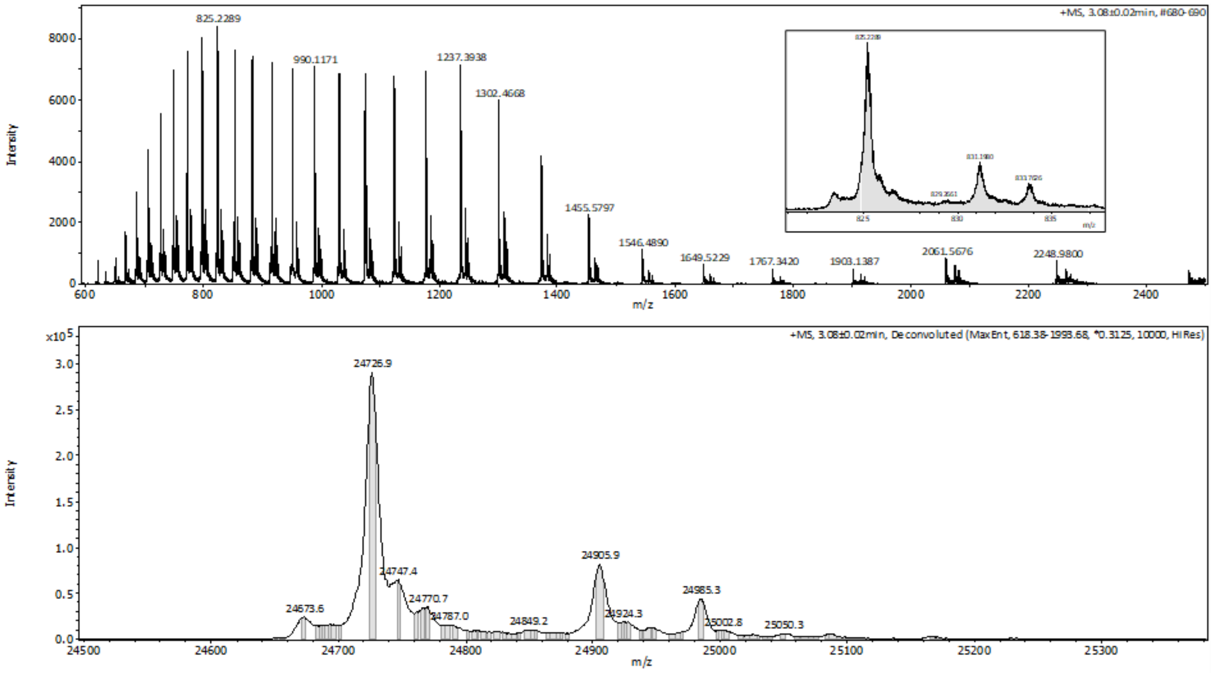

Figure S3. ESI mass spectrum (top) and deconvoluted spectrum (bottom) of nitroreductase NfsB from *E. coli*. The insert is a zoom-in of the highest peak at 825.2289 Da (+30 charge state). The experimental deconvoluted protein mass is 24726.9 Da (expected mass: 24727.8 Da).

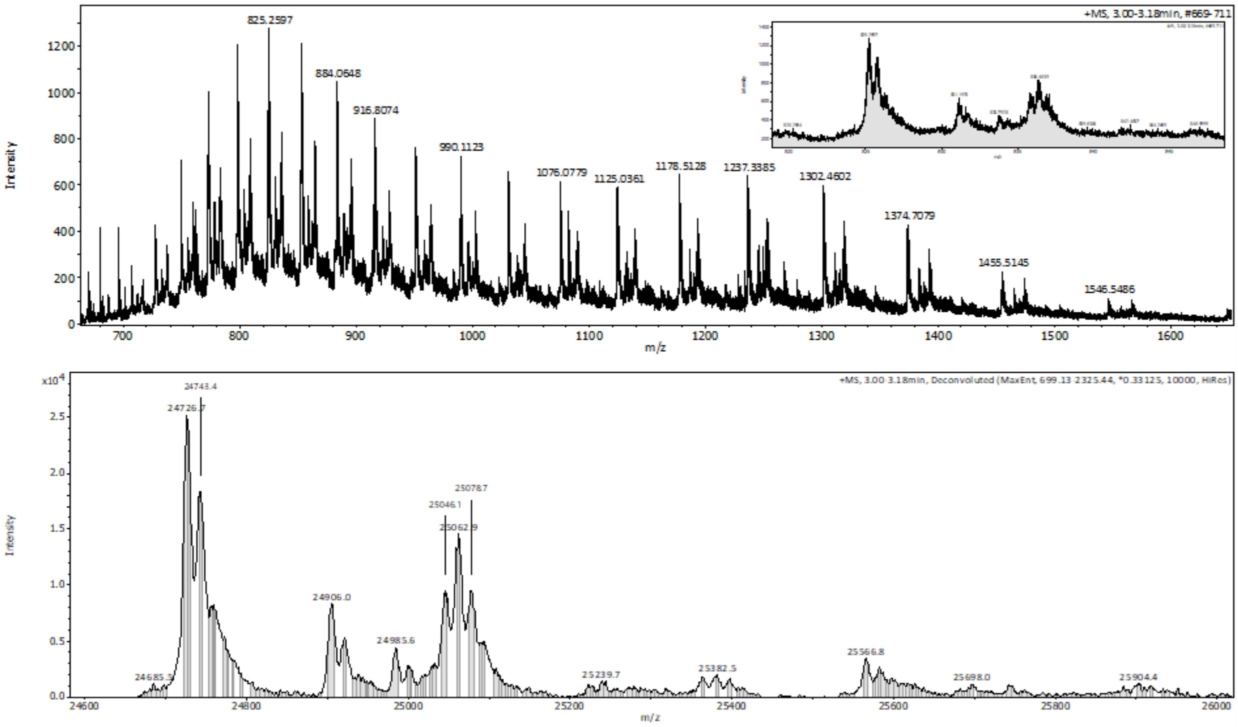

Figure S4. ESI mass spectrum (top) and deconvoluted spectrum (bottom) of nitroreductase NfsB from *E. coli* labelled with ITC **19** originating from ***ps*GSL$_{\mathbf{PEG}}$(NO$_2$)-N$_3$**. The insert is a zoom-in of the highest peak at 825.2289 Da (+30 charge state). A new mass of 25062.9 Da, corresponding to NfsB modified with ITC **19**, is visible.

Modification of synthetic model peptides with *ps*GSL(NO_2)-BODIPY$_{FL}$

Labeling of model **peptides 1-5** was conducted by incubation of ***ps*GSL(NO_2)-BODIPY$_{FL}$** following 3 different conditions.

Condition A: *ps*GSL(NO_2)-BODIPY$_{FL}$ (500 µM) was incubated with 5 mM NADH, 20 µM FMN, ~20 µg of protein (**model peptide 1-5**) and 10 µM nitroreductase (*NfsB*) from *E. coli* (Sigma-Aldrich, #N9284) in 20 mM HEPES buffer (Sigma-Aldrich, #H4034, Lot No. SLCD6051) pH 7.4 (total volume 50 µL) at 37°C for 1 h (Concentrations of stock solutions are given in Table S3). 4°C cold CH_3CN (50 µL) was added to precipitate *NfsB* and the mixture was centrifuged at 4°C and 7800 x *g* for 2 min twice using Centrifugal Nanosep column with 10K Omega (LOT: FJ4655) to separate the protein precipitate from the supernatant. Then samples of the supernatant (25 µl corresponding to approximately 10 µg of respective peptide) were purified using Pierce peptide desalting spin columns (Thermo Scientific), according to the manufacturer´s instructions.

Condition B: *ps*GSL(NO_2)-BODIPY$_{FL}$ (500 µM) was incubated with 5 mM NADH, 20 µM FMN, ~20 µg of protein (**model peptide 1-5**) and 10 µM nitroreductase (*NfsB*) from *E. coli* (Sigma-Aldrich, #N9284) in 20 mM HEPES buffer (Sigma-Aldrich, #H4034, Lot No. SLCD6051) pH 7.4 (total volume 50 µL) at 37°C for 1 h (Concentrations of stock solutions are given in Table S3). DiPEA (2 µL) was added resulting in a pH of 8.5-9 and the resulting mixtures were incubated for additional 2 h at 37°C. Afterward, 4°C cold CH_3CN (48 µL) was added to precipitate *NfsB* and the mixture was centrifuged at 4°C and 7800 x g for 2 min twice using Centrifugal Nanosep column with 10K Omega to separate the protein precipitate from the supernatant. Then samples of the supernatant (25 µl corresponding to approximately 10 µg of respective peptide) were purified using Pierce peptide desalting spin columns (Thermo Scientific), according to the manufacturer´s instructions.

Conditions C: *ps*GSL(NO_2)-BODIPY$_{FL}$ (500 µM) was incubated with 5 mM NADH, 20 µM FMN, ~20 µg of protein (**model peptide 1-5**) and 10 µM nitroreductase (*NfsB*) from *E. coli* (Sigma-Aldrich, #N9284) in 20 mM HEPES buffer (Sigma-Aldrich, #H4034, Lot No. SLCD6051) pH 7.4 (total volume 50 µL) at 37°C for 1 h (Concentrations of stock solutions are given in Table S3). 4°C cold CH_3CN (50 µL) was added in the first aliquot to precipitate *NfsB* and the mixture was centrifuged at 4°C and 7800 x *g* for 2 min twice using Centrifugal Nanosep column with 10K Omega (LOT: FJ4655) to separate the protein precipitate from the supernatant. DiPEA (2 µL) was added resulting in a pH of 8.5-9 and the resulting mixtures were incubated for additional 2 h at 37°C. Then a solution of TEAB (2 µL, 0.1 M in H_2O) was added resulting in a pH of 8.5-9 and the resulting mixture was subjected to an additional 2 h incubation at 37°C. Afterward, 4°C cold CH_3CN (46 µL) was added to precipitate *NfsB* and the mixture was centrifuged at 4°C and 7800 x g for 2 min twice using Centrifugal Nanosep column with 10K Omega to separate the protein precipitate (NfsB) from the supernatant. Then samples of the supernatant (25 µl corresponding to approximately 10 µg of respective peptide) were purified using Pierce peptide desalting spin columns (Thermo Scientific), according to the manufacturer´s instructions.

Table S3. Concentrations of stock solutions for enzymatic assays using nitroreductase (*NfsB*).

Compound	C_{Stock}	V_{Stock}	final C_{Assay} in 50-54 µL
***ps*GSL(NO_2)-BODIPY$_{FL}$**	5 mM in DMSO	5 µL	500 µM
NADH	10 mM in HEPES buffer 20 mM, pH 7.4	25 µL	5 mM
FMN	2 mM in HEPES buffer 20 mM, pH 7.4	0.5 µL	20 µM
NfsB	41.7 µM in HEPES buffer 20 mM, pH 7.4	12 µL	10 µM
**Model peptide 1-5**	5 mM in HEPES buffer 20 mM, pH 7.4-9	5 µL	500 µM
DIPEA		2 µL	
TEAB	0.1 M in H_2O	2 µL	
+ 2.5 µL HEPES buffer 20 mM, pH 7.4; + 46-50 µL Acetonitrile (cold), pH 7.4-9)			

LC-MS analysis: The samples were analyzed on an Orbitrap Lumos Tribrid mass spectrometer interfaced with an Easy-nLC1200 liquid chromatography system (both Thermo Fisher Scientific). Peptides (1 µg) were trapped on an Acclaim Pepmap 100 C18 trap column (100 µm x 2 cm, particle size 5 µm, Thermo Fisher Scientific) and separated on an in-house packed analytical column (45 cm x 75 µm, particle size 3 µm, Reprosil-Pur C18, Dr. Maisch) using a gradient from 4% to 28% acetonitrile in 0.1% formic acid over 32 min at a flow of 300 nL/min, followed by 13 minutes wash with 80% of acetonitrile in 0.1% formic acid. The precursor ion mass spectra were acquired at a resolution of 120 000 and an m/z range of 375-1500. Using a cycle time of 3 seconds and exclusion time of 30 seconds the most abundant precursors with charges 2–6 were isolated with an m/z window of 1.4 and fragmented by collision-induced dissociation (CID) at 30%. Fragment spectra were recorded in the Orbitrap at a resolution of 30000.

Proteomic data analysis: Raw files were processed and analyzed with Proteome Discoverer (Ver 3.0, Thermo Scientific). The data was matched against peptide sequences using Sequest as a search engine with a precursor tolerance of 5 ppm and a fragment ion tolerance of 0.03 Da. Addition of thiourea/dithiocarbamate (C,K), amidine (C/K) and deamidation (N) were set as variable modifications and cysteine carbamidomethylation was set as a fixed modification

Modification of the proteome of *S. aureus* SH1000 with *ps*GSL(NO_2)-alkyne

The chemoproteomic experiments were performed identical to an already published procedure.[12] Two identical samples of 1.00 mL freshly prepared lysate of *S. aureus* SH1000 (a kind gift from Simon J. Foster, The Krebs Institute, Department of Molecular Biology and Biotechnology, University of Sheffield)[13] were incubated with 10 µL flavin mononucleotide (2.5 mM stock in PBS for a final concentration of 25 µM), 20 µL NADH (50 mM stock in PBS for a final concentration of 1 mM), 10 µL ***ps*GSL(NO_2)-alkyne** (10 mM stock in DMSO for a final concentration of 100 µM) and 20 µL nitroreductase *NfsB* from *E. coli* (505 µM stock in 20 mM Tris-HCl and 150 mM NaCl buffer for a final concentration of 10 µM) for one hour at 37 °C while shaking (200 rpm). One sample was clicked to the heavy and one to the light isoDTB tag[14] by adding 120 µL of a solution consisting of 60 µL TBTA ligand (0.9 mg/mL in 4:1 *t*BuOH/DMSO), 20 µL $CuSO_4$ 5 H_2O (12.5 mg/L in H_2O), 20 µL TCEP (13 mg/mL in H_2O) and 20 µL of the respective isoDTB tag (5 mM in DMSO). After incubation of the samples (1 h, 25 °C), the light- and heavy-labelled samples were combined into 8 mL cold acetone to precipitate all proteins. Precipitates were stored at −20°C overnight.

MS sample preparation: The protein precipitates were centrifuged (3,500 ×g, 10 min, 25 °C) and the supernatant was removed. The precipitates were resuspended in 1 mL cold MeOH by sonification and centrifuged (10 min, 21,100 ×g, 4 °C). The supernatant was removed and the washing step with MeOH was repeated once. The pellets were dissolved in 300 µL urea (8 M in 0.1 M aqueous triethylammonium bicarbonate (TEAB) by sonification. 900 µL TEAB (0.1 M in H_2O) were added, and this solution was added to 1.2 mL of washed high capacity streptavidin agarose beads (50 µL initial slurry, Fisher Scientific, 10733315) in NP40 substitute (0.2% in PBS). The samples were rotated for 1 h at 25 °C to assure binding to the beads. The beads were centrifuged (1 min, 1,000 ×g, 25 °C) and the supernatant was removed. The beads were resuspended in 600 µL NP40 substitute (0.1% in PBS) and transferred to a centrifuge column (Fisher Scientific, 11894131). Beads were washed with 2 × 600 µL NP40 substitute (0.1% in PBS), 3 × 600 µL PBS and 3 × 600 µL H_2O. The beads were resuspended in 600 µL urea (8 M in 0.1 M aqueous TEAB), transferred to a Protein LoBind tube (Eppendorf) and centrifuged (1 min, 1,000 ×g). The supernatant was removed, the beads were resuspended in 300 µL urea (8 M in 0.1 M aqueous TEAB), incubated sequentially with 15 µL dithiothreitol (DTT; 31 mg/mL in H_2O) (45 min, 200 rpm, 37 °C), 15 µL iodoacetamide (74 mg/mL in H_2O) (30 min, 200 rpm, 25 °C) and 15 µL DTT (31 mg/mL in H_2O) (30 min, 200 rpm, 25 °C). The samples were diluted with 900 µL TEAB (0.1 M in H_2O) and centrifuged (1 min, 1,000 ×g). After removal of the supernatant, the beads were resuspended in 200 µL urea (2 M in 0.1 M aqueous TEAB) and incubated with 4 µL trypsin (0.5 mg/mL; Promega, V5113) (overnight, 200 rpm, 37 °C). The samples were diluted by adding 400 µL NP40 substitute (0.1% in PBS) and transferred to a centrifuge column (Fisher Scientific, 11894131). Beads were washed with 3 × 600 µL NP40 substitute (0.1% in PBS), 3 × 800 µL PBS and 3 × 800 µL H_2O. Peptides were eluted into Protein LoBind tubes with 1 × 200 µL and 2 × 100 µL trifluoroacetic acid (TFA) (0.1% in 50% aqueous MeCN) followed by a final centrifugation (3 min, 3,000 ×g). The solvent was removed in a rotating vacuum concentrator (~5 h, 30 °C) and the resulting residue was dissolved in 30 µL TFA (0.1% in H_2O) by sonification for 5 min. Samples were filtered through filters (Merck, UVC30GVNB) washed with the same solution by centrifugation (3 min, 17,000 ×g). The samples were then transferred to MS sample vials and stored at −20 °C until measurement.

Sample analysis by LC-MS/MS: 5 µL of the samples were analyzed using a Qexactive Plus mass spectrometer (ThermoFisher) coupled to an Ultimate 3000 nano HPLC system (Dionex). Samples were loaded on an Acclaim C18 PepMap100 trap column (75 µm ID × 2 cm, Acclaim, PN 164535) and washed with 0.1% TFA. The subsequent separation was carried out on an AURORA series AUR2-25075C18A column (75 µm ID × 25 cm, Serial No. IO257504282) with a flow rate of 400 nL/min using buffer A (0.1% formic acid in water) and buffer B (0.1% formic acid in acetonitrile). The column was heated to 40 °C. Analysis started with washing in 5% B for 7 min followed by a gradient from 5% to 40% buffer B over 105 min, an increase to 60% B in 10 min and another increase to 90% B in 10 min. 90% B was held for 10 min, then decreased to 5% in 0.1 min and held at 5% for another 9.9 min. The Qexactive Plus mass spectrometer was run in a TOP10 data-dependent mode. In the orbitrap, full MS scans were collected in a scan range of 300-1500 m/z at a resolution of 70,000 and an AGC target of 3e6 with 80 ms maximum injection time. The most intense peaks were selected for MS2 measurement with a minimum AGC target of 1e3 and isotope exclusion and dynamic exclusion (exclusion duration: 60 s) enabled. Peaks with unassigned charge or a charge of +1 were excluded. Peptide match was "preferred". MS2 spectra were collected at a resolution of 17,500 aiming at an AGC target of 1e5 with a maximum injection time of 100 ms. Isolation was conducted in the quadrupole using a window of 1.6 m/z. Fragments were generated using higher-energy collisional dissociation (HCD, normalized collision energy: 27%) and finally detected in the orbitrap.

isoDTB-ABPP Data Analysis

General setup of analysis software: Raw data of the LC-MS/MS analyses was converted into the mzML format using the MSconvert tool (version: 3.0.19172-57d620127) of the ProteoWizard software (version: 3.0.19172 64bit)[15] using standard settings with vendor's peak picking enabled. For all data analysis using MSFragger-based FragPipe,[16,17] the FragPipe interface (version: 14.0) was used with MSFragger (version: 3.1.1),[16,17] Philosopher (version: 3.3.10),[18] IonQuant (version 1.4.6)[19] and Python (version: 3.7.3) enabled. A FASTA database for *S. aureus* SH1000 was downloaded from www.uniport.org using a search for "93061" as "Taxonomy [OC]" at UniProtKB on 27.02.2018. This corresponds to the FASTA database for the strain NCTC8325. Comparative sequencing of these two strains has been reported[20] and the respective changes have been manually made to the FASTA file. These include several point mutations, the deletion of the partial proteins Q2FWJ0 and Q2FWJ1 as well as the addition of the rsbU gene from *S. aureus* Newman (A0A0H3KE27). *E. coli NfsB* (P38489) was added to the FASTA database. The reverse sequences were manually added to the FASTA databases.

Analysis of the mass of modifications with FragPipe: To survey the landscape of all mass shifts observed on peptides in the data sets, an OpenSearch was performed with MSFragger.[16,17] For this purpose, the following settings were used: Precursor mass tolerance −150 to 1000 Da, (initial) fragment mass tolerance 20 ppm, Calibration and Optimization "Mass calibration, parameter optimization" enabled, Isotope Error "0", enzyme name "trypsin", cut after "KR", but not before "P", cleavage "enzymatic", missed cleavages "2", Clip N-term N enabled, peptide length 7 to 50, peptide mass range 500 to 5000 Da, no variable modifications, no fixed modifications, all other options were left at the standard settings. Crystal-C was enabled. PeptideProphet was run with the following settings: "--nonparam --expectscore --decoyprobs --masswidth 1000.0 -- clevel -2". PTMProphet was disabled. ProteinProphet was run with the following settings: "--maxppmdiff 2000000". Generate report was enabled with the following settings: "--sequential --razor --mapmods --prot 0.01". Run MS1 quant was disabled. Run TMT-Integrator was disabled. PTM-Shepherd was enabled with the following settings: Smoothing factor "2", Precursor tolerance "0.01 Da", Prominence ratio "0.3", Peak picking width "0.002 Da", Localization background "4", Annotation tolerance "0.01 Da", Custom mass shifts: a custom mass shift list was used including only UniMod modifications with less than 400 Da molecular weight as previously published[12], Ion Types for modification with "b" and "y" enabled and mass fragment charge "2". Generate Spectral Library was disabled. For downstream data analysis, the "global.modsummary.tsv" file was loaded as previously published and the values for the number of PSMs ("default-ptmshepherd-dataset (PSMs)") were plotted against the "Theoretical Mass Shift" in the mass range between 400 and 1000 Da. The main detected mass shifts were 574.3310 Da and 580.3382 Da for the light and the heavy isoDTB-tag, respectively.

Analysis of amino acid selectivity with FragPipe: To analyse the amino acid selectivity, an Offset Search was performed in MSFragger[16,17] For this purpose, the following settings were used in MSFragger: Precursor mass tolerance −20 to 20 ppm, fragment mass tolerance 20 ppm, Calibration and Optimization "None", Isotope Error "0/1/2", enzyme name "trypsin", cut after "KR", but not before "P", cleavage "enzymatic", missed cleavages "2", Clip N-term N enabled, peptide length 7 to 50, peptide mass range 500 to 5000 Da, variable modification of 57.02146 Da on C with max. 3 occurrences, no fixed modifications, mass offsets set according to the detected mass shifts), all other options were left at the standard settings. Crystal-C was disabled. PeptideProphet was run with the following settings: "--nonparam --expectscore --decoyprobs --masswidth 1000.0 -- clevel -2". PTMProphet was disabled. ProteinProphet was run with the following settings: "--maxppmdiff 2000000". Generate report was enabled with the following settings: "--sequential --razor --mapmods --prot 0.01". Run MS1 quant was enabled with the following settings: IonQuant enabled, M/Z Window "10 ppm", RT Window "0.4 min", Labeling based quant with the detected masses on any amino acid as indicated by "*",Re-quantify enabled, Top N ions "3" Min freq. "0.5", Min exps "1", Min isotopes "2", Normalize disabled. Run TMT-Integrator was disabled. PTM-Shepherd was enabled with the following settings: Smoothing factor "2", Precursor tolerance 20 ppm, Prominence ratio "0.3", Peak picking width "20 ppm", Localization background "4", Annotation tolerance "0.01 Da", Custom mass shifts: "Failed_Carbamidomethylation:-57.021464", a custom mass shift list was used including only UniMod modifications with less than 400 Da molecular weight as previously published,[12] Ion Types for modification with b and y enabled and mass fragment charge "2". Generate Spectral Library was disabled. Both runs were analysed as the same experiment. For downstream data analysis, the two "*.tsv" files for the two experiments were individually processed. They were filtered to retain only entries that are present in the "psm.tsv" file, which contains the PSMs filtered by 1% PSM- and protein-level FDR. The column "best locs" indicates the possible residues modified by the mass offset. MSFragger puts the mass offset on each residue one-by-one and calculates hyperscores. The residues with the highest hyperscore are indicated by lower-case letters. Only entries were retained that were localized to a unique residue as seen by containing one lower-case letter (If there is no lower-case letter, the score for the unmodified peptide was higher than that for the best modified peptide and therefore no localization was performed). Next, the entries were filtered for a delta score > 1, where delta score is the difference of the highest hyperscore and the second highest hyperscore during the localization. For each entry, the UniProt Code was isolated from the column "Protein" and the full protein sequence was linked into the table. Based on this information, all peptide sequences that do not occur exactly once in the identified protein were excluded and the residue number of the modified residue was determined. Next, the amino acid at the modified residue was determined. If the *N*-terminus (modification at amino acid 1 or amino acid 2 if amino acid 1 is not present in the peptide (clipping of *N*-terminal methionine)) or the *C*-terminus (last amino acid of the protein) were modified, this was only counted and labelled as modification of the terminus and not of the respective amino acid at that position. For each entry, an identifier was generated in the format "UniProtCode"_X_"residue number", where X is the one letter code of the modified amino acid or "N-terminal" or "C-terminal" for terminal modifications. Duplicates of entries with the same identifier were retained only once. The data of both experiments was then combined and only residues were counted in the final analysis that were present in both replicates. The fraction of all sites that was modified at each amino acid and the termini was reported. In some cases, amino acids with the same reactive group (D+E, N+Q and S+T) were clustered together.

Quantification with FragPipe: For this purpose, the following settings were used in MSFragger[16,17]: Precursor mass tolerance −50 to 50 ppm, fragment mass tolerance 20 ppm, Calibration and Optimization "None", Isotope Error "0/1/2", enzyme name "trypsin", cut after "KR", but no before "P", cleavage "enzymatic", missed cleavages "2", Clip N-term N enabled, peptide length 7 to 50, peptide mass range 500 to 5000 Da, fixed modification of 57.20146 Da on C, no mass offsets, All other options were left at the standard settings. Variable modifications were set to the masses of modification on lysines, *N*-terminus and *C*-terminus with max. 1 occurrence. Crystal-C was disabled. PeptidePropeht was run with the following settings: "—decoyprobs –ppm –accmass –nonparam –expectscore". PTMProphet was disabled. ProteinProphet was run with the following settings: "—maxppmdiff 2000000". Generate report was enabled with the following settings: " –sequential –razor –mapmods –prot 0.01". Run MS1 quant was enabled with the following settings: IonQuant enabled, M/Z Window "10 ppm", RT Window "0.4 min", Labeling based quant with the masses of modification on lysines, *N*-terminus and *C*-terminus, Re-quantify enabled,

Top N ions "3" Min freq. "0.5", Min exps "1", Min isotopes "2", Normalize disabled Run TMT-Integrator was disabled. PTM-Shepherd was disabled. Generate Spectral Library was disabled. For downstream data analysis, the "ion_label_quant.tsv" files of the two experiments were analysed separately. For each entry, the "Modified peptide" was generated as either the "Light Modified Peptide" or the "Heavy Modified Peptide" based on the entry with the higher "PeptideProhpet Probability". The Masses of probe modification in the "Modified Peptide" were replaced by an "*" and the mass of carbamidomethylation ([57.2015]) in this entry was deleted, if present. The full protein sequence was linked into the table. Based on this information, all peptide sequences that do not occur exactly once in the same protein were excluded and the residue number of the modified residue was determined. The "Identifier" was generated in the format "UniProtCode"_X_"residue number", where X is the one letter code of the modified amino acid or "N-term" or "C-term" for terminal modifications. For each "Identifier", the averaged "Log2 ratio HL", which is the $\log_2$ transformed ratio of heavy and light ions, was determined as average of the "Log2 ratio HL" of all corresponding ions weighted with the "Total intensity" of the ion, which was calculated as the sum of "Light Intensity" and "Heavy Intensity" for each ion. The value was disregarded if the standard deviation of the "Log2 ratio HL" values for all ions of the same "Identifier" was > 1.41. Furthermore, for each "Identifier" the "Total Intensity", "Total Light Intensity" and "Total Heavy Intensity" were calculated as the sum of all "Total Intensity", "Light Intensity" and "Heavy Intensity" values of the individual ions, respectively. If several different "Modified peptides" were detected for the same "Identifier", the "Modified Peptide" and "Peptide Sequence" with the shortest sequence were kept. For all identifiers, the data for both replicates was now combined into one table. If different "Modified peptide" were detected for the same "Identifier" in the different replicates, the "Modified Peptide" and "Peptide Sequence" with the shortest sequence were kept. The "Total Intensity", "Total Light Intensity" and "Total Heavy Intensity" was calculated as the sum of all "Total Intensity", "Total Light Intensity" and "Total Heavy Intensity" values for all replicates, respectively. The "Log2 ratio HL" values for the replicates were named "Log2 ratio HL replicate 1" and "Log2 ratio HL replicate 2". The average of these two values was calculated and named "Log2 ratio HL". The value was disregarded, if the standard deviation between the replicates was > 1.41 or if the identifier was only quantified in one of the replicates. The "Log2 ratio HL" data for all "Identifiers" was plotted as a violin plot with all individual values shown. The data was visualized for lysines, *N*-terminus and *C*-terminus individually. The expected value of the ratio ($\log_2(R) = 0$) as well as the preferred quantification window ($-1 < \log_2(R) < 1$) were indicated by dashed lines.

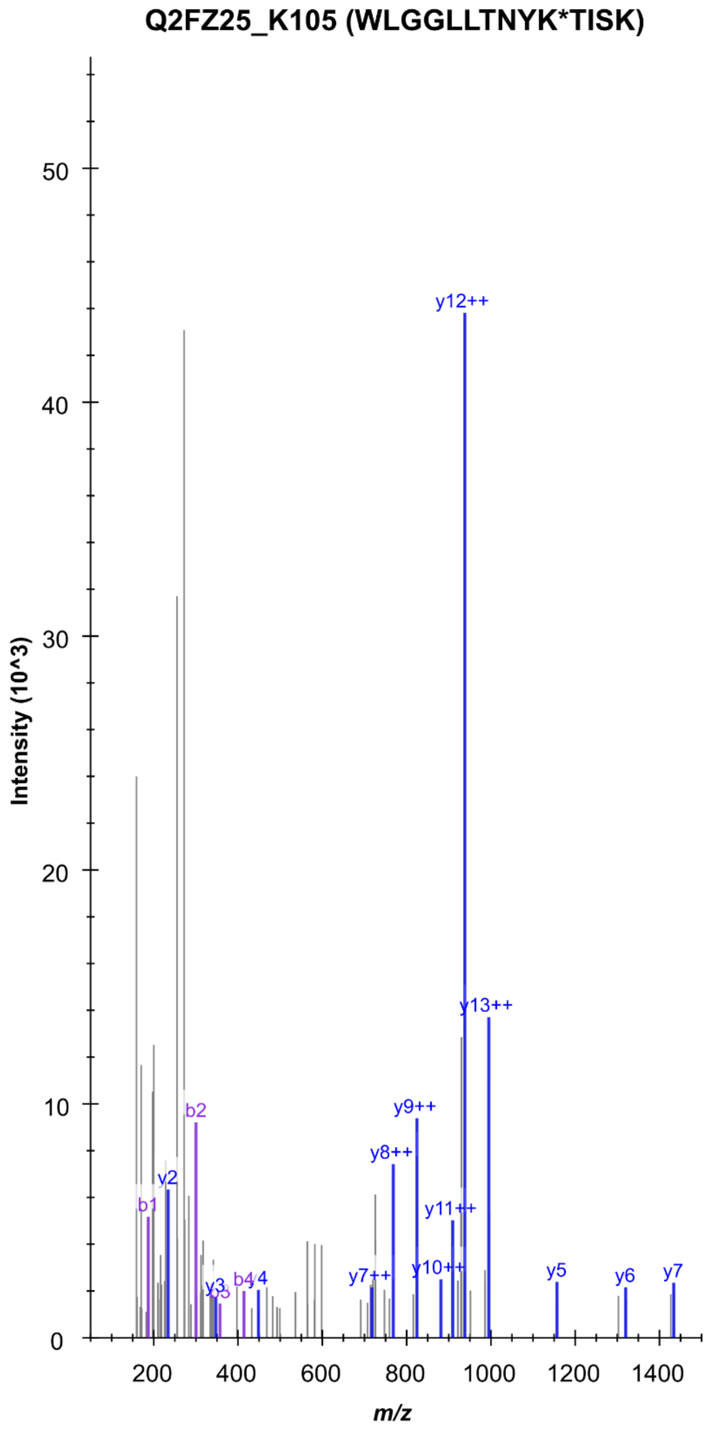

Figure S5. Representative MS/MS spectra of the relative b and y ions of the amidine modified lysine K105.

LC-MS analysis of amidine formation from the nitro-reductase mediated release of ITCs from *ps*GSLs

Incubation of ***ps*GSL$_{PEG}$(NO$_2$)-N$_3$** was carried out with a substrate concentration of 500 µM with 5 mM NADH, 20 µM FMN, and 10 µM nitro-reductase (NfsB) from *E. coli* (Sigma-Aldrich, #N9284) in 20 mM HEPES buffer pH 7.4 (total volume 50 µL) at 37°C for 2 h (Concentrations of stock solutions are given in Table S1). 4°C cold CH_3CN (50 µL) was added to precipitate NfsB and the mixture was centrifuged at 4°C and 7800 x *g* for 2 min twice using Centrifugal Nanosep column with 10K Omega (LOT: FJ4655) to separate the protein precipitate from the supernatant. The filtrate was immediately used to analyze the resulting ITC by LCMS at λ = 254 nm (Eluent mixture: H_2O:CH_3CN + 0.01% FA 10-90%, 4.5 min).
The thiourea was prepared by adding methylamine (30% aq. 20 µL) to 100 µL of the ITC containing solution at 23°C and incubating for 5 min. The thiourea was analyzed by LCMS at λ = 254 nm (Eluent mixture: H_2O:CH_3CN + 0.01% FA 10-90%, 4.5 min). The thiourea solutions was further divided into two separate LCMS vials containing 50 µL to which TFA (20 µL) and FA (20 µL) were added respectively and incubated at 23°C for 19 h and the resulting amidines were analyzed by LCMS at λ = 254 nm (Eluent mixture: H_2O:CH_3CN + 0.01% FA 10-90%, 4.5 min).

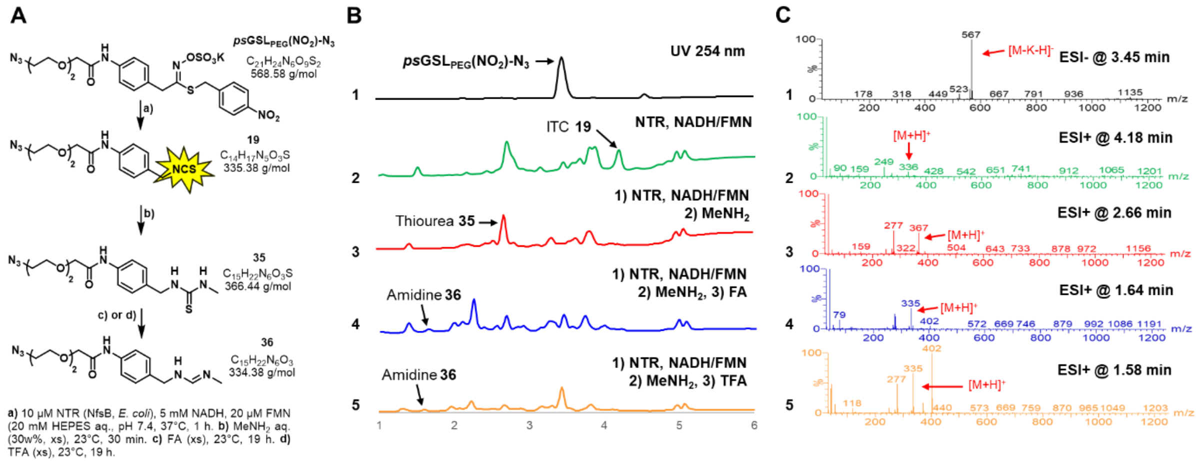

Figure S6. LC-MS analysis after incubation of ***ps*GSL$_{PEG}$(NO$_2$)** with nitroreductase (NTR) NfsB from *E. coli* and subsequent derivatization with $MeNH_2$ solution. **A:** Structures of ***ps*GSL$_{PEG}$(NO$_2$)-N$_3$**, ITC **19**, thiourea **35** and amidine **36**. **B:** UV chromatogram at 254 nm of (B1) pure ***ps*GSL$_{PEG}$(NO$_2$)-N$_3$** (500 µM) in HEPES buffer (20 mM, pH 7.4), (B2) incubation of ***ps*GSL$_{PEG}$(NO$_2$)** (500 µM) with NfsB (10 µM), NADH (5 mM) and FMN (20 µM) in HEPES buffer (20mM, pH 7.4) after 2 h at 37°C, (B3) addition of aqueous $MeNH_2$ solution (30 w%, 30 µL) at 23°C after 5 min and (B4) addition of FA (30 µL) at 23°C after 19 h or (B5) addition of TFA (30 µL) at 23°C after 19 h. **C:** ESI+ or ESI- mass analysis at (C1) 3.45 min of B1, (C2) 4.18 min of B2, (C3) 2.66 min of B3, (C4) 1.64 min of B4 and (C5) 1.58 min of B5.

Fluorescence microscopy imaging in *C. elegans* showing enzymatic conversion of *ps*GSLs into corresponding ITCs and covalent binding to intestinal lumen

C. elegans N2 hermaphrodites were grown on NGM (normal growth media) agar plates at 20°C with OP50 E. coli.[21] Day 1 adult worms were used in all experiments. The worms were incubated in M9 buffer (22 mM KH_2PO_4, 42 mM Na_2HPO_4, 85.6 mM NaCl, 0.1 mM $MgSO_4$) containing 500 µM of ***ps*GSL$_{PEG}$(NO$_2$)-BODIPY** for 1 h (total volume 100 µL), after which 10 µM of prokaryotic nitro-reductase (NfsB) from *E. coli* (Sigma-Aldrich, #N9284, previously dissolved in 20 mM HEPES buffer pH 7.4) and the co-factors 5 mM NADH and 20 µM FMN were added for an additional 2 h incubation. This was followed by a 1 h M9 incubation to wash out excessive amounts. The worms were imaged either immediately after the washing step, or put back on NGM plates with OP50 overnight to be imaged 24h after exposure. Three conditions were tested: (Table S4): only 500 µM of ***ps*GSL$_{PEG}$(NO$_2$)-BODIPY**, (Table S5): 500 µM of ***ps*GSL$_{PEG}$(NO$_2$)-BODIPY** and co-factors 5 mM NADH and 20 µM FMN and (Table S5): 500 µM of ***ps*GSL$_{PEG}$(NO$_2$)-BODIPY**, 10 µM of prokaryotic nitro-reductase (NfsB) from *E. coli* and co-factors 5 mM NADH and 20 µM FMN.

Table S4. B1 conditions: Concentrations of stock solutions of control experiment in *C. elegans* analyzed by fluorescence microscopy.

Compound	C_{Stock}	V_{Stock}	final C_{Assay} in 125 µL
*ps*GSL(NO_2)	8.25 mM in DMSO	3 µL	198 µM
+ 97.6 µL M9 buffer			

Table S5. B2 conditions: Concentrations of stock solutions for covalent labeling in *C. elegans* analyzed by fluorescence microscopy without addition of nitro-reductase.

Compound	C_{Stock}	V_{Stock}	final C_{Assay} in 125 µL
*ps*GSL(NO_2)	8.25 mM in DMSO	2.4 µL	198 µM
NADH	6.25 M in M9 buffer	0.8 µL	5 mM
FMN	5 mM in M9 buffer	0.4 µL	20 µM
+ 96.4 µL M9 buffer			

Table S6. B3 conditions: Concentrations of stock solutions for covalent labeling in *C. elegans* analyzed by fluorescence microscopy.

Compound	C_{Stock}	V_{Stock}	final C_{Assay} in 125 µL
*ps*GSL(NO_2)	8.25 mM in DMSO	3 µL	198 µM
NADH	6.25 M in M9 buffer	1.25 µL	5 mM
FMN	5 mM in M9 buffer	0.5 µL	20 µM
NfsB	41.7 µM in HEPES buffer 20 mM, pH 7.4	30 µL	10 µM
+ 90.5 µL M9 buffer			

Worms were mounted on 2% agarose in M9 pads and immobilized using 75 mM NaN_3. Images were collected using a Zeiss LSM 700inv or a Zeiss LSM 880 Airyscan microscope, using 20x/0.8 air or 40x/1.2 water immersion objectives. Z-stacks were collected at 0.5 µm distance, using either a 350 nm or 488 nm laser with a PMT or Airyscan detector (in confocal mode), a BP 420-480 + BP 495-550 filter, as well as a T-PMT detector for transmitted light. Z-projections were collapsed and processed for MAX intensity using Fiji/ImageJ.[22]

References

[1] M. Frigerio, M. Santagostino, S. Sputore, *J. Org. Chem.* **1999**, *64*, 4537–4538.

[2] Y. Perez-Riverol, J. Bai, C. Bandla, D. García-Seisdedos, S. Hewapathirana, S. Kamatchinathan, D. J. Kundu, A. Prakash, A. Frericks-Zipper, M. Eisenacher, M. Walzer, S. Wang, A. Brazma, J. A. Vizcaíno, *Nucleic Acids Res.* **2022**, *50*, D543–D552.

[3] Y. Nishio, R. Mifune, T. Sato, S. Ishikawa, H. Matsubara, *Tetrahedron Lett.* **2017**, *58*, 1190–1193.

[4] J. R. Guo, H. Y. Huang, Y. L. Yan, C. F. Liang, *Asian J. Org. Chem.* **2018**, *7*, 179–188.

[5] N. Dubey, P. Sharma, A. Kumar, *Synth. Commun.* **2015**, *45*, 2608–2626.

[6] M. Rauschenberg, E. C. Fritz, C. Schulz, T. Kaufmann, B. J. Ravoo, *Beilstein J. Org. Chem.* **2014**, *10*, 1354–1364.

[7] G. Clavé, H. Boutal, A. Hoang, F. Perraut, H. Volland, P. Y. Renard, A. Romieu, *Org. Biomol. Chem.* **2008**, *6*, 3065–3078.

[8] C. P. Glindemann, A. Backenköhler, M. Strieker, U. Wittstock, P. Klahn, *ChemBioChem* **2019**, *19*, 1668–1694.

[9] C. Kanstrup, C. C. Jimidar, J. Tomas, G. Cutolo, C. Crocoll, M. Schuler, P. Klahn, A. Tatibouët, H. H. Nour-Eldin, *Int. J. Mol. Sci.* **2023**, *24*, 920.

[10] T. Rizk, E. J.-F. Bilodeau, A. M. Beauchemin, *Angew. Chem. Int. Ed.* **2009**, *48*, 8325–8327.

[11] K. Kitamura, H. Itoh, K. Sakurai, S. Dan, M. Inoue, *J. Am. Chem. Soc.* **2018**, *140*, 12189–12199.

[12] P. R. A. Zanon, F. Yu, P. Z. Musacchio, L. Lewald, M. Zollo, K. Krauskopf, D. Mrdovic, P. Raunft, T. E. Maher, M. Cigler, C. J. Chang, K. Lang, F. D. Toste, A. I. Nesvihskii, S. M. Hacker, D. Mrdović, P. Raunft, T. E. Maher, M. Cigler, C. J. Chang, K. Lang, F. D. Toste, A. I. Nesvizhskii, S. M. Hacker, *ChemRxiv* **2021**, *1*, DOI: 10.26434/chemrxiv-2021-w7rss-v2.

[13] M. J. Horsburgh, J. L. Aish, I. J. White, L. Shaw, J. K. Lithgow, S. J. Foster, *J. Bacteriol.* **2002**, *184*, 5457–5467.

[14] P. R. A. Zanon, L. Lewald, S. M. Hacker, *Angew. Chem. Int. Ed.* **2020**, *59*, 2829–2836.

[15] D. Kessner, M. Chambers, R. Burke, D. Agus, P. Mallick, *Bioinformatics* **2008**, *24*, 2534–2536.

[16] A. T. Kong, F. V Leprevost, D. M. Avtonomov, D. Mellacheruvu, A. I. Nesvizhskii, *Nat. Methods* **2017**, *14*, 513–520.

[17] F. Yu, G. C. Teo, A. T. Kong, S. E. Haynes, D. M. Avtonomov, D. J. Geiszler, A. I. Nesvizhskii, *Nat. Commun.* **2020**, *11*, 4065.

[18] F. da Veiga Leprevost, S. E. Haynes, D. M. Avtonomov, H.-Y. Chang, A. K. Shanmugam, D. Mellacheruvu, A. T. Kong, A. I. Nesvizhskii, *Nat. Methods* **2020**, *17*, 869–870.

[19] F. Yu, S. E. Haynes, G. C. Teo, D. M. Avtonomov, D. A. Polasky, A. I. Nesvizhskii, *Mol. Cell. Proteomics* **2020**, *19*, 1575–1585.

[20] A. J. O'Neill, *Lett. Appl. Microbiol.* **2010**, *51*, 358–361.

[21] T. Stiernagle, in *WormBook*, **2006**, pp. 51–67.

[22] J. Schindelin, I. Arganda-Carreras, E. Frise, V. Kaynig, M. Longair, T. Pietzsch, S. Preibisch, C. Rueden, S. Saalfeld, B. Schmid, J.-Y. Tinevez, D. J. White, V. Hartenstein, K. Eliceiri, P. Tomancak, A. Cardona, *Nat. Methods* **2012**, *9*, 676–682.

[23] A. Brand, L. Allen, M. Altman, M. Hlava, J. Scott, *Learn. Publ.* **2015**, *28*, 151–155.

Author Contributions

Contribution are given with CRediT definition according to Brand et al..[23]

Conceptualization: PK; Methodology: CCJ, CSGG, MDK, AC, LW, MZ, LCC, JM and UB; Software: - ; Validation: CSGG, MZ and CCJ; Formal analysis: MZ; Investigation: CCJ, CSGG, MDK, AC, LW, MZ, LCC, JM, KB and UB; Resources: PK, MB, SAS, SMH and JM; Data curation: PK; Writing—original draft: CSGG, CCJ, AC, MDK, MZ, SMH and PK; Writing—review & editing: all authors; Visualization: PK, AC, CSGG, MDK, MZ, JM and SMH; Supervision: PK, MB, SAS, JM and SMH; Project administration: PK; Funding acquisition: PK, MB, SAS, JM and SMH.

.

Appendix: ^{1}H, ^{13}C, ^{19}F and ^{11}B NMR Spectra of the Compounds

1-(Bromomethyl)-4-nitrobenzene (**2**)

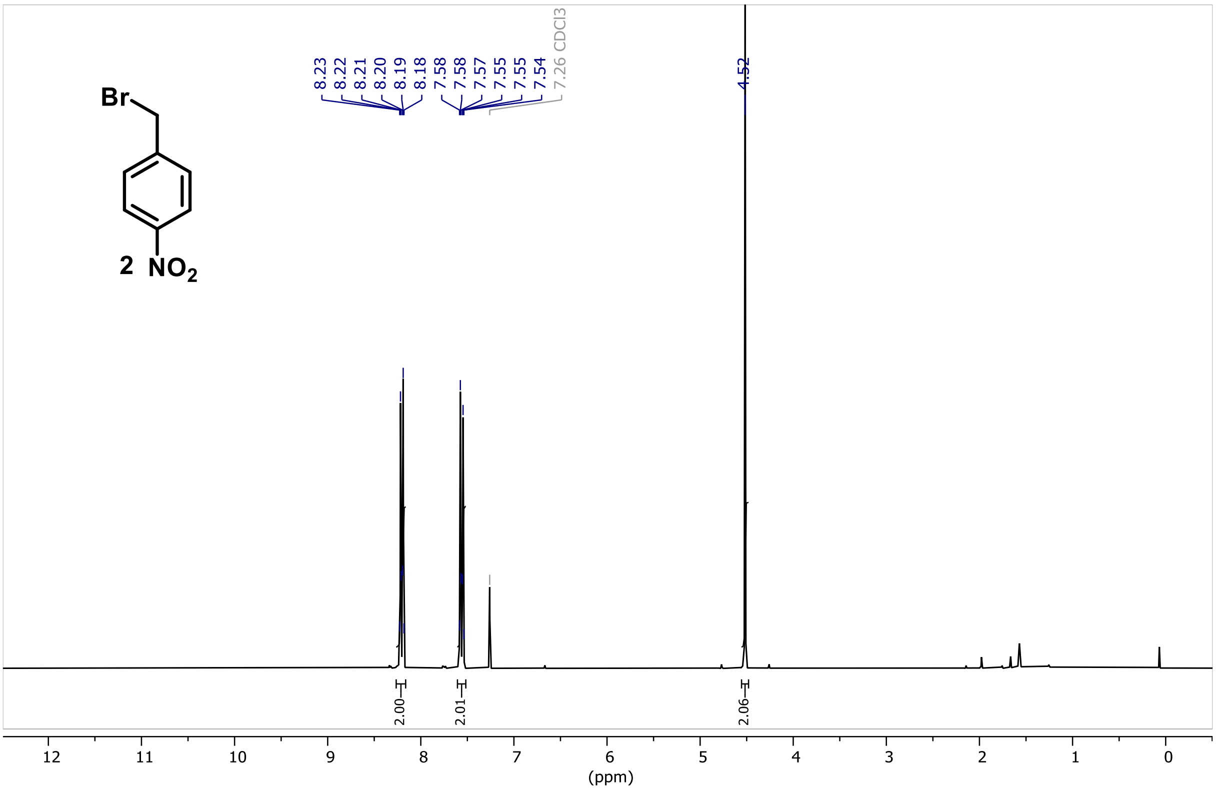

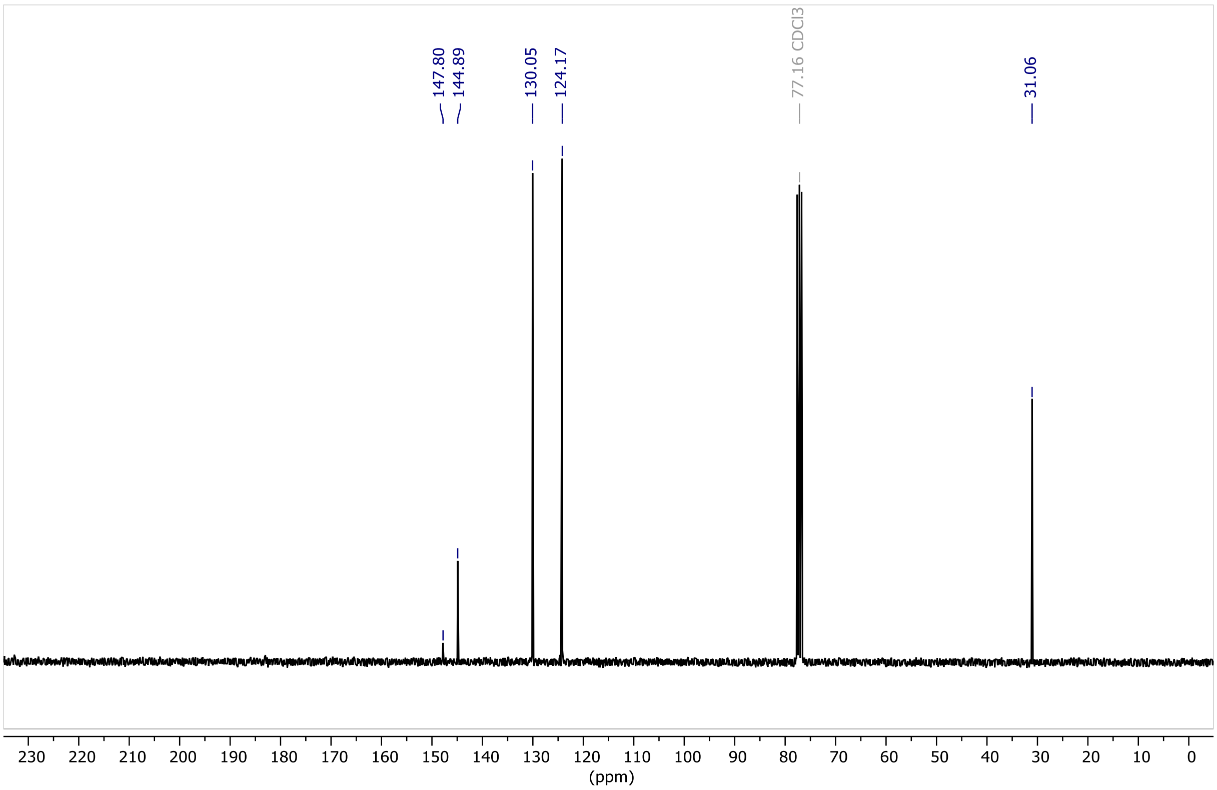

S-(4-Nitrobenzyl)ethanethioate (**3**)

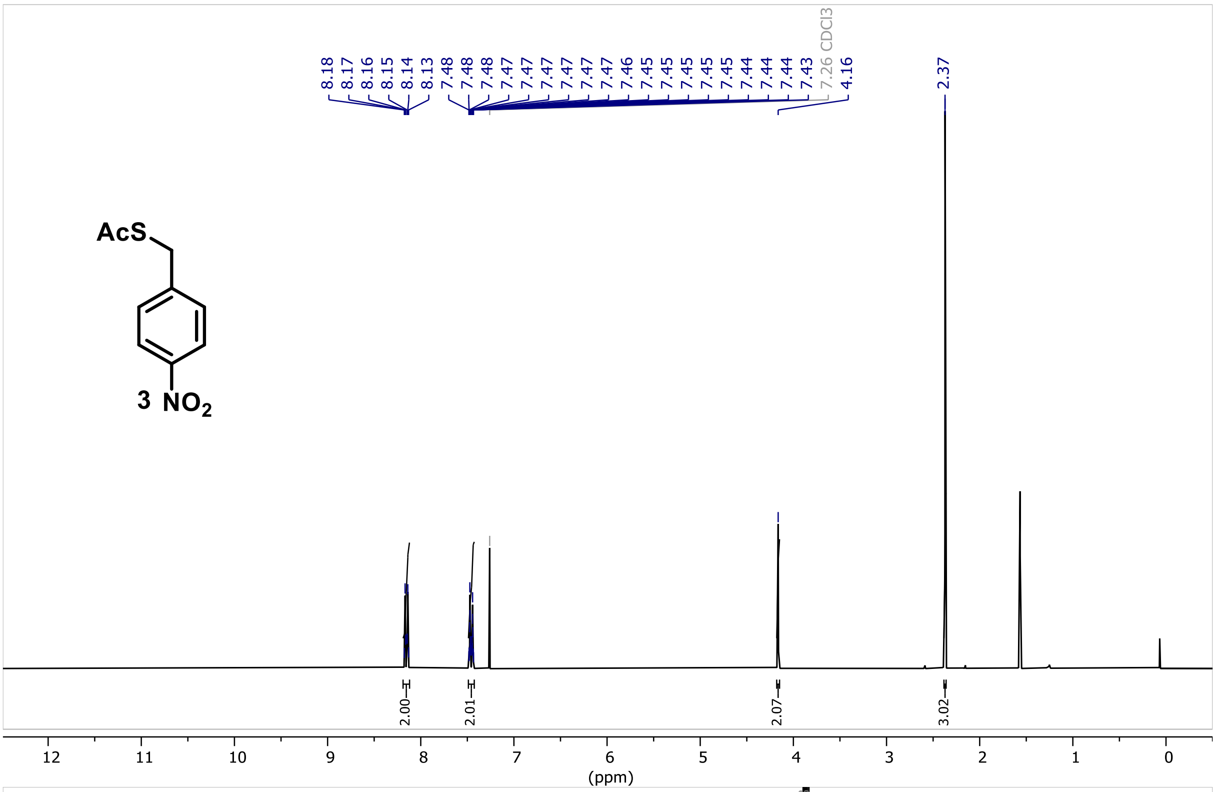

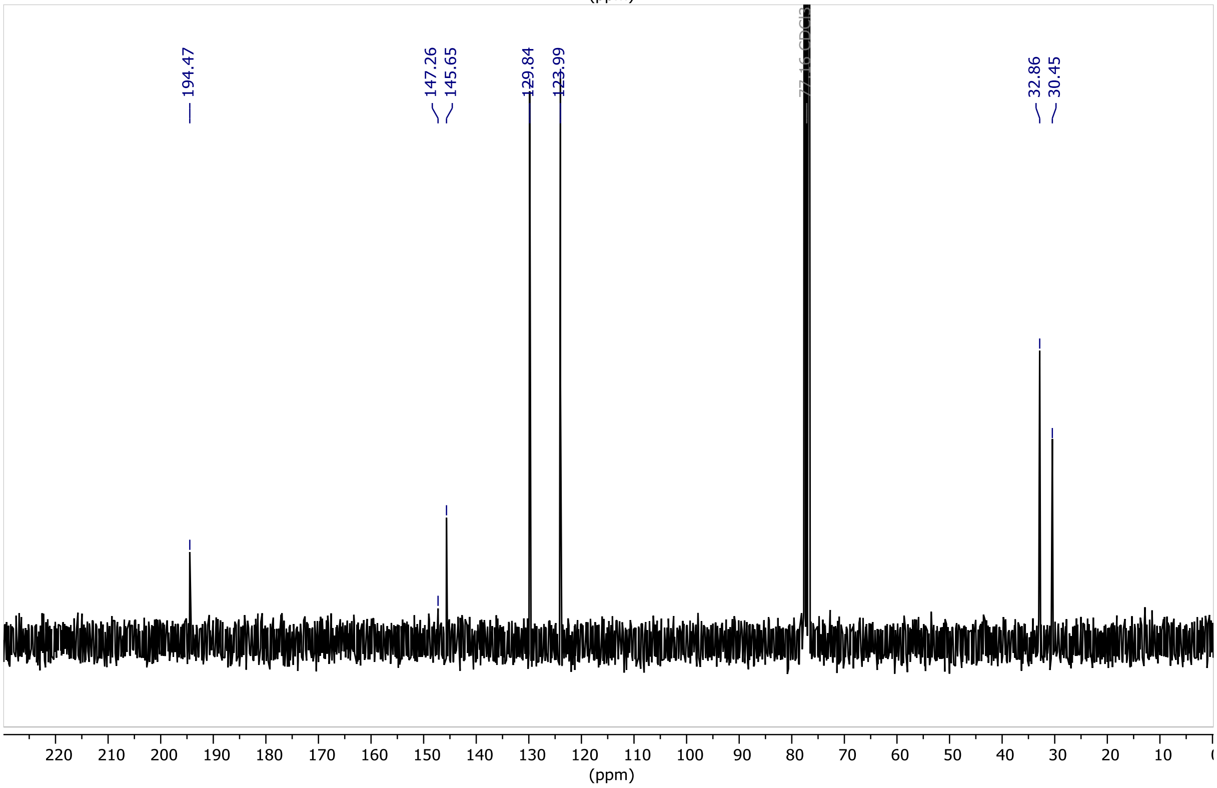

(4-Nitrophenyl)methanethiol (**4**)

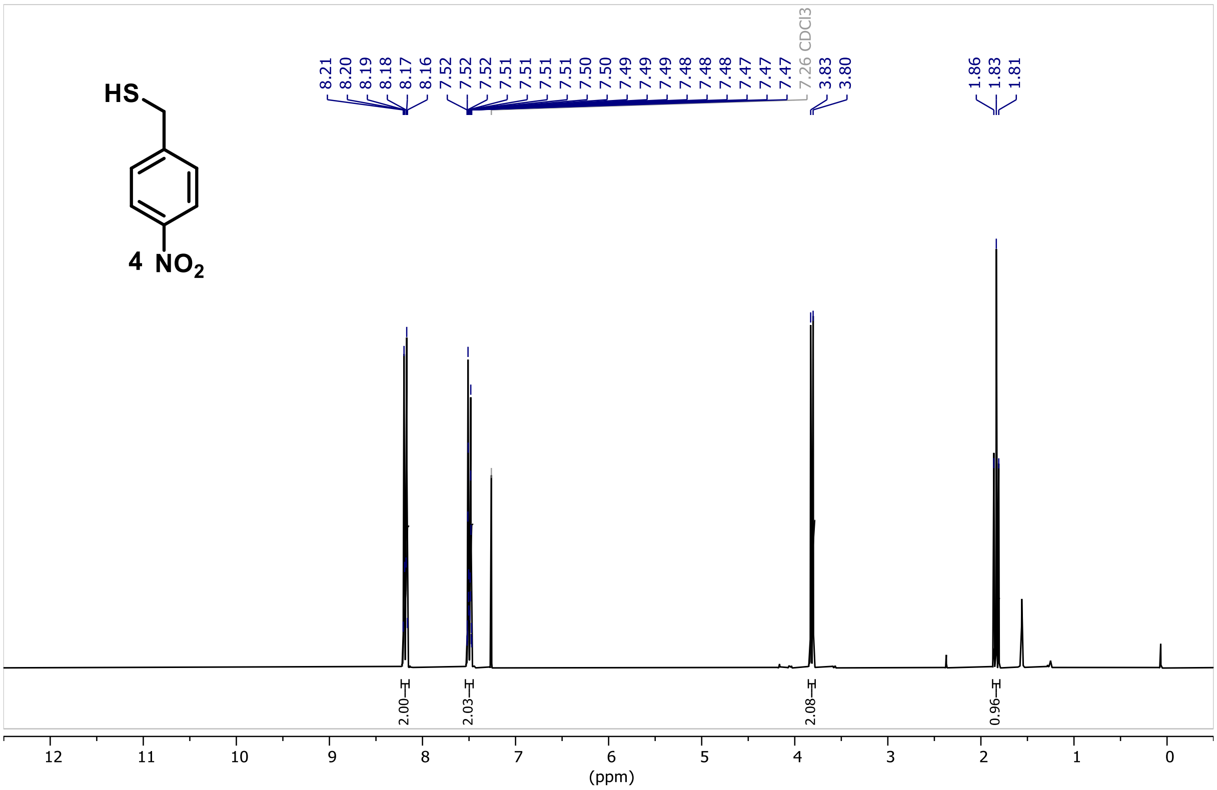

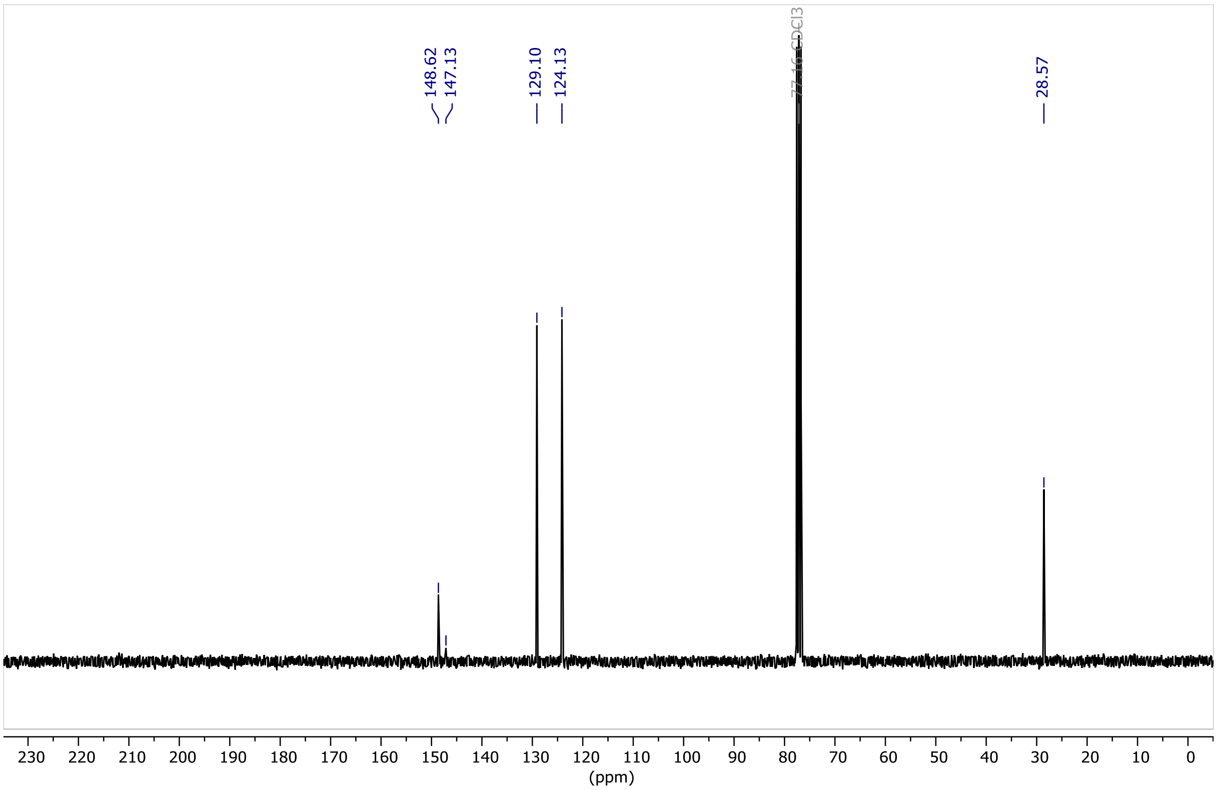

2-(4-Azidophenyl)ethan-1-ol (**6**)

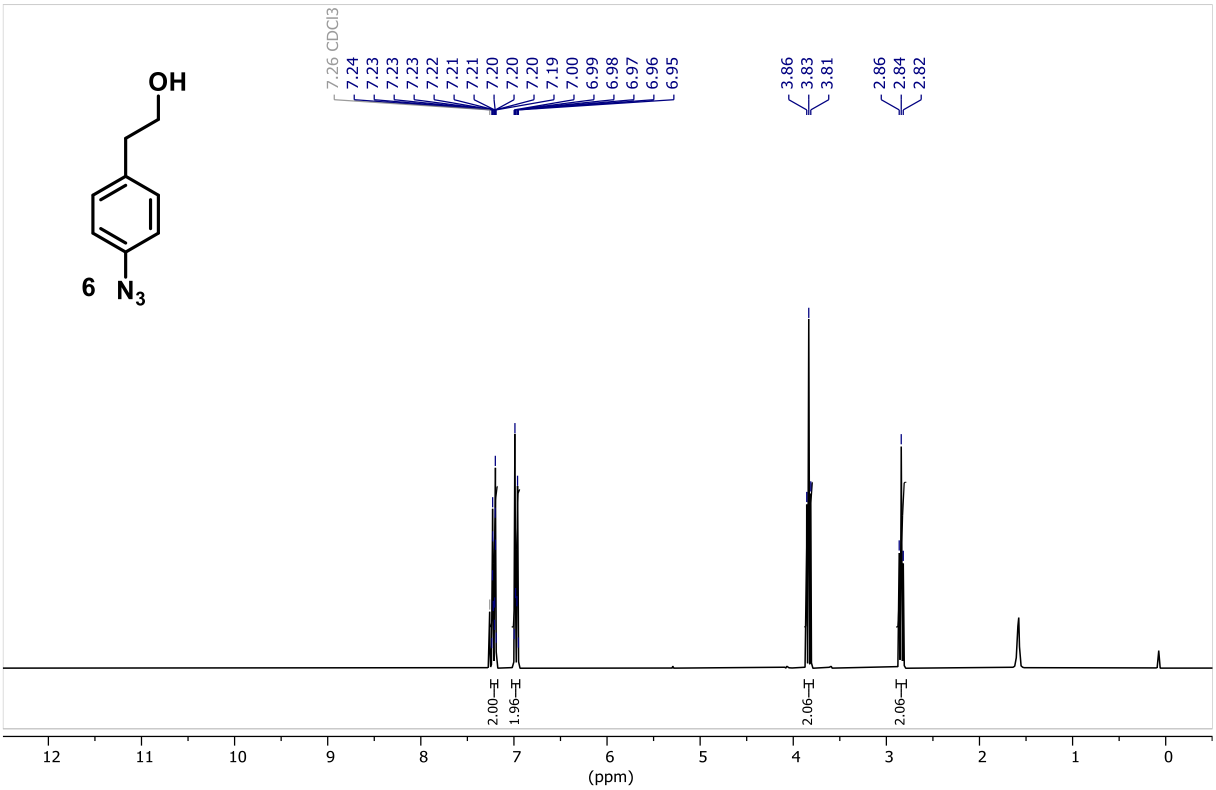

2-(4-Azidophenyl)acetaldehyde (**7**)

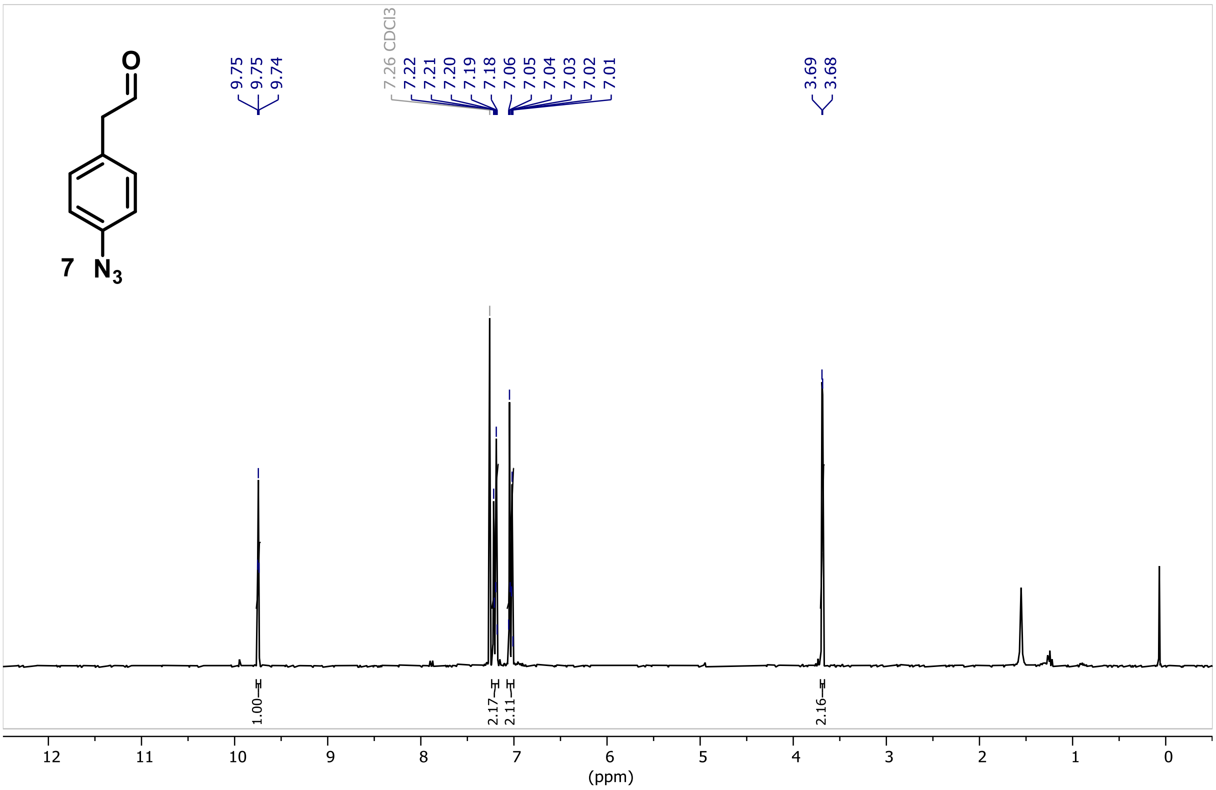

2-(4-Azidophenyl)acetaldehyde oxime (**8**)

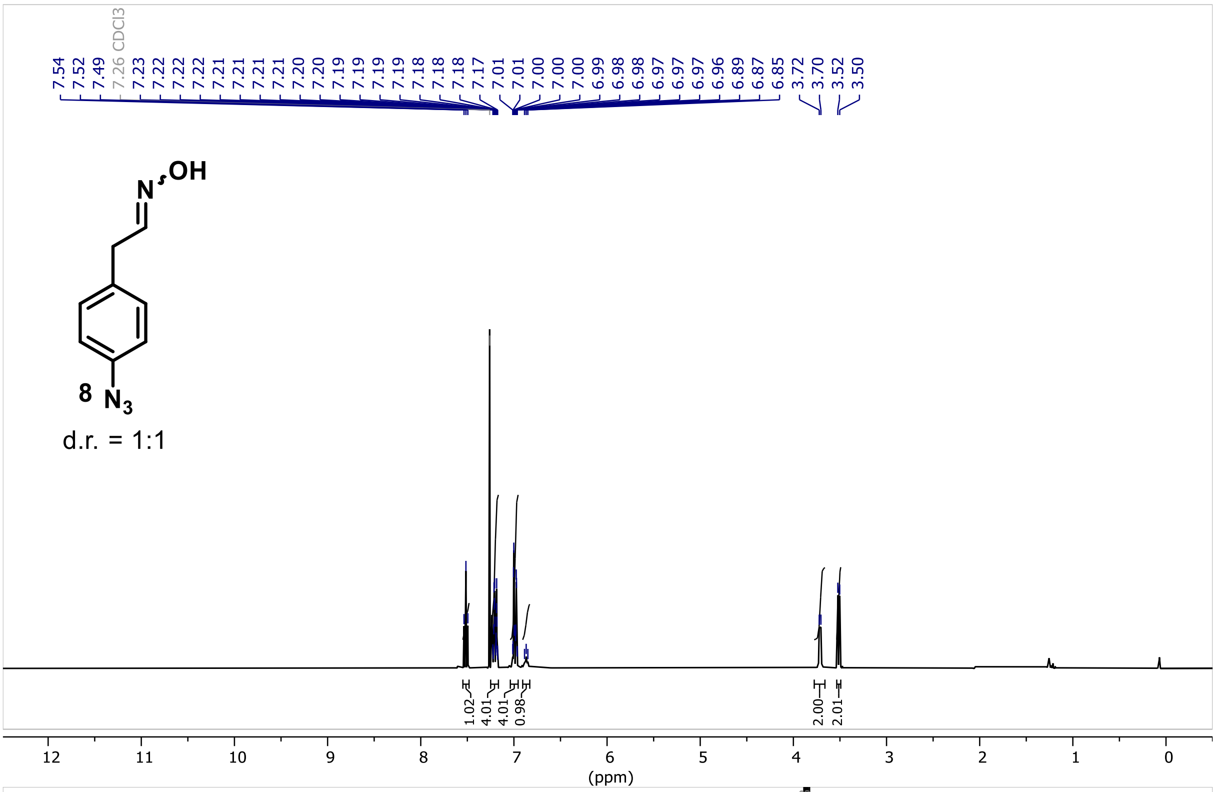

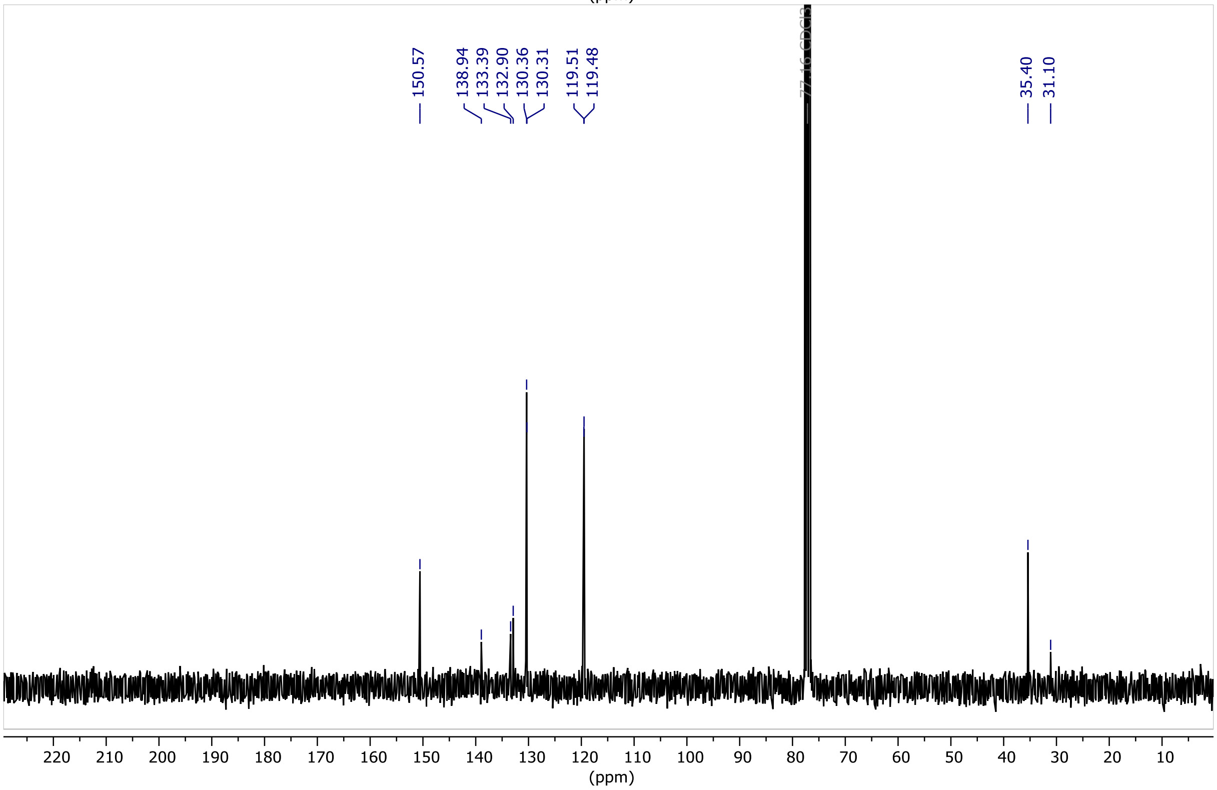

4-Nitrobenzyl (*Z*)-2-(4-azidophenyl)-N-hydroxyethanimidothioate (**9**)

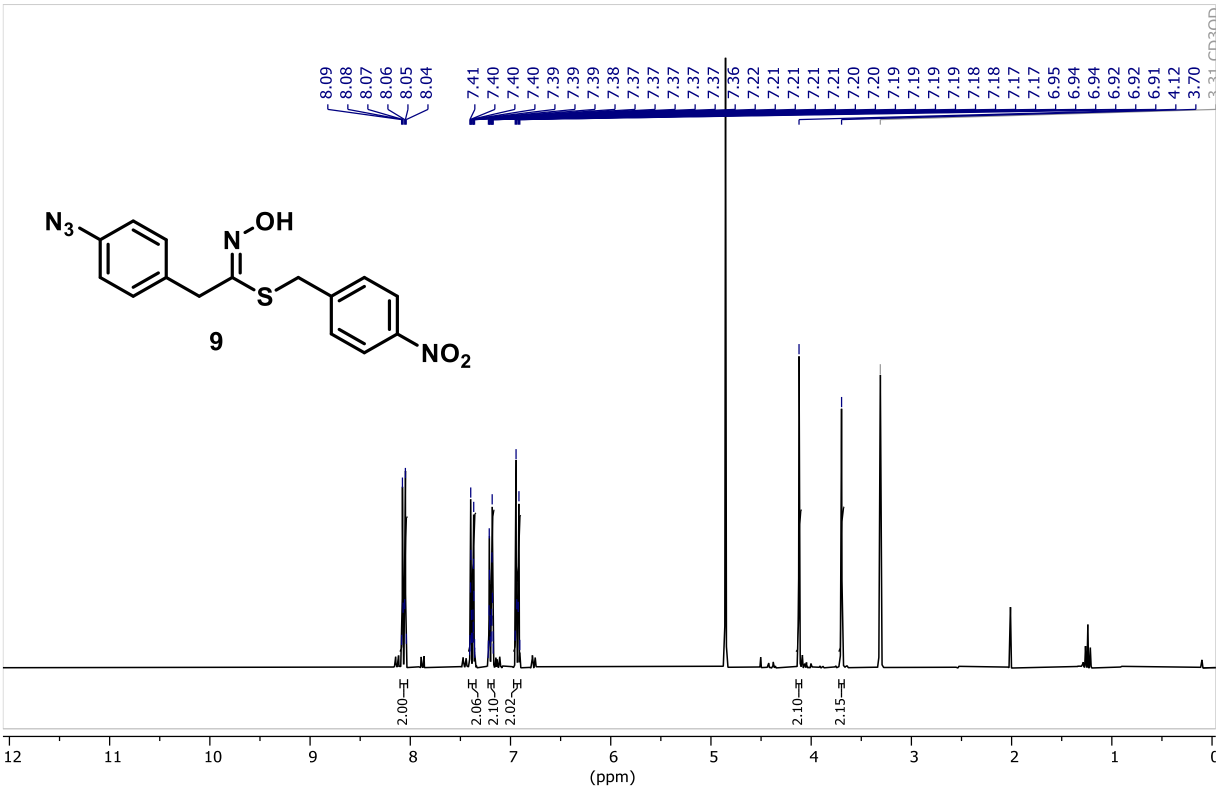

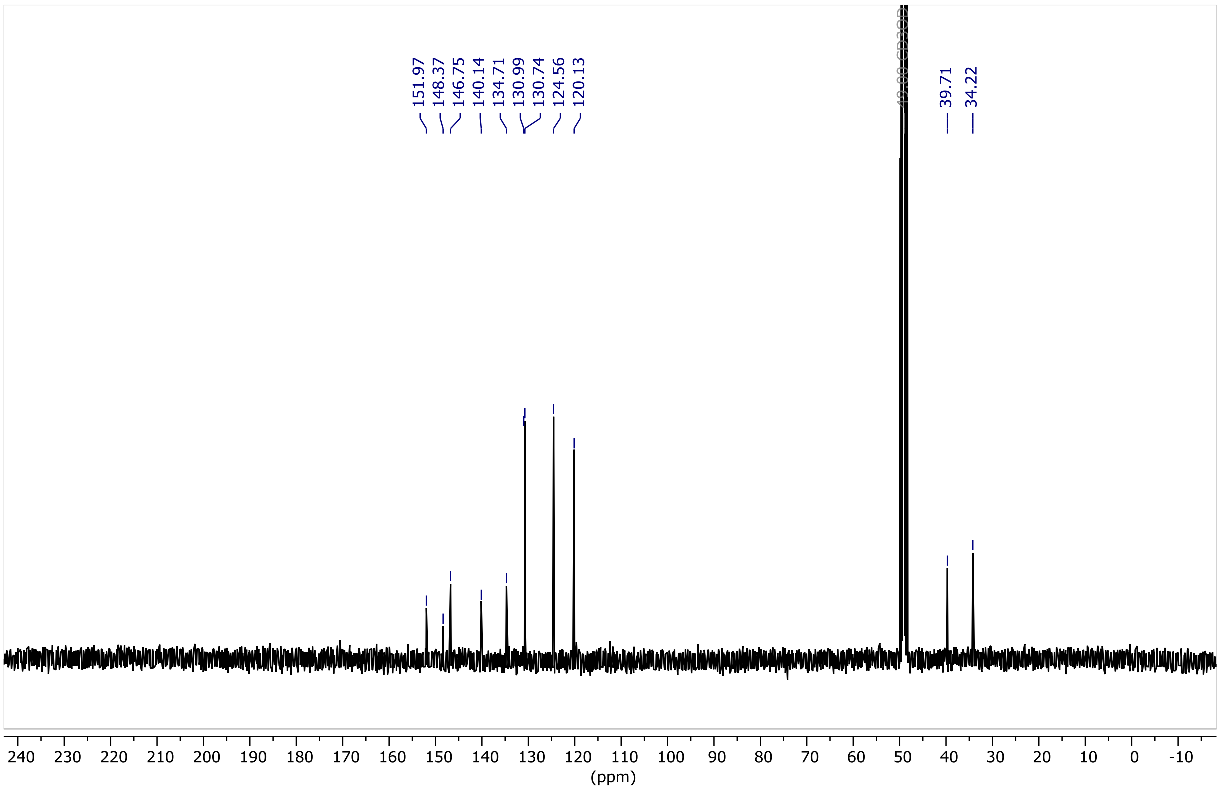

Potassium (*Z*)-(2-(4-azidophenyl)-1-((4-nitrobenzyl)thio)ethylidene)amino sulfate (***ps*GSL(NO$_2$)-N$_3$**)

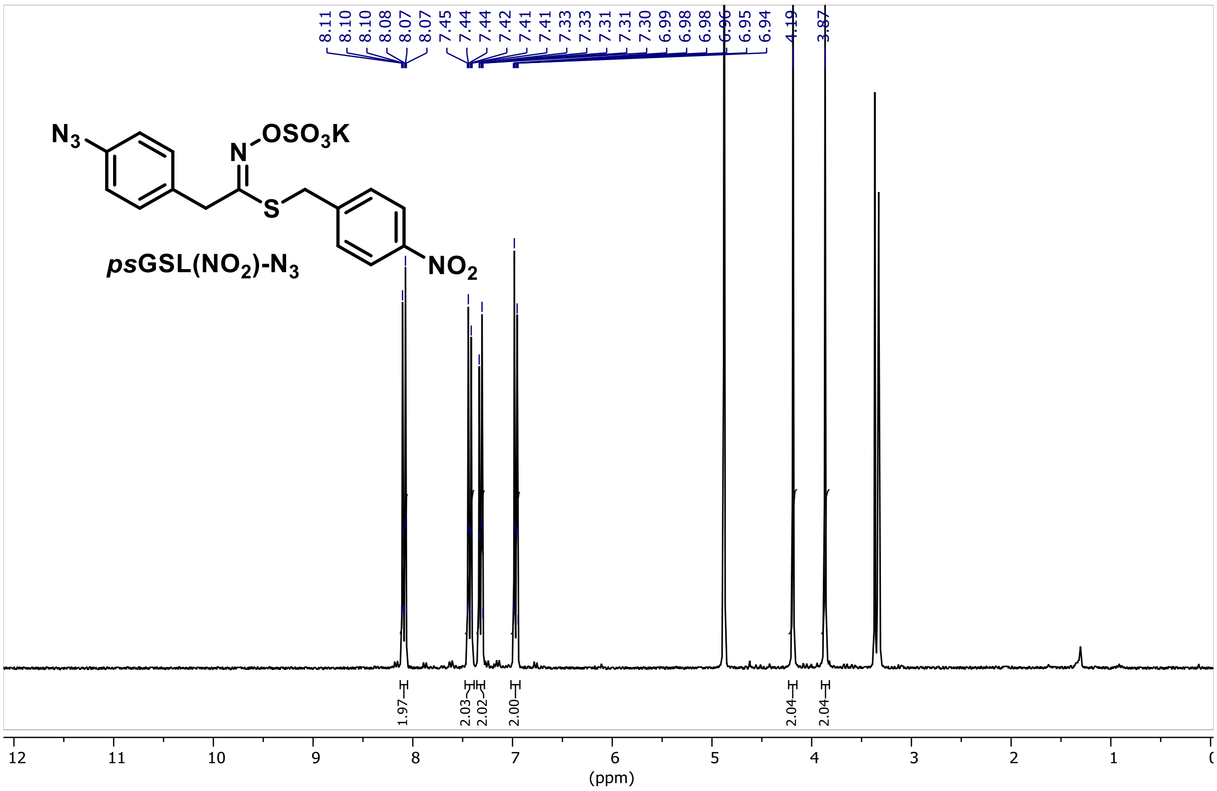

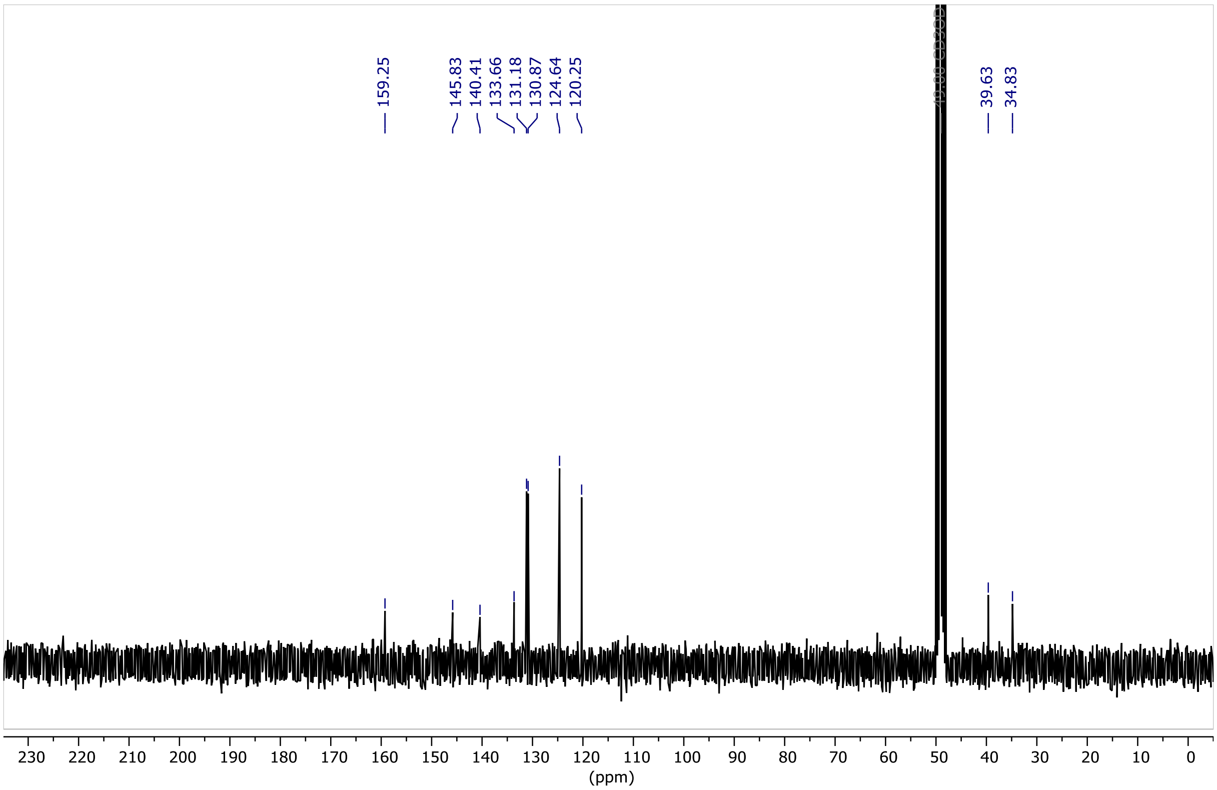

Potassium (*Z*)-(2-(4-(4-((4-(5,5-difluoro-1,3,7,9-tetramethyl-5*H*-4λ4,5λ4-dipyrrolo[1,2-c:2',1'-*f*][1,3,2]diazaborinin-10-yl)phenoxy)methyl)-1*H*-1,2,3-triazol-1-yl)phenyl)-1-((4-nitrobenzyl)thio)ethylidene)amino sulfate (***ps*GSL(NO₂)-BODIPY**)

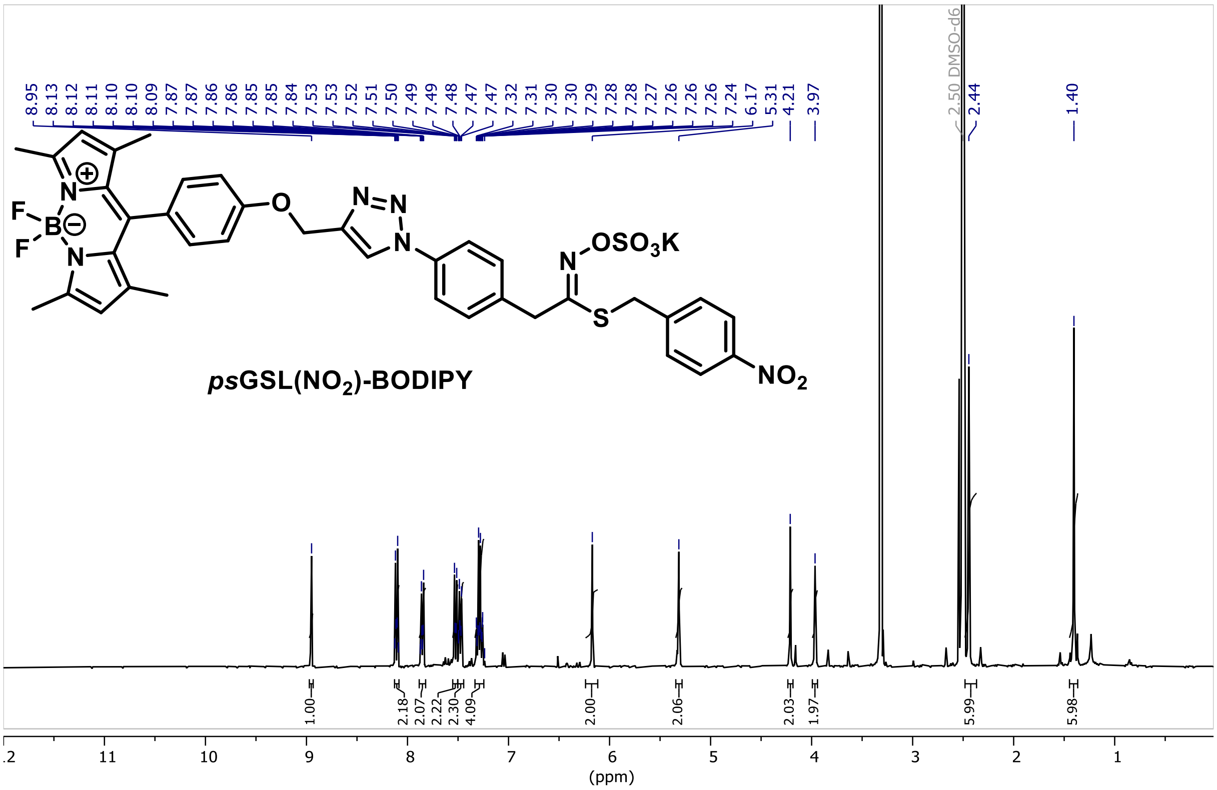

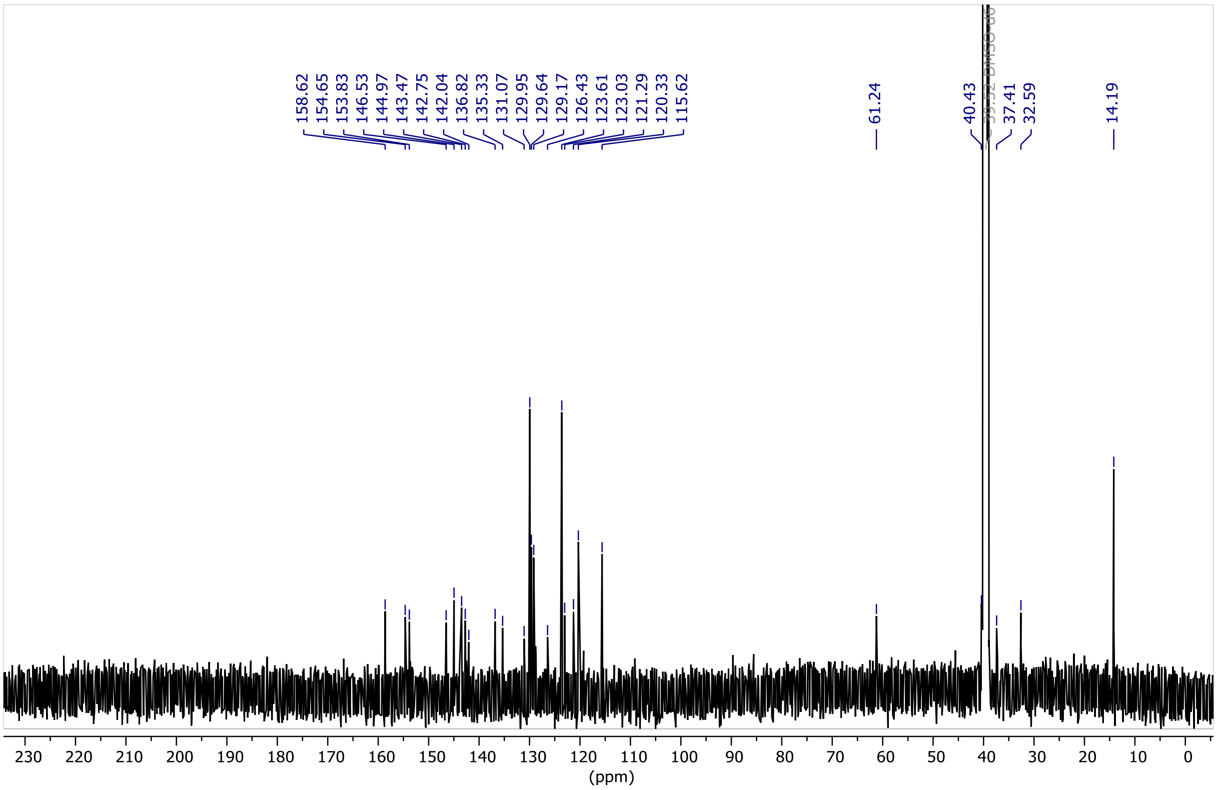

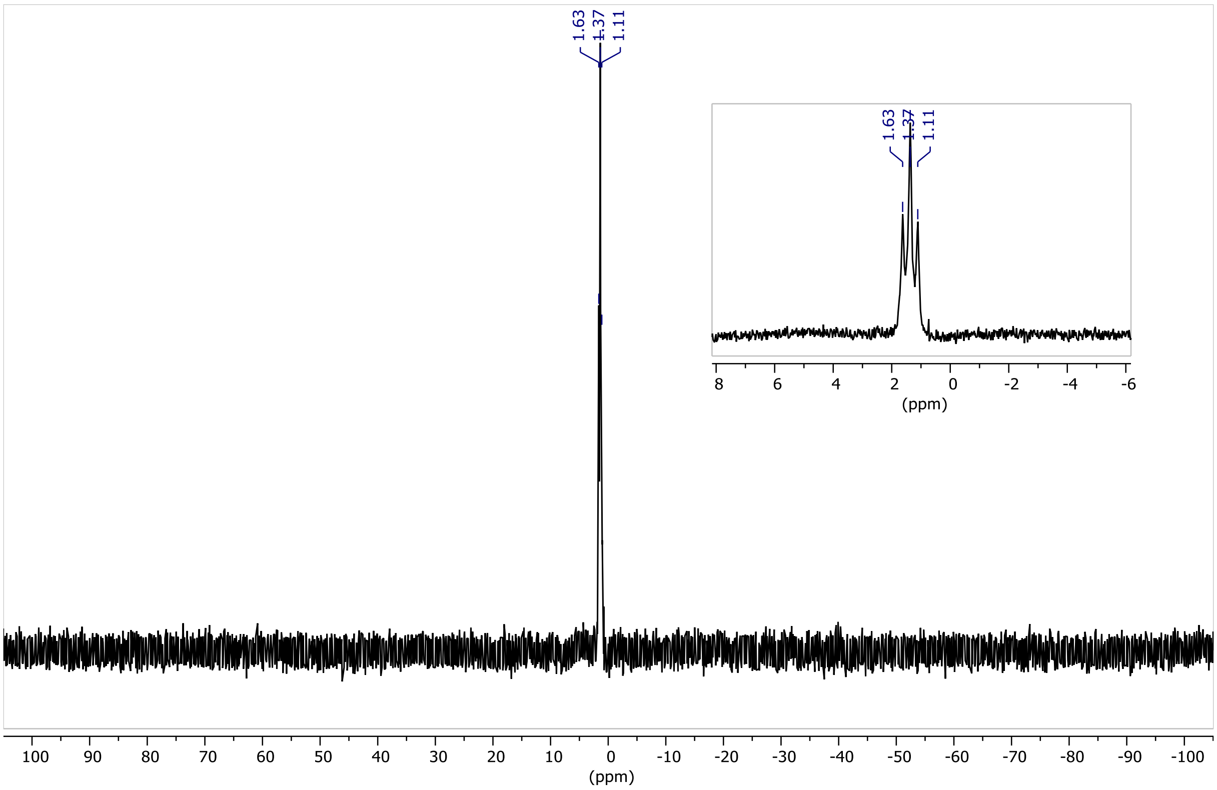
1.63
1.37
1.11
8 6 4 2 0 -2 -4 -6
(ppm)
100 90 80 70 60 50 40 30 20 10 0 -10 -20 -30 -40 -50 -60 -70 -80 -90 -100
(ppm)

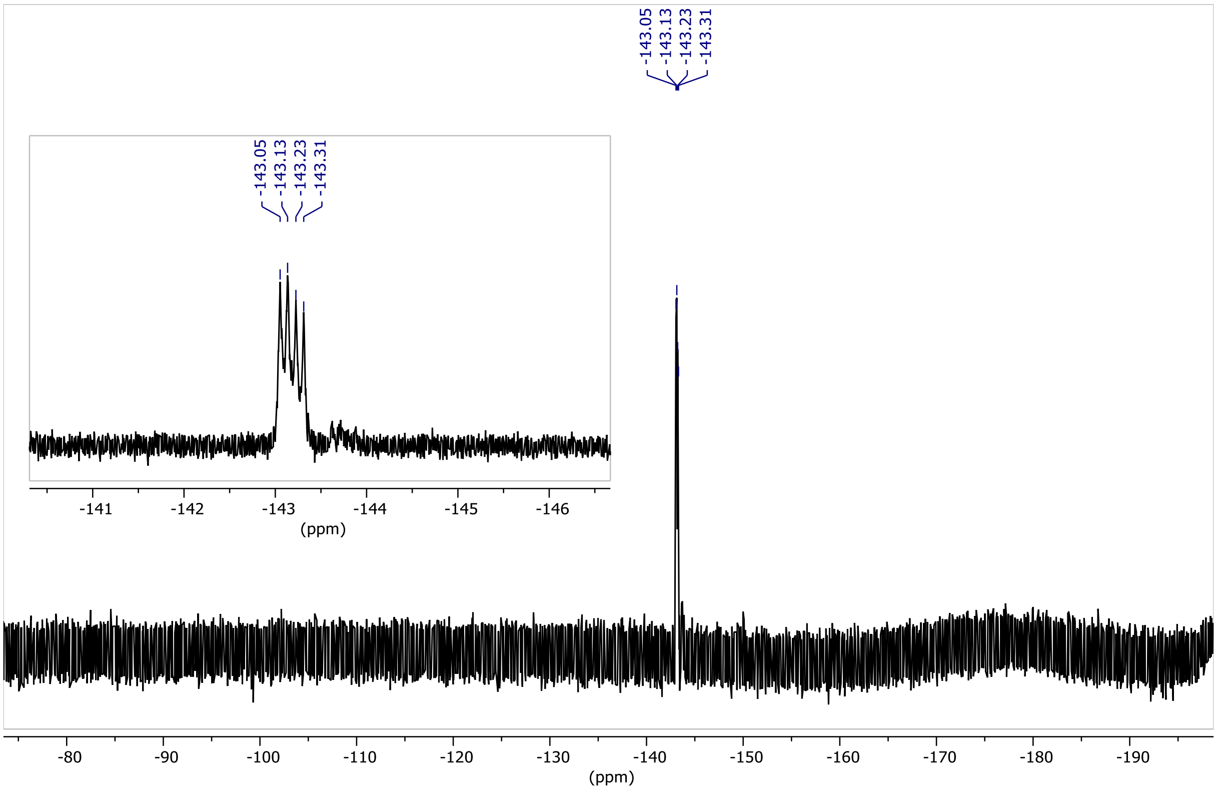
-143.05
-143.13
-143.23
-143.31
-141 -142 -143 -144 -145 -146
(ppm)
-80 -90 -100 -110 -120 -130 -140 -150 -160 -170 -180 -190
(ppm)

2-(2-(2-Azidoethoxy)ethoxy)ethan-1-ol (**12**)

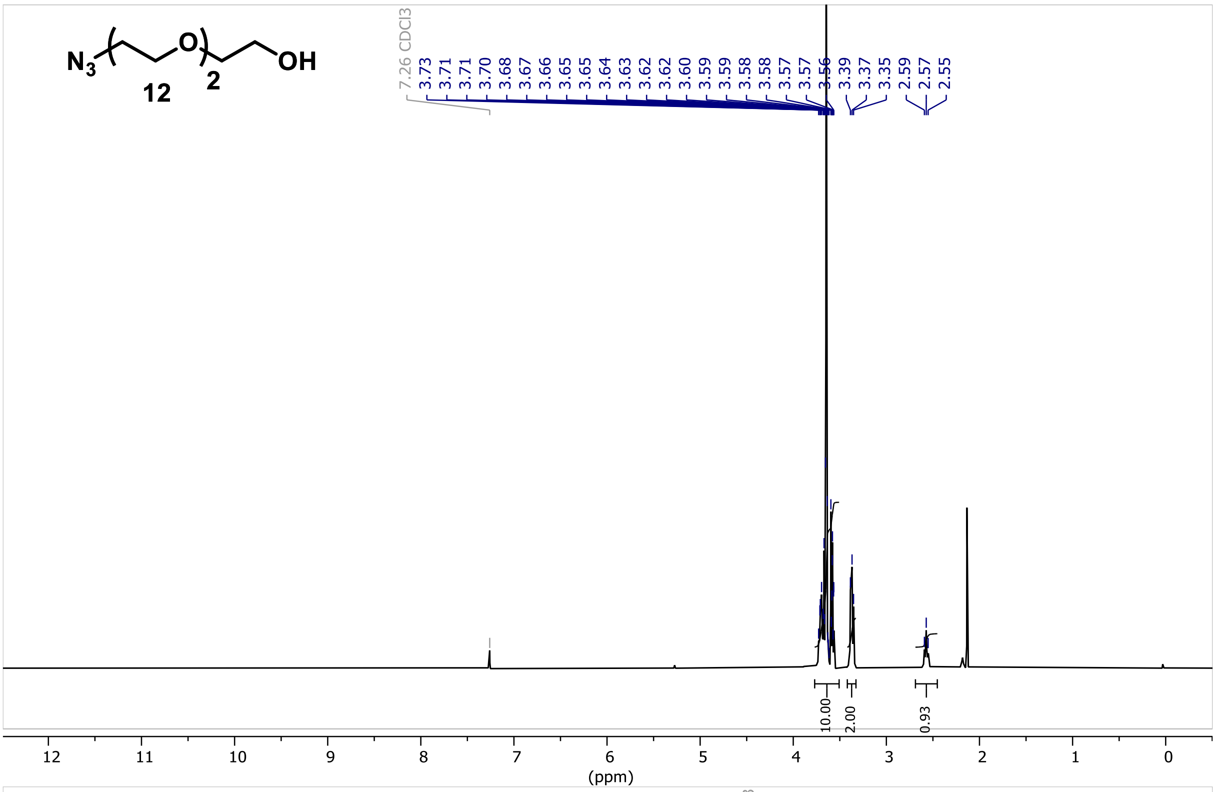

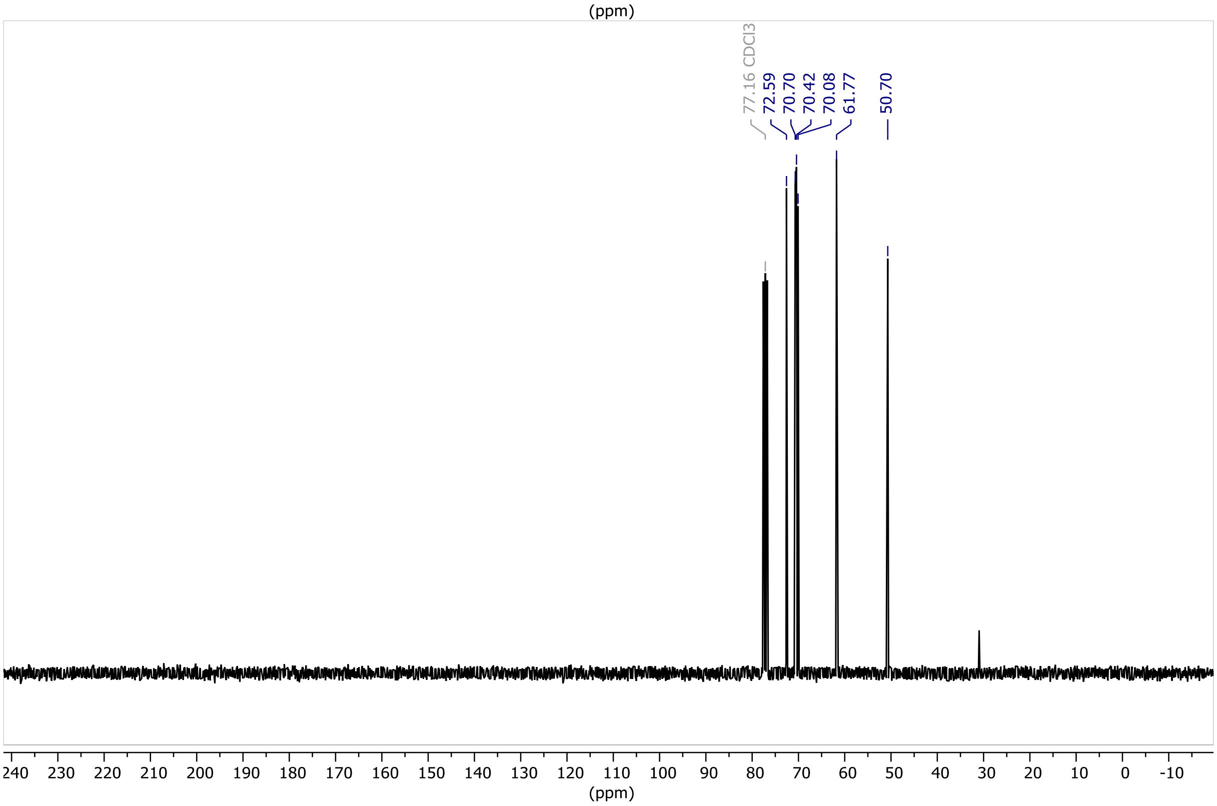

2-(2-(2-Azidoethoxy)ethoxy)acetic acid (**13**)

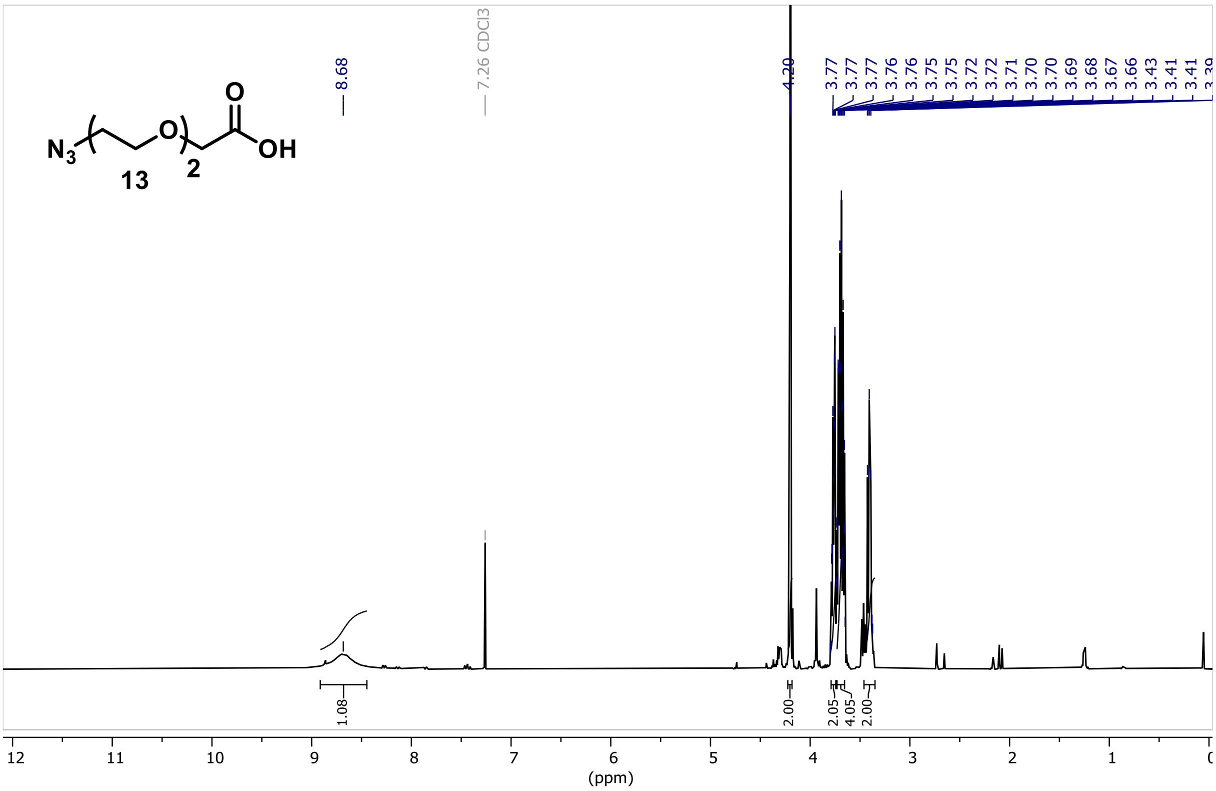

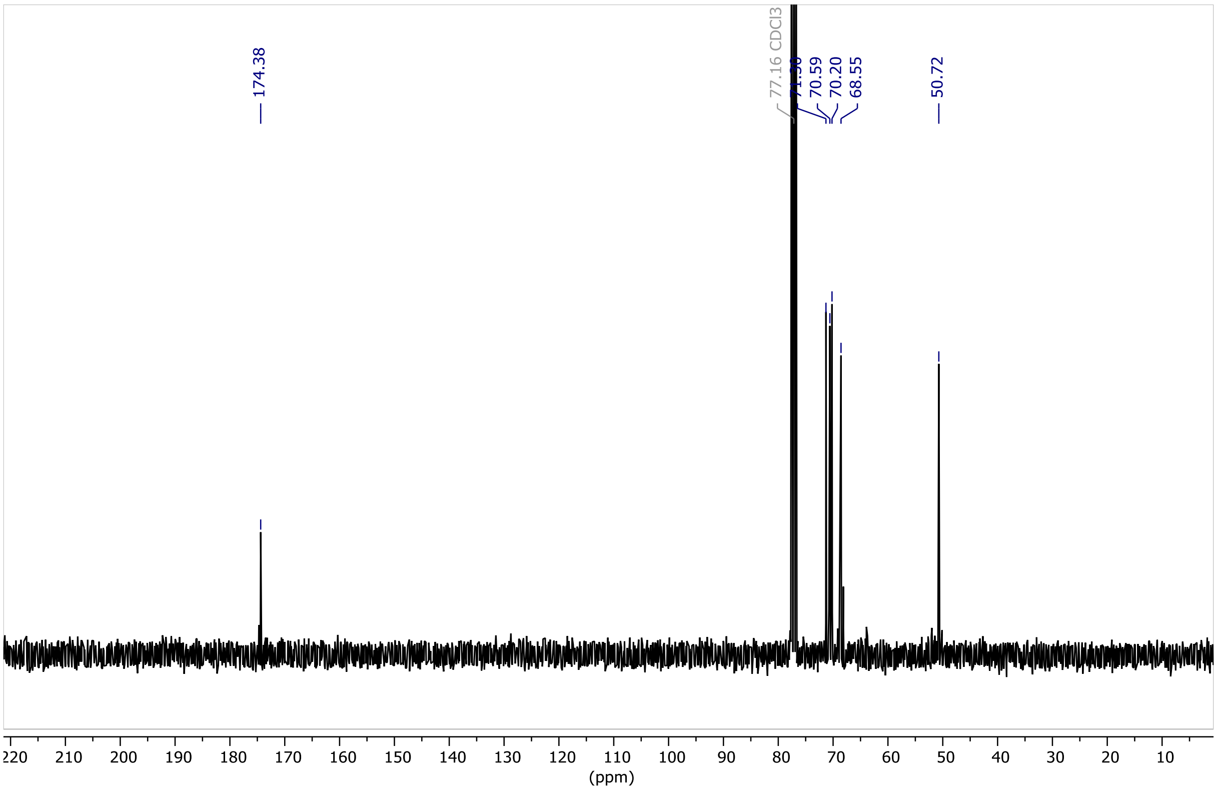

2-(2-(2-Azidoethoxy)ethoxy)-*N*-(4-(2-hydroxyethyl)phenyl)acetamide (**14**)

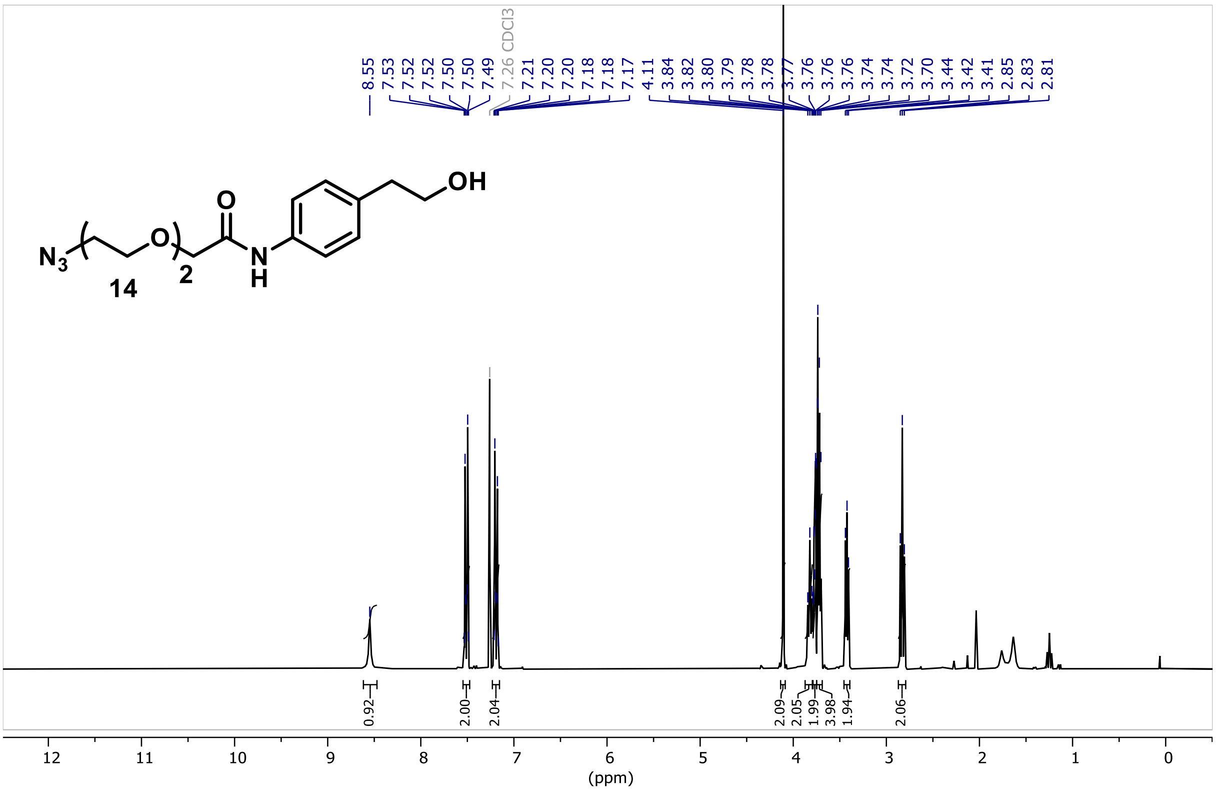

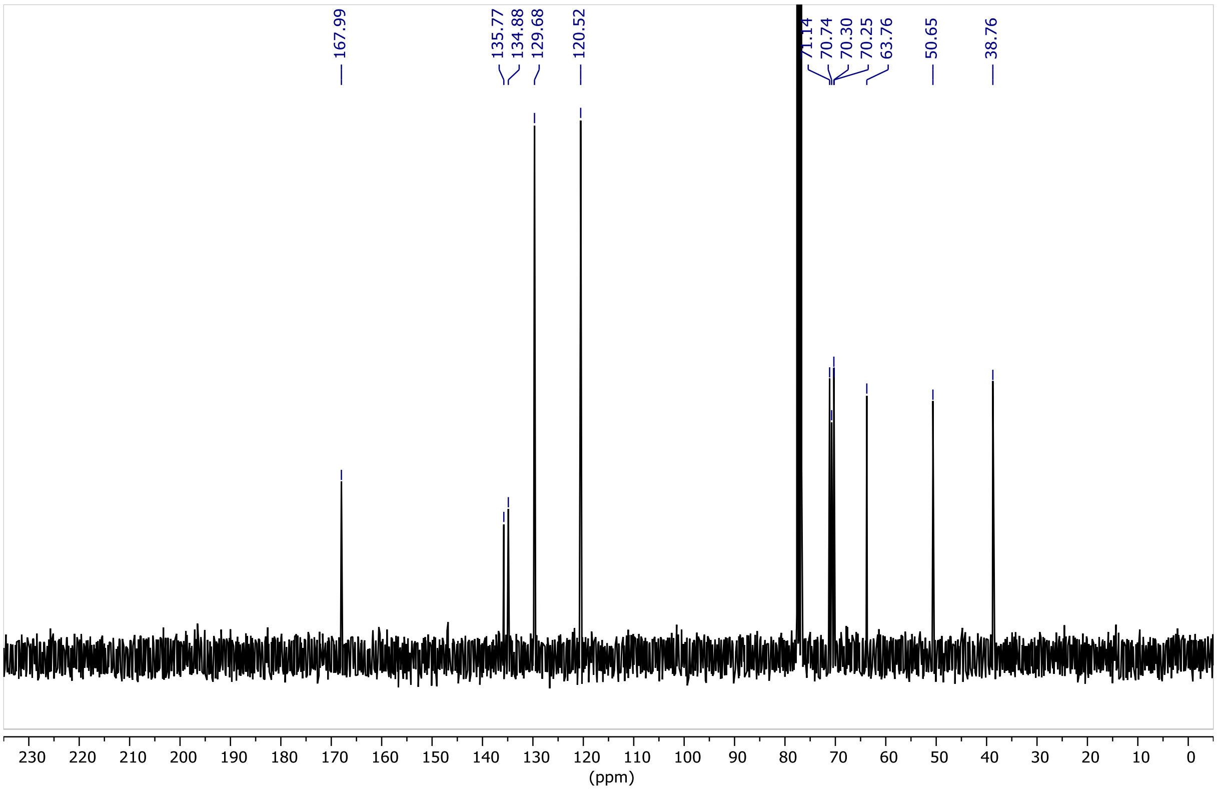

2-(2-(2-Azidoethoxy)ethoxy)-*N*-(4-(2-oxoethyl)phenyl)acetamide (**15**)

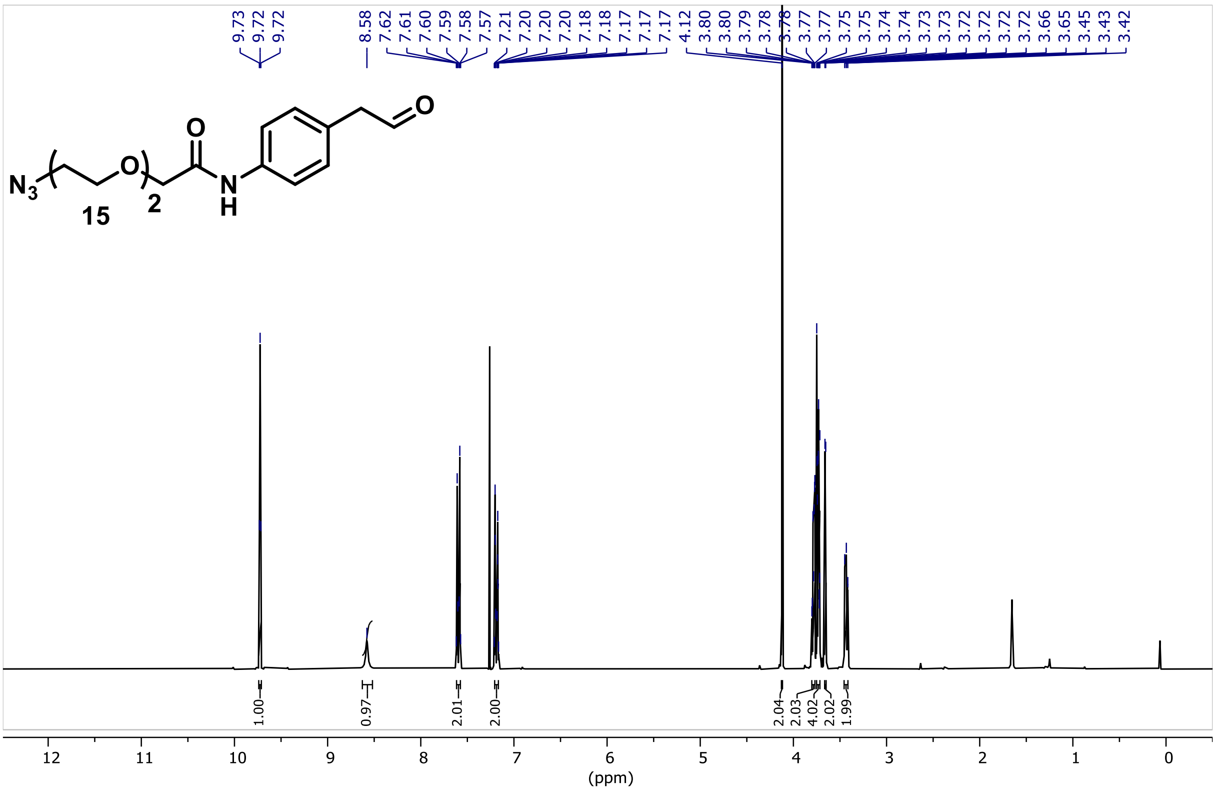

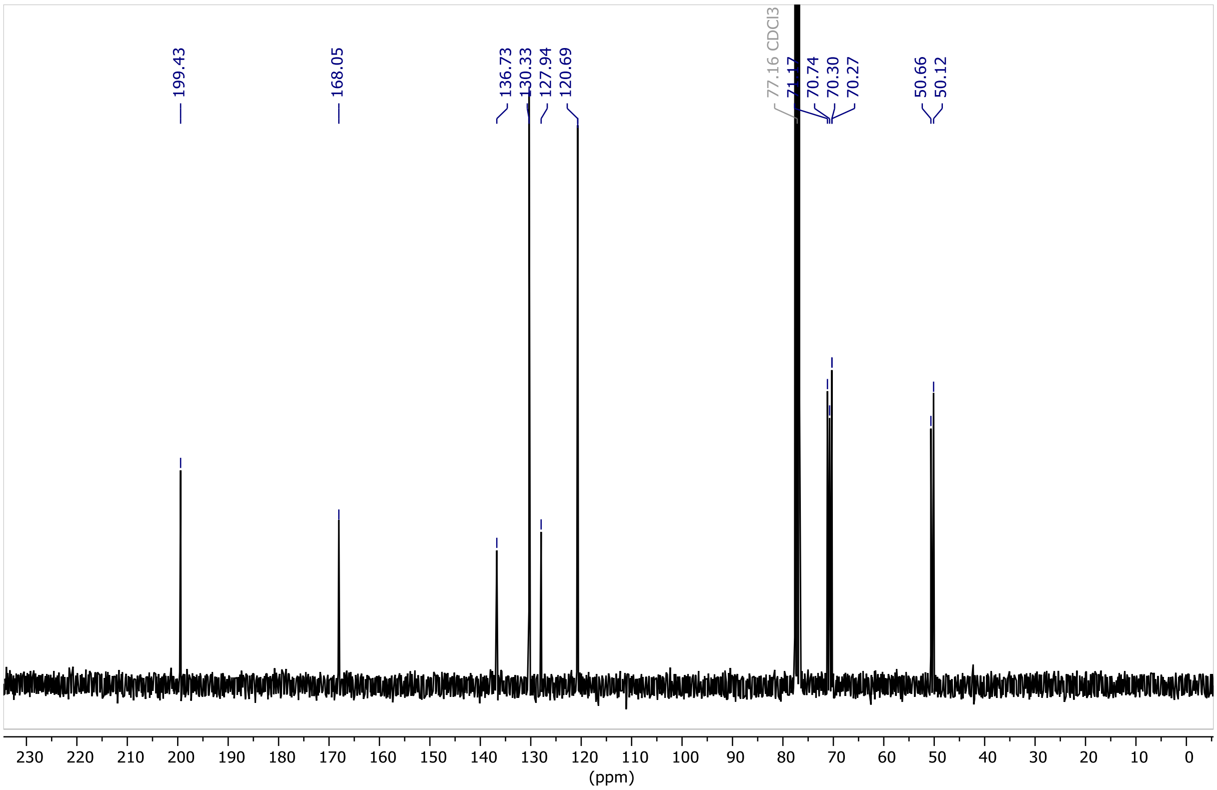

2-(2-(2-Azidoethoxy)ethoxy)-*N*-(4-(2-(hydroxyimino)ethyl)phenyl)acetamide (**16**)

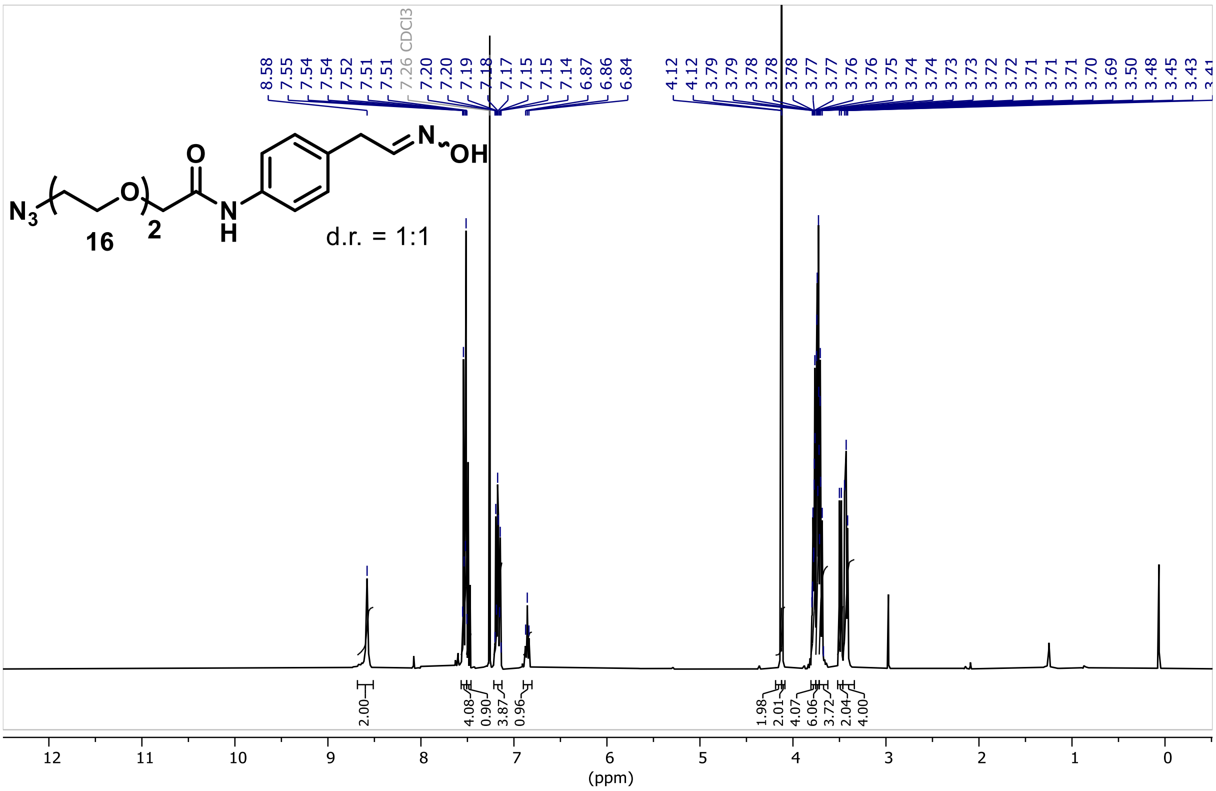

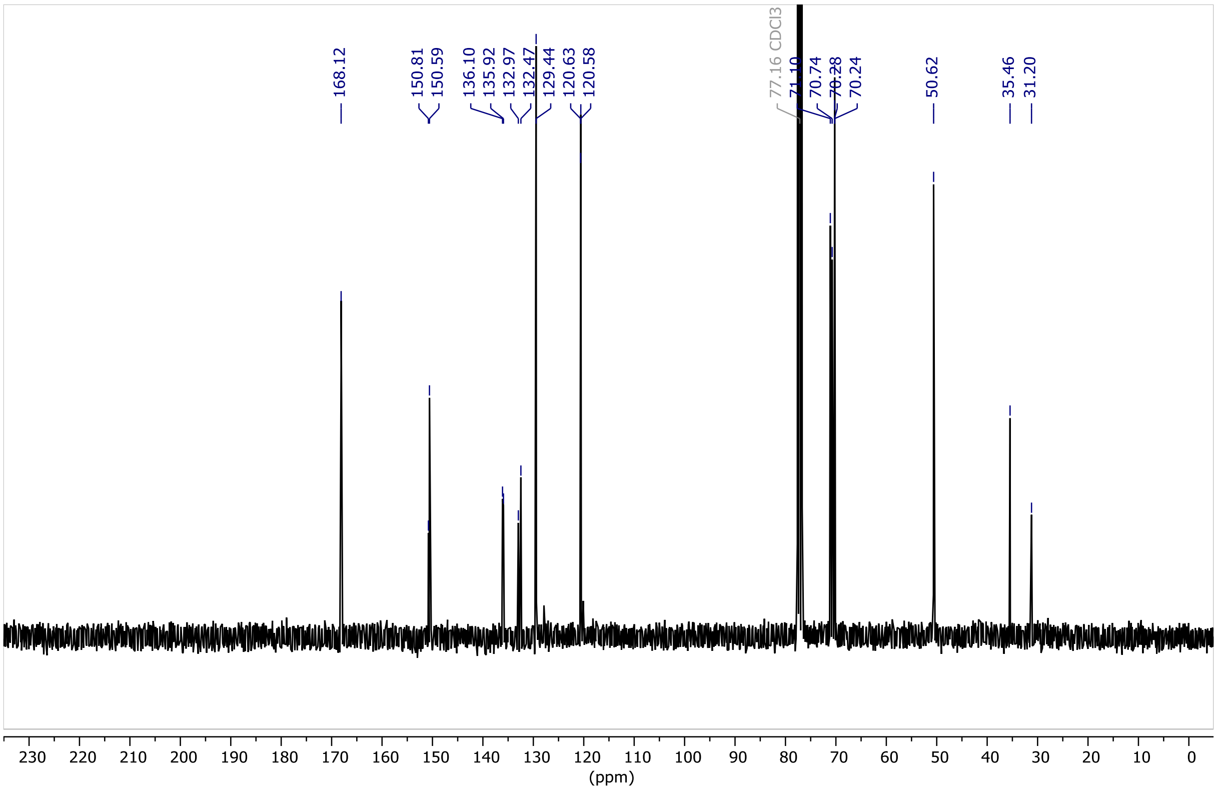

4-Nitrobenzyl (*Z*)-2-(4-(2-(2-(2-azidoethoxy)ethoxy)acetamido)phenyl)-*N*-hydroxyethanimido-thioate (**17**)

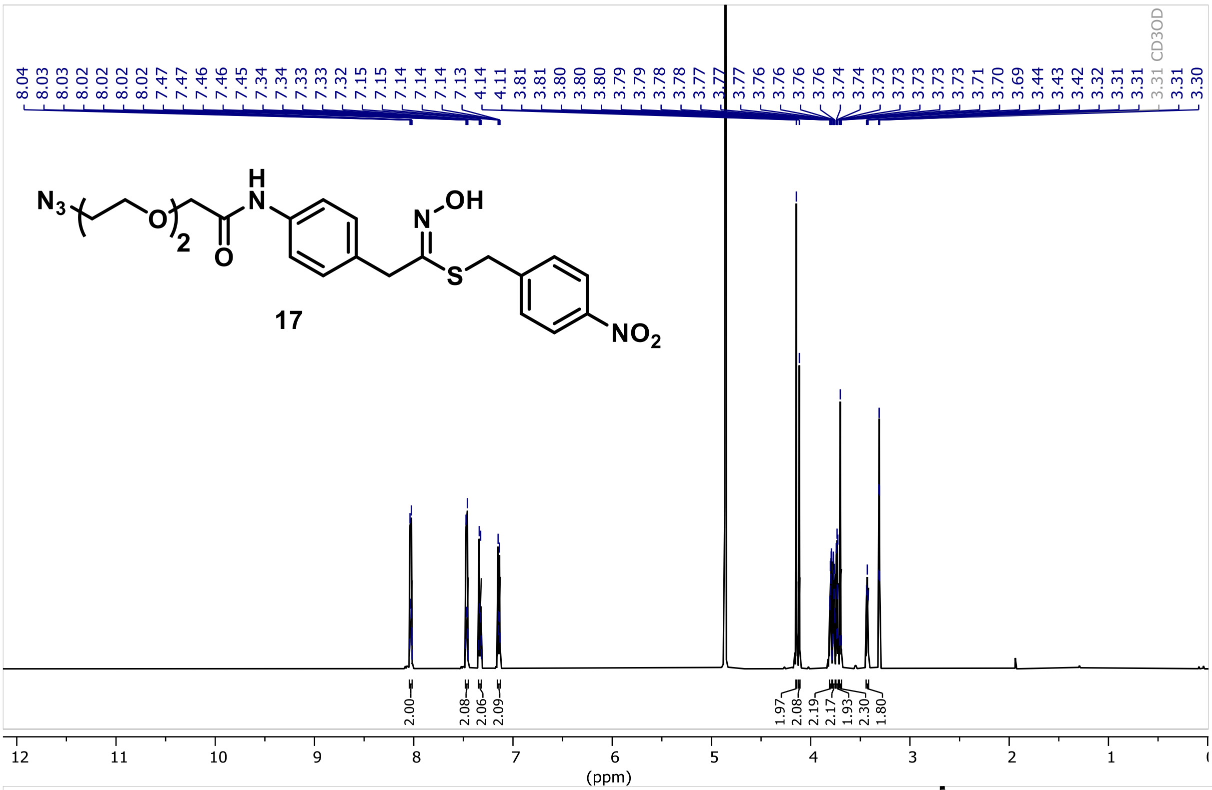

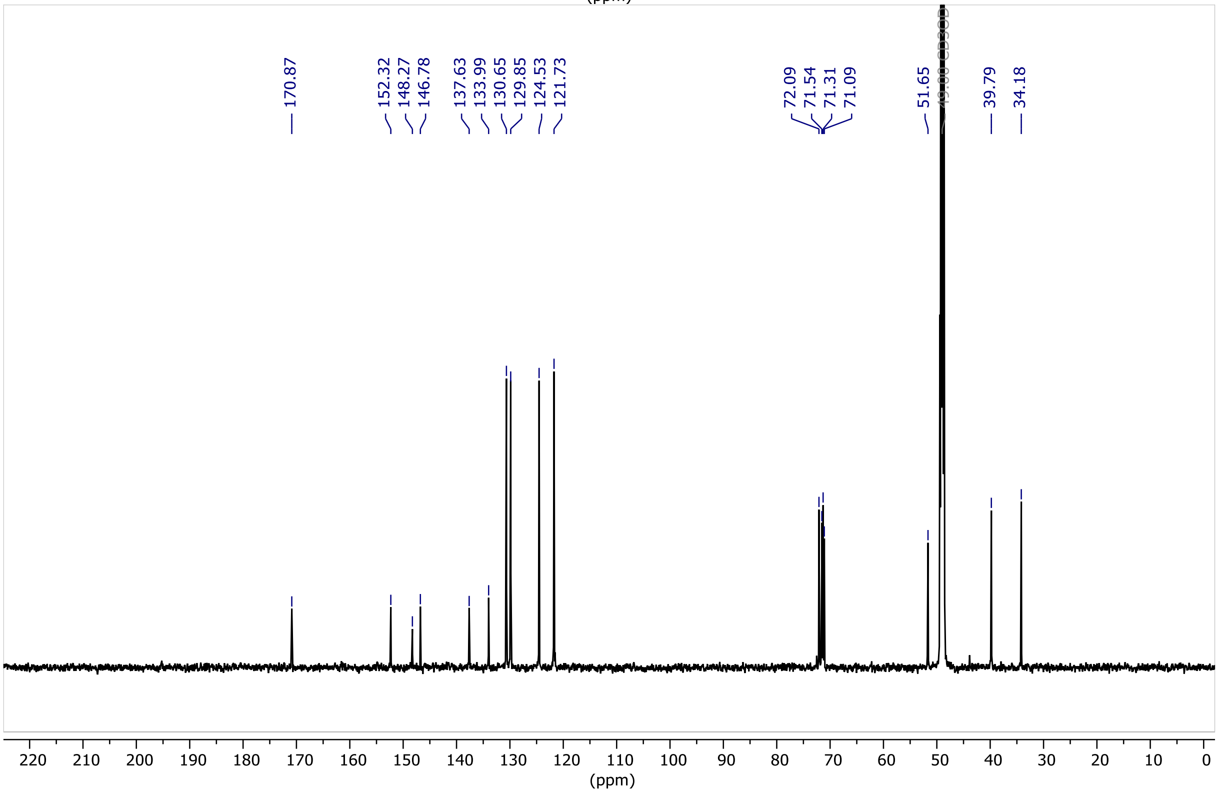

Potassium (*Z*)-(2-(4-(2-(2-(2-azidoethoxy)ethoxy)acetamido)phenyl)-1-((4-nitrobenzyl)thio)-ethylidene)amino sulfate (***ps*GSL$_{PEG}$(NO$_2$)-N$_3$**)

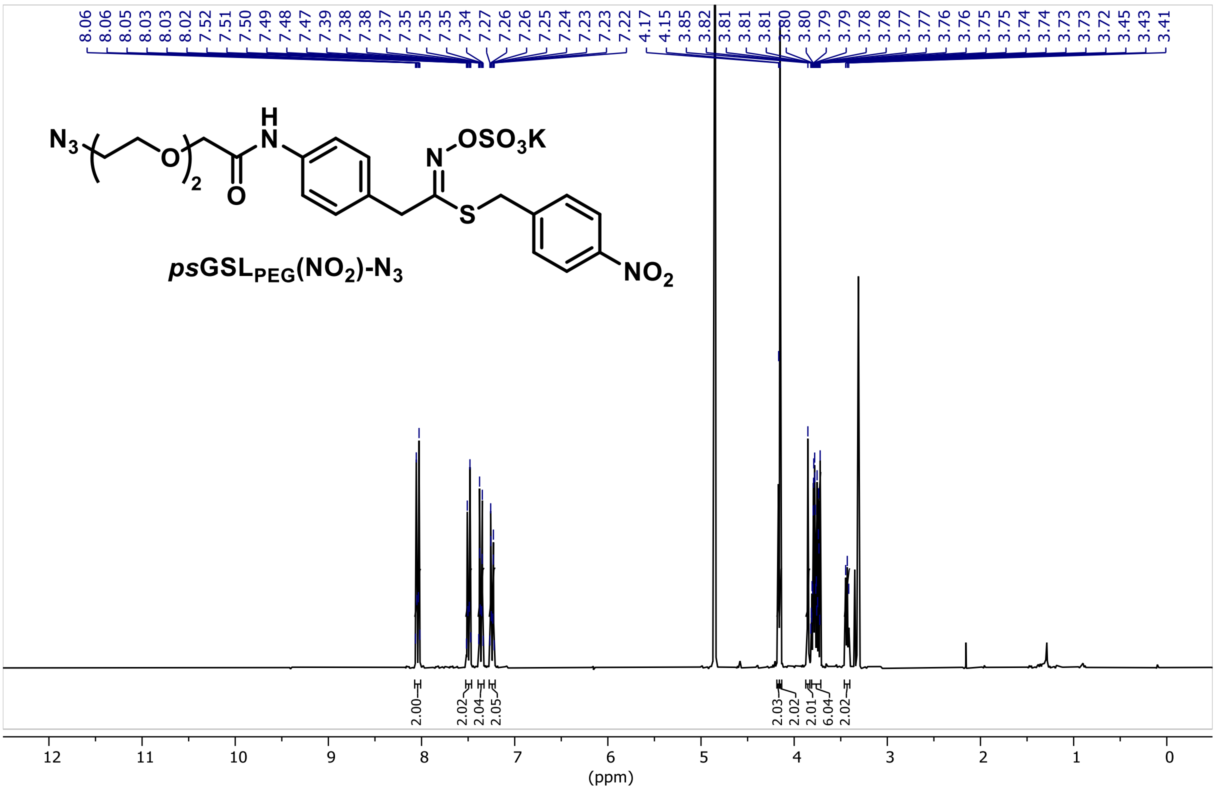

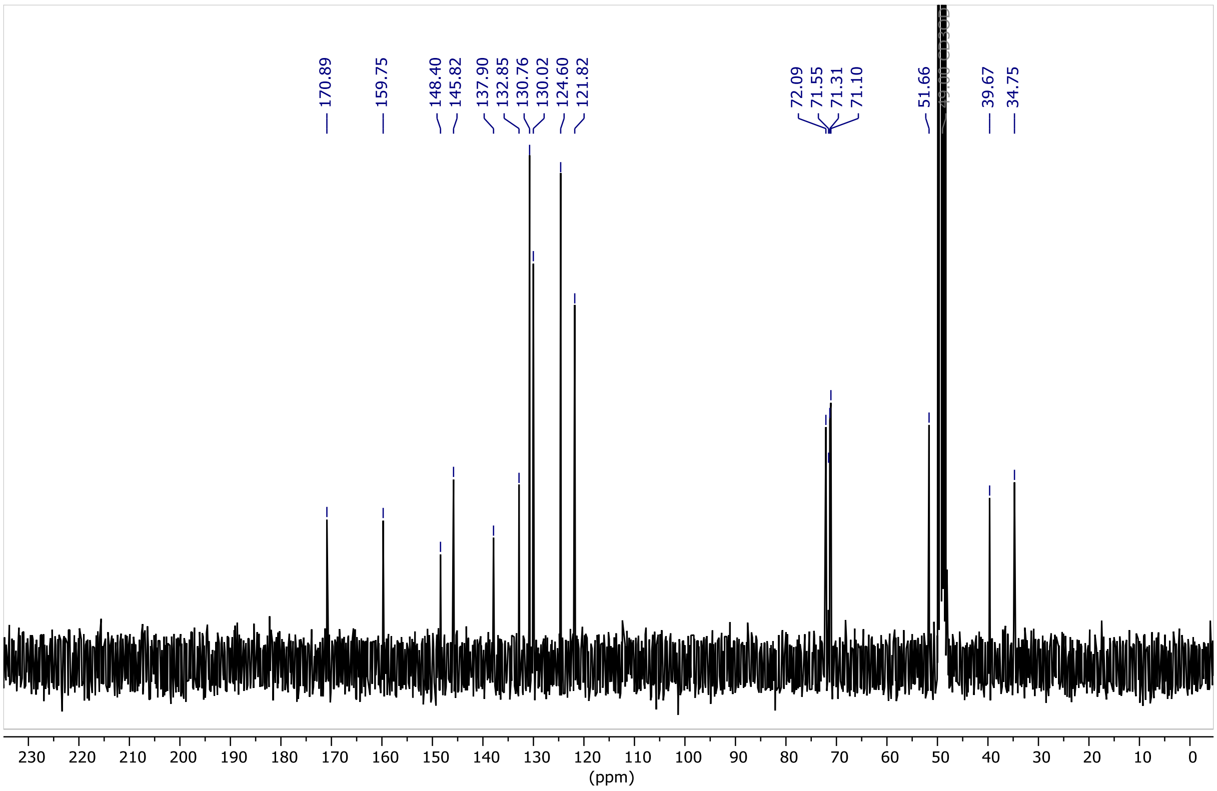

Potassium (*Z*)-(2-(4-(2-(2-(2-(4-((4-(5,5-difluoro-1,3,7,9-tetramethyl-5*H*-4λ⁴,5λ⁴-dipyrrolo[1,2-*c*:2',1'-*f*][1,3,2]diazaborinin-10-yl)phenoxy)methyl)-1*H*-1,2,3-triazol-1-yl)ethoxy)ethoxy)acet-amido)phenyl)-1-((4-nitrobenzyl)thio)ethylidene)amino sulfate (***ps*GSL$_{PEG}$(NO$_2$)-BODIPY**)

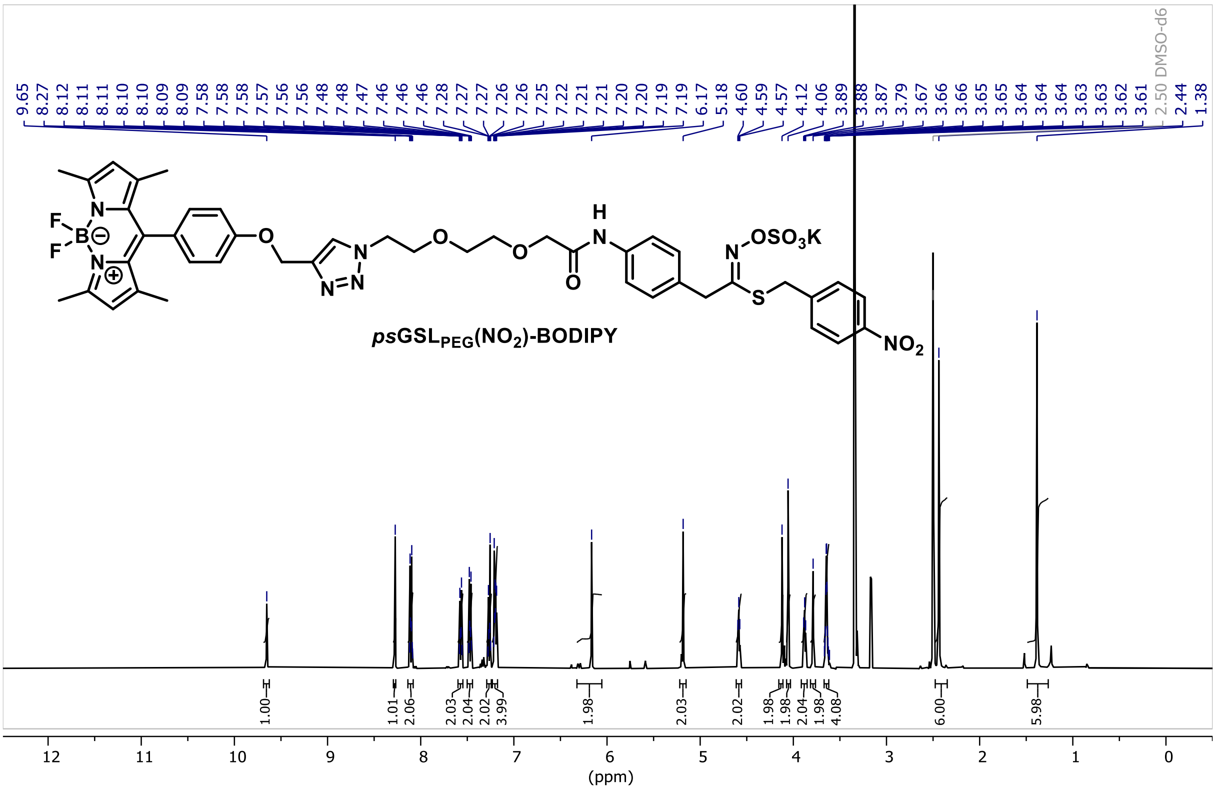

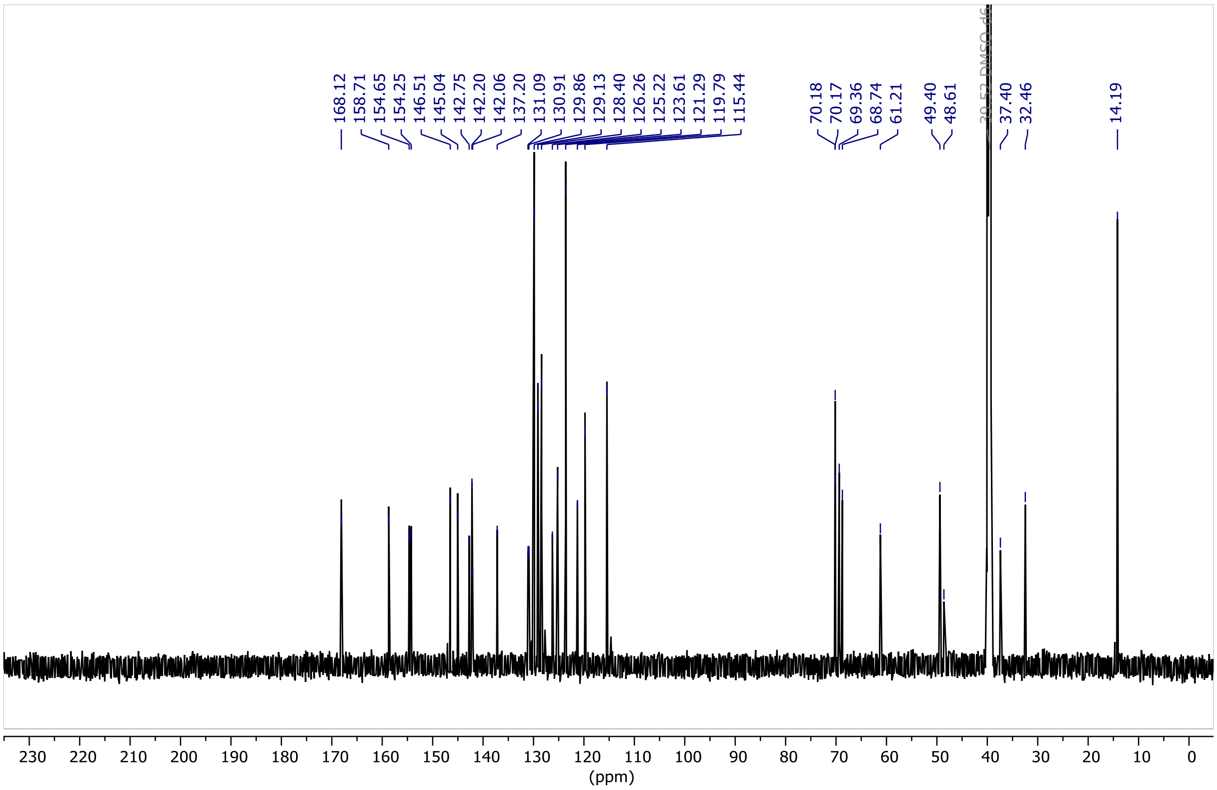

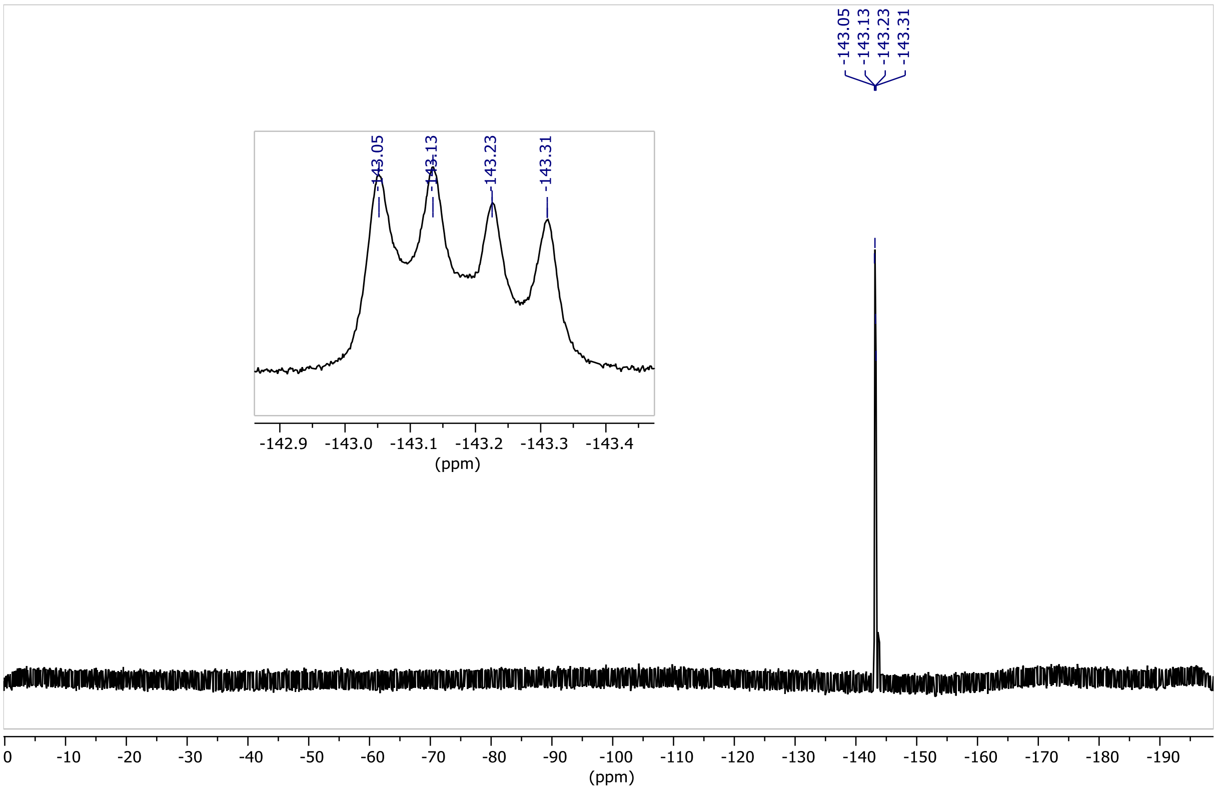
-143.05
-143.13
-143.23
-143.31
-142.9 -143.0 -143.1 -143.2 -143.3 -143.4
(ppm)
0 -10 -20 -30 -40 -50 -60 -70 -80 -90 -100 -110 -120 -130 -140 -150 -160 -170 -180 -190
(ppm)

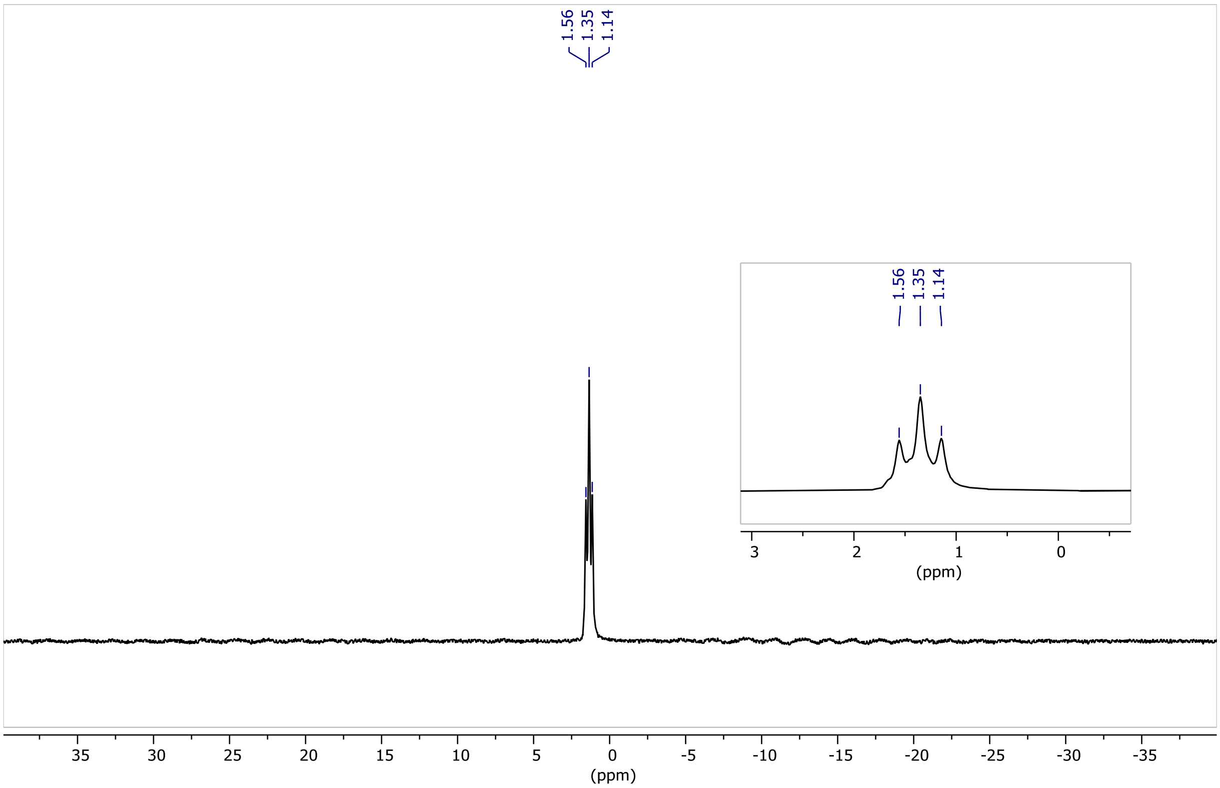
1.56
1.35
1.14
3 2 1 0
(ppm)
35 30 25 20 15 10 5 0 -5 -10 -15 -20 -25 -30 -35
(ppm)

Potassium (*Z*)-(2-(4-(2-(2-(2-(4-(((5-(dimethylamino)naphthalene)-1-sulfonamido)methyl)-1*H*-1,2,3-triazol-1-yl)ethoxy)ethoxy)acetamido)phenyl)-1-((4-nitrobenzyl)thio)ethylidene)amino sulfate (***ps*GSL$_{PEG}$(NO$_2$)-DNSA**)

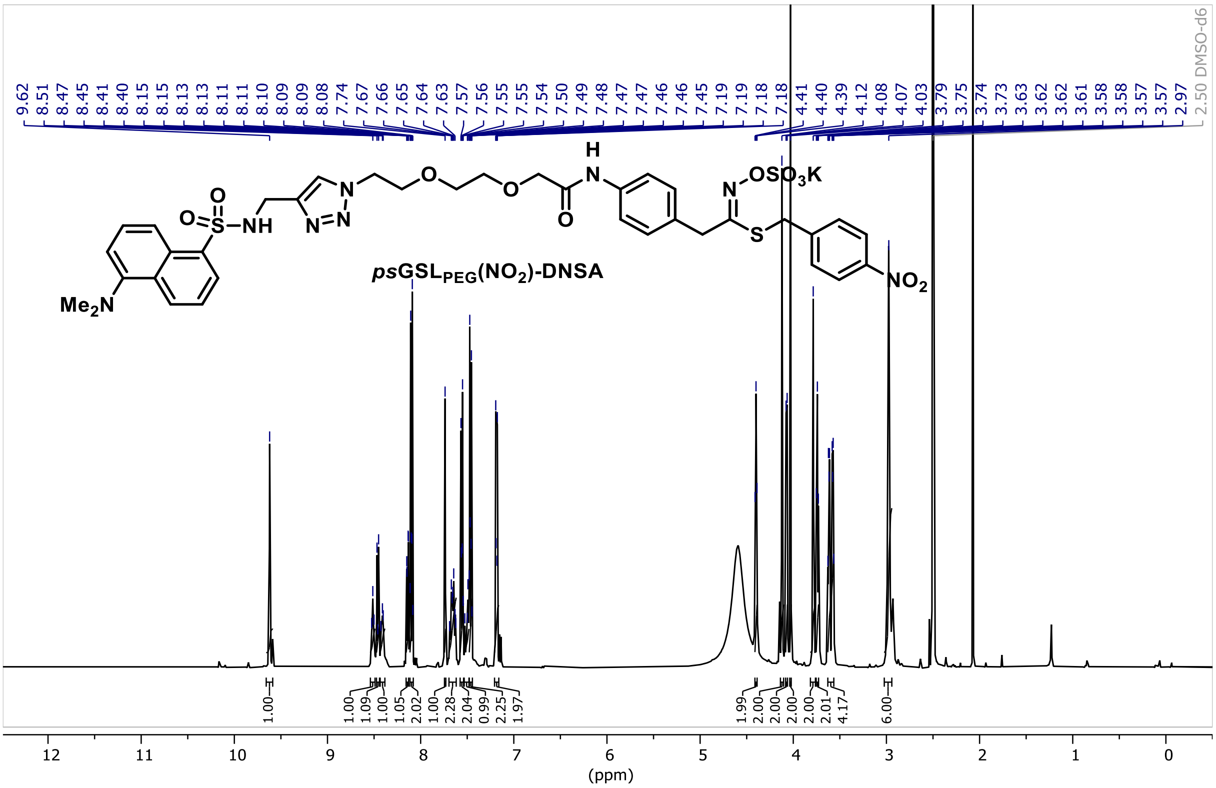

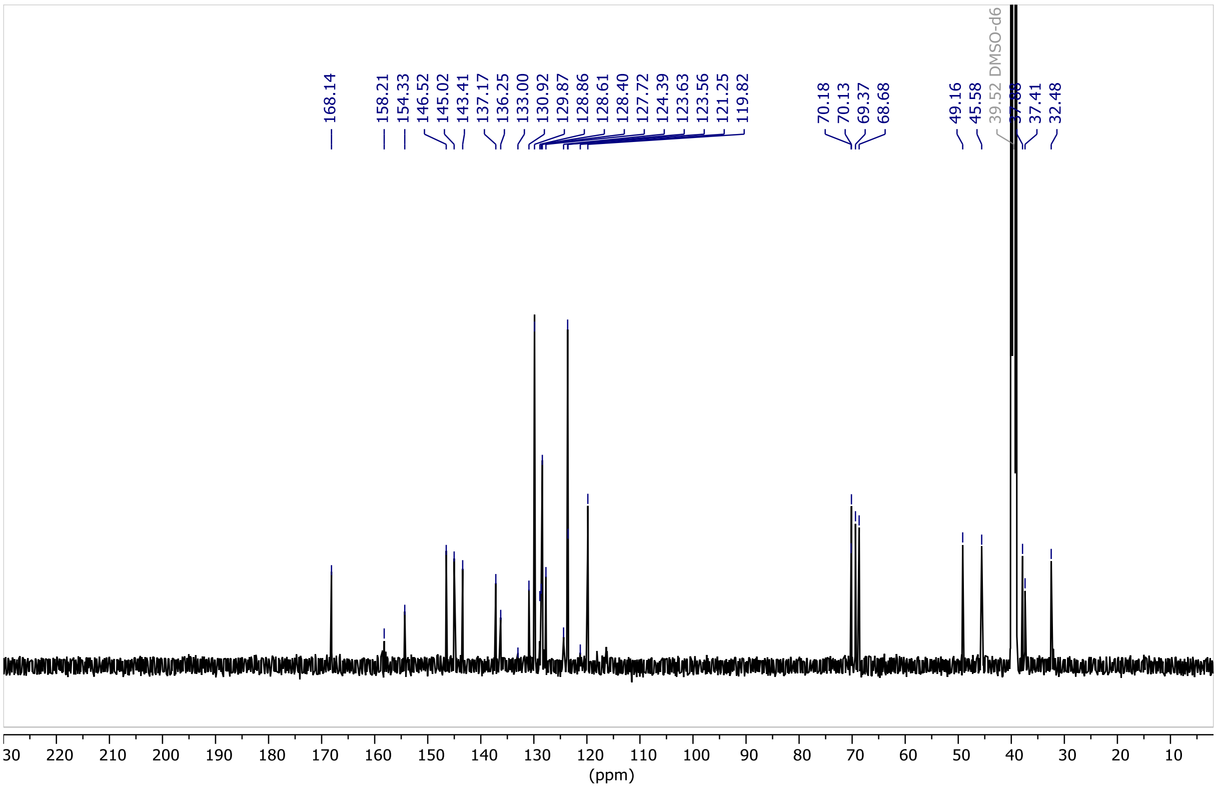

Hex-5-ynal oxime (**31**)

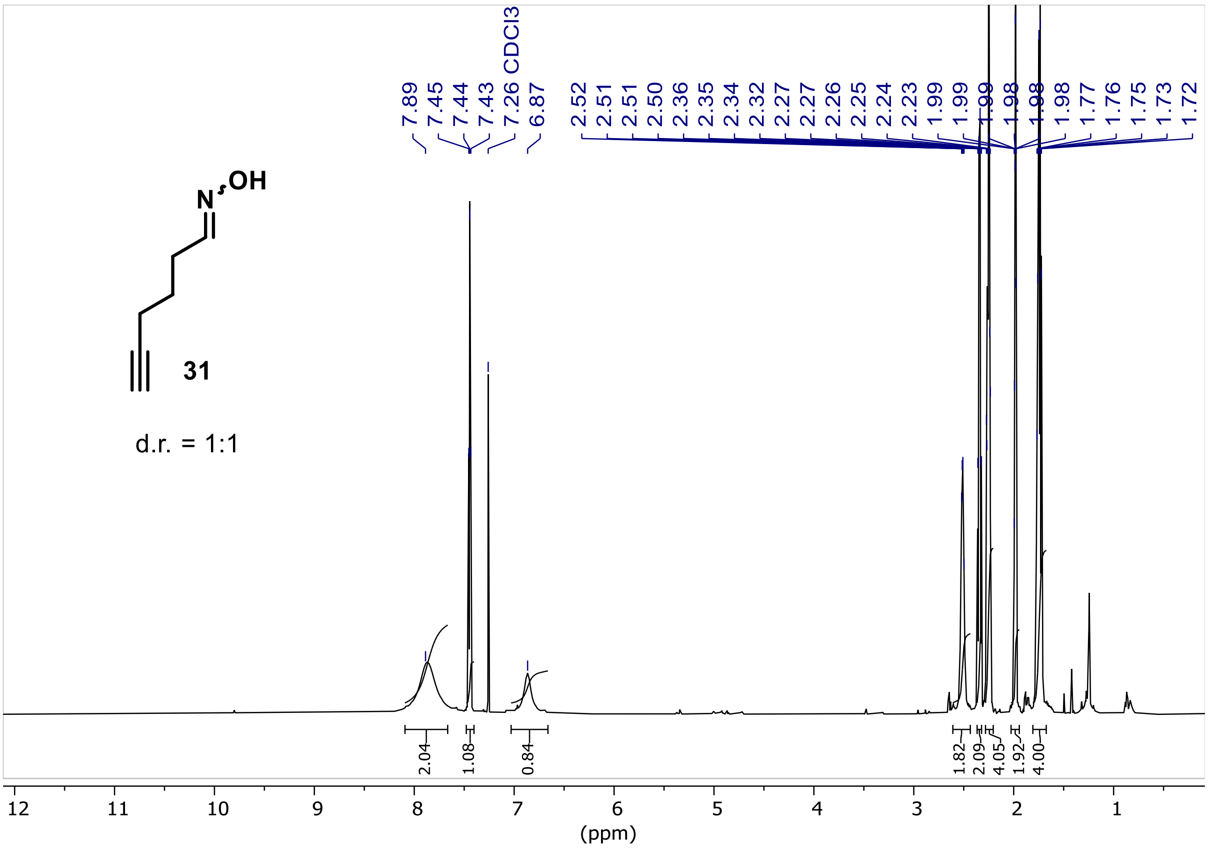

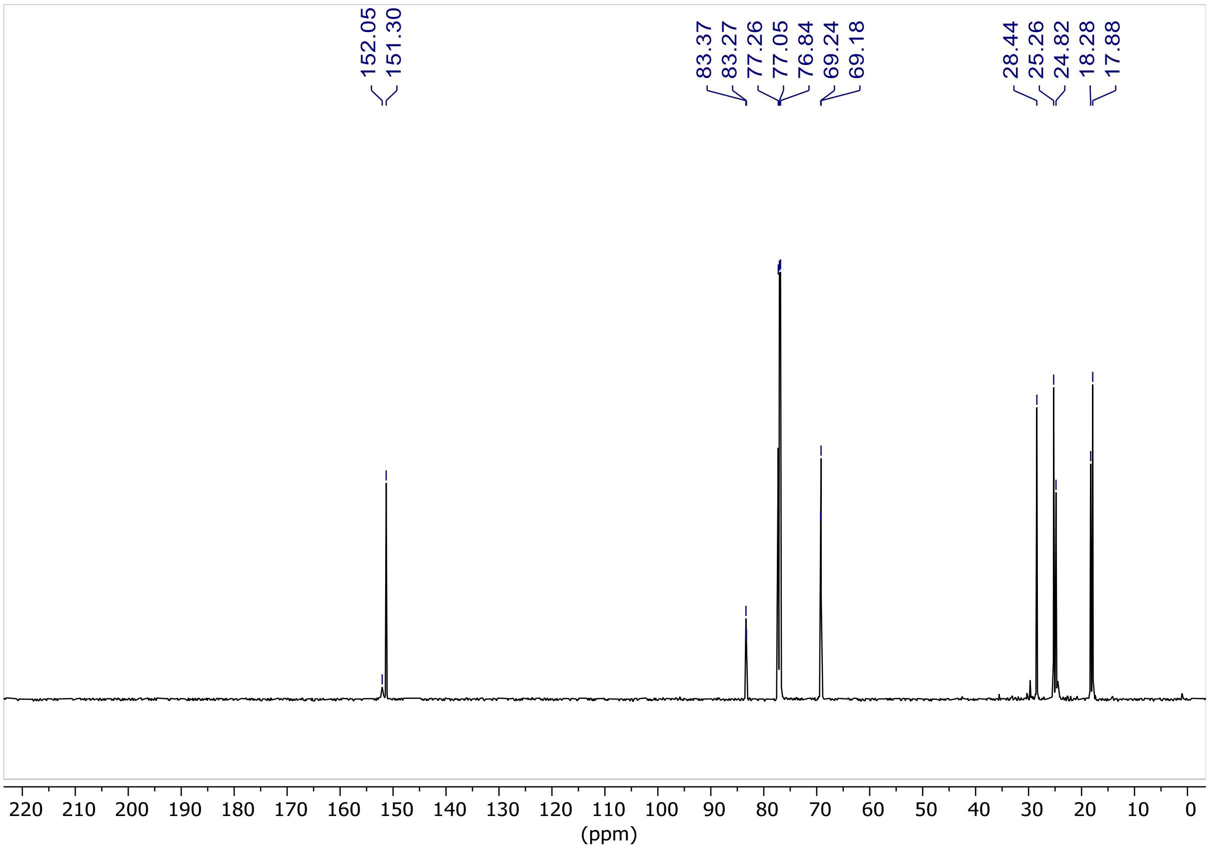

4-Nitrobenzyl (Z)-*N*-hydroxyhex-5-ynimidothioate (**32**)

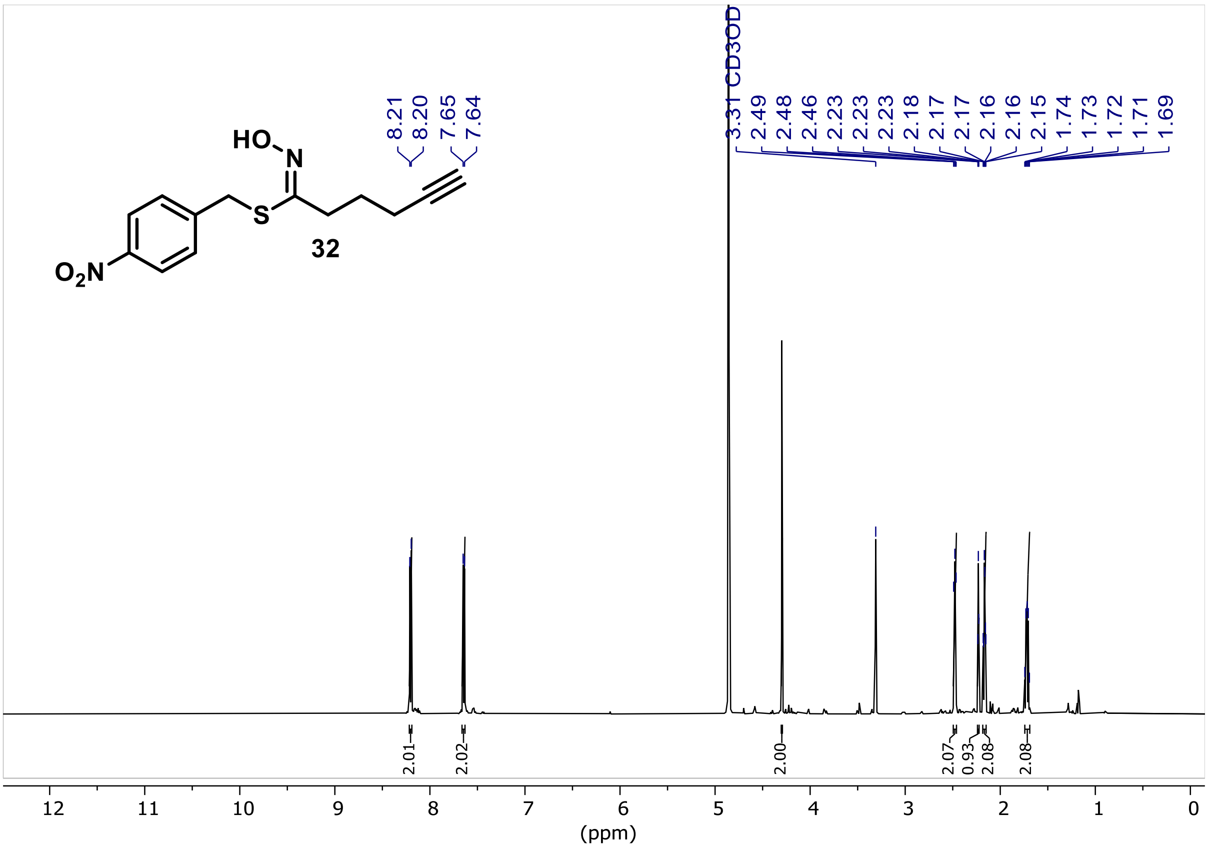

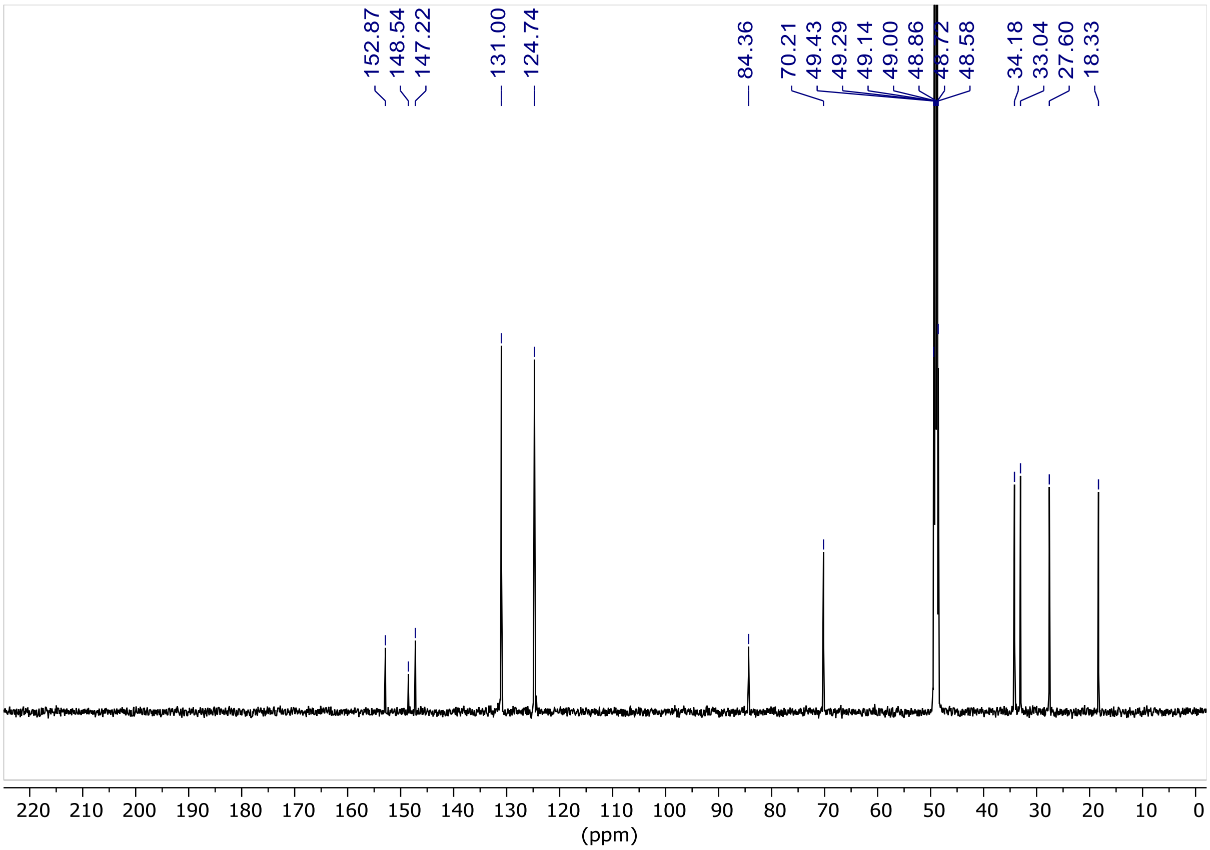

Potassium (Z)-(1-((4-nitrobenzyl)thio)hex-5-yn-1-ylidene)amino sulfate (***ps*GSL(NO₂)-alkyne**)

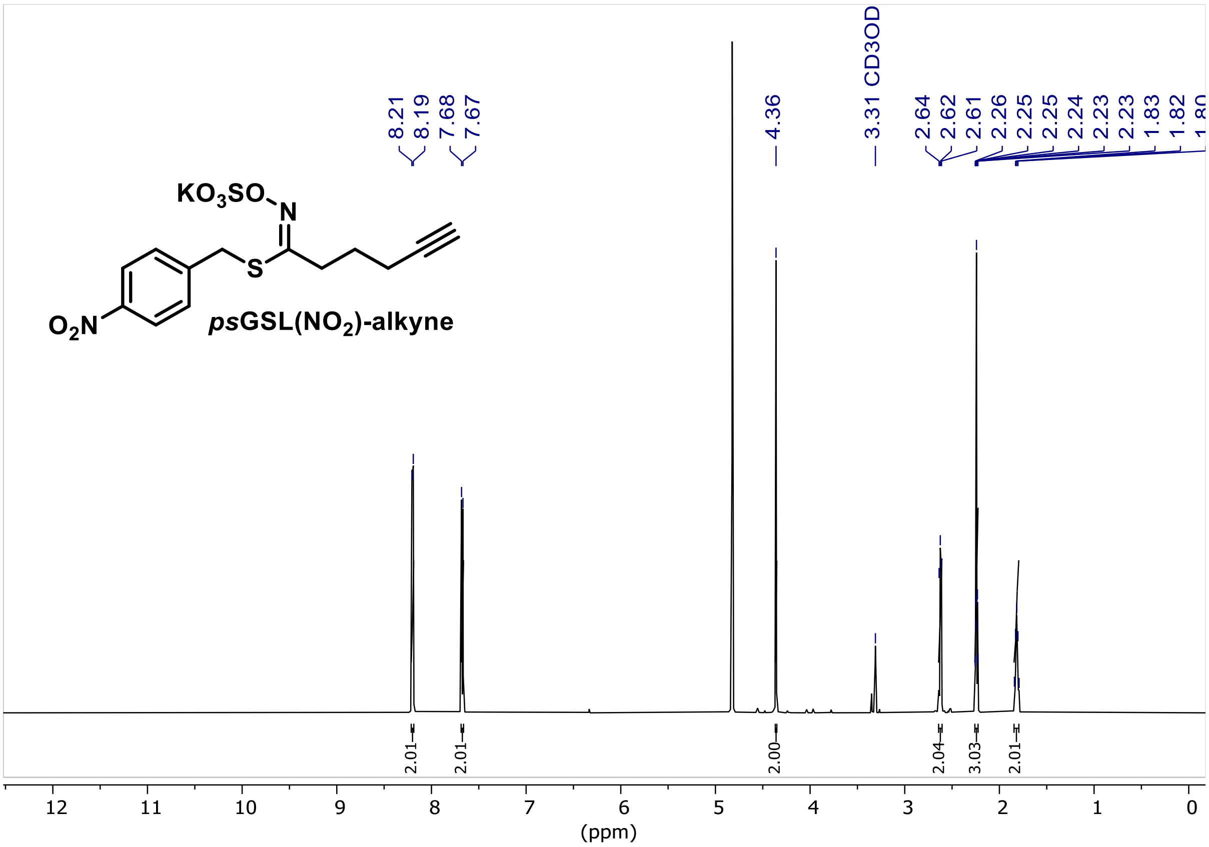

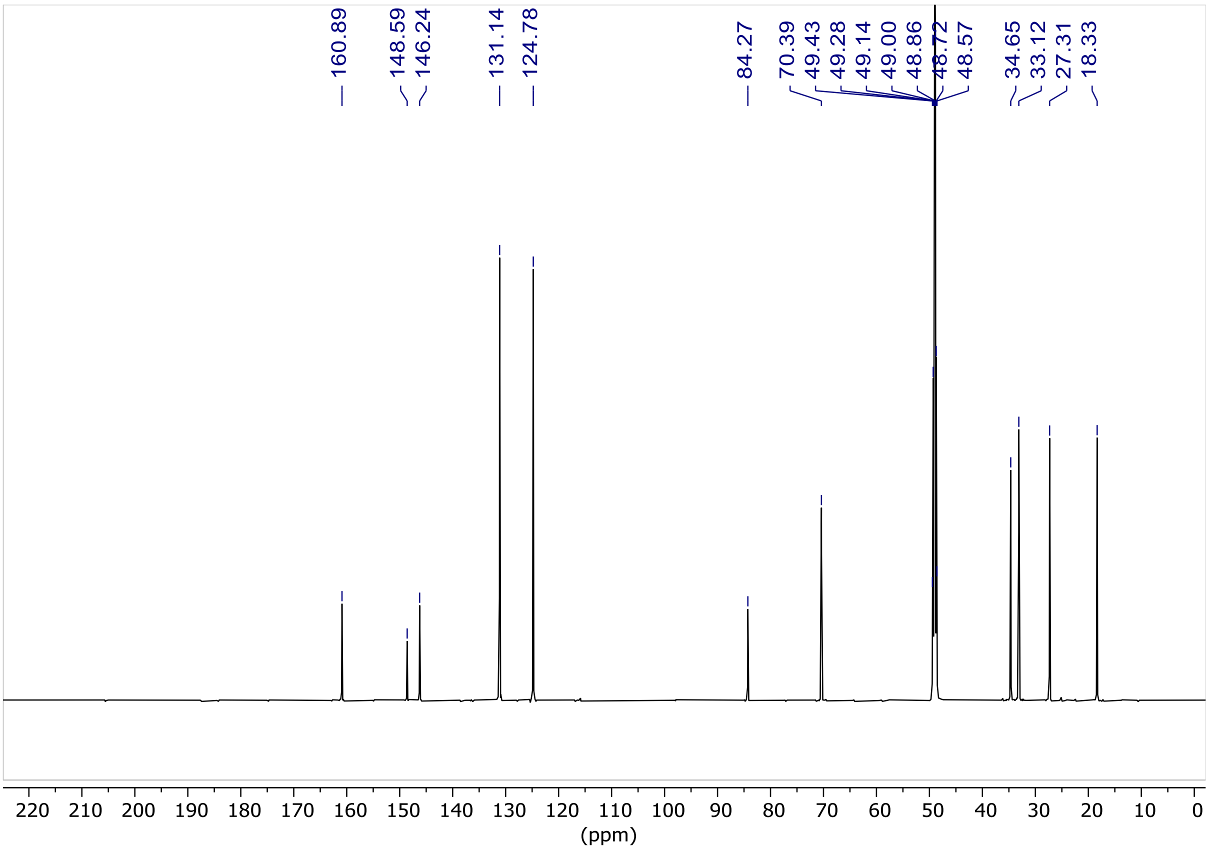

N-(2-(2-(2-(2-azidoethoxy)ethoxy)ethoxy)ethyl)-3-(5,5-difluoro-7,9-dimethyl-5*H*-5λ[4],6λ[4]-dipyrrolo[1,2-*c*:2',1'-*f*][1,3,2]diazaborinin-3-yl)propenamide (**33**)

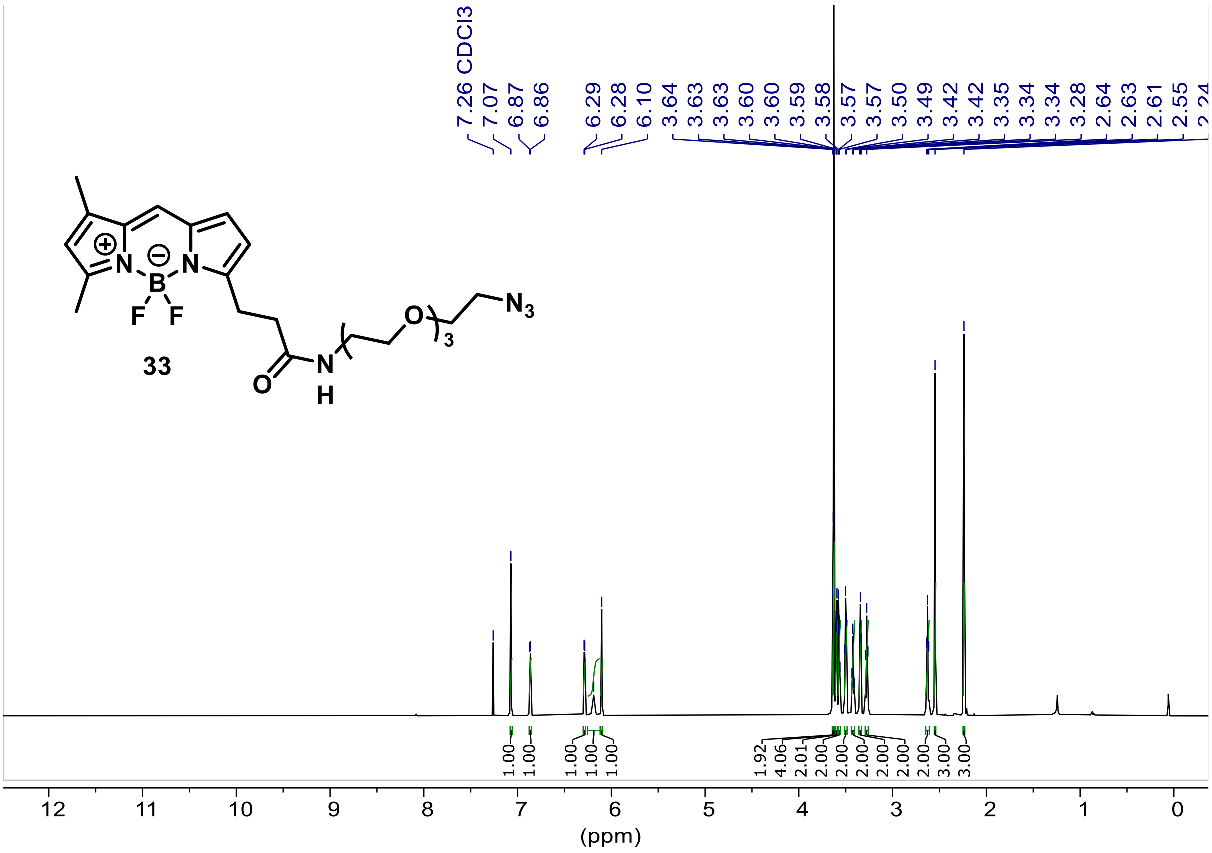

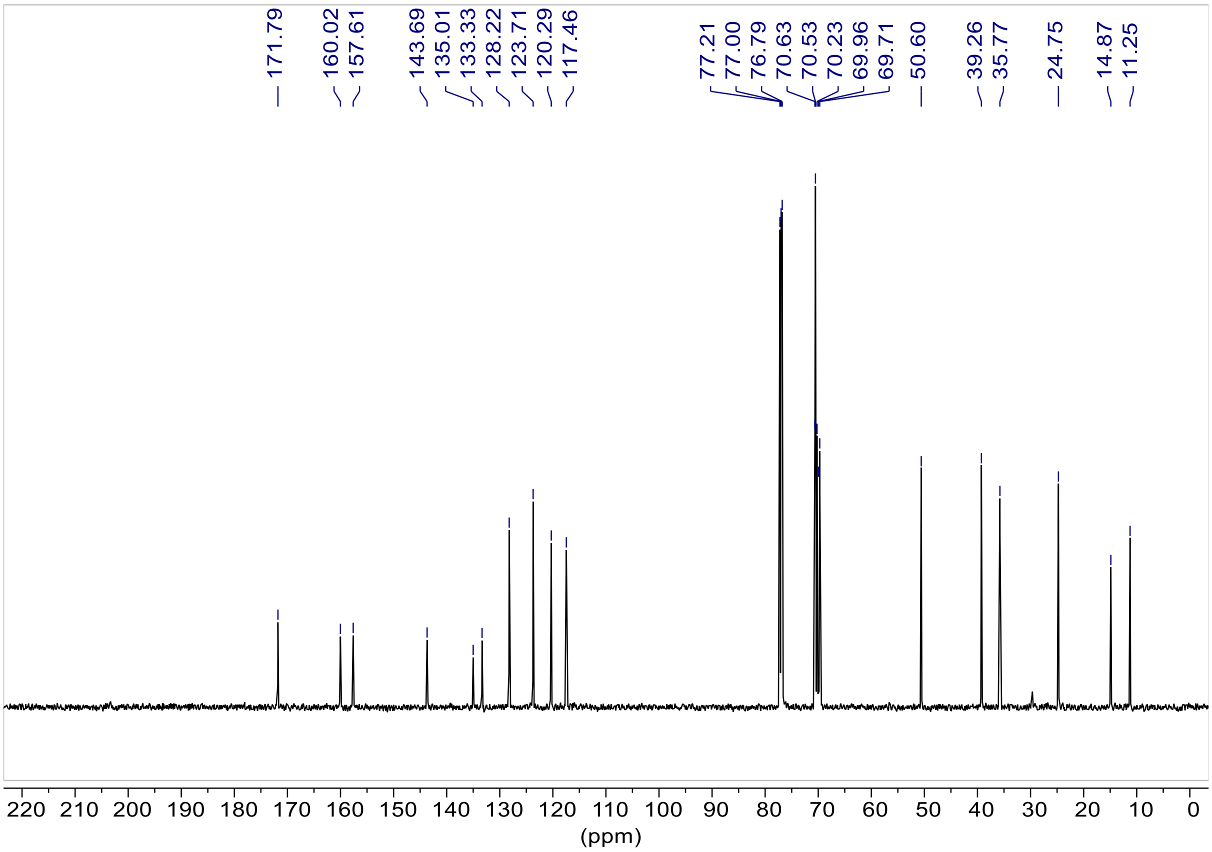

Potassium (*Z*)-(4-(1-(15-(5,5-difluoro-7,9-dimethyl-5*H*-5λ^4,6λ^4-dipyrrolo[1,2-*c*:2',1'-*f*][1,3,2]diazaborinin-3-yl)-13-oxo-3,6,9-trioxa-12-azapentadecyl)-1*H*-1,2,3-triazol-4-yl)-1-((4-nitrobenzyl)thio)butylidene)amino sulfate (***ps*GSL(NO$_2$)-BODIPY$_{FL}$**)

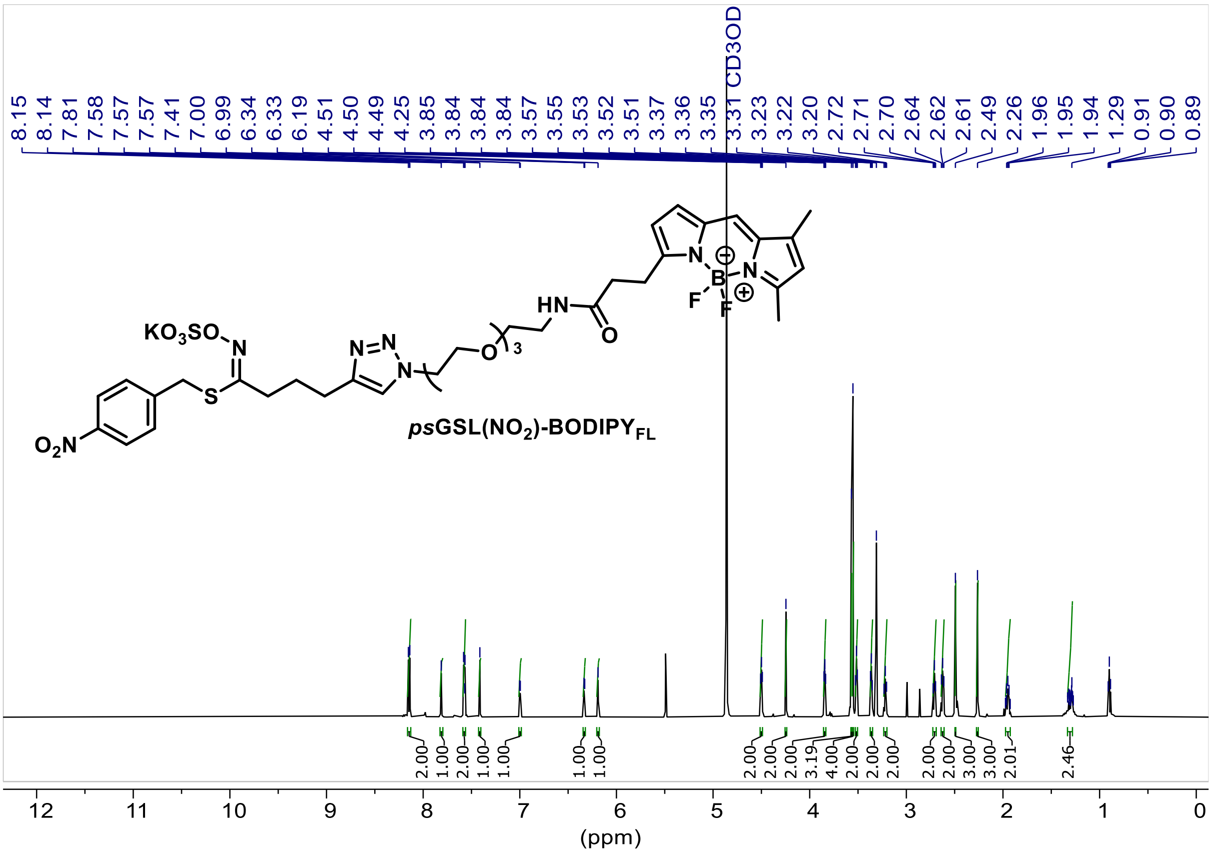

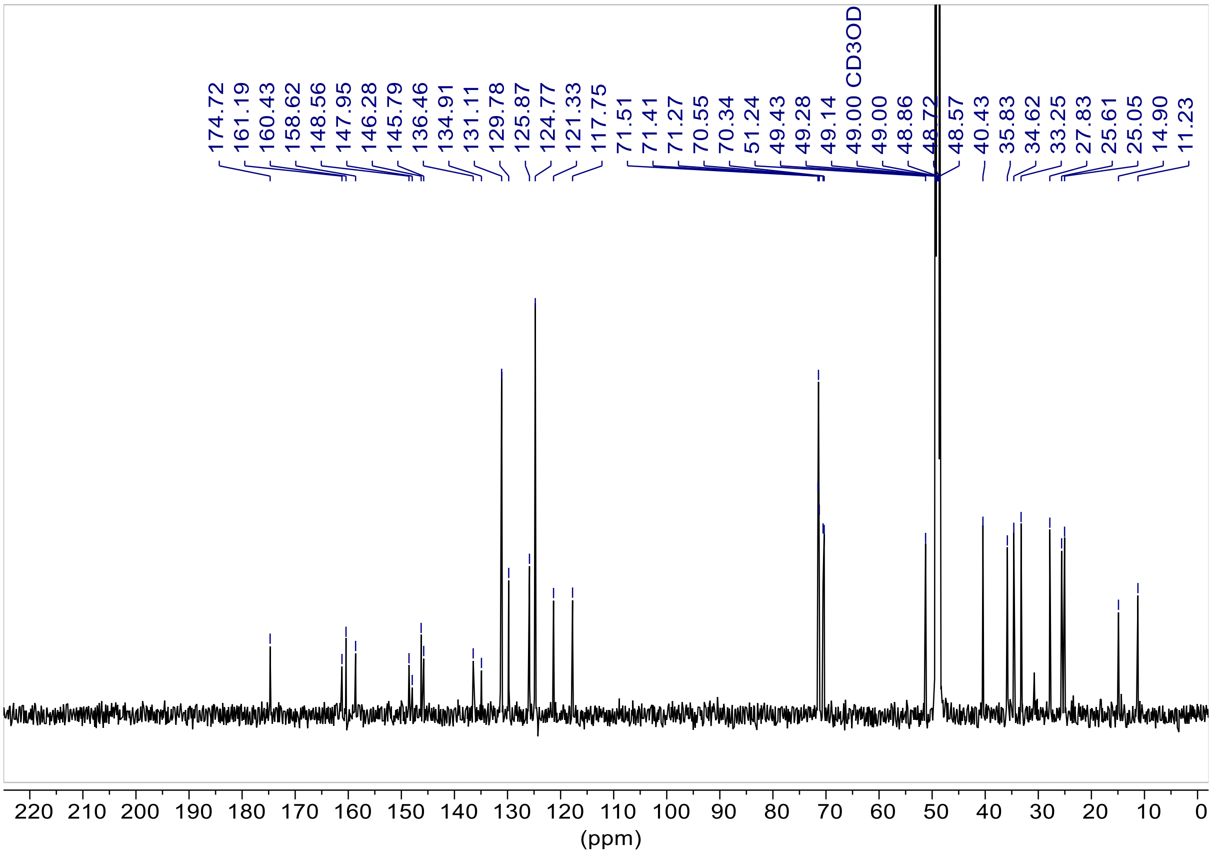

L-Asparaginyl-*L*-alanyl-*L*-cysteinylglycyl-*L*-lysyl-*L*-asparaginyl-*L*-alanylglycyl-*L*-lysine (**Model peptide 1**)

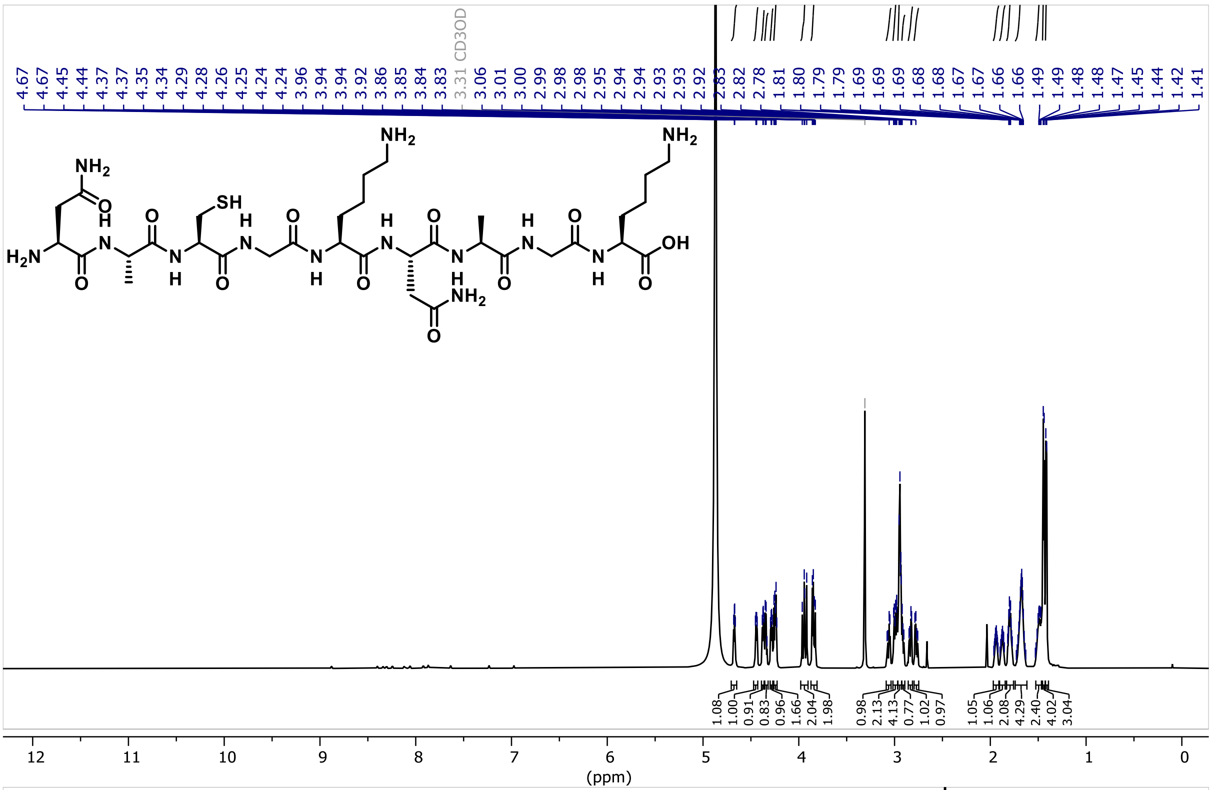

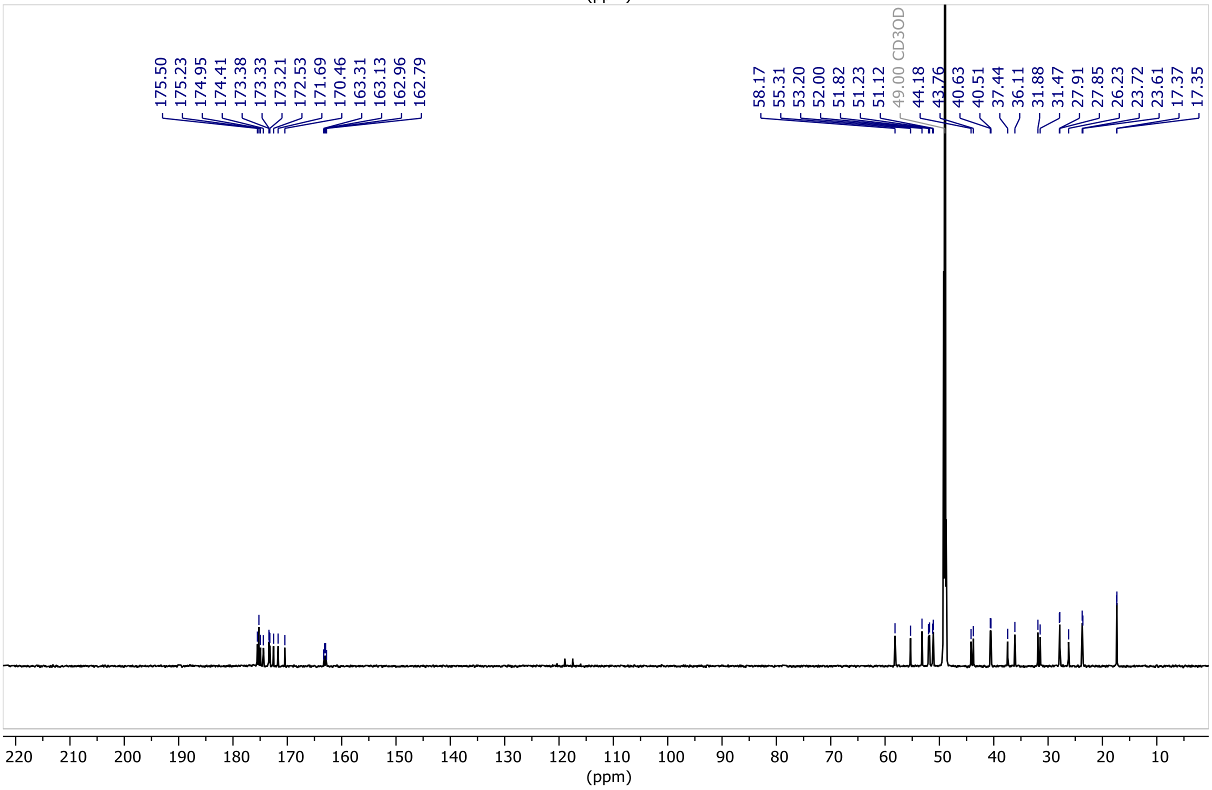

Acetyl-*L*-asparaginyl-*L*-alanyl-*L*-cysteinylglycyl-*L*-lysyl-*L*-asparaginyl-*L*-alanylglycyl-*L*-lysine (**Model peptide 2**)

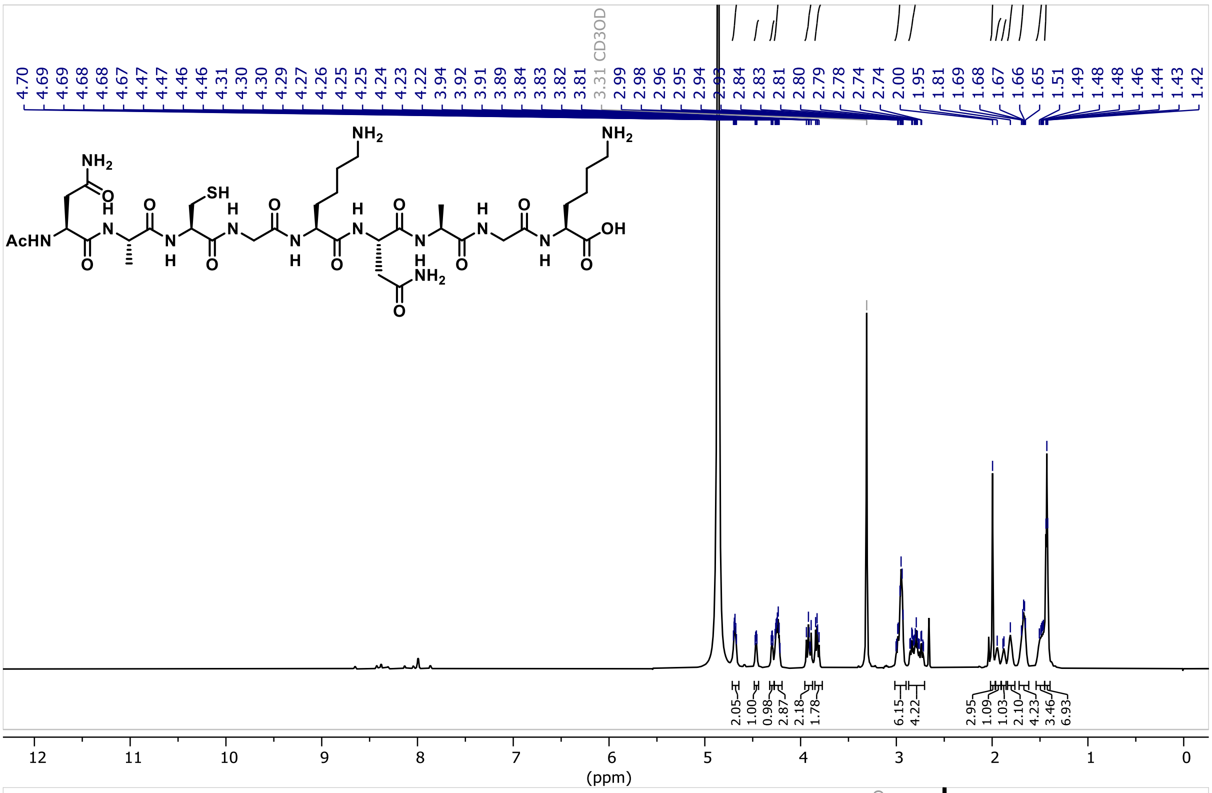

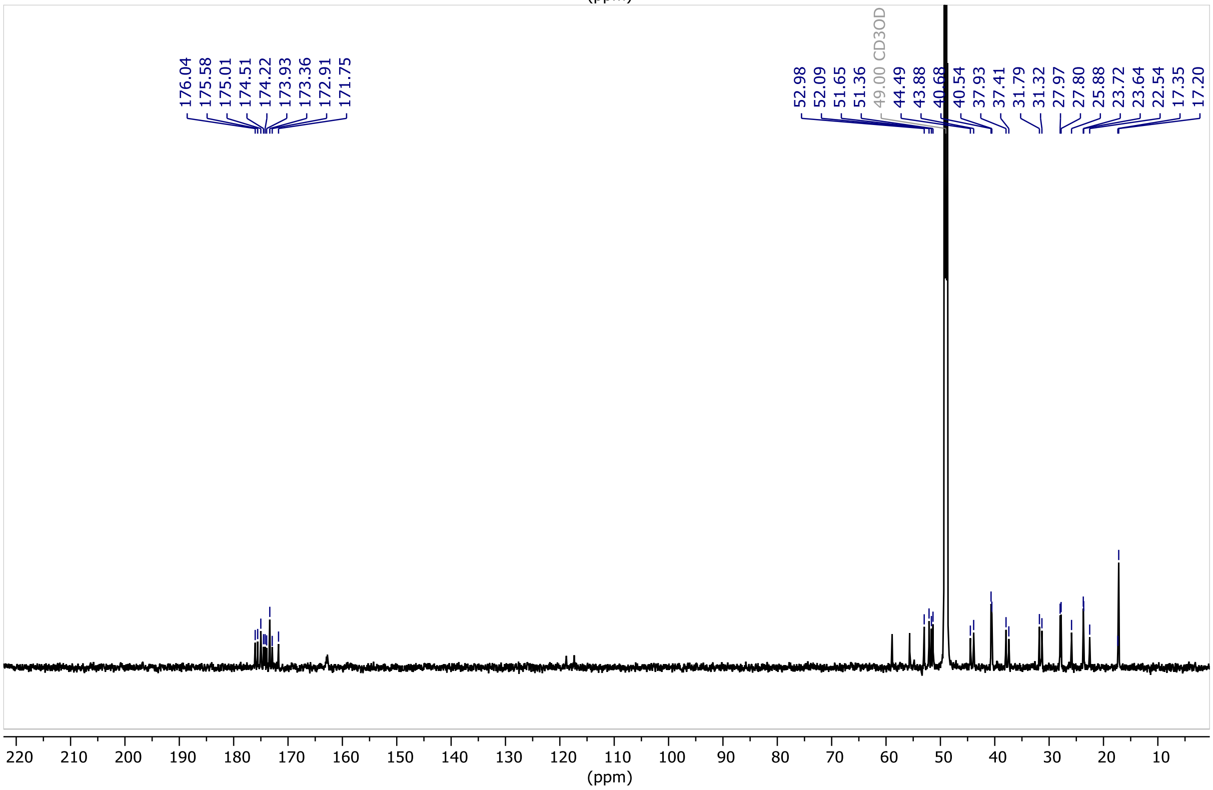

L-Asparaginyl-*L*-alanylglycylglycyl-*L*-lysyl-*L*-asparaginyl-*L*-alanylglycyl-*L*-lysine (**Model peptide 3**)

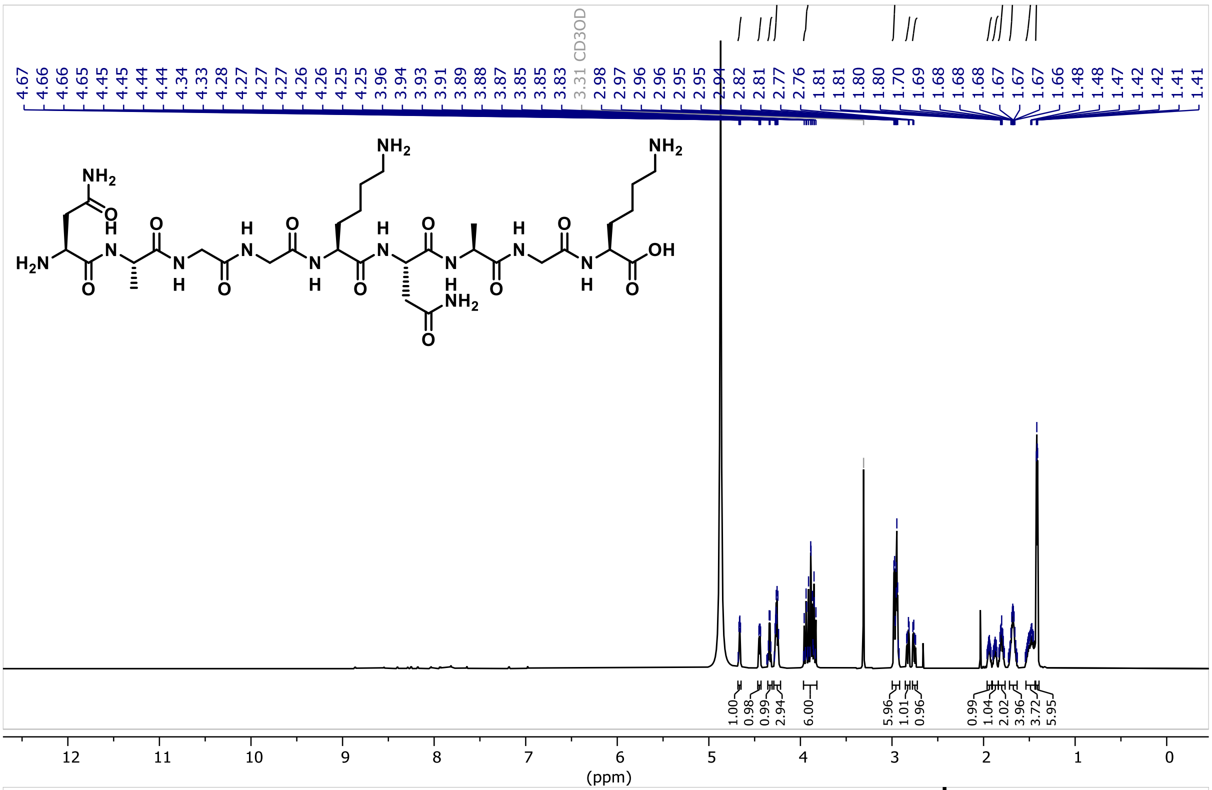

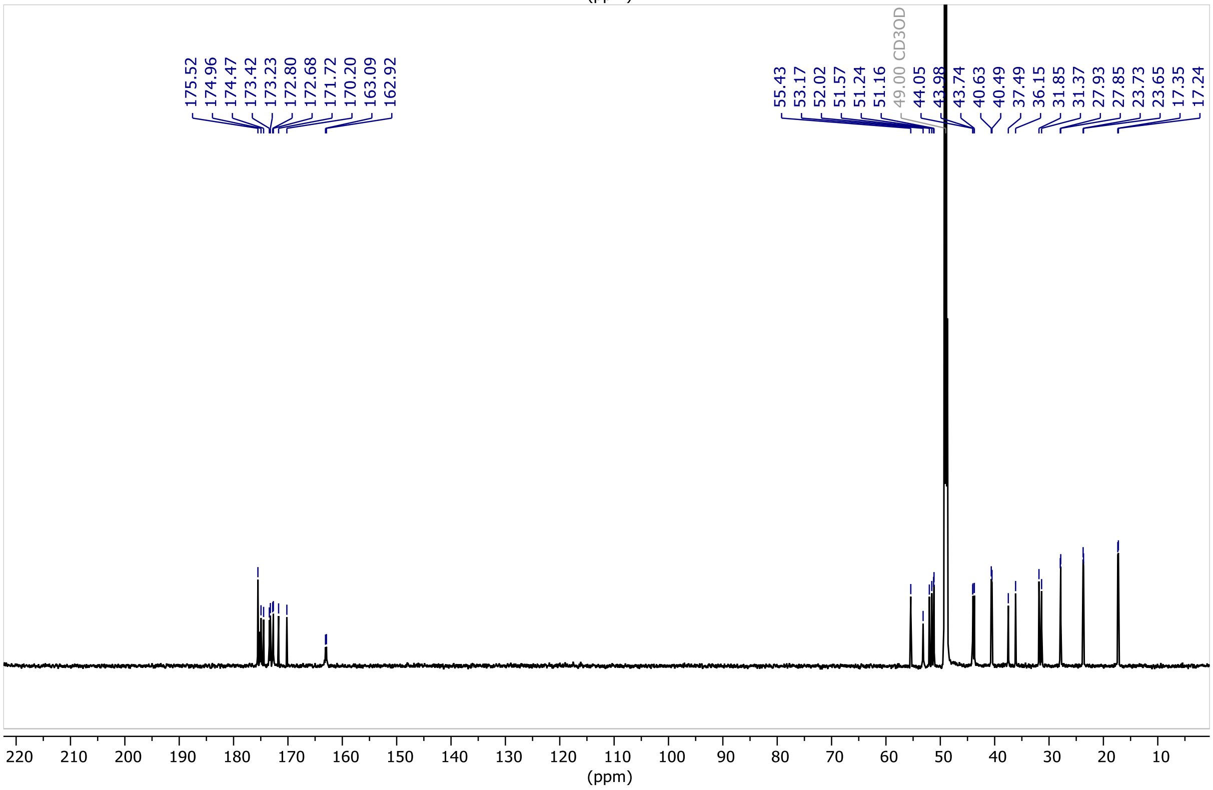

Acetyl-*L*-asparaginyl-*L*-alanylglycylglycyl-*L*-lysyl-*L*-asparaginyl-*L*-alanylglycyl-*L*-lysine (**Model peptide 4**)

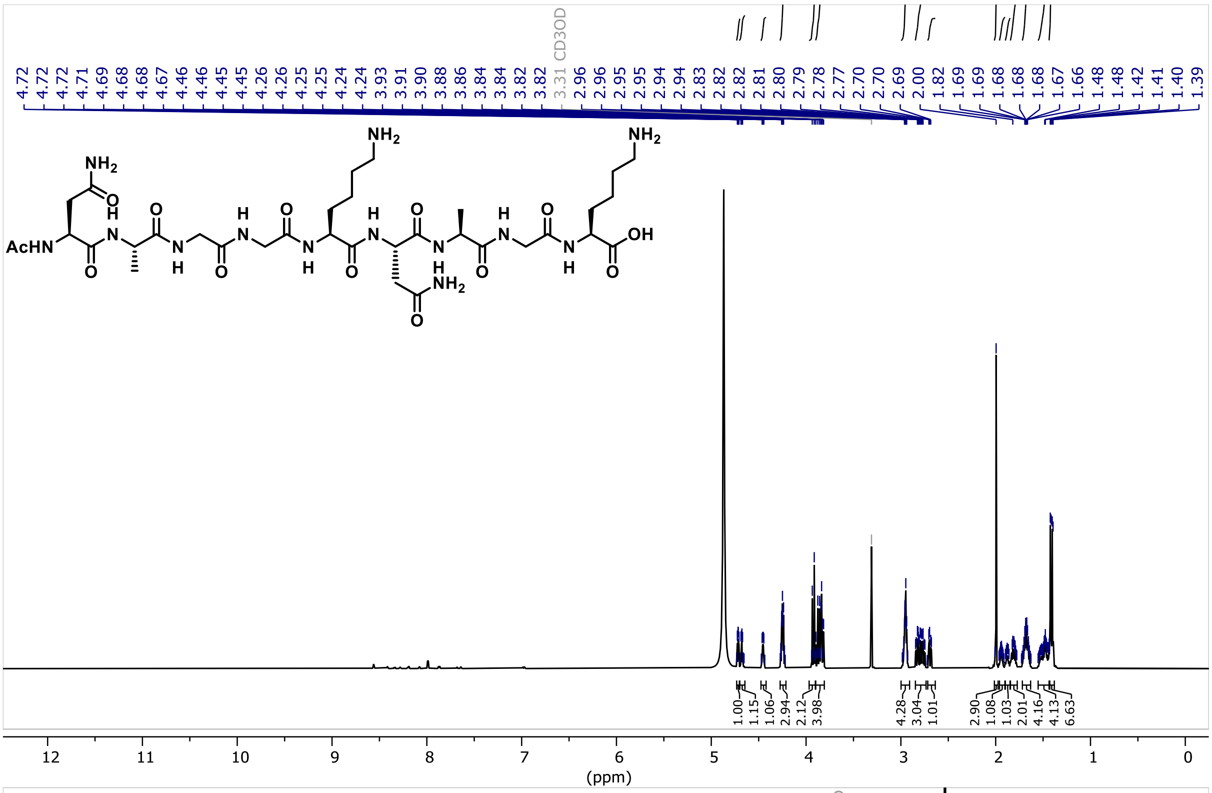

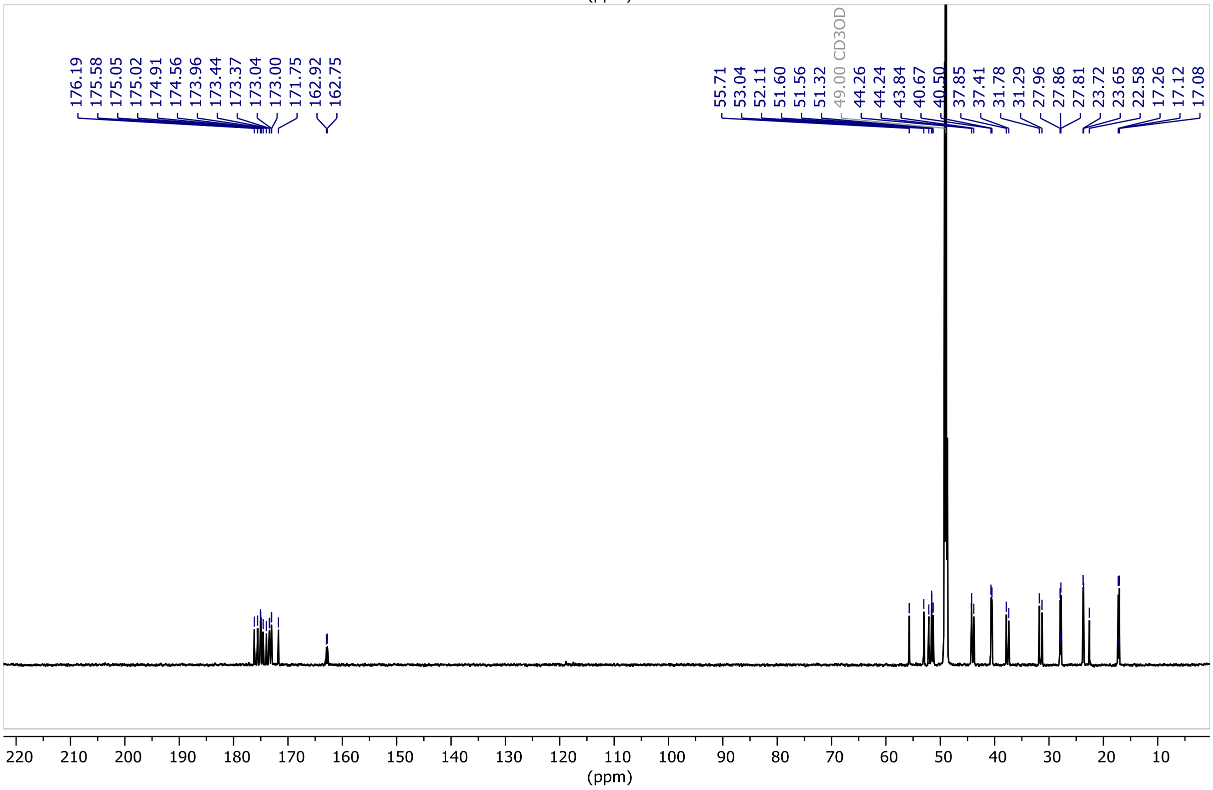

Acetyl-*L*-asparaginyl-*L*-alanyl-*L*-cysteinylglycylglycyl-*L*-asparaginyl-*L*-alanylglycylglycine (**Model peptide 5**)

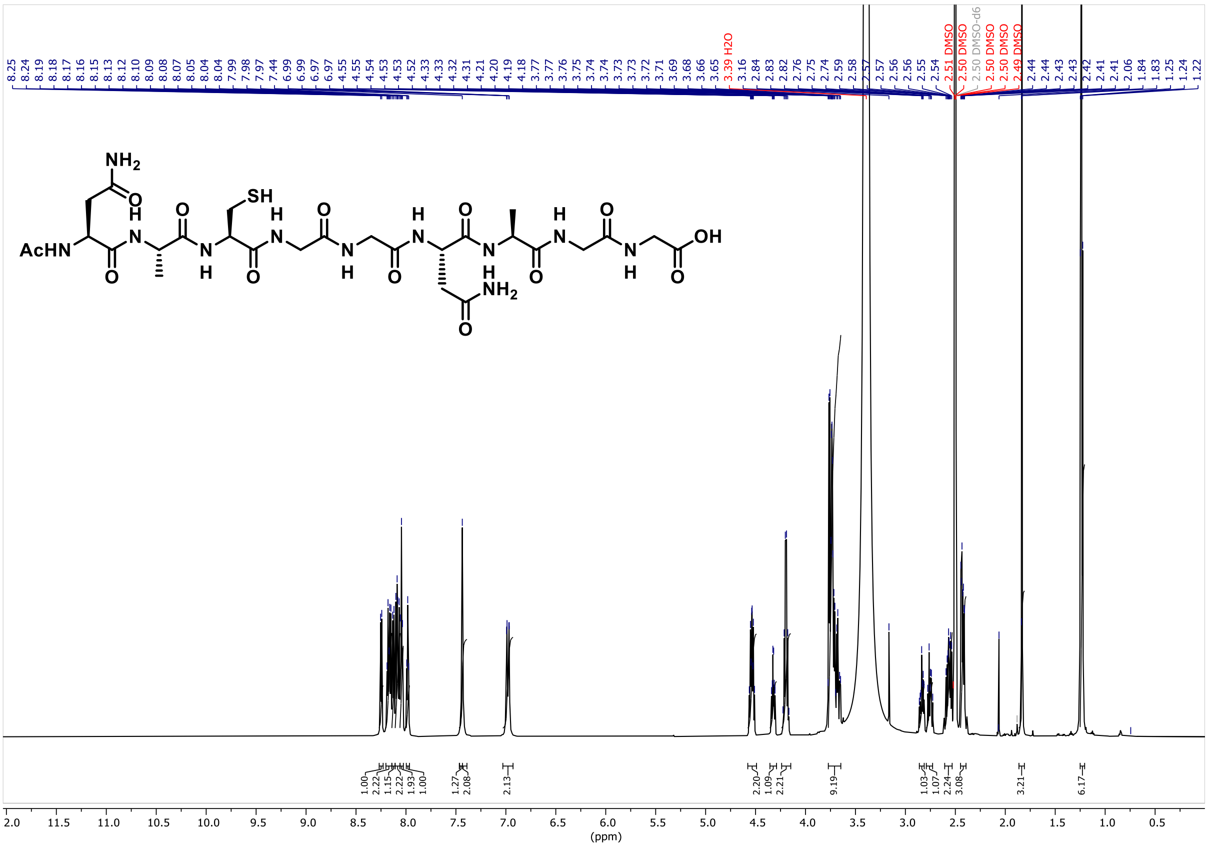

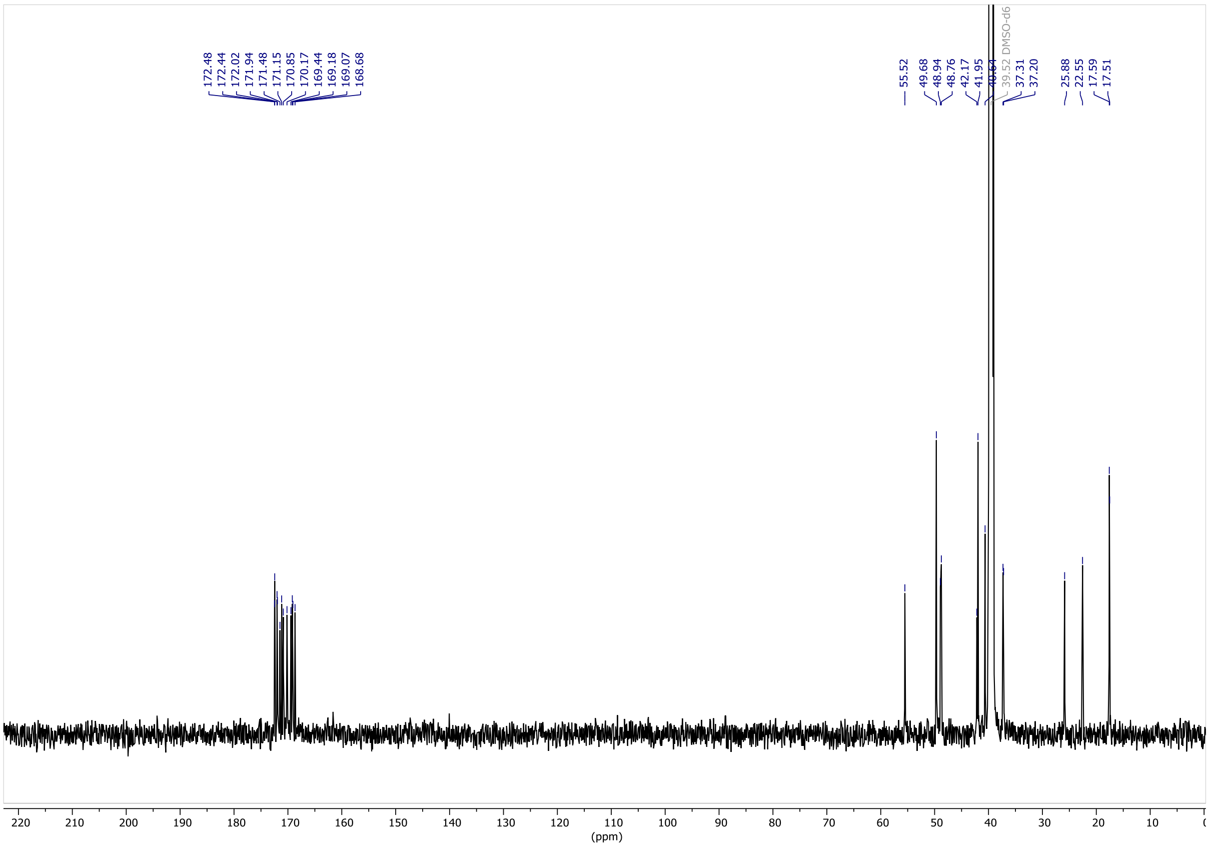

Supplement: Supplementary file 1 — The authors have cited additional references within the Supporting Information [68, 69, 70, 75, 76, 77, 78, 79, 80, 81, 82, 83, 84, 85, 86, 87, 88, 89, 90, 92]. [file CHEM-32-e71012-s001.pdf]
